# Helicase-like transcription factor (*Hltf*) gene-deletion promotes oxidative phosphorylation (OXPHOS) in colorectal tumors of AOM/DSS-treated mice

**DOI:** 10.1371/journal.pone.0221751

**Published:** 2019-08-28

**Authors:** Rebecca A. Helmer, Gurvinder Kaur, Lisa A. Smith, Beverly S. Chilton

**Affiliations:** 1 Department of Cell Biology & Biochemistry, Texas Tech University Health Sciences Center, Lubbock, Texas, United States of America; 2 Department of Pathology, Texas Tech University Health Sciences Center, Lubbock, Texas, United States of America; Università degli Studi della Campania, ITALY

## Abstract

The helicase-like transcription factor *(HLTF)* gene—a tumor suppressor in human colorectal cancer (CRC)—is regulated by alternative splicing and promoter hypermethylation. In this study, we used the AOM/DSS-induced mouse model to show *Hltf*-deletion caused poor survival concomitant with increased tumor multiplicity, and dramatically shifted the topographic distribution of lesions into the rectum. Differential isoform expression analysis revealed both the truncated isoform that lacks a DNA-repair domain and the full length isoform capable of DNA damage repair are present during adenocarcinoma formation in controls. iPathwayGuide identified 51 dynamically regulated genes of 10,967 total genes with measured expression. Oxidative Phosphorylation (Kegg: 00190), the top biological pathway perturbed by *Hltf*-deletion, resulted from increased transcription of *Atp5e*, *Cox7c*, *Uqcr11*, *Ndufa4* and *Ndufb6* genes, concomitant with increased endogenous levels of ATP (p = 0.0062). Upregulation of gene expression, as validated with qRT-PCR, accompanied a stable mtDNA/nDNA ratio. This is the first study to show *Hltf*-deletion in an inflammation-associated CRC model elevates mitochondrial bioenergetics.

## Introduction

Colorectal cancer (CRC)—the second leading cause of cancer-related deaths in a combined population of men and women in the United States [1]—can originate proximal to the splenic flexure (right-sided CRC), or distal to that anatomic landmark (left-sided CRC). Increased left-sided CRC in men and women under the age of 50 drives poor survival statistics despite the availability of screening tools. Patients with inflammatory bowel disease that constitute the greatest at risk population are the only ones recommended for screening at this age. For these individuals, their CRC derives from the pro-neoplastic effects of severe, chronic inflammation [2]. About 95 percent of CRCs are adenocarcinomas initiated via genomic instability [3, 4]. Tumor suppressor genes are targets of genetic/epigenetic alteration as part of this process. For example, inactivating mutations in the adenomatous polyposis coli (*APC*) tumor suppressor gene characterize 70–85% of all human colon tumors [5]. The helicase-like transcription factor (*HLTF*) tumor suppressor gene is hypermethylated during the transition from aberrant crypt focus to adenoma/polyp. Epigenetic silencing of *HLTF* characterizes 43% of primary CRC tumors [6]. The methylation status of *HLTF* DNA in serum and stools is a biomarker in CRC cancer patients and a therapeutic predictor of patient outcome [7–10]. However, its relationship to CRC localization is unknown.

Rodent models for CRC have provided unique insights to the function of *Apc* [11] and *Hltf* [12]. For more than 20 years, the *Apc*^*Min/+*^ mouse has been an experimental model for human familial adenomatous polyposis [11]. However, unlike the human condition, the tumor burden in the *Apc*^*Min*^ mouse is in the small intestine rather than the large intestine [13]. Tumors in the *Apc*^*Min/+*^ mouse occur at a small intestine-to-colon ratio of 40:1, and the inflammation profiles of the two tissues differ [14, 15]. Colonic tumors have more infiltrating immune cells and cytokine expression. The *Apc*^*Min/+*^ mouse has a gender-specific phenotype for tumor multiplicity such that female mice developed more tumors in the small intestine, and male mice developed more colon tumors [16–18]. The loss of *Hltf* function does not drive oncogenesis in the gastrointestinal tract. However, *Hltf* deficiency on the Apc^min/+^ mutant background produced genomic instability in colon tumor cells [12].

The alkylating agent azoxymethane (AOM) is one of the most frequently used carcinogens to induce tumor development in the colon [19–23]. Although alkylating agents are unlikely contributors to the development of human sporadic CRC, the AOM-treated mouse is a model for some of the molecular events that occur in human CRC. The combination of AOM and the proinflammatory agent DSS shortens the latency period about 3-fold compared to spontaneous colorectal tumor progression. The AOM/DSS model increases the multiplicity of colorectal tumors, and is a model for the process of tumorigenesis driven by inflammatory bowel disease.

In this study, we combined our global *Hltf*-deleted mouse model [24–26] with AOM/DSS-treatment, to probe the effects of *Hltf* loss of expression on survival, colorectal tumor localization, multiplicity, morphology, and gene expression. To eliminate gender bias as well as genetic background effects [27], we used male mice congenic on the C57Bl/J6 background. The AOM/DSS-treatment recapitulate the aberrant crypt foci—adenoma—carcinoma progression found in human CRC [19]. Our findings are particularly relevant to individuals with inflammatory bowel disease (ulcerative colitis, Crohn’s disease) and an increased risk of developing tumors in the distal colon or rectum.

## Materials and methods

### Reagents and kits

Abcam (Cambridge, MA) was the source of the luminescent ATP detection assay kit (ab113849). Isolation of genomic DNA from tail biopsies and tumor samples was achieved with the DNeasy Blood & Tissue Kit (69506) purchased from Qiagen (Valencia, CA). Applied Biosystems TaqMan^®^MGB (minor groove binder) probes with a 5´ reporter (FAM or VIC) and a 3´ nonfluorescent quencher (MGB-NFQ), Invitrogen^™^TRIzol^™^ Reagent (15596026), Invitrogen SuperScript^™^Vilo^™^cDNA Synthesis Kit (11754050), and SequalPrep^™^ Long PCR Kit with dNTPs (A10498) were purchased from ThermoFisher Scientific (Waltham, MA). Midland Certified Reagent Company (Midland, TX) synthesized the PCR primers. OmniPur agarose (2120) was purchased from Calbiochem division of EMD4Biosciences (San Diego, CA). Promega (Madison, WI) was the source of the Lambda DNA/EcoRI + HindIII agarose gel markers (G173A). Azoxymethane (A2853) was purchased from Sigma-Aldrich (St. Louis, MO) and dextran sulfate sodium salt colitis grade (160110) was purchased from MP Biomedicals (Santa Ana, CA). Mouse Mitochondrial DNA copy number assay kit (MCN 3) was purchased from Detroit R&D, Inc. (Detroit, MI). Alcian-blue staining solution was purchased from EMD Millipore Corp (Burlington, MA). All protocols are accessible in protocols.io (dx.doi.org/10.17504/protocols.io.4argsd6).

### *Hltf*-deleted and control mice

The development of global *Hltf*-deleted mice in collaboration with genOway (Lyon, France) was previously described [24–26]. Mice are fully congenic (N11) on the C57BL/6J genomic background. Global *Hltf*-deleted mice presented a neonatal lethal phenotype [24–26]. *Hltf*-deleted mice and their littermate controls (Hltf +/+) breathed freely at birth, and acquired a characteristic pink color suggesting normal lung and diaphragm function. Collectively, they displayed a sucking reflex, drank immediately after birth, and milk was always visible in their stomachs. However, 6–8 hours postpartum three of four pups were hypoglycemic. *Hltf*-deleted pups developed progressive cyanosis and became moribund. Postpartum death occurred at the same frequency in *Hltf*-deleted mice from both heterozygous (+/-) and homozygous (-/-) mothers [24] thus eliminating the maternal genotype as a confounding factor. Neonatal hypoglycemia is not caused by the placenta [26]. PCR screening reactions authenticated the *Hltf*-deleted and wild type control genotypes. Mice were housed with a 12:12 light/dark cycle with access to food and water *ad libitum* and bedding was changed 2–3 times/week. Routine testing of sentinel mice ensured the colony was disease free. All studies and the anticipated mortality were conducted in accord with the NIH Guidelines for the Care and Use of Laboratory Animals, as reviewed and approved by the Animal Care and Use Committee at Texas Tech University Health Sciences Center (NIH Assurance of Compliance A3056-01; USDA Certification 74-R-0050, Customer 1481, S1 Checklist). TTUHSC’s IACUC specifically approved this study. BSC and veterinary staff monitored the mice at least twice daily. Mice experienced increased stooling with stool consistency ranging from normal to soft but formed, and in a few instances very soft. There was no incidence of diarrhea or dysenteric diarrhea. Trace amounts of blood associated with rectal prolapse were evident in cage litter toward the end of the study. Monitoring mice for anemia included the pallor or non-pink condition of their footpads in conjunction with their behavior. All efforts were made to minimize pain and suffering (S2 Checklist), i.e. if mice experienced a sustained weight loss of 20%, or became lethargic with evidence of piloerection and poor grooming they were removed from the study and immediately euthanized (CO_2_ followed by cervical dislocation). Euthanasia was preferred to drug therapy with the potential to alter the tumor transcriptome.

To recreate the aberrant crypt foci—adenoma—carcinoma sequence in human CRC [19–23], randomly selected six- to eight-week old *Hltf*-deleted (n = 109) and control (n = 47) male mice were given a single intraperitoneal (IP) injection of 10 mg/kg AOM, a potent carcinogen used to induce colon cancer in rodents. All experimental protocols began at 8 AM in the LARC housing facility. Mice received the first of four cycles of 3% DSS *ad libitum* after 3-days of recovery. Each cycle lasted five days followed by a 16-day recovery period except for the last cycle in which mice were sacrificed 26-days after the last DSS treatment. Mice were weighed every seven days throughout the 97-day treatment protocol.

### Techniques

The entire colon (from the cecum to the anus) for each mouse was excised, flushed in a physiologically accurate direction (proximal to distal) with ice-cold phosphate buffered saline, and cut open longitudinally along the mesenteric side. Alcian blue dye (1%) highlighted the texture of the colon and demarcate the borders of individual tumors [23]. Each colon was subdivided into three roughly equal regions, i.e. proximal colon (with rugae), mid or central colon, and distal (colon/rectum) colon. Tumors were counted and measured (BSC) with a dissecting microscope fitted with an eyepiece reticle. Measurements were confirmed (LAS) in histological preparations.

Some colons were rolled with the mucosa outwards [28], or tumors were snared with forceps and carefully separated from the muscularis mucosae. After histological processing (fixed in 4% paraformaldehyde, paraffin embedded, sectioned @ 3–4 μm, and processed for staining), all lesions were evaluated (double-blind) by LAS and BSC, and scored for degree of dysplasia, lymphocytic response, and invasive colorectal carcinoma (LAS). Human criteria were used for tumor staging with stage 0 the earliest stage followed by a range from I through IV. For a diagnosis of stage 1, the cancer had grown through either the muscularis mucosa into the submucosa (pT1) or the muscularis propria (pT2), but not invaded local lymph nodes or distant sites.

Blood glucose was monitored with OneTouch Ultra Mini and OneTouch Ultra Mini Blue test strips from LifeScan (Malpitas, CA), a Johnson & Johnson Company. Serum glucose from non-fasting males was tested with blood from a tail-snip prior to euthanasia. Luminescent ATP detection assay (96-well plate) was performed according to the manufacturer’s instructions with tumor samples from *Hltf*-deleted (n = 8) and control (n = 8) mice. DNA was quantified with a PicoGreen dsDNA assay. A Kaplan-Meier survival plot (log-rank Mantel-Cox test, p<0.05) and hazard ratio (Manel-Haenszel) were calculated for *Hltf*-deleted (n = 109) and control (n = 47) mice. With the exception of RNA-seq data analyses, all statistical procedures were performed with GraphPad Prism v8.1.1 (significance, p<0.05).

### Tumor transcriptome analysis (RNA-seq)

Individual samples (tumors from an individual mouse/sample x 3 biological replicates for *Hltf*-deleted and control male mice = 6 total samples) were flash frozen and sent to Otogenetics Corp. (Norcross, GA) for RNA-seq assays as previously described [24–26]. Briefly, total RNA was isolated, and evaluated for its integrity and purity with an Agilent Bioanalyzer (Table 1). RNA samples were rRNA-depleted prior to Illumina library preparation and sequencing (HiSeq2500). Paired-end 106 nucleotide reads (Table 1) were mapped against the reference genome mm10 with star2.4.0j. Comparison of expression level (fpkm_tracking) for differential expression (DE) analysis as well as alternative splicing (isoform) analysis was done with cufflinks.cuffdiff (2.2.1). All RNA-seq data in this publication are accessible through NCBI’s Gene Expression Omnibus (GEO) Series accession number GSE132814 (https://www.ncbi.nlm.nih.gov/geo/query/acc.cgi?acc=GSE132814). DE data were imported to iPathwayGuide (Advaita Corporation, Plymouth, MI) to utilize a systems biology analysis that considers the role, type, function, position and interactions of each gene in various pathways to identify significantly impacted genes in a specific condition. Standard enrichment parameters (0.6, p<0.05) were used for bioinformatics analyses.

**Table 1:** Sample quality control and RNA-seq outcome.

Sample ID	OD260/280	RIN^a^	Total Bases	Total Reads
1-*Hltf*-deleted	2.12	6.8	3,189,918,844	30,093,574
2-*Hltf*-deleted	2.07	8.0	3,860,089,428	36,415,938
3-*Hltf*-deleted	2.09	8.3	3,136,679,496	29,591,316
4-Control	2.09	6.8	2,341,766,840	22,092,140
5-Control	2.07	8.0	3,005,725,824	28,355,904
6-Control	2.12	7.3	3,126,388,380	29,494,230

^a^An RNA integrity number (RIN) from an Agilent Bioanalyzer

iPathwayGuide scored pathways with the Impact Analysis method that uses two types of evidence: the over-representation of DE genes in a given pathway and the perturbation of that pathway computed by propagating the measured expression changes across the pathway topology. Evidence from two independent probability values was combined into a unique pathway-specific p-value. Underlying pathway topologies, comprised of genes and their directional interactions, were obtained from the following: Kyoto Encyclopedia of Genes and Genomes (KEGG) database (Release 84.0+/10-26, Oct 17), gene ontologies from the Gene Ontology Consortium database (2017-Nov6), miRNAs from the miRBase (Release 21) and TARGETSCAN (Targetscan version: Mouse:7.1, Human:7.1) databases, network of regulatory relations from BioGRID: Biological General Repository for Interaction Datasets v3.4.154. October 25th, 2017, and diseases from the KEGG database (Release 84.0+/10-26, Oct 17).

### RNA isolation and qRT-PCR

Tumors (n = 14 control mice, 23 *Hltf*-deleted mice) were dissolved in 1ml of Trizol reagent and RNA was extracted. The RNA was DNAse-treated and cDNA was synthesized from 100ng of RNA using the Superscript VILO kit. Real time PCR with target-specific primers (*ATP5e*, Assay ID-Mm01239887_m1; *Uqcr10*, Assay ID-Mm01186961_m1; *Cox7c*, Assay ID-Mm01340476_m1; *Nufa4*, Assay ID-Mm04208480_g1; *Nufb6*, Assay ID-Mm01208591_g1; and *Rn18s*, Assay ID-Mm04277571_s1) was performed using TaqMan Gene expression assays. Real time PCR was conducted in triplicate for the 37 biological samples. Non-template controls contained water instead of cDNA. The expression level of the gene of interest was evaluated using the comparative Ct method (ΔΔCt method). Threshold values (Ct) for the gene of interest and the reference gene *Rn18s* were determined using QuantStudio™ 12K Flex software (Applied Biosystems Technology). Ct values for each gene of interest were normalized to Ct values for *Rn18s* in each sample and then the fold change for the gene of interest was calculated relative to the level in the control sample.

### Mitochondrial DNA copy number (qPCR)

Tumors (n = 6 control mice, 16 *Hltf*-deleted mice) were flash frozen prior to total DNA isolation with the DNeasy^®^ Blood & Tissue Kit. Concentrations of dsDNA were determined with a NanoDrop^™^ One^C^ microvolume UV-Vis spectrophotometer (Thermo Scientific). Mitochondrial (mt) copy number was achieved with the Mouse Mitochondrial DNA Copy Number Assay Kit. Real time PCR was conducted in duplicate for each biological sample. Non-template controls contained water instead of cDNA. The kit provided a positive reference control, i.e. total DNA isolated from liver of B6 mouse, SYBR^™^ green master mix, and primers for amplification of either the nuclear (n) gene beta actin (*Actb*) or the mitochondrial (mt) gene NADH dehydrogenase 4 (*Nd4*). Threshold values (Ct) for nuclear and mitochondrial genes for each sample were determined using QuantStudio 12K Flex software (Applied Biosystems Technology). The relative mtDNA content was quantified by normalizing the mitochondrial gene to the nuclear gene using the comparative Ct method (ΔΔCt method). A total of 20ng genomic DNA was used for mitochondrial and nuclear DNA markers.

## Results

### *Hltf*-deletion reduced survival

**Fig 1:**
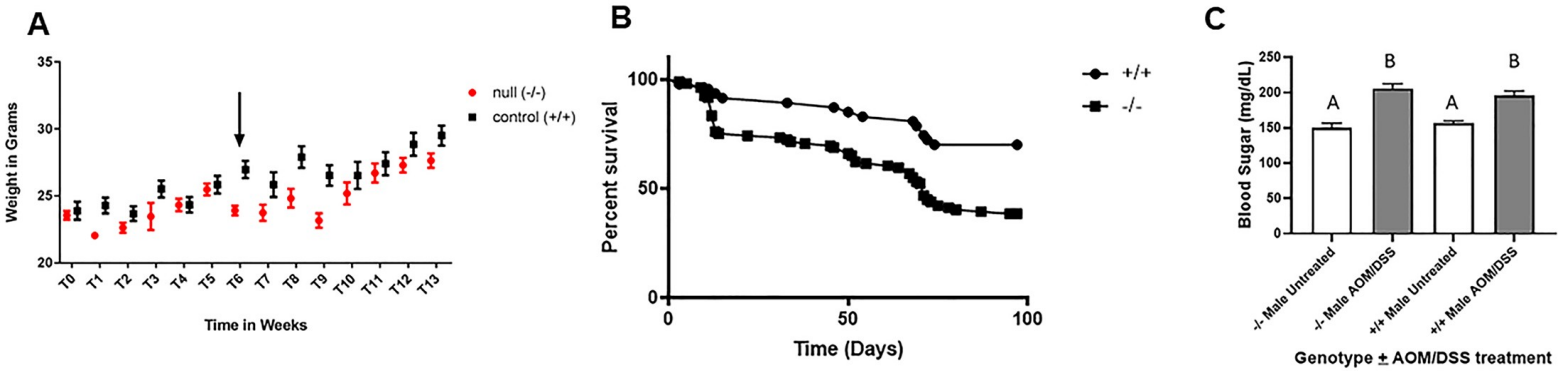
AOM/DSS-treatment effects. **A. Comparative weight loss as surrogate marker for morbidity**. *Hltf*-deleted designated null -/- (n = 23) and control +/+ (n = 17) mice were weighed on the first (T0) and last weeks (T13) and at 7-day intervals during the treatment protocol. One-way ANOVA (p <0.00001) with Tukey’s multiple comparison test was performed to determine statistical significance. *p <0.05. The first significant weight loss occurred in *Hltf*-deleted mice at treatment week 6 (arrow). **B. Kaplan-Meier survival plot**. Comparison of the cumulative survival curve of male *Hltf*-deleted (n = 109) and control (n = 47) mice indicated differential survival times for *Hltf*-deleted vs control AOM/DSS-treated mice. **C. Blood glucose levels in non-fasting adult male mice**. Blood glucose levels were normal for untreated *Hltf*-deleted vs control, and elevated to the same extent in both groups by AOM/DSS-treatment. In panels A and C, values are mean±SEM. Values with the same letter designation in panel C are not significantly different.

There was no difference in body weight between treatment groups (p>0.9999) at the time of AOM injection (Fig 1A). DSS treatment produces colitis because it has a toxic effect on the epithelium of the colon. Daily monitoring revealed no evidence of bloody diarrhea in the cage bedding of either *Hltf*-deleted or control mice. Data evaluation with a one-way ANOVA (p <0.00001) with Tukey’s multiple comparison test (*p <0.05) showed the first significant weight loss (Fig 1A) occurred in *Hltf*-deleted mice at treatment week six. Comparison of survival curves for male *Hltf*-deleted (n = 109) and control (n = 47) mice with the Logrank (Mantel-Cox) test (Chi square 12.27, p = 0.0005), and the Gehan-Breslow-Wilcoxon test (Chi square 11.88, p = 0.0006), indicated *Hltf*-deletion negatively effects the mortality of AOM/DSS-treated mice (Fig 1B) The hazard ratio (logrank) indicated the risk of dying is 2.646-fold higher when *Hltf* is deleted. The median survival time for *Hltf*-deleted mice was 71 days. The value for controls was undefined because most were alive at the end of the 97-day treatment protocol.

The *Hltf*-deleted phenotype is a perinatal lethal phenotype [24–26] in which newborn *Hltf*-deleted mice become severely hypoglycemic, and 75% die 12–24 hours after birth. Because we performed experiments with surviving *Hltf*-deleted mice, it was important to measure their blood glucose levels (Fig 1C). Statistical evaluation (ANOVA p<0.0001; Brown-Forsythe test p = 0.0143; Bartlett’s test p = 0.0010; Tukey’s Multiple comparisons test p<0.0001) of *Hltf*-deleted, untreated (n = 17) and AOM/DSS-treated (n = 33) mice, and control untreated (n = 16) and AOM/DSS-treated (n = 16) mice, showed elevated blood glucose levels in response to AOM/DSS-treatment. However, these increases did not achieve the level necessary for a diagnosis of diabetes, i.e. glucose concentration in blood ≥250 mg/dL and chronic elevation ≥300 mg/dL [29, 30].

### Tumor histology

**Fig 2:**
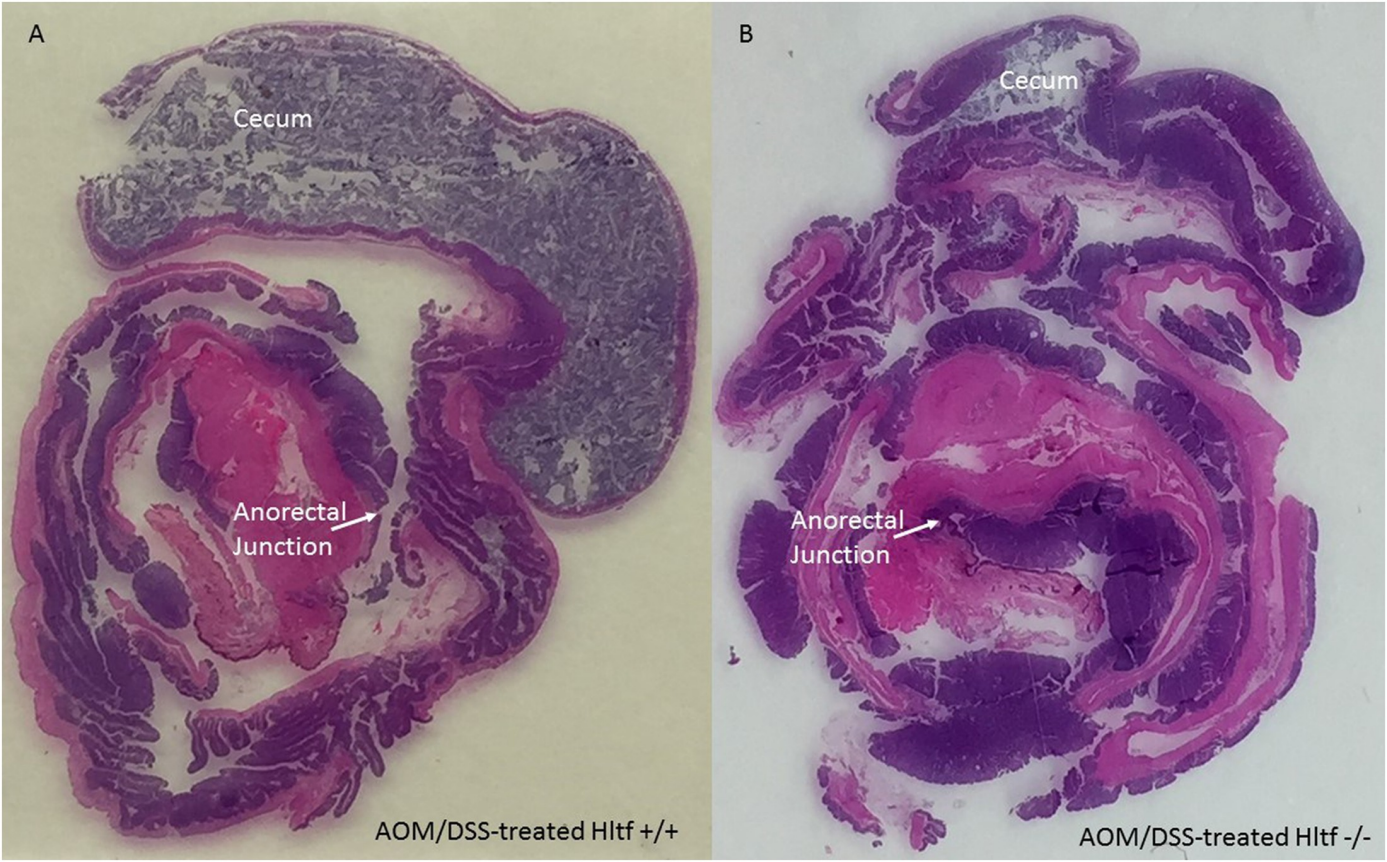
H&E stained Swiss-rolled colons. The entire large intestine for wild type control (A) and *Hltf*-deleted (B) mice is shown in each photomicrograph beginning with the cecum in the outmost region of the roll, continuing to the rugated region of the proximal colon, and terminating with the anorectal junction in the innermost region of the roll. Tumors and cellular inflammatory infiltrate in tumor stroma are evident in the distal colon. Pronounced loss of crypt architecture is evident in *Hltf*-deleted tissue.

The Swiss-roll technique aids in the complete assessment of the colon. Swiss-rolled colons from AOM/DSS-treated *Hltf*-deleted and control mice were stained with hematoxylin and eosin (H&E). Pronounced architectural distortion in *Hltf*-deleted mice is shown in Fig 2.

**Fig 3:**
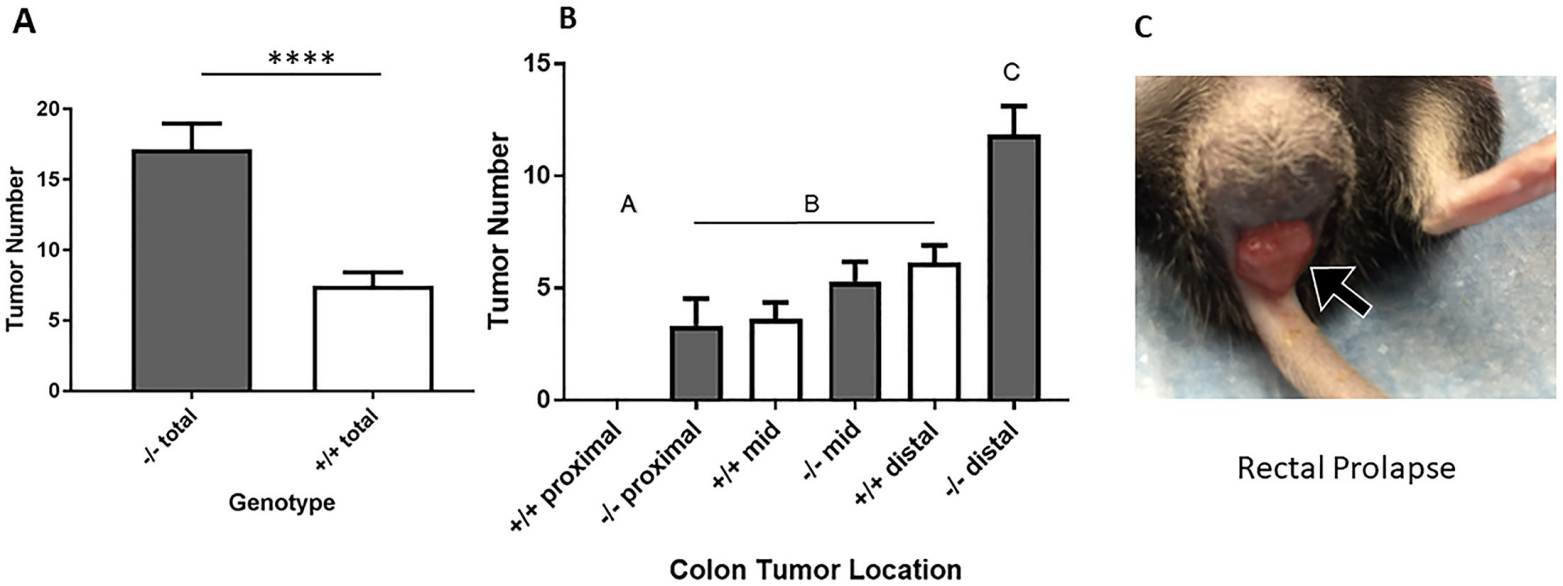
Histograms of tumor number (A) and gross distribution (B), along with rectal prolapse (C). Statistical confirmation of the dramatic effect of *Hltf*-deletion on the total number of tumors (A) and on the spatial distribution of tumors (B) is consistent with the complete protrusion of the rectum through the anal canal beyond the anus (C). Values in panels A and B are mean ± SEM. Values with the same letter designation in panel B are not significantly different.

**Fig 4:**
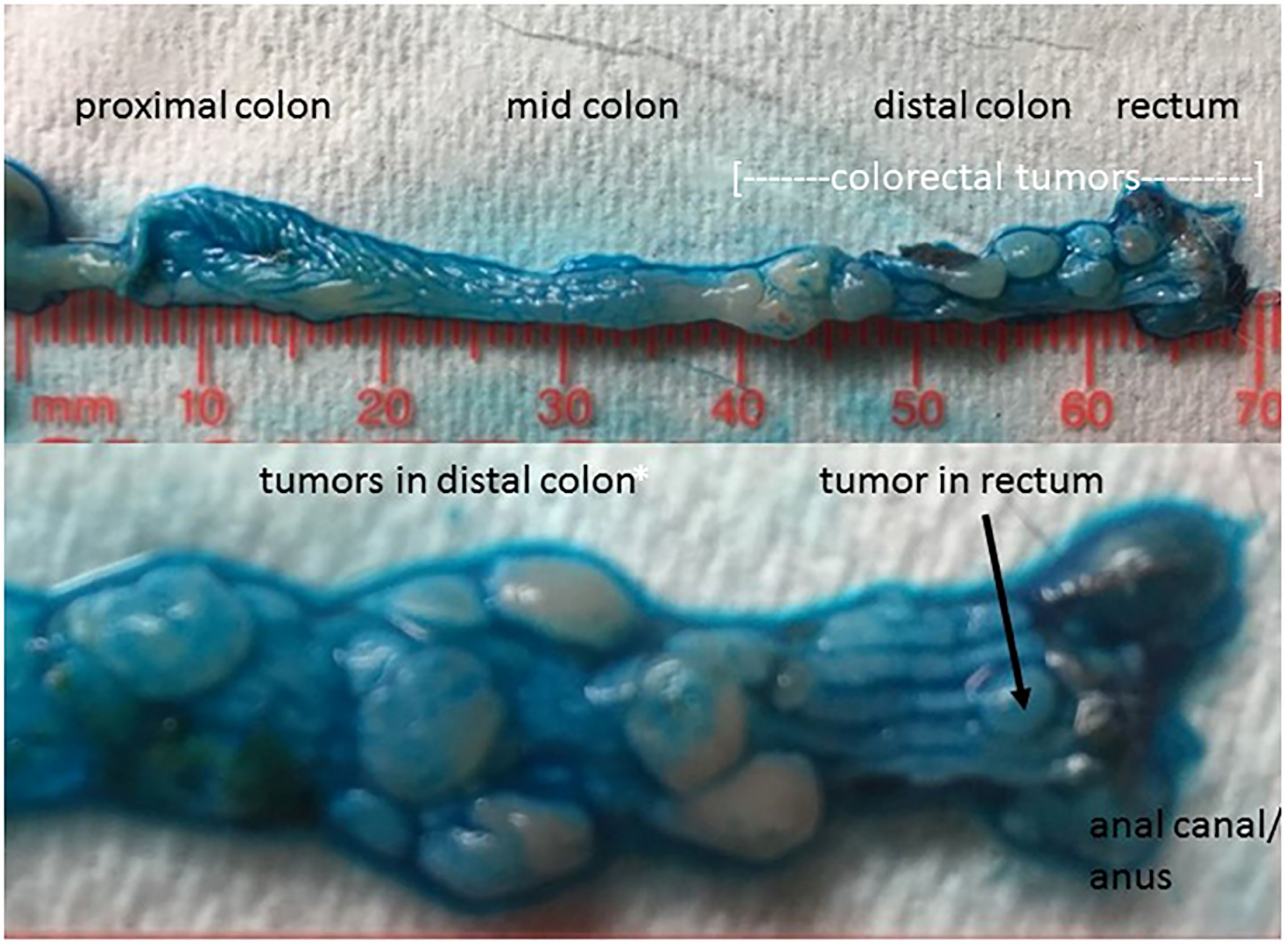
Macroscopic visualization of tumors in *Hltf*-deleted colon. Alcian blue (1%) was topically applied to longitudinally opened colons from two different *Hltf*-deleted mice to highlight the borders of the individual tumors against the normal epithelium of the colon (upper panel) and each other (lower panel). The heterogeneity of the tumor burden in the distal colon/rectum is well demarcated by the Alcian blue stain.

Decreased survival of *Hltf*-deleted mice coincided with an increased tumor burden. An unpaired t-test (****p <0.0001) was used to show the dramatic increase in the total number of colon tumors (Fig 3A) in *Hltf*-deleted mice compared with controls. The impact of *Hltf*-deletion on the regional distribution of tumors (Fig 3B) was significant (ANOVA p<0.0001; Brown-Forsythe test p = 0.0312; Tukey’s Multiple comparisons test p<0.0001). The ‘distal shift’ of tumors in the large intestine of *Hltf*-deleted mice that often resulted in rectal prolapse (Fig 3C) is evident in Fig 4.

**Fig 5:**
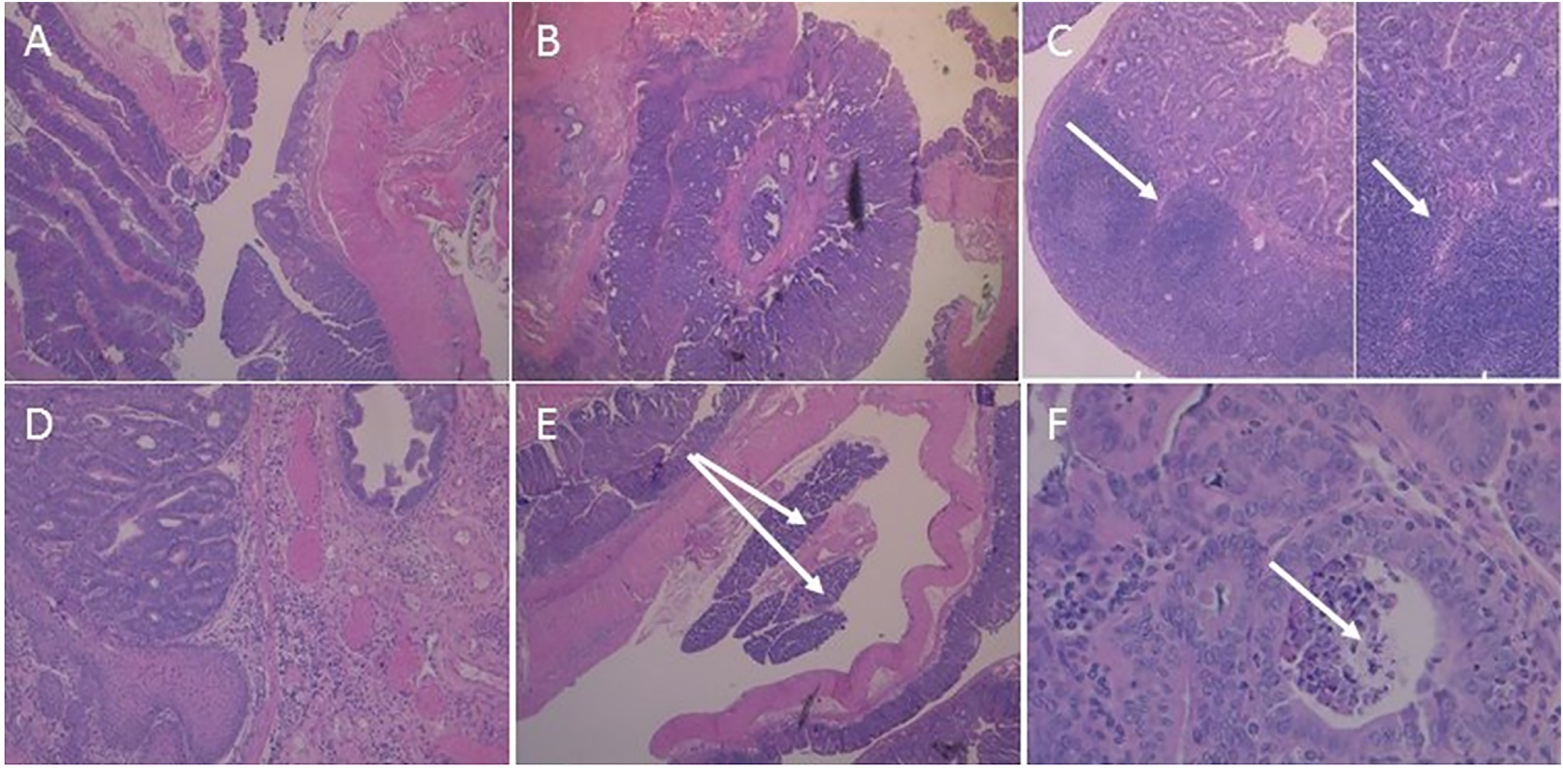
Representative histology of AOM/DSS-induced invasive adenocarcinoma. H&E stained sections from Swiss-rolled colons in wild type control (A; 25x magnification) and *Hltf*-deleted (B-F) tumors. B. A very large tumor nearly encircles the anal canal (25x magnification). C. Crohn’s disease-like reaction consisting of discrete lymphoid aggregates with germinal centers and invasion at arrow at 25x magnification and 100x magnification (inset). D. Anal canal with invasive tumor (100x magnification). E. Heterotopic (or ectopic) pancreas at arrows (25x magnification). F. Tumor necrosis (arrow) in the anal canal (100x magnification).

AOM/DSS-treatment produced low-grade invasive adenocarcinomas (pT1) in the colons of *Hltf*-deleted and control mice (Fig 5). Foci extend to the squamocolumnar junctions in both groups. Histopathological evidence for inter-tumor and intra-tumor heterogeneity (Fig 5) includes submucosal invasion and regional lymphocytic response.

### Gene expression profiling

In mice, helicase-like transcription factor is alternatively spliced [24–26]. RNA processing yields a full-length message isoform (4955-bp; exons 1–25) and a 3´-truncation mutant (3059-bp; exons 1–21 with exon 21 extended via a partial intron retention event). Cuff.diff alternative splicing analysis quantified the usage of each exon and each possible splice junction for *Hltf* in RNA-seq samples from control tumors to yield a 3.1:1.8 ratio of truncated to full-length isoform. These findings suggest dual regulation of downstream targets by *Hltf* isoforms.

**Fig 6:**
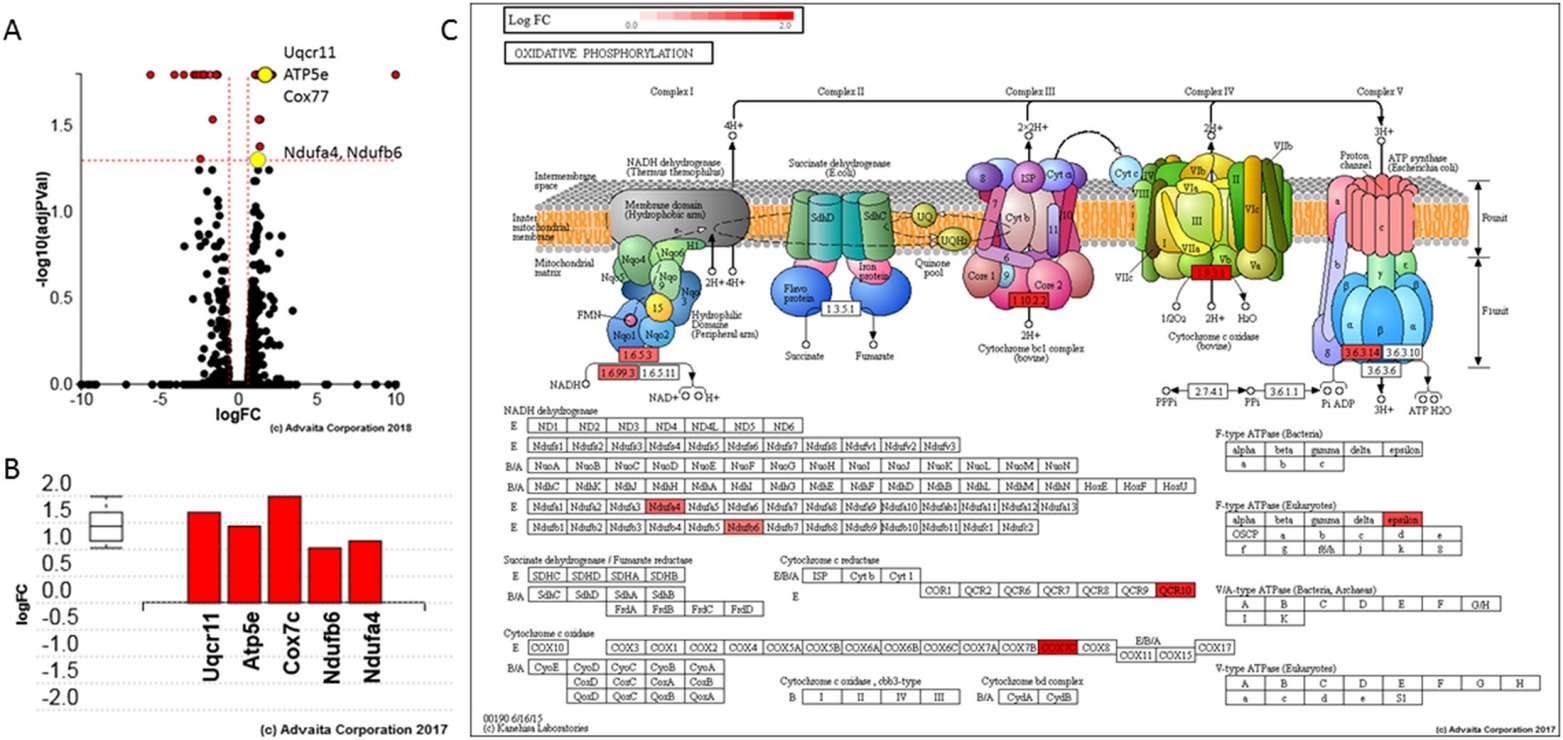
*Hltf* regulates OXPHOS. **A**. **Volcano plot**. Differential expression (DE) of *ATP5e*, *Cox7c*, *Uqcr11*, *Ndufa4* and *Ndufb6* is the measured expression of change (x-axis) and the significance of the change (y-axis). Groups of genes represented by a single yellow dot share the same change in gene expression compared to their matched controls. Significance is the negative log (base 10) of the p-value, and the most significant changes are plotted higher on the y-axis. The dotted lines represent the thresholds used to select the DE genes: LogFC = **0.6** *for expression change and* **0.05** for significance (p-value shown in terms of the negative log (base 10) value). **B. Gene perturbation bar plot**. All the genes in OXPHOS (KEGG: 00190) are ranked based on their absolute perturbation values. The box and whisker plot on the left summarizes the distribution of all the differentially increased genes annotated to this pathway. The box represents the 1st quartile, the median and the 3rd quartile. **C. OXPHOS (KEGG: 00190) pathway diagram is overlaid** with the computed perturbation of each gene. Perturbation accounts for a gene’s measured fold change and for the accumulated perturbation propagated from any upstream genes (accumulation). The highest positive perturbation is in dark red. Genes are highlighted in all locations in the diagram.

With iPathwayGuide, **51** differentially expressed genes (S1 Table) were identified from a total of **10,967** genes (S2 Table) with measured expression. iPathwayGuide obtained these data using a threshold of **0.05** for statistical significance (p-value) and a logfold change of expression with an absolute value of at least **0.6**. Oxidative phosphorylation (KEGG: 00190) was identified as the top pathway (Fig 6) with the following associated statistics: p = 1.157^e-4^, p-value (FDR) = 0.006, and p-value (Bonferroni) = 0.006.

**Fig 7:**
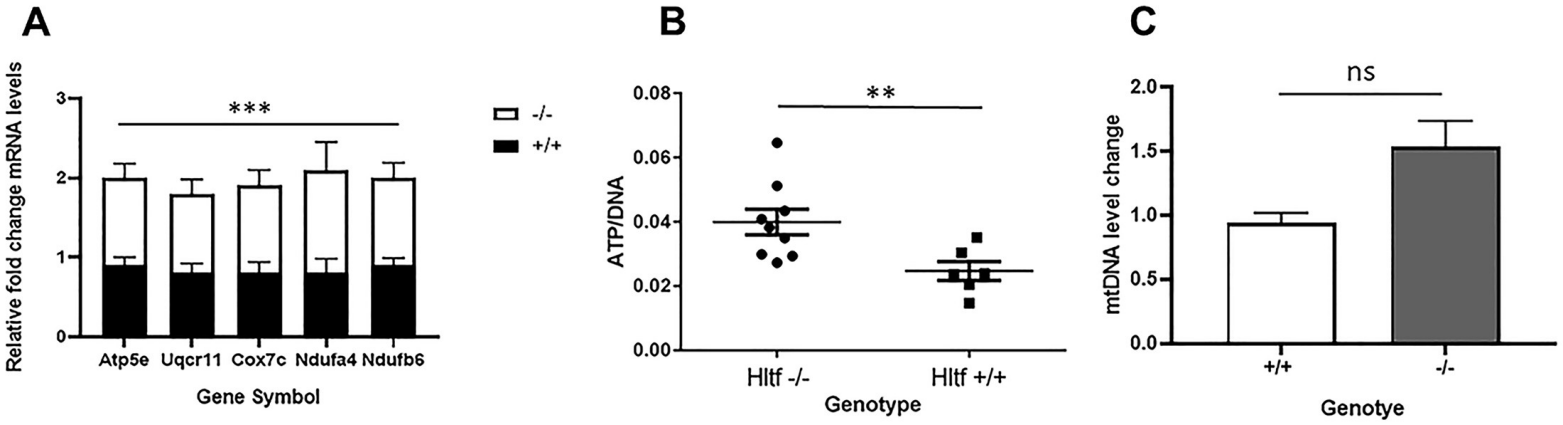
Gene expression and mitochondrial function. A. qRT-PCR confirmed upregulation of the five genes annotated to oxidative phosphorylation in tumors from *Hltf*-deleted (n = 23) compared to control (n = 14) mice. B. Total cellular ATP was normalized to DNA from *Hltf*-deleted (n = 8) and control (n = 8) samples that were assayed in duplicate. C. Mouse mitochondrial DNA copy number was determined by the comparison of mitochondrial (mt) and nuclear (n) DNA measured by qPCR. Of the 16 tumor samples from *Hltf*-deleted mice, the one sample that failed to amplify was excluded from the calculations. Values in each panel are expressed as mean±SEM, ***p≤0.001, **p≤0.01, non-significant (ns) = P>0.05.

Upregulation of the five genes annotated to oxidative phosphorylation was confirmed qRT-PCR (Fig 7A). Data imported into GraphPad Prism for statistical analysis (unpaired t-test, ***p = 0.0009) are represent as the relative fold increase in gene expression (mean ± SEM) in tumors from *Hltf*-deleted mice (n = 23) compared to controls (n = 14). Increased transcription of five oxidative phosphorylation (OXPHOS) genes–*ATP5e*, *Cox7c*, *Uqcr11*, *Ndufa4*, and *Ndufb6* –encoded by the nuclear genome—caused an increase in endogenous levels of ATP in *Hltf*-deleted tumors (Fig 7B). Standards, *Hltf*-deleted (n = 8) and control (n = 8) samples were assayed with Abcam’s luminescent ATP detective assay kit (ab113849). We measured the DNA content in duplicate with a PicoGreen dsDNA assay. R^2^ = 0.999 correlation coefficient for the standard curve. Duplicate samples were averaged, corrected for background, and normalized to DNA. Multiple ratio data were ranked and the ranks between the groups were analyzed by Mann Whitney U (nonparametric) test (**p = 0.0062). Increased transcription of the five oxidative phosphorylation (OXPHOS) genes concomitant with increased endogenous levels of ATP in *Hltf*-deleted tumors occurred in the absence of any *Hltf*-induced change in the tumor mtDNA:nDNA ratio as determined by qPCR (Fig 7C). Mouse mitochondrial DNA copy number was determined by the comparison of mitochondrial (mt) and nuclear (n) DNA measured by qPCR. The ΔΔCt was calculated for each *Hltf*-deleted tumor sample by using the mean of the +/+ samples (n = 6) as control. Data were imported into GraphPad Prism for statistical analysis (unpaired t-test, p = 0.0853).

In agreement with these findings, *Hltf*-deletion increased transcription of the gene for *Cdk5rap1* (p = 0.016) that catalyzes 2-methylthio (ms^2^) modifications of four mt-tRNAs that optimize mitochondrial translation and OXPHOS activity [31]. By comparison, *Hltf*-deletion had no effect on transcription of the master regulators of mitochondrial biogenesis, i.e. the *PGC-1α—NRF -Tfam* pathway [32]. Furthermore, there was no *Hltf*-deletion effect on expression of mitochondrial polymerase gamma (*Polg*) required for replication of mtDNA [33], and no change in the expression of *VDAC/porin*, a marker for mitochondrial abundance [34].

## Discussion

Nearly all mammals have two *HLTF* splice variants, a full-length message isoform and a 3´-truncated isoform. During development, the full-length *Hltf* mRNA encodes a protein that regulates the G/2M transition of the cell cycle in mouse brain [24] and heart [25]. This function is consistent with its role in DNA damage repair [35]. Expression of the *Hltf* C-terminal truncated isoform that lacks the DNA repair domain is exclusive to placenta where genome amplification in trophoblasts requires endocycles and suppression of the DNA damage response [26]. The expression of both isoforms at a ratio that favors the truncated isoform is a newly identified characteristic in this AOM/DSS-CRC model.

The mitochondrial genome consists of 37 genes encoding 13 subunits of the OXPHOS pathway, 22 tRNAs and 2 rRNAs. However, 97 genes are required for OXPHOS. Nuclear DNA encodes the remaining 84 genes [36]. In this study, RNA-seq validated by qRT-PCR, showed *HLTF*-deletion transcriptionally upregulated five nuclear genes that participate in OXPHOS concordant with increased cellular ATP availability. These genes encode five components of the OXPHOS electron transport chain (OXPHOS-ETC) located in the inner mitochondrial membrane [37]. Four are members of the supercomplex known as the respirasome: complex I (*Ndufa4*, *Ndufb6*), complex III (*Uqcr11*), and complex IV (*Cox7c*). The fifth member of complex V (*Atp5e*) uses the energy from the respirasome to synthesize ATP. In contrast, there was no *Hltf*-deletion effect on transcription of the genes primarily responsible for the generation of new mitochondria [32] such as nuclear-respiratory factor 1 and 2 (*NRF-1*, *NRF-2*), mitochondrial transcription factor A (*TFAM*), and peroxisome proliferator-activated receptor-gamma coactivator 1-α (*PGC-1α*). These findings were in agreement with the absence of an *Hltf*-deletion effect on the mtDNA/nDNA ratio. Additionally, *Hltf*-deletion neither effects the expression of the nuclear-encoded mitochondrial polymerase gamma (*Polg*) nor the expression of mitochondrial enzymes such as citrate synthase (*CS*). Collectively, our data show silencing *Hltf* alters mitochondrial function not abundance.

These findings are consistent with recent literature indicating CRC, unlike other cancers and contrary to the Warburg hypothesis, utilizes OXPHOS-ETC as it major energy source [38, 39]. The Warburg hypothesis states that impaired mitochondrial function causes a metabolic shift from OXPHOS to inefficient non-oxidative breakdown of glucose in cancer cells otherwise known as aerobic glycolysis. However, most tumor cells retain functional mitochondria [40]. Sun et al [39] recently demonstrated increased mtDNA copy number promoted OXPHOS and CRC progression. In our study, the benefit to silencing the tumor suppressor gene *Hltf* in CRC is increased OXPHOS and mitochondrial energy production. At the most fundamental level, silencing *Hltf* promotes tumor survival in an inflammatory model of CRC. To overcome the problem of tumor heterogeneity [41], the next step in understanding metabolic reprogramming due to *Hltf*-deletion requires studies that can differentiate tumor epithelial cells from the tumor microenvironment.

## Conclusions

Examining the AOM/DSS-induced colon carcinogenesis process in global *Hltf*-deleted mice provided the first opportunity to study the effects of *Hltf*-deletion on tumor histology and the tumor transcriptome without the complications of crossbreeding into a tumorigenic strain. The *Hltf*-deletion increased the tumor burden and the number of tumors in the distal colon/rectum, and significantly shortened overall survival. RNA-seq analysis and confirmatory qRT-PCR revealed *Hltf*-deleted tumors had increased transcripts of nuclear genes for OXPHOS concomitant with increased endogenous levels of ATP. *Hltf*-deletion had no effect on the mtDNA/nDNA ratio indicating *Hltf*-deletion in this inflammation-associated CRC model supports the process of OXPHOS, an energy-converting mechanism essential for life [37].

## Supporting information

S1 Checklist NC3Rs ARRIVE Guidelines Checklist (fillable). (PDF)

S2 Checklist Plos-one-humane-endpoints-checklist-1. (PDF)

S1 Table All differentially regulated genes. Cuff.diff, a component of Cufflinks, uses RPKM values to calculate changes in gene expression between *Hltf*-deleted and control tumors. When these data were analyzed by iPathwayGuide a total of 34 upregulated genes and 13 downregulated genes were identified in *Hltf*-deleted tumors. The logfold change (logfc) and adjusted p values (adjpv) are provided.
(PDF)

S2 Table All genes with measured expression. All genes with measured expression in the tumor transcriptome are listed in this table.
(PDF)
