# Supplementary material for: Helicase-like transcription factor (Hltf) gene-deletion promotes oxidative phosphorylation (OXPHOS) in colorectal tumors of AOM/DSS-treated mice
Source: PLoS One. 2019 Aug 28;14(8):e0221751. doi: 10.1371/journal.pone.0221751 (PMC6713344; doi:10.1371/journal.pone.0221751)
Supplement: S1 Table — Cuff.diff, a component of Cufflinks, uses RPKM values to calculate changes in gene expression between Hltf-deleted and control tumors. When these data were analyzed by iPathwayGuide a total of 34 upregulated genes and 13 downregulated genes were identified in Hltf-deleted tumors. The logfold change (logfc) and adjusted p values (adjpv) are provided. (PDF) [file pone.0221751.s003.pdf]

S1 Table A list of all DE genes

| symbol | entrez | logfc | adjpv |
|---|---|---|---|
| Bglap-rs1 | 12095 | 1.56301 | 0.015933 |
| Cox7c | 12867 | 1.99311 | 0.015933 |
| Ctsh | 13036 | 1.14443 | 0.015933 |
| Des | 13346 | -2.84596 | 0.015933 |
| Ifit1 | 15957 | 1.54251 | 0.015933 |
| Fabp5 | 16592 | -1.82288 | 0.015933 |
| Krt31 | 16660 | 10 | 0.015933 |
| Krt84 | 16680 | 2.15183 | 0.015933 |
| Krt6a | 16687 | -3.48854 | 0.015933 |
| Krt6b | 16688 | -2.69113 | 0.015933 |
| Ltf | 17002 | -2.13982 | 0.015933 |
| Mmp13 | 17386 | -1.33781 | 0.015933 |
| Mt2 | 17750 | 1.31927 | 0.015933 |
| Pmp22 | 18858 | 1.39383 | 0.015933 |
| Pnliprp2 | 18947 | 2.01637 | 0.015933 |
| Rmrp | 19782 | 1.73389 | 0.015933 |
| Cxcl2 | 20310 | -1.39794 | 0.015933 |
| Slfn4 | 20558 | 1.06062 | 0.015933 |
| Hltf | 20585 | -2.33892 | 0.015933 |
| Phgr1 | 53906 | 1.55776 | 0.015933 |
| Pdcd10 | 56426 | 1.19809 | 0.015933 |
| Ppbp | 57349 | -2.49867 | 0.015933 |
| 1810030J1 | 66289 | -4.08685 | 0.015933 |
| Mgst3 | 66447 | 1.55322 | 0.015933 |
| Uqcr11 | 66594 | 1.69535 | 0.015933 |
| Serpinb11 | 66957 | 1.48056 | 0.015933 |
| Cdk5rap1 | 66971 | 1.62609 | 0.015933 |
| 2010109I0 | 67038 | 1.69133 | 0.015933 |
| Atp5e | 67126 | 1.43442 | 0.015933 |
| Krt33a | 71888 | 10 | 0.015933 |
| Clca6 | 99663 | 2.0791 | 0.015933 |
| Calcb | 116903 | -5.61513 | 0.015933 |
| Cwh43 | 231293 | 1.39567 | 0.015933 |
| Dhrs9 | 241452 | 1.2084 | 0.015933 |
| Parp12 | 243771 | 1.06013 | 0.015933 |
| Hist1h2an | 319170 | -1.45094 | 0.015933 |
| Hist1h2ab | 319172 | -2.22424 | 0.015933 |
| Hist1h2bk | 319184 | 1.84487 | 0.015933 |
| AA467197 | 433470 | 1.30095 | 0.015933 |
| 1810006K2 | 69038 | -1.65071 | 0.028968 |
| Ifi27l2b | 217845 | 1.30839 | 0.028968 |
| Hist1h3b | 319150 | 1.38803 | 0.028968 |
| Muc6 | 353328 | 1.27761 | 0.028968 |
| Ddx60 | 234311 | 1.34752 | 0.041563 |
| Isg15 | 1E+08 | 1.3583 | 0.041563 |

| | | | |
|---|---|---|---|
| Ndufa4 | 17992 | 1.15834 | 0.049023 |
| Smpdl3a | 57319 | 1.24813 | 0.049023 |
| Rpp21 | 67676 | 1.27543 | 0.049023 |
| Krt5 | 110308 | -2.43485 | 0.049023 |
| Ndufb6 | 230075 | 1.02754 | 0.049023 |
| Ccdc68 | 381175 | 1.00076 | 0.049023 |
