# Supplementary material for: Helicase-like transcription factor (Hltf) gene-deletion promotes oxidative phosphorylation (OXPHOS) in colorectal tumors of AOM/DSS-treated mice
Source: PLoS One. 2019 Aug 28;14(8):e0221751. doi: 10.1371/journal.pone.0221751 (PMC6713344; doi:10.1371/journal.pone.0221751)
Supplement: S2 Table — All genes with measured expression in the tumor transcriptome are listed in this table. (PDF) [file pone.0221751.s004.pdf]

S2 Table List of all genes with measured expression

symbol	entrez	logfc	adjpv
Bglap-rs1	12095	1.56301	0.015933
Cox7c	12867	1.99311	0.015933
Ctsh	13036	1.14443	0.015933
Des	13346	-2.84596	0.015933
Ifit1	15957	1.54251	0.015933
Fabp5	16592	-1.82288	0.015933
Krt31	16660	10	0.015933
Krt84	16680	2.15183	0.015933
Krt6a	16687	-3.48854	0.015933
Krt6b	16688	-2.69113	0.015933
Ltf	17002	-2.13982	0.015933
Mmp13	17386	-1.33781	0.015933
Mt2	17750	1.31927	0.015933
Pmp22	18858	1.39383	0.015933
Pnliprp2	18947	2.01637	0.015933
Rmrp	19782	1.73389	0.015933
Cxcl2	20310	-1.39794	0.015933
Slfn4	20558	1.06062	0.015933
Hltf	20585	-2.33892	0.015933
Phgr1	53906	1.55776	0.015933
Pdcd10	56426	1.19809	0.015933
Ppbp	57349	-2.49867	0.015933
1810030J14Rik	66289	-4.08685	0.015933
Mgst3	66447	1.55322	0.015933
Uqcr11	66594	1.69535	0.015933
Serpinb11	66957	1.48056	0.015933
Cdk5rap1	66971	1.62609	0.015933
2010109I03Rik	67038	1.69133	0.015933
Atp5e	67126	1.43442	0.015933
Krt33a	71888	10	0.015933
Clca6	99663	2.0791	0.015933
Calcb	116903	-5.61513	0.015933
Cwh43	231293	1.39567	0.015933
Dhrs9	241452	1.2084	0.015933
Parp12	243771	1.06013	0.015933
Hist1h2an	319170	-1.45094	0.015933
Hist1h2ab	319172	-2.22424	0.015933
Hist1h2bk	319184	1.84487	0.015933
AA467197	433470	1.30095	0.015933
1810006K21Rik	69038	-1.65071	0.028968
Ifi27l2b	217845	1.30839	0.028968
Hist1h3b	319150	1.38803	0.028968
Muc6	353328	1.27761	0.028968
Ddx60	234311	1.34752	0.041563
Isg15	1E+08	1.3583	0.041563

Ndufa4	17992	1.15834	0.049023
Smpdl3a	57319	1.24813	0.049023
Rpp21	67676	1.27543	0.049023
Krt5	110308	-2.43485	0.049023
Ndufb6	230075	1.02754	0.049023
Ccdc68	381175	1.00076	0.049023
Igj	16069	-1.682	0.056902
Msln	56047	1.94054	0.056902
Oasl1	231655	1.23907	0.056902
Tmprss11bnl	319875	-2.51532	0.056902
Rnf19b	75234	0.988946	0.065928
Apcdd1	494504	1.10322	0.065928
Myh11	17880	-1.9975	0.075612
Dsg3	13512	-1.32004	0.084973
Mcpt1	17224	-1.29578	0.092511
Atp12a	192113	1.09183	0.092511
Ret	19713	1.19616	0.099578
Mgat4c	67569	0.963529	0.099578
Alb	11657	1.35986	0.104631
Krt20	66809	1.35866	0.104631
Nox1	237038	-1.21355	0.104631
Mmp3	17392	-2.56785	0.112465
Tmem126a	66271	1.17272	0.118356
Psca	72373	-1.41418	0.118356
Crip1	12925	1.28974	0.123919
Rps21	66481	1.18505	0.123919
Acsbg1	94180	1.19203	0.129182
Nanos1	332397	1.12063	0.129182
Cyp2f2	13107	-2.53449	0.134168
Tmprss11d	231382	-1.90637	0.134168
Slc26a3	13487	-2.49527	0.137141
Madcam1	17123	2.75145	0.137141
Dapl1	76747	1.9497	0.137141
Nudt21	68219	1.09909	0.13821
Atp1a2	98660	1.10564	0.13821
Pqlc2	212555	0.994529	0.13821
Slc9a2	226999	1.13942	0.13821
Calml3	70405	-2.12014	0.148209
Fam57a	116972	-1.14156	0.148209
Suox	211389	1.14609	0.148209
H2-DMb1	14999	-1.51503	0.157535
Hba-a1	15122	-3.45459	0.157535
Tmem181b-ps	547127	1.11315	0.157535
Actg2	11468	-1.32995	0.157612
Ralgapa1	56784	-2.43502	0.157612
Rpf2	67239	0.926925	0.157612
Ndufs5	595136	1.00539	0.157612

Slc25a20	57279	0.958102	0.162715
Cd55	13136	0.933061	0.167711
Erdr1	170942	2.60867	0.172602
H2afx	15270	-0.91223	0.182086
Vtcn1	242122	-0.98378	0.182086
Krt17	16667	-1.42649	0.186684
Tm2d1	94043	1.37692	0.197563
Capg	12332	1.22897	0.20222
Reg3b	18489	1.16489	0.20222
Vaultrc5	378472	1.4163	0.20222
Ptprh	545902	0.927626	0.20222
Kcnu1	16532	1.80448	0.212433
Car5b	56078	1.39173	0.216442
1190003J15Rik	76974	-0.8791	0.218334
Nlrp6	101613	-1.05906	0.218334
Pck1	18534	-2.01703	0.228025
Clec2h	94071	0.88758	0.249126
Isl1	16392	1.04056	0.250368
Mab21l3	242125	-1.07923	0.250368
Mrpl54	66047	0.90885	0.253792
Pkhd1	241035	0.895836	0.257156
Plac8	231507	0.895575	0.277087
Ier3	15937	-0.85256	0.277797
Hist1h2bm	319186	1.07617	0.277797
Tmprss11g	320454	-1.32901	0.286245
Ifit3	15959	1.15761	0.286785
1110032A04Rik	66183	1.06938	0.286785
5730437N04Rik	70544	0.819626	0.289682
Cldn2	12738	-0.8719	0.292531
Ndufb8	67264	0.909428	0.295334
Slpi	20568	0.902258	0.300806
4732456N10Rik	239673	1.06874	0.300806
Snhg5	72655	-0.80694	0.301087
Slc5a8	216225	-0.99856	0.301087
Prnp	19122	0.850357	0.301623
Rbm8a	60365	-1.2414	0.301623
Bloc1s2	73689	1.00217	0.301623
Crnn	381457	1.47155	0.301623
Epb4.1l4b	54357	0.824182	0.301879
Cdhr2	268663	0.873835	0.301879
Tmem181c-ps	1E+08	1.08157	0.304382
Stmn2	20257	-1.97782	0.304592
Wdfy1	69368	1.04461	0.304592
Enoph1	67870	2.01809	0.30702
Penk	18619	-2.90622	0.31865
Ndufb10	68342	0.803305	0.31865
Epb4.1l5	226352	0.861741	0.31865

Gprc5a	232431	0.828239	0.32091
Klk1	16612	-1.22248	0.323138
Rdh16	19683	1.02355	0.325153
Rps27a	78294	-0.99327	0.325153
2210407C18Rik	78354	-0.96549	0.325153
Efhb	211482	2.22267	0.325153
Gulo	268756	1.28309	0.325153
Pitx1	18740	-1.90343	0.327262
Mt4	17752	0.938302	0.337897
Coro2a	107684	0.830265	0.344142
Llph	66225	0.862026	0.346083
Gcnt4	218476	0.998269	0.347999
Cplx2	12890	0.84794	0.349094
Abcg2	26357	1.08936	0.349094
Cxcl14	57266	1.02001	0.349094
AI467606	101602	1.53034	0.349094
I830012O16Rik	667370	1.0736	0.349094
Car12	76459	-0.9792	0.354952
Gm6289	622139	2.23535	0.360736
Pnliprp1	18946	1.1561	0.365857
Tmem38b	52076	0.934043	0.365857
Beta-s	1.01E+08	-1.65969	0.365857
Cdc34	216150	0.782845	0.369168
Sap130	269003	-0.78015	0.369168
Cyp2b10	13088	1.28586	0.372398
Prr9	109314	3.44656	0.372398
Hoxd12	15432	-1.0751	0.377801
Pla2g4f	271844	1.13241	0.383139
Ssr4	20832	1.05352	0.388414
Irf7	54123	1.97268	0.389878
Ndufab1	70316	0.886865	0.391325
Ccnb2	12442	0.742703	0.40522
Nt5e	23959	0.954715	0.40522
Dkk3	50781	0.812282	0.421204
Igfbp3	16009	-1.22249	0.422439
Hsd11b2	15484	-1.07299	0.424867
Pam	18484	0.755931	0.424867
Sat1	20229	0.825244	0.424867
Srd5a1	78925	0.824268	0.424867
Gpr137b	83924	0.847727	0.424867
Fam3b	52793	0.80294	0.430703
Thoc7	66231	0.853085	0.430703
Bax	12028	-0.7336	0.438572
Col17a1	12821	-1.20416	0.438572
Hao2	56185	-1.74013	0.438572
Higd1a	56295	0.748285	0.438572
Ggh	14590	0.796359	0.442756

Oat	18242	0.90125	0.442756
Rrp1b	72462	-0.76666	0.442756
Krba1	77827	0.878594	0.442756
Scnn1a	20276	0.751778	0.443774
Nop2	110109	-0.7325	0.448102
Farsa	66590	-0.73121	0.454281
Hist2h3b	319154	1.01899	0.454281
Hist1h2bn	319187	0.812616	0.454281
Cast	12380	0.749396	0.458466
Adh1	11522	-0.91306	0.473972
Jagn1	67767	-0.7609	0.473972
Otop2	237987	-1.37706	0.473972
Gm1821	218963	0.795826	0.474789
Psmb4	19172	0.774818	0.485109
Mtap1b	17755	-0.91097	0.485709
Slc46a1	52466	0.809929	0.485709
Fer1l4	74562	0.81987	0.485709
Cyp2d34	223706	0.8577	0.485709
Esyt3	272636	0.97469	0.485709
Hist1h2br	665622	1.74732	0.48952
Clca1	23844	1.07381	0.493295
Fgfrl1	116701	1.36367	0.493984
Il23a	83430	-0.82294	0.500736
Parm1	231440	0.769656	0.504403
Ascl4	67341	1.13108	0.514049
Vsig1	78789	0.784181	0.517619
Fzd1	14362	0.78643	0.517988
Krt16	16666	-1.7138	0.517988
Fxyd3	17178	0.774562	0.517988
Nbl1	17965	1.15865	0.517988
Slc34a2	20531	-1.02309	0.517988
Slc16a10	72472	0.800529	0.517988
Gpcpd1	74182	0.863926	0.521427
Cd63	12512	0.727285	0.530603
Cd38	12494	0.776571	0.552703
Tgm3	21818	-0.82484	0.552703
Rpl36	54217	0.721106	0.552703
AI747448	99709	1.97812	0.552703
Slc25a28	246696	0.861438	0.552703
Trdmt1	13434	0.943039	0.557638
Ppid	67738	-0.69993	0.557638
Pllp	67801	0.695038	0.557638
Mrpl34	94065	-0.73493	0.557638
Mboat1	218121	0.699782	0.557638
Oas1a	246730	0.774405	0.557638
Spink3	20730	0.946926	0.56345
Usmg5	66477	0.759075	0.563765

Xpa	22590	1.13185	0.574926
Vps29	56433	0.68855	0.57519
Thsd4	207596	0.831938	0.588897
Hist3h2ba	78303	1.32073	0.589101
Ccl4	20303	-0.99587	0.589303
Fbl	14113	-1.02483	0.589701
Wls	68151	2.59905	0.589701
Cxcl1	14825	-0.69569	0.590093
Nme2	18103	2.26267	0.590093
Defa17	23855	1.26196	0.590478
Oas3	246727	0.901361	0.590478
Ifrd1	15982	0.743534	0.59123
Spink4	20731	1.80632	0.59123
Zscan21	22697	1.26173	0.59123
1810063B05Rik	67892	0.820165	0.59123
Cyp2j6	13110	0.690769	0.591779
Tpm2	22004	-1.18953	0.591779
Snhg3	399101	0.815822	0.591779
Tspan15	70423	0.703488	0.602173
Isx	71597	0.817521	0.602173
Iigp1	60440	-1.09372	0.602311
Hist4h4	320332	0.782297	0.602448
Lhfp	108927	1.1054	0.607543
F2rl1	14063	0.679182	0.620076
Ngp	18054	-2.20246	0.620076
Arf2	11841	0.728161	0.629947
Lrmp	16970	0.852526	0.629975
Nudc	18221	-0.66742	0.630003
Cops3	26572	0.699553	0.6373
Tmem37	170706	0.886094	0.6373
Alox15	11687	1.0729	0.658863
Hint1	15254	0.723201	0.658863
Ifi204	15951	-1.46189	0.665943
Glrx3	30926	1.45621	0.677726
Sema6d	214968	1.34903	0.694159
Unc5b	107449	0.750024	0.700795
Ltbp1	268977	0.718234	0.700795
Fpr1	14293	-2.34078	0.702426
Naip2	17948	0.656493	0.702426
Ptprj	19271	0.939319	0.702426
Dsg4	16769	1.28846	0.708366
Tppp3	67971	0.964641	0.708366
C2cd4b	75697	-1.5996	0.708366
1700019E19Rik	76411	-0.7468	0.708366
Nat2	17961	1.27526	0.714411
Igsf5	72058	0.913378	0.714411
Ism1	319909	0.938469	0.714411

Smad7	17131	0.655323	0.720917
Dynlt1b	21648	1.27137	0.722573
Ndnl2	66647	-0.80309	0.722573
Ifi30	65972	0.675618	0.726433
Snhg11	319317	0.971444	0.726433
Dgka	13139	0.667586	0.732678
Fosb	14282	0.996453	0.732678
Orc1	18392	0.89764	0.732678
Pvrl1	58235	-1.04925	0.732678
Mrpl13	68537	0.812205	0.732678
Ceacam20	71601	0.677994	0.732678
Sft2d1	106489	0.770968	0.732678
Sectm1a	209588	1.03991	0.732678
Cxcl9	17329	-1.22824	0.738493
Crip2	68337	-0.69966	0.738493
Cib1	23991	0.657499	0.739609
Ms4a8a	64381	0.77395	0.739609
Ppef1	237178	-1.59741	0.739609
Bola1	69168	0.765352	0.745641
Scin	20259	-1.74409	0.751254
St3gal1	20442	-0.73008	0.751254
Iqgap2	544963	0.723146	0.759291
Mfsd4	213006	0.806025	0.763083
2610019E17Rik	75614	1.54948	0.764343
Hist1h2ah	319168	-1.34856	0.764343
Ly6g6e	70274	0.835326	0.765588
Kctd9	105440	1.10694	0.765588
Pmm1	29858	0.798578	0.767235
Sprr2h	20762	1.54063	0.767611
Gipc2	54120	0.659319	0.767611
Tmem9b	56786	0.681616	0.767611
Pdcd2l	68079	0.759789	0.767611
Dlx3	13393	1.14007	0.790575
Clca2	229933	0.674729	0.790575
Paqr5	74090	0.706666	0.793129
Gpr137b-ps	664862	0.733255	0.793129
Serpinb1a	66222	0.838841	0.801573
Ecscr	68545	-0.62575	0.801573
Mgat4b	103534	0.63661	0.801573
Saa1	20208	0.988335	0.803037
Tcf7	21414	0.674128	0.824397
2510049J12Rik	70291	0.685883	0.824397
Colec12	140792	0.997774	0.824397
Duox2	214593	0.791851	0.824397
Cyyr1	224405	-1.16594	0.827713
Pafah1b3	18476	0.764611	0.827912
S100a1	20193	0.636219	0.827912

Gpnmb	93695	1.0133	0.827912
Spns2	216892	0.662039	0.831177
Gjb3	14620	0.704216	0.832814
Nnt	18115	0.702376	0.832814
Sprr1a	20753	0.696417	0.832814
Rnf128	66889	0.686795	0.832814
Endod1	71946	0.658816	0.832814
2010109K11Rik	72123	0.677631	0.832814
Lactb2	212442	0.693519	0.832814
Il11	16156	-0.98667	0.84354
Unc119	22248	0.824206	0.84354
Prss23	76453	0.765607	0.84354
Mon1b	270096	-0.86169	0.84354
Ppp1r1b	19049	-0.63956	0.846039
Gm2382	1E+08	0.876629	0.846039
Pvrl3	58998	0.671484	0.846672
2410006H16Rik	69221	0.702798	0.846672
Enpp3	209558	-1.42036	0.860721
Cacybp	12301	-0.63252	0.864651
Ckb	12709	-0.8154	0.864651
Csn1s1	12990	-2.18573	0.864651
Mxd1	17119	0.68544	0.864651
Mcpt2	17225	-0.91919	0.864651
Ccl2	20296	-1.01145	0.864651
Rer1	67830	0.62391	0.864651
Gpr110	77596	0.710271	0.864651
Thbs2	21826	0.653592	0.874062
Ddah1	69219	0.729121	0.874062
Stx11	74732	1.64055	0.882279
Dcn	13179	0.970493	0.88377
Ly6d	17068	0.720869	0.88377
Abhd12	76192	0.653957	0.88377
Apobec1	11810	0.849369	0.884558
Ubr3	68795	0.96088	0.884558
Myl9	98932	-0.87037	0.884558
Lhfpl2	218454	0.835311	0.884558
Ndufs4	17993	0.605446	0.891874
Mrpl20	66448	0.664078	0.891874
Cpa3	12873	-1.35526	0.897387
Rnf186	66825	-0.6247	0.897387
Klf4	16600	0.617194	0.897702
Ehmt1	77683	-1.63032	0.897702
Mrps12	24030	-0.66501	0.898713
Adamts15	235130	0.736483	0.899718
Itgb6	16420	0.606715	0.900313
Lect2	16841	0.987372	0.900313
Rps28	54127	0.695752	0.900313

Pink1	68943	0.665371	0.900313
Dusp22	105352	1.25825	0.904663
B4galt5	56336	0.653309	0.910668
S100a14	66166	0.678301	0.919247
Nudt4	71207	0.631808	0.925167
Naip1	17940	0.610205	0.925485
9530003J23Rik	77397	2.65071	0.925485
Onecut2	225631	-0.85326	0.925485
Gm14446	667373	2.43524	0.925485
Chgb	12653	-0.79658	0.951048
Csf3	12985	-1.91496	0.951048
2410015M20Rik	224904	0.699056	0.951048
Ubd	24108	0.945205	0.951875
C1ra	50909	0.838824	0.952698
Il1a	16175	-0.77429	0.953518
4930539E08Rik	207819	0.717314	0.954332
Sult1a1	20887	-1.22738	0.955143
Cxcl10	15945	-1.44378	0.95595
Ttbk2	140810	-1.16567	0.958358
Clcn2	12724	0.725157	0.961512
Gng5	14707	0.695344	0.961512
Naip5	17951	0.598466	0.961512
Rps29	20090	0.695535	0.961512
Bmp3	110075	-1.20653	0.965462
Ndufa2	17991	0.757749	0.966229
Ipmk	69718	0.578565	0.971725
Alad	17025	0.589312	0.972473
Rasl11a	68895	1.90158	0.973217
Col6a1	12833	0.782427	0.974694
Apob	238055	0.787726	0.974694
Fxyd2	11936	2.36319	0.976894
Ctla2a	13024	1.36991	0.976894
Gpc4	14735	0.598671	0.976894
Krt14	16664	-2.25225	0.976894
Pdgfa	18590	0.603037	0.976894
Pthlh	19227	-1.03152	0.976894
Rpl10a	19896	0.586112	0.976894
Snrpn	20646	-1.25656	0.976894
Rnasel	24014	0.603892	0.976894
Pias1	56469	-0.65825	0.976894
Tmem140	68487	0.837293	0.976894
4632434I11Rik	74041	-0.78652	0.976894
Clrn3	212070	0.709067	0.976894
Nt5c1a	230718	1.13793	0.976894
Pdap1	231887	-0.58924	0.976894
Ccdc141	545428	0.632376	0.976894
2210403K04Rik	1E+08	0.879452	0.976894

Gm9926	1.01E+08	0.657774	0.976894
Pdk4	27273	0.986658	0.979084
Nop16	28126	-0.57082	0.981146
Tmem208	66320	0.840379	0.981146
1810033B17Rik	69189	-2.27754	0.981146
Sult1b1	56362	0.617358	0.981826
Gtpbp2	56055	0.610125	0.983979
Mrpl17	27397	-0.57993	0.985319
Gm11062	1.01E+08	-1.00143	0.985319
Fam101b	76566	0.580523	0.985984
H19	14955	-0.69321	0.986646
Abca1	11303	0.187796	0.998356
Abcb7	11306	0.081927	0.998356
Abcg1	11307	0.137444	0.998356
Abi1	11308	1.37989	0.998356
Abl1	11350	-0.07454	0.998356
Abl2	11352	0.659018	0.998356
Acadl	11363	0.408624	0.998356
Acadm	11364	0.535827	0.998356
Acadvl	11370	-0.10867	0.998356
Acads	11409	-0.01156	0.998356
Slc33a1	11416	-0.07765	0.998356
Ace	11421	0.125107	0.998356
Apoc4	11425	0.588963	0.998356
Macf1	11426	-0.05699	0.998356
Aco1	11428	-0.18383	0.998356
Aco2	11429	0.035637	0.998356
Acox1	11430	0.050478	0.998356
Acp1	11431	0.029512	0.998356
Acp2	11432	0.276476	0.998356
Acp5	11433	0.240026	0.998356
Chrnb1	11443	0.340272	0.998356
Acta1	11459	-0.11518	0.998356
Actb	11461	0.099444	0.998356
Actg1	11465	-0.156	0.998356
Acta2	11475	-0.30873	0.998356
Acvr1	11477	-0.10737	0.998356
Acvr1b	11479	-0.02999	0.998356
Acvr2a	11480	-0.04229	0.998356
Aspa	11484	0.127544	0.998356
Ada	11486	0.36636	0.998356
Adam10	11487	0.178396	0.998356
Adam12	11489	0.094688	0.998356
Adam15	11490	-0.00197	0.998356
Adam17	11491	0.041191	0.998356
Adam19	11492	-0.07068	0.998356
Adam8	11501	0.052896	0.998356

Adam9	11502	0.129786	0.998356
Adamts1	11504	-0.4676	0.998356
Adcy6	11512	0.008163	0.998356
Adcy8	11514	-0.35465	0.998356
Adcy9	11515	0.380603	0.998356
Add1	11518	0.13923	0.998356
Adh5	11532	-0.43221	0.998356
Adk	11534	0.278353	0.998356
Adm	11535	0.383762	0.998356
Gpr182	11536	-0.07017	0.998356
Adnp	11538	0.167565	0.998356
Adprh	11544	0.147821	0.998356
Parp1	11545	0.074147	0.998356
Parp2	11546	-0.04504	0.998356
Adrb2	11555	0.168859	0.998356
Adsl	11564	-0.33348	0.998356
Adss	11566	-0.30092	0.998356
Avil	11567	-0.32907	0.998356
Aebp1	11568	0.595805	0.998356
Aebp2	11569	-0.80627	0.998356
Aga	11593	-0.19076	0.998356
Angpt2	11601	0.097501	0.998356
Agrn	11603	0.263099	0.998356
Gla	11605	-0.38475	0.998356
Agt	11606	-0.2176	0.998356
Agtrap	11610	-0.00128	0.998356
Ahr	11622	-0.03897	0.998356
Aif1	11629	-0.10785	0.998356
Aim1	11630	0.304324	0.998356
Aip	11632	0.061799	0.998356
Ak2	11637	-0.03083	0.998356
Ak4	11639	0.227961	0.998356
Akap1	11640	-0.10941	0.998356
Akap2	11641	0.115595	0.998356
Akt1	11651	0.218787	0.998356
Akt2	11652	-0.27246	0.998356
Alas1	11655	-0.136	0.998356
Alas2	11656	-1.19108	0.998356
Alcam	11658	0.425503	0.998356
Abcd1	11666	0.057953	0.998356
Aldh2	11669	0.225466	0.998356
Aldh3a2	11671	0.518735	0.998356
Aldoa	11674	-0.29875	0.998356
Aldoc	11676	-0.16418	0.998356
Akr1b3	11677	0.206964	0.998356
Alox12	11684	-0.5236	0.998356
Alox12e	11685	0.019248	0.998356

Alox5ap	11690	-0.79025	0.998356
Gfer	11692	-0.33087	0.998356
Amd1	11702	5.67555	0.998356
Ampd3	11717	0.186839	0.998356
Ang	11727	0.084257	0.998356
Ank	11732	-0.20989	0.998356
Ank3	11735	-0.64528	0.998356
Ankfy1	11736	-0.02169	0.998356
Anp32a	11737	-0.33062	0.998356
Slc25a4	11739	-0.51781	0.998356
Slc25a5	11740	-0.20393	0.998356
Anxa11	11744	-0.27264	0.998356
Anxa3	11745	-0.11808	0.998356
Anxa4	11746	0.277083	0.998356
Anxa5	11747	0.535898	0.998356
Anxa6	11749	-1.21867	0.998356
Anxa7	11750	0.10242	0.998356
Anxa8	11752	0.642349	0.998356
Prdx3	11757	0.117109	0.998356
Prdx6	11758	0.710997	0.998356
Ap1b1	11764	0.15139	0.998356
Ap1g1	11765	0.133917	0.998356
Ap1g2	11766	-0.07426	0.998356
Ap1m1	11767	-0.04966	0.998356
Ap1m2	11768	-0.28987	0.998356
Ap1s1	11769	0.20129	0.998356
Fabp4	11770	-1.02327	0.998356
Ap2a1	11771	-0.00677	0.998356
Ap2a2	11772	0.115185	0.998356
Ap2m1	11773	0.022166	0.998356
Ap3b1	11774	-0.2022	0.998356
Ap3d1	11776	0.079629	0.998356
Ap3s1	11777	-0.12542	0.998356
Ap3s2	11778	-0.0448	0.998356
Ap4m1	11781	0.133166	0.998356
Ap4s1	11782	-0.27253	0.998356
Apaf1	11783	0.12513	0.998356
Apbb2	11787	-0.33463	0.998356
Apc	11789	0.069095	0.998356
Apex1	11792	-0.32853	0.998356
Atg5	11793	0.242734	0.998356
Birc3	11796	0.154713	0.998356
Birc2	11797	0.094178	0.998356
Xiap	11798	0.10087	0.998356
Birc5	11799	-0.32739	0.998356
Api5	11800	-0.12438	0.998356
Aplp1	11803	0.068674	0.998356

Aplp2	11804	0.024975	0.998356
Apoa2	11807	-0.84282	0.998356
Apobec2	11811	0.121302	0.998356
Apoc2	11813	0.671603	0.998356
Apod	11815	-0.18457	0.998356
Apoe	11816	0.729913	0.998356
Nr2f2	11819	-0.391	0.998356
App	11820	-0.04921	0.998356
Aprt	11821	0.536998	0.998356
Aqp1	11826	0.238177	0.998356
Aqp3	11828	-0.13137	0.998356
Aqp4	11829	-0.19465	0.998356
Aqp8	11833	0.05051	0.998356
Aqr	11834	-0.0906	0.998356
Araf	11836	0.853762	0.998356
Rplp0	11837	-0.2159	0.998356
Arc	11838	-0.60045	0.998356
Areg	11839	0.175181	0.998356
Arf1	11840	0.913217	0.998356
Arf3	11842	0.030808	0.998356
Arf4	11843	-0.41861	0.998356
Arf5	11844	0.339529	0.998356
Arf6	11845	0.218859	0.998356
Arg1	11846	0.116761	0.998356
Arg2	11847	-0.41342	0.998356
Rhoa	11848	0.153771	0.998356
Rhob	11852	0.335571	0.998356
Rhoc	11853	0.438641	0.998356
Rhod	11854	0.671399	0.998356
Arhgap5	11855	0.012357	0.998356
Arhgdib	11857	-0.12157	0.998356
Arl4a	11861	-0.33258	0.998356
Arnt	11863	0.013198	0.998356
Arnt2	11864	-0.24989	0.998356
Arntl	11865	0.048643	0.998356
Arpc1b	11867	0.087155	0.998356
Arvcf	11877	-0.0151	0.998356
Arsb	11881	0.013767	0.998356
Asah1	11886	0.43709	0.998356
Rab27a	11891	-0.28521	0.998356
Ass1	11898	-2.52634	0.998356
Zfhx3	11906	-0.04791	0.998356
Ate1	11907	-0.10252	0.998356
Atf1	11908	0.023913	0.998356
Atf2	11909	-0.30846	0.998356
Atf3	11910	0.416238	0.998356
Atf4	11911	0.118106	0.998356

Atm	11920	-0.0216	0.998356
Atoh1	11921	-0.52854	0.998356
Atox1	11927	-0.04478	0.998356
Atp1a1	11928	-0.13231	0.998356
Atp1b1	11931	0.108937	0.998356
Atp1b3	11933	-0.08547	0.998356
Atp2a2	11938	0.077085	0.998356
Atp5a1	11946	-0.28764	0.998356
Atp5b	11947	-0.10213	0.998356
Atp5c1	11949	0.144766	0.998356
Atp5f1	11950	-0.09721	0.998356
Atp5g1	11951	0.504067	0.998356
Atp5j	11957	0.411507	0.998356
Atp5k	11958	0.637925	0.998356
Atp6v1a	11964	-0.11585	0.998356
Atp6v1b2	11966	0.071805	0.998356
Atp6v0d1	11972	0.288476	0.998356
Atp6v1e1	11973	0.271919	0.998356
Atp6v0e	11974	0.184128	0.998356
Atp6v0a1	11975	-0.27999	0.998356
Atp7a	11977	0.184077	0.998356
Atp8a1	11980	0.444128	0.998356
Atp9a	11981	0.201079	0.998356
Atp10a	11982	0.34658	0.998356
Atpif1	11983	-0.07965	0.998356
Atp6v0c	11984	0.311114	0.998356
Slc7a1	11987	-0.20682	0.998356
Atrn	11990	0.358637	0.998356
Hnrnpd	11991	-0.30947	0.998356
Auh	11992	0.078264	0.998356
Aup1	11993	0.139826	0.998356
Axin1	12005	-0.03535	0.998356
Axin2	12006	0.13877	0.998356
B2m	12010	-0.11307	0.998356
Bach1	12013	0.029449	0.998356
Bad	12015	-0.11827	0.998356
Bag1	12017	0.10515	0.998356
Bak1	12018	0.102505	0.998356
Bcap29	12033	0.540848	0.998356
Phb2	12034	-0.04651	0.998356
Bcat2	12036	0.003367	0.998356
Bckdha	12039	0.612436	0.998356
Bckdhb	12040	-0.50351	0.998356
Bckdk	12041	-0.25052	0.998356
Bcl10	12042	0.008496	0.998356
Bcl2	12043	-0.1441	0.998356
Bcl2a1a	12044	-0.46618	0.998356

Bcl2a1b	12045	-0.13328	0.998356
Bcl2a1d	12047	-1.03797	0.998356
Bcl2l1	12048	-0.06234	0.998356
Bcl2l2	12050	-0.00601	0.998356
Bcl3	12051	-0.18543	0.998356
Bcl6	12053	-0.0335	0.998356
Bcl7b	12054	0.213411	0.998356
Bcl7c	12055	0.646796	0.998356
Bet1	12068	0.006666	0.998356
Bex2	12069	0.090746	0.998356
Ngfrap1	12070	-1.39987	0.998356
Glb1	12091	0.203812	0.998356
Bgn	12111	0.235268	0.998356
Bicd1	12121	0.040775	0.998356
Bid	12122	0.198393	0.998356
Bik	12124	-0.17242	0.998356
Bcl2l11	12125	-0.39476	0.998356
Prdm1	12142	-0.48336	0.998356
Blm	12144	-0.27937	0.998356
Bmi1	12151	0.280391	0.998356
Bmp1	12153	0.121385	0.998356
Bmp2	12156	0.371544	0.998356
Bmp4	12159	0.165739	0.998356
Bmp5	12160	0.731324	0.998356
Bmp7	12162	0.409357	0.998356
Bmp8b	12164	0.60382	0.998356
Bmpr1a	12166	0.088833	0.998356
Bmpr2	12168	0.116626	0.998356
Bnip2	12175	-0.64756	0.998356
Bnip3	12176	0.359204	0.998356
Bnip3l	12177	-0.01772	0.998356
Bop1	12181	0.31047	0.998356
Bpgm	12183	0.162203	0.998356
Brca1	12189	-0.22246	0.998356
Brca2	12190	-0.51184	0.998356
Zfp36l1	12192	-0.42023	0.998356
Zfp36l2	12193	-0.35214	0.998356
Birc6	12211	0.093171	0.998356
Bsg	12215	0.023421	0.998356
Btc	12223	-0.13013	0.998356
Klf5	12224	-0.11668	0.998356
Btg1	12226	0.252765	0.998356
Btg2	12227	-0.0889	0.998356
Btrc	12234	-0.25019	0.998356
Bub1	12235	-0.77496	0.998356
Bub1b	12236	-0.08913	0.998356
Bub3	12237	0.166298	0.998356

Commd3	12238	-0.00291	0.998356
Tspo	12257	0.285711	0.998356
Serping1	12258	0.659698	0.998356
C1qa	12259	0.103764	0.998356
C1qb	12260	-0.25426	0.998356
C1qbp	12261	-0.26134	0.998356
C1qc	12262	0.351325	0.998356
C2	12263	-0.34651	0.998356
C3	12266	-0.07734	0.998356
C4b	12268	0.884738	0.998356
C4bp	12269	-0.08621	0.998356
C5ar1	12273	-0.12812	0.998356
Hyou1	12282	-0.06712	0.998356
Cab39	12283	0.435923	0.998356
Cacnb3	12297	0.145208	0.998356
Pdia4	12304	-0.05285	0.998356
Ddr1	12305	0.113356	0.998356
Anxa2	12306	0.474962	0.998356
S100g	12309	0.603977	0.998356
Calca	12310	-4.74655	0.998356
Calm1	12313	0.111652	0.998356
Calm2	12314	0.018422	0.998356
Calm3	12315	0.127556	0.998356
Aspm	12316	-0.14652	0.998356
Calr	12317	0.101939	0.998356
Calu	12321	-0.057	0.998356
Camk2g	12325	-0.51245	0.998356
Caml	12328	-0.1457	0.998356
Canx	12330	-0.51286	0.998356
Cap1	12331	0.532268	0.998356
Capn1	12333	0.568991	0.998356
Capn2	12334	0.321833	0.998356
Capns1	12336	0.231686	0.998356
Capn5	12337	0.514957	0.998356
Capn7	12339	0.168358	0.998356
Capza1	12340	0.121949	0.998356
Capza2	12343	0.064566	0.998356
Capzb	12345	-0.04744	0.998356
Car1	12346	0.390007	0.998356
Car2	12349	-0.3424	0.998356
Car4	12351	-0.83769	0.998356
Cat	12359	-0.19992	0.998356
Cask	12361	0.138979	0.998356
Casp1	12362	0.149673	0.998356
Casp4	12363	0.197121	0.998356
Casp12	12364	0.239679	0.998356
Casp2	12366	-0.33799	0.998356

Casp3	12367	0.068002	0.998356
Casp6	12368	-0.13327	0.998356
Casp7	12369	0.284525	0.998356
Casp8	12370	0.006409	0.998356
Casp9	12371	0.365269	0.998356
Casq1	12372	0.172716	0.998356
Ctnna1	12385	-0.09976	0.998356
Ctnnb1	12387	0.19845	0.998356
Ctnnd1	12388	0.17811	0.998356
Cav1	12389	-1.22736	0.998356
Cav2	12390	0.281066	0.998356
Runx1	12394	-0.24195	0.998356
Cbfa2t2	12396	-1.47916	0.998356
Cbfb	12400	0.065195	0.998356
Cbl	12402	-0.20278	0.998356
Serpinh1	12406	0.003757	0.998356
Cbr1	12408	-0.08384	0.998356
Cbr2	12409	-0.65878	0.998356
Cbs	12411	-0.58915	0.998356
Cbx1	12412	0.257354	0.998356
Cbx2	12416	-0.01265	0.998356
Cbx3	12417	0.167963	0.998356
Cbx4	12418	0.000711	0.998356
Cbx5	12419	-0.56612	0.998356
Rb1cc1	12421	0.289247	0.998356
Ccna2	12428	-0.14155	0.998356
Ccnd1	12443	-0.59245	0.998356
Ccnd2	12444	-0.11434	0.998356
Ccnd3	12445	0.327024	0.998356
Ccne1	12447	-0.62883	0.998356
Ccne2	12448	-0.41223	0.998356
Ccnf	12449	-0.23167	0.998356
Ccng1	12450	-0.19072	0.998356
Ccng2	12452	0.5109	0.998356
Ccni	12453	0.424296	0.998356
Ccnk	12454	-0.30897	0.998356
Ccnt1	12455	0.254809	0.998356
Ccrn4l	12457	-0.00228	0.998356
Ccs	12460	-0.08271	0.998356
Cct2	12461	-0.35763	0.998356
Cct3	12462	0.083189	0.998356
Cct4	12464	-0.27286	0.998356
Cct5	12465	-0.2258	0.998356
Cct6a	12466	-0.40204	0.998356
Cct7	12468	-0.32751	0.998356
Cct8	12469	0.184955	0.998356
Cd14	12475	-0.41498	0.998356

Cd151	12476	-0.17892	0.998356
Cd1d1	12479	-0.173	0.998356
Cd24a	12484	-0.36371	0.998356
Cd2ap	12488	0.262687	0.998356
Cd33	12489	0.154704	0.998356
Cd34	12490	-1.11566	0.998356
Scarb2	12492	0.065325	0.998356
Entpd1	12495	-0.14604	0.998356
Entpd2	12496	0.417227	0.998356
Entpd6	12497	0.170822	0.998356
Entpd5	12499	-0.01023	0.998356
Cd3g	12502	-0.75668	0.998356
Cd44	12505	0.56897	0.998356
Cd53	12508	-0.35696	0.998356
Cd68	12514	-0.05936	0.998356
Cd81	12520	-0.1417	0.998356
Cd82	12521	1.9035	0.998356
Cd83	12522	-0.17155	0.998356
Cd9	12527	-0.02857	0.998356
Cdc25a	12530	-0.2226	0.998356
Cdc25b	12531	-0.11915	0.998356
Cdc25c	12532	0.339958	0.998356
Cdk1	12534	0.02895	0.998356
Cdk11b	12537	-0.19605	0.998356
Cdc37	12539	-0.42001	0.998356
Cdc42	12540	0.344715	0.998356
Cdc45	12544	-0.00908	0.998356
Cdc7	12545	-0.12159	0.998356
Cdh1	12550	0.21387	0.998356
Cdh11	12552	0.373187	0.998356
Cdh13	12554	-0.04497	0.998356
Cdh17	12557	0.538742	0.998356
Cdh5	12562	0.550434	0.998356
Cdk2	12566	-0.02942	0.998356
Cdk4	12567	-0.0437	0.998356
Cdk5	12568	-0.24695	0.998356
Cdk6	12571	0.074567	0.998356
Cdk7	12572	0.055591	0.998356
Cdkn1a	12575	0.396552	0.998356
Cdkn1b	12576	-0.17535	0.998356
Cdkn1c	12577	1.36548	0.998356
Cdkn2a	12578	0.836639	0.998356
Cdkn2b	12579	0.392337	0.998356
Cdkn2d	12581	0.023969	0.998356
Cdo1	12583	-0.04027	0.998356
Cdr2	12585	-0.14776	0.998356
Ift81	12589	0.259364	0.998356

Cdx1	12590	-0.04564	0.998356
Cdx2	12591	0.33697	0.998356
Cdyl	12593	-0.46641	0.998356
Cebpa	12606	-0.27397	0.998356
Cebpz	12607	-0.23747	0.998356
Cebpb	12608	0.276998	0.998356
Cebpd	12609	-0.27771	0.998356
Cebpg	12611	-0.00638	0.998356
Celsr1	12614	0.176824	0.998356
Cenpa	12615	0.337448	0.998356
Cenpb	12616	0.416455	0.998356
Cenpc1	12617	-0.17962	0.998356
Cetn3	12626	-0.33293	0.998356
Cfh	12628	0.157467	0.998356
Cfi	12630	-0.20403	0.998356
Cfl1	12631	-0.25069	0.998356
Cfl2	12632	0.099822	0.998356
Cflar	12633	0.075344	0.998356
Cftr	12638	0.123341	0.998356
Chd1	12648	-0.11475	0.998356
Chek1	12649	-0.17474	0.998356
Chkb	12651	0.584713	0.998356
Chga	12652	-0.51096	0.998356
Chka	12660	-0.21908	0.998356
Chm	12662	-0.06142	0.998356
Chml	12663	-0.10197	0.998356
Chuk	12675	0.776038	0.998356
Cideb	12684	0.340493	0.998356
Inadl	12695	-0.66994	0.998356
Cirbp	12696	0.112612	0.998356
Cish	12700	0.016689	0.998356
Socs3	12702	0.010285	0.998356
Socs1	12703	-1.04713	0.998356
Cit	12704	-0.35479	0.998356
Cited1	12705	-0.19164	0.998356
Ckmt1	12716	0.128118	0.998356
Coro1a	12721	0.293454	0.998356
Clcn3	12725	0.319969	0.998356
Clcn4-2	12727	0.058613	0.998356
Clcn5	12728	0.020032	0.998356
Clns1a	12729	-0.35159	0.998356
Cldn3	12739	-0.13009	0.998356
Cldn4	12740	0.133194	0.998356
Clk1	12747	0.649805	0.998356
Clk2	12748	-0.15374	0.998356
Clk4	12750	0.099514	0.998356
Tpp1	12751	0.025488	0.998356

Cln3	12752	-0.25471	0.998356
Clock	12753	-0.19557	0.998356
Clta	12757	-0.29768	0.998356
Clu	12759	0.041392	0.998356
Cmas	12764	0.228918	0.998356
Cxcr2	12765	0.380841	0.998356
Ccr1	12768	0.086906	0.998356
Cxcr7	12778	0.316848	0.998356
Abcc2	12780	0.265951	0.998356
Cnbp	12785	-0.02669	0.998356
Cnih	12793	0.415084	0.998356
Plk3	12795	0.748528	0.998356
Cnn1	12797	-3.45336	0.998356
Cnn2	12798	0.035204	0.998356
Cnp	12799	-0.33905	0.998356
Hps3	12807	0.533256	0.998356
Coil	12812	-0.60878	0.998356
Col12a1	12816	0.501755	0.998356
Col15a1	12819	0.169408	0.998356
Col18a1	12822	0.436256	0.998356
Col3a1	12825	0.561816	0.998356
Col4a1	12826	-0.01926	0.998356
Col4a2	12827	0.005821	0.998356
Col5a1	12831	0.008846	0.998356
Col5a2	12832	0.140295	0.998356
Col6a2	12834	0.276452	0.998356
Col6a3	12835	-0.14111	0.998356
Col9a2	12840	0.128034	0.998356
Col9a3	12841	0.092355	0.998356
Col1a1	12842	0.138922	0.998356
Col1a2	12843	0.359623	0.998356
Comt	12846	0.03756	0.998356
Copa	12847	0.115422	0.998356
Cops2	12848	-0.15414	0.998356
Coq7	12850	-0.02509	0.998356
Cox17	12856	0.336314	0.998356
Cox4i1	12857	0.348354	0.998356
Cox5a	12858	-0.11683	0.998356
Cox5b	12859	0.54676	0.998356
Cox6a1	12861	0.106975	0.998356
Cox6c	12864	-0.41017	0.998356
Cox7a1	12865	-0.47907	0.998356
Cox7a2	12866	0.029085	0.998356
Cox8a	12868	0.093937	0.998356
Cp	12870	0.262052	0.998356
Cpd	12874	0.205807	0.998356
Cpe	12876	-0.17944	0.998356

Cpox	12892	-0.42316	0.998356
Cpt1a	12894	0.250317	0.998356
Cpt2	12896	0.12465	0.998356
Cradd	12905	-0.31827	0.998356
Crat	12908	0.270681	0.998356
Crcp	12909	0.471478	0.998356
Creb1	12912	-0.38782	0.998356
Creb3	12913	0.264588	0.998356
Crebbp	12914	0.004162	0.998356
Atf6b	12915	-0.37813	0.998356
Crem	12916	-0.79796	0.998356
Bcar1	12927	-0.09275	0.998356
Crk	12928	0.263306	0.998356
Crkl	12929	-0.04842	0.998356
Dpysl2	12934	0.030363	0.998356
Dmbt1	12945	0.944291	0.998356
Cr1l	12946	0.336983	0.998356
Cry1	12952	-0.02881	0.998356
Cry2	12953	0.032842	0.998356
Cryab	12955	0.433071	0.998356
Cryz	12972	0.179482	0.998356
Cs	12974	-0.08074	0.998356
Csf1	12977	-0.92214	0.998356
Csf1r	12978	0.141424	0.998356
Csf2	12981	-0.93585	0.998356
Csf2ra	12982	0.003642	0.998356
Csf2rb	12983	-0.10148	0.998356
Csf3r	12986	0.379953	0.998356
Csk	12988	0.233765	0.998356
Csnk2a1	12995	0.113676	0.998356
Csnk2a2	13000	-0.06463	0.998356
Csnk2b	13001	-0.16017	0.998356
Dnajc5	13002	0.291778	0.998356
Smc3	13006	-0.17298	0.998356
Csrp1	13007	-0.31794	0.998356
Csrp2	13008	0.341798	0.998356
Cst3	13010	-0.11408	0.998356
Cstb	13014	0.226936	0.998356
Ctbp1	13016	-4.94369	0.998356
Ctbp2	13017	0.213802	0.998356
Ctcf	13018	-0.09064	0.998356
Pcyt1a	13026	0.123401	0.998356
Ctsb	13030	0.137581	0.998356
Ctsc	13032	0.001838	0.998356
Ctsd	13033	0.241222	0.998356
Ctse	13034	0.050846	0.998356
Ctsl	13039	0.00543	0.998356

Ctss	13040	0.417892	0.998356
Cttn	13043	0.244753	0.998356
Celf1	13046	-2.23185	0.998356
Cux1	13047	-0.18935	0.998356
Cx3cr1	13051	0.355652	0.998356
Cxadr	13052	-0.14008	0.998356
Cyb561	13056	0.126396	0.998356
Cyba	13057	-0.1873	0.998356
Cybb	13058	0.008621	0.998356
Cycs	13063	-0.22365	0.998356
Cyp21a1	13079	0.627864	0.998356
Cyp2d10	13101	-0.10316	0.998356
Cyp3a13	13113	-0.25711	0.998356
Cyp51	13121	-0.03427	0.998356
Dab2	13132	0.526173	0.998356
Dach1	13134	0.460161	0.998356
Dad1	13135	-0.22701	0.998356
Dag1	13138	0.228824	0.998356
Dapk2	13143	-0.32675	0.998356
Dapk3	13144	-0.07534	0.998356
Daxx	13163	0.126981	0.998356
Dbi	13167	-1.08225	0.998356
Dbnl	13169	0.183491	0.998356
Dbt	13171	0.169673	0.998356
Eci1	13177	-0.02642	0.998356
Dck	13178	-0.11644	0.998356
Pcbd1	13180	0.269349	0.998356
Dscr3	13185	0.119856	0.998356
Dctn1	13191	0.290424	0.998356
Ddb1	13194	0.173798	0.998356
Ddc	13195	0.064963	0.998356
Asap1	13196	0.423895	0.998356
Gadd45a	13197	0.028772	0.998356
Ddit3	13198	-0.17514	0.998356
Ddost	13200	-0.11743	0.998356
Ddt	13202	-0.38162	0.998356
Dhx15	13204	-0.11498	0.998356
Ddx3x	13205	0.207277	0.998356
Ddx5	13207	-0.06889	0.998356
Ddx6	13209	-0.98364	0.998356
Dhx9	13211	0.179209	0.998356
Defa-rs1	13218	-1.29693	0.998356
Defa3	13237	0.812044	0.998356
Defa4	13238	1.76703	0.998356
Defa5	13239	-0.05332	0.998356
Defa6	13240	10	0.998356
Degs1	13244	-0.26363	0.998356

Dffa	13347	-0.1591	0.998356
Dgat1	13350	-0.23153	0.998356
Dgcr6	13353	0.155787	0.998356
Dgcr2	13356	0.650269	0.998356
Slc25a1	13358	0.153031	0.998356
Dhcr7	13360	-0.29654	0.998356
Dhfr	13361	-0.06083	0.998356
Diap1	13367	0.303752	0.998356
Dffb	13368	0.651925	0.998356
Dio1	13370	-0.33105	0.998356
Dld	13382	0.284057	0.998356
Dlg1	13383	-0.12226	0.998356
Mpp3	13384	-0.21373	0.998356
Dmpk	13400	-10	0.998356
Dmwd	13401	-0.06248	0.998356
Dnahc8	13417	0.081853	0.998356
Dnajc1	13418	-0.08235	0.998356
Dnase2a	13423	0.282565	0.998356
Dync1h1	13424	0.057689	0.998356
Dync1i2	13427	-0.27966	0.998356
Dnm2	13430	0.0558	0.998356
Dnmt1	13433	-0.25715	0.998356
Dnmt3a	13435	-0.08697	0.998356
Dnpep	13437	0.33273	0.998356
Cdk2ap1	13445	0.529858	0.998356
Dok1	13448	-0.05028	0.998356
Dok2	13449	0.895815	0.998356
Reep5	13476	-0.19715	0.998356
Dpagt1	13478	0.13197	0.998356
Dpm1	13480	0.15692	0.998356
Dpm2	13481	-0.4843	0.998356
Dpp4	13482	-0.4205	0.998356
Dr1	13486	-0.10771	0.998356
Drg1	13494	-0.34636	0.998356
Drg2	13495	0.012864	0.998356
Arid3a	13496	0.358427	0.998356
Atn1	13498	-0.24456	0.998356
Dsc2	13506	0.023269	0.998356
Dsc3	13507	-0.30369	0.998356
Dsg2	13511	0.477007	0.998356
Dst	13518	0.234701	0.998356
Slc26a2	13521	-0.09454	0.998356
Adam28	13522	1.85905	0.998356
Dtna	13527	-0.24787	0.998356
Dvl1	13542	0.406707	0.998356
Dvl2	13543	-0.03438	0.998356
Dvl3	13544	-0.11173	0.998356

Dyrk1a	13548	0.065214	0.998356
Dyrk1b	13549	0.369164	0.998356
E2f3	13557	0.043777	0.998356
E2f5	13559	0.092654	0.998356
E4f1	13560	0.1554	0.998356
Mapre1	13589	0.007027	0.998356
Ebp	13595	0.185035	0.998356
Ecm1	13601	-0.94553	0.998356
Sparcl1	13602	0.197803	0.998356
Ect2	13605	0.355933	0.998356
S1pr1	13609	0.073496	0.998356
Edn1	13614	-0.23679	0.998356
Edn3	13616	0.690507	0.998356
Ednra	13617	0.474994	0.998356
Phc1	13619	0.835246	0.998356
Eed	13626	-0.30554	0.998356
Eef1a1	13627	-0.23748	0.998356
Eef2	13629	-0.18989	0.998356
Eef2k	13631	-0.23332	0.998356
Efna1	13636	0.298343	0.998356
Efna4	13639	-0.09388	0.998356
Efnb1	13641	0.126434	0.998356
Efnb2	13642	0.19732	0.998356
Egfr	13649	0.570024	0.998356
Rhbdf1	13650	-0.01532	0.998356
Egr1	13653	0.246987	0.998356
Ehd1	13660	0.10553	0.998356
Ehf	13661	-0.16837	0.998356
Ei24	13663	-0.3738	0.998356
Eif1a	13664	-0.10909	0.998356
Eif2s1	13665	-0.59382	0.998356
Eif2ak3	13666	0.296932	0.998356
Eif2b4	13667	-0.25762	0.998356
Eif3a	13669	-0.20133	0.998356
Ddx19a	13680	-0.08593	0.998356
Eif4a1	13681	-0.74469	0.998356
Eif4a2	13682	-0.44543	0.998356
Eif4e	13684	-0.11252	0.998356
Eif4ebp1	13685	0.191831	0.998356
Eif4ebp2	13688	-0.15461	0.998356
Eif4g2	13690	0.57436	0.998356
Elf1	13709	0.148201	0.998356
Elf3	13710	0.020604	0.998356
Elk1	13712	0.005159	0.998356
Elk3	13713	-0.17618	0.998356
Elk4	13714	0.023074	0.998356
Ell	13716	0.321541	0.998356

Aimp1	13722	0.043522	0.998356
Emb	13723	-0.70533	0.998356
Emd	13726	-0.33313	0.998356
Mark2	13728	0.149697	0.998356
Emp1	13730	0.293935	0.998356
Emp2	13731	0.064802	0.998356
Emp3	13732	0.02794	0.998356
Enah	13800	-0.70271	0.998356
Enc1	13803	-0.19228	0.998356
Eng	13805	0.704282	0.998356
Eno1	13806	0.203507	0.998356
Eno3	13808	-10	0.998356
Epas1	13819	0.059325	0.998356
Epb4.1l1	13821	0.472607	0.998356
Epb4.1l2	13822	-0.13981	0.998356
Epb4.1l3	13823	-0.2222	0.998356
Epb4.1l4a	13824	0.089764	0.998356
Stom	13830	0.425922	0.998356
Epc1	13831	0.457725	0.998356
Epha1	13835	-0.15914	0.998356
Epha2	13836	0.35629	0.998356
Epha4	13838	-0.31862	0.998356
Epha7	13841	-1.07485	0.998356
Ephb2	13844	0.306619	0.998356
Ephb3	13845	-0.01597	0.998356
Ephb4	13846	0.69111	0.998356
Ephb6	13848	0.031971	0.998356
Ephx1	13849	-0.40189	0.998356
Ephx2	13850	0.645448	0.998356
Epn1	13854	-0.28864	0.998356
Epn2	13855	-0.19896	0.998356
Eps15	13858	0.109884	0.998356
Eps15l1	13859	0.039911	0.998356
Eps8	13860	0.583139	0.998356
Nr2f6	13864	0.213795	0.998356
Erbb2	13866	0.329964	0.998356
Erbb3	13867	0.408995	0.998356
Ercc1	13870	-0.15634	0.998356
Ercc2	13871	-0.29291	0.998356
Ercc3	13872	-0.28454	0.998356
Ereg	13874	-0.79064	0.998356
Erf	13875	-0.15712	0.998356
Erh	13877	-0.0735	0.998356
Esd	13885	0.14605	0.998356
Amz2	13929	0.137076	0.998356
Esr1	13982	-0.11954	0.998356
Smarcad1	13990	0.039612	0.998356

Khdrbs3	13992	0.804792	0.998356
Fgd6	13998	0.028064	0.998356
Drosha	14000	-3.41733	0.998356
Chchd2	14004	0.04799	0.998356
Etv6	14011	-0.30811	0.998356
Mpzl2	14012	-0.39685	0.998356
Mecom	14013	0.096486	0.998356
Evi2a	14017	0.486064	0.998356
Evi5	14020	-0.01489	0.998356
Bcl11a	14025	0.068309	0.998356
Evl	14026	0.155661	0.998356
Evpl	14027	0.145483	0.998356
Evx1	14028	0.642518	0.998356
Ewsr1	14030	0.124055	0.998356
Expi	14038	-0.38316	0.998356
Ext1	14042	-0.05645	0.998356
Ext2	14043	-0.25783	0.998356
Eya2	14049	-0.56926	0.998356
Eya3	14050	-0.05127	0.998356
Ezh1	14055	0.104895	0.998356
Ezh2	14056	-0.19594	0.998356
Sfxn1	14057	-0.33068	0.998356
F2r	14062	0.396177	0.998356
F3	14066	0.246489	0.998356
F8a	14070	0.077471	0.998356
Faah	14073	-0.16129	0.998356
Acsl1	14081	0.031606	0.998356
Fadd	14082	-0.09506	0.998356
Ptk2	14083	-0.26134	0.998356
Faf1	14084	0.386758	0.998356
Fscn1	14086	-0.26078	0.998356
Fancc	14088	0.998675	0.998356
Fas	14102	0.274278	0.998356
Fasn	14104	-0.20632	0.998356
Srsf10	14105	-0.31269	0.998356
Fat1	14107	0.274351	0.998356
Fau	14109	0.050109	0.998356
Fbln1	14114	-0.31506	0.998356
Fbn1	14118	0.624358	0.998356
Fbp2	14120	0.083913	0.998356
Fbrs	14123	-0.31743	0.998356
Fcer1g	14127	-0.08334	0.998356
Fcgr2b	14130	-0.36518	0.998356
Fcgr3	14131	-0.08067	0.998356
Fcgrt	14132	0.163474	0.998356
Fdft1	14137	0.187049	0.998356
Fdx1	14148	0.003892	0.998356

Fech	14151	0.22261	0.998356
Fem1a	14154	0.08934	0.998356
Fem1b	14155	0.024651	0.998356
Fen1	14156	-0.12476	0.998356
Fert2	14158	-0.08699	0.998356
Fes	14159	-0.00301	0.998356
Lgr5	14160	0.327829	0.998356
Fgf1	14164	-0.11574	0.998356
Fgf3	14174	0.527742	0.998356
Fgf7	14178	0.051517	0.998356
Fgfbp1	14181	-0.38989	0.998356
Fgfr2	14183	-0.04255	0.998356
Fgfr4	14186	0.159642	0.998356
Akr1b8	14187	0.029135	0.998356
Fgl2	14190	0.019995	0.998356
Fh1	14194	-0.02582	0.998356
Fhit	14198	0.146745	0.998356
Fhl1	14199	0.579497	0.998356
Fhl2	14200	0.425353	0.998356
Fhl3	14201	-0.50227	0.998356
Ppm1g	14208	-0.10003	0.998356
Smc2	14211	-0.27846	0.998356
Sh3pxd2a	14218	0.598305	0.998356
Ctgf	14219	0.55064	0.998356
Fkbp1a	14225	-0.0157	0.998356
Fkbp2	14227	-0.3445	0.998356
Fkbp4	14228	-0.32597	0.998356
Fkbp5	14229	-0.90765	0.998356
Fkbp7	14231	-0.26289	0.998356
Fkbp8	14232	0.129359	0.998356
Foxm1	14235	-0.06032	0.998356
Foxn2	14236	-0.24956	0.998356
Lpin1	14245	-0.54611	0.998356
Fli1	14247	0.224324	0.998356
Flii	14248	0.47124	0.998356
Flot1	14251	0.490407	0.998356
Flot2	14252	0.100374	0.998356
Fmn1	14260	0.323562	0.998356
Fmr1	14265	0.002077	0.998356
Fn1	14268	-0.21668	0.998356
Fnbp1	14269	-0.44223	0.998356
Srgap2	14270	0.079188	0.998356
Fnta	14272	0.246376	0.998356
Fos	14281	0.333208	0.998356
Fosl1	14283	0.275385	0.998356
Fosl2	14284	-0.06707	0.998356
Fpgs	14287	-0.39408	0.998356

Fxn	14297	-0.55766	0.998356
Ncs1	14299	-0.51465	0.998356
Frg1	14300	0.080593	0.998356
Frk	14302	0.276329	0.998356
Brd2	14312	0.099677	0.998356
Fst	14313	0.289036	0.998356
Fstl1	14314	0.473964	0.998356
Fth1	14319	0.718045	0.998356
Ftl1	14325	0.23537	0.998356
Aktip	14339	0.130138	0.998356
Fut2	14344	0.400978	0.998356
Fut4	14345	-0.06231	0.998356
Fut9	14348	0.047766	0.998356
Fv1	14349	-0.31264	0.998356
Fxc1	14356	-0.59631	0.998356
Fxr1	14359	-0.26052	0.998356
Fyn	14360	0.626637	0.998356
Fzd4	14366	-0.50572	0.998356
Fzd5	14367	-0.01806	0.998356
Fzd6	14368	0.162671	0.998356
Fzd7	14369	0.335003	0.998356
G0s2	14373	0.301264	0.998356
Xrcc6	14375	-0.20249	0.998356
Ganab	14376	-0.13877	0.998356
G6pdx	14381	0.092556	0.998356
Slc37a4	14385	-0.26919	0.998356
Gaa	14387	-0.0717	0.998356
Gab1	14388	0.187794	0.998356
Gab2	14389	-0.15169	0.998356
Gabpa	14390	0.152214	0.998356
Gabpb1	14391	0.036246	0.998356
Galc	14420	0.006254	0.998356
B4galnt1	14421	1.43291	0.998356
B4galnt2	14422	-0.21979	0.998356
Galnt1	14423	-0.02089	0.998356
Galnt3	14425	0.391763	0.998356
Galnt4	14426	-0.18465	0.998356
Galt	14430	-0.04541	0.998356
Gapdh	14433	-0.10679	0.998356
Gart	14450	0.007294	0.998356
Gas1	14451	0.578955	0.998356
Gas5	14455	-0.07045	0.998356
Gas6	14456	-0.1049	0.998356
Gata5	14464	0.306049	0.998356
Gata6	14465	0.303217	0.998356
Gba	14466	0.31553	0.998356
Gbas	14467	0.00248	0.998356

Gbp2	14469	-0.07552	0.998356
Rabac1	14470	0.221432	0.998356
Usp15	14479	-0.06183	0.998356
Mtpn	14489	-0.23783	0.998356
Gcg	14526	-1.49468	0.998356
Gch1	14528	0.469335	0.998356
Bloc1s1	14533	-0.29664	0.998356
Kat2a	14534	-0.10087	0.998356
Nr6a1	14536	0.160429	0.998356
Gcnt1	14537	0.517732	0.998356
Gcnt2	14538	0.197249	0.998356
Gda	14544	0.396606	0.998356
Gdap2	14547	0.084178	0.998356
Mrps33	14548	0.144009	0.998356
Gpd1	14555	-0.42597	0.998356
Gdf11	14561	0.603246	0.998356
Gdi1	14567	-0.06492	0.998356
Gdi2	14569	0.016699	0.998356
Gpd2	14571	-0.1005	0.998356
Gem	14579	0.696242	0.998356
Gfpt1	14583	-0.05135	0.998356
Gfpt2	14584	-0.13533	0.998356
Ggps1	14593	-0.07511	0.998356
B4galt1	14595	0.371032	0.998356
Ggt1	14598	0.841632	0.998356
Ghr	14600	0.414716	0.998356
Tsc22d3	14605	-0.0287	0.998356
Gja1	14609	-0.42383	0.998356
Gjb1	14618	0.345736	0.998356
Gjb2	14619	-0.3776	0.998356
Gjb4	14621	0.237737	0.998356
Gjb5	14622	0.33534	0.998356
Ostm1	14628	0.032406	0.998356
Gclc	14629	0.302249	0.998356
Gclm	14630	0.355751	0.998356
Galk1	14635	0.226185	0.998356
Glul	14645	0.213783	0.998356
Hagh	14651	0.216487	0.998356
Glrp1	14659	-0.72792	0.998356
Gls	14660	0.287373	0.998356
Glud1	14661	0.388036	0.998356
Slc6a9	14664	0.453833	0.998356
Gm2a	14667	-0.17918	0.998356
Gnl1	14670	0.061431	0.998356
Gna11	14672	0.52888	0.998356
Gna12	14673	0.069576	0.998356
Gna13	14674	-0.0356	0.998356

Gna14	14675	0.240609	0.998356
Gna15	14676	0.633634	0.998356
Gnai1	14677	0.390996	0.998356
Gnai2	14678	0.258959	0.998356
Gnai3	14679	0.179058	0.998356
Gnal	14680	0.251742	0.998356
Gnaq	14682	0.239352	0.998356
Gnas	14683	-0.12457	0.998356
Gnb1	14688	-0.02814	0.998356
Gnb2	14693	0.211787	0.998356
Gnb2l1	14694	0.084796	0.998356
Gnb5	14697	-0.40297	0.998356
Gng10	14700	0.292469	0.998356
Gng12	14701	-0.12535	0.998356
Bscl2	14705	-0.09348	0.998356
Gngt2	14710	0.204073	0.998356
Gnpat	14712	0.01619	0.998356
Got1	14718	0.150755	0.998356
Got2	14719	-0.22253	0.998356
Pdpn	14726	-0.77882	0.998356
Gp49a	14727	-0.20611	0.998356
Lilrb4	14728	-0.01924	0.998356
Gpaa1	14731	0.024893	0.998356
Gpam	14732	0.161981	0.998356
Gpc1	14733	0.352283	0.998356
S1pr2	14739	-0.267	0.998356
Lpar1	14745	0.332186	0.998356
Gpi1	14751	-0.05883	0.998356
Pigq	14755	0.080788	0.998356
Gpld1	14756	0.136021	0.998356
Gpm6b	14758	0.086471	0.998356
Gpr56	14766	0.052991	0.998356
Lancl1	14768	0.178857	0.998356
Grk5	14773	-0.14318	0.998356
Gpx1	14775	0.213043	0.998356
Gpx2	14776	-0.44043	0.998356
Gpx2-ps1	14777	-0.41356	0.998356
Gpx3	14778	0.673599	0.998356
Gsr	14782	-0.067	0.998356
Grb10	14783	0.809902	0.998356
Grb2	14784	0.180954	0.998356
Grb7	14786	-0.22073	0.998356
Grcc10	14790	0.15941	0.998356
Emg1	14791	-0.38659	0.998356
Lpcat3	14792	-0.11238	0.998356
Cdca3	14793	0.03869	0.998356
Spsb2	14794	0.338778	0.998356

Aes	14797	0.058515	0.998356
Nr3c1	14815	0.360727	0.998356
Grn	14824	0.156006	0.998356
Pdia3	14827	-0.38154	0.998356
Hspa5	14828	0.268948	0.998356
Gsg2	14841	-0.30242	0.998356
Gspt1	14852	-0.30384	0.998356
Gss	14854	0.133477	0.998356
Gsta3	14859	0.666477	0.998356
Gsta4	14860	-0.30941	0.998356
Gstm1	14862	-0.05146	0.998356
Gstm2	14863	0.010296	0.998356
Gstm3	14864	-1.01385	0.998356
Gstm4	14865	-0.75712	0.998356
Gstm5	14866	0.0705	0.998356
Gstm6	14867	-0.40487	0.998356
Gstp2	14869	-0.73738	0.998356
Gstp1	14870	0.40126	0.998356
Gstt1	14871	0.5403	0.998356
Gstt2	14872	-0.28895	0.998356
Gsto1	14873	-0.14412	0.998356
Gstz1	14874	1.22085	0.998356
Gtf2h1	14884	-0.18667	0.998356
Gtf2h4	14885	0.002338	0.998356
Gtf2i	14886	-0.61074	0.998356
Gtl3	14894	-0.15655	0.998356
Trip12	14897	0.043671	0.998356
Gtpbp1	14904	-0.10911	0.998356
Gt(ROSA)26Sor	14910	-0.90579	0.998356
Thumpd3	14911	-0.01196	0.998356
Guca2a	14915	0.518359	0.998356
Guca2b	14916	0.269406	0.998356
Gucy2c	14917	-0.19959	0.998356
Guk1	14923	-1.85212	0.998356
Magi1	14924	0.312416	0.998356
Gyk	14933	-0.18835	0.998356
Gys1	14936	-0.10364	0.998356
Gzmb	14939	-0.93726	0.998356
H13	14950	-0.56533	0.998356
Hist1h1d	14957	-0.36237	0.998356
H1f0	14958	-0.0271	0.998356
H2-Aa	14960	-0.2599	0.998356
H2-Ab1	14961	-0.05275	0.998356
Cfb	14962	0.087174	0.998356
H2-D1	14964	0.17888	0.998356
H2-Eb1	14969	-0.65641	0.998356
H2-K1	14972	0.03043	0.998356

H2-Ke2	14976	-10	0.998356
Slc39a7	14977	-0.25217	0.998356
H2-Ke6	14979	-0.21584	0.998356
H2-M3	14991	0.443158	0.998356
H2-DMa	14998	-1.03978	0.998356
H2-Q10	15007	0.038255	0.998356
H2-Q2	15013	0.367953	0.998356
H2-Q4	15015	0.239446	0.998356
H2-Q7	15018	-0.4214	0.998356
H2-Q8	15019	-0.90661	0.998356
H2-T22	15039	0.053726	0.998356
H2-T23	15040	0.279016	0.998356
H2-T24	15042	0.081066	0.998356
H2-T9	15051	-0.37227	0.998356
Mr1	15064	0.087494	0.998356
H3f3a	15078	-0.01562	0.998356
H3f3b	15081	-0.32252	0.998356
Hadh	15107	0.180116	0.998356
Hsd17b10	15108	-0.05059	0.998356
Hap1	15114	-0.64723	0.998356
Hars	15115	-0.16501	0.998356
Hbb-b1	15129	-3.527	0.998356
Hccs	15159	0.286762	0.998356
Hcfc1	15161	0.019228	0.998356
Hck	15162	0.12167	0.998356
Hcls1	15163	-0.31429	0.998356
Ptpn6	15170	-0.39958	0.998356
Hdac2	15182	-0.0188	0.998356
Hdac3	15183	0.084039	0.998356
Hdac5	15184	-0.61629	0.998356
Hdac6	15185	-0.14139	0.998356
Hdc	15186	-0.28977	0.998356
Hdgf	15191	-0.11594	0.998356
Hdgfrp2	15193	-0.20976	0.998356
Htt	15194	-0.03024	0.998356
Hbegf	15200	0.234492	0.998356
Hells	15201	-0.40704	0.998356
Heph	15203	0.543057	0.998356
Herc2	15204	0.24292	0.998356
Hes1	15205	-0.23734	0.998356
Hexa	15211	-0.18502	0.998356
Hexb	15212	0.865382	0.998356
Foxq1	15220	-0.04019	0.998356
Foxf1a	15227	0.413658	0.998356
Hgf	15234	0.061088	0.998356
Hgs	15239	0.641439	0.998356
Hiat1	15247	-0.1239	0.998356

Hif1a	15251	0.093227	0.998356
Hipk1	15257	0.016725	0.998356
Hipk2	15258	-0.09472	0.998356
Hipk3	15259	0.113036	0.998356
Hira	15260	0.198189	0.998356
Hist2h2aa1	15267	0.447993	0.998356
Hivep2	15273	-0.09219	0.998356
Hk1	15275	-0.00668	0.998356
Hk2	15277	0.31701	0.998356
Tfb2m	15278	0.093781	0.998356
Hmbs	15288	0.014971	0.998356
Hmgb1	15289	-0.43541	0.998356
Hmgn1	15312	0.022517	0.998356
Hmgn2	15331	-0.0603	0.998356
Hmg20b	15353	-0.27293	0.998356
Hmgb3	15354	-0.27901	0.998356
Hmgcl	15356	-0.14866	0.998356
Hmgcr	15357	0.069579	0.998356
Hmgcs2	15360	0.024483	0.998356
Hmga1	15361	-1.00288	0.998356
Hmga2	15364	0.003512	0.998356
Hmmr	15366	-0.20508	0.998356
Hmox1	15368	-0.44335	0.998356
Hmox2	15369	-0.16509	0.998356
Nr4a1	15370	-0.00982	0.998356
Hn1	15374	-0.05287	0.998356
Foxa1	15375	0.370349	0.998356
Foxa2	15376	0.159879	0.998356
Foxa3	15377	-0.1014	0.998356
Hnf4a	15378	0.093075	0.998356
Hnrnpc	15381	-0.48831	0.998356
Hnrnpa1	15382	-0.67224	0.998356
Hnrnpab	15384	-0.06735	0.998356
Hnrnpk	15387	-0.29557	0.998356
Hnrnpl	15388	0.165052	0.998356
Hoxa10	15395	0.101215	0.998356
Hoxa11	15396	-0.28921	0.998356
Hoxa11as	15397	-0.12157	0.998356
Hoxa13	15398	0.634798	0.998356
Hoxa5	15402	0.192622	0.998356
Hoxa6	15403	-0.64048	0.998356
Hoxa7	15404	0.016433	0.998356
Hoxa9	15405	-0.23401	0.998356
Hoxb13	15408	0.070568	0.998356
Hoxb9	15417	-0.08628	0.998356
Hoxd10	15430	0.107776	0.998356
Hoxd11	15431	-0.27223	0.998356

Hoxd13	15433	-0.21574	0.998356
Hp	15439	-0.39975	0.998356
Hp1bp3	15441	-1.11813	0.998356
Hpse	15442	0.533302	0.998356
Hpd	15445	0.241888	0.998356
Hpgd	15446	-0.18975	0.998356
Hprt	15452	0.167215	0.998356
Hr	15460	-0.07375	0.998356
Hras1	15461	0.70569	0.998356
Agfg1	15463	0.291114	0.998356
Eif2ak1	15467	0.19684	0.998356
Prmt2	15468	0.022202	0.998356
Prmt1	15469	-0.30109	0.998356
Hrsp12	15473	-0.48429	0.998356
Hs3st1	15476	-0.15919	0.998356
Hspa8	15481	-0.4996	0.998356
Hsd17b2	15486	0.231313	0.998356
Hsd17b4	15488	0.167998	0.998356
Hsd17b7	15490	0.138045	0.998356
Hsf1	15499	-0.3276	0.998356
Hsf2	15500	-0.27249	0.998356
Dnaja1	15502	-0.23466	0.998356
Hsph1	15505	-0.45883	0.998356
Hspb1	15507	-0.1823	0.998356
Hspd1	15510	-0.68358	0.998356
Hspa1b	15511	0.042455	0.998356
Hspa2	15512	-0.04683	0.998356
Hsp90ab1	15516	-0.09141	0.998356
Hsp90aa1	15519	-0.30935	0.998356
Hspa4	15525	-0.06802	0.998356
Hspa9	15526	-0.04945	0.998356
Hspe1	15528	-0.04916	0.998356
Sdc2	15529	-0.29356	0.998356
Hspg2	15530	0.353909	0.998356
Ndst1	15531	0.19904	0.998356
Trmt2a	15547	0.461158	0.998356
Elavl1	15568	-0.07171	0.998356
Hus1	15574	-0.42034	0.998356
Hyal1	15586	-0.03753	0.998356
Hyal2	15587	-0.25106	0.998356
Ica1	15893	-0.18833	0.998356
Icam1	15894	-0.38152	0.998356
Irf8	15900	0.019256	0.998356
Id1	15901	-0.16164	0.998356
Id2	15902	-0.03397	0.998356
Id3	15903	0.156824	0.998356
Ide	15925	-0.30976	0.998356

Idh1	15926	0.130549	0.998356
Idh3g	15929	-0.03686	0.998356
Ido1	15930	-0.35508	0.998356
Ids	15931	-0.04846	0.998356
Idua	15932	-0.05955	0.998356
Ier2	15936	-0.28458	0.998356
Ier5	15939	0.557797	0.998356
Irgm1	15944	0.083289	0.998356
Ifi47	15953	-0.1065	0.998356
Ifit2	15958	0.446813	0.998356
Ifnar1	15975	0.143331	0.998356
Ifnar2	15976	-0.34949	0.998356
Ifngr1	15979	0.369172	0.998356
Ifngr2	15980	-0.0282	0.998356
Ifrd2	15983	-0.05579	0.998356
Igf1	16000	-0.10385	0.998356
Igf1r	16001	0.309342	0.998356
Igf2r	16004	0.012533	0.998356
Cyr61	16007	-0.16758	0.998356
Igfbp4	16010	-0.19241	0.998356
Igfbp5	16011	-0.46544	0.998356
Il18bp	16068	0.052014	0.998356
Igtp	16145	0.150585	0.998356
Ihh	16147	-0.23601	0.998356
Cd74	16149	-0.94807	0.998356
Ikbkb	16150	0.094318	0.998356
Ikbkg	16151	0.212771	0.998356
Il10rb	16155	0.415056	0.998356
Il11ra1	16157	0.388811	0.998356
Il13ra1	16164	0.211217	0.998356
Il17ra	16172	0.110799	0.998356
Il18	16173	0.308213	0.998356
Il1b	16176	-0.08407	0.998356
Il1r1	16177	0.559212	0.998356
Il1r2	16178	0.572834	0.998356
Irak1	16179	0.353398	0.998356
Il1rap	16180	-0.10115	0.998356
Il1rn	16181	-1.00901	0.998356
Il2rg	16186	0.067516	0.998356
Il4ra	16190	0.162383	0.998356
Il6	16193	-3.47178	0.998356
Il6st	16195	0.149203	0.998356
Ilf3	16201	-0.99772	0.998356
Ilk	16202	0.046517	0.998356
Gimap1	16205	0.528667	0.998356
Lrig1	16206	-0.07406	0.998356
Impact	16210	-0.07771	0.998356

Kpnb1	16211	-0.10056	0.998356
Incenp	16319	-0.13708	0.998356
Inhba	16323	-0.62695	0.998356
Inhbb	16324	-0.00688	0.998356
Cep250	16328	-3.36966	0.998356
Inpp1	16329	-0.17364	0.998356
Inpp5b	16330	-0.11526	0.998356
Inppl1	16332	0.064853	0.998356
Insl3	16336	-0.83076	0.998356
Insr	16337	-0.00982	0.998356
Eif3e	16341	0.305483	0.998356
Invs	16348	-0.30919	0.998356
Ipp	16351	-0.28642	0.998356
Irf1	16362	0.294279	0.998356
Irf2	16363	-0.03741	0.998356
Irg1	16365	-0.20066	0.998356
Irs1	16367	0.200537	0.998356
Irf9	16391	-3.31406	0.998356
Itch	16396	0.537194	0.998356
Itga2	16398	0.321984	0.998356
Itga3	16400	0.405147	0.998356
Itga5	16402	-0.75691	0.998356
Itga6	16403	0.242069	0.998356
Itgam	16409	0.359698	0.998356
Itgav	16410	0.57859	0.998356
Itgax	16411	0.18252	0.998356
Itgb1	16412	0.167552	0.998356
Itgb1bp1	16413	0.734079	0.998356
Itgb2	16414	-0.12251	0.998356
Itgb3	16416	0.042097	0.998356
Eif6	16418	0.181509	0.998356
Itgb5	16419	0.620507	0.998356
Cd47	16423	-0.17529	0.998356
Itln1	16429	0.231981	0.998356
Stt3a	16430	-0.42698	0.998356
Itm2b	16432	0.143604	0.998356
Itpa	16434	0.591479	0.998356
Itpr1	16438	0.036361	0.998356
Itpr2	16439	0.155072	0.998356
Itpr3	16440	0.383781	0.998356
Itsn1	16443	-0.25669	0.998356
Jag1	16449	-0.16909	0.998356
Jag2	16450	0.032754	0.998356
Jak1	16451	0.050423	0.998356
Jak2	16452	0.588805	0.998356
Jak3	16453	-0.83518	0.998356
F11r	16456	0.545247	0.998356

Jarid2	16468	-0.11159	0.998356
Jub	16475	-0.29987	0.998356
Jun	16476	-0.2304	0.998356
Junb	16477	0.050248	0.998356
Jund	16478	0.448663	0.998356
Jup	16480	0.20707	0.998356
Kcnj2	16518	0.125474	0.998356
Kcnk1	16525	0.009518	0.998356
Kcnk5	16529	-0.22427	0.998356
Kcnn4	16534	0.038657	0.998356
Kcnq1	16535	-0.32783	0.998356
Khsrp	16549	-0.2038	0.998356
Kif11	16551	-0.12665	0.998356
Kif12	16552	-0.33323	0.998356
Kif13a	16553	0.25252	0.998356
Kif13b	16554	0.31956	0.998356
Kif16b	16558	0.295157	0.998356
Kif1a	16560	0.173406	0.998356
Kif1b	16561	0.073966	0.998356
Kif1c	16562	0.311233	0.998356
Kif2a	16563	0.054423	0.998356
Kif21a	16564	-0.00935	0.998356
Kif21b	16565	-0.28584	0.998356
Kif3a	16568	0.048061	0.998356
Kif3b	16569	0.134467	0.998356
Kif3c	16570	0.162566	0.998356
Kif4	16571	0.181682	0.998356
Kif5b	16573	-0.09402	0.998356
Kifap3	16579	0.34523	0.998356
Kifc5b	16580	-0.26506	0.998356
Kin	16588	0.143794	0.998356
Uhmk1	16589	0.256971	0.998356
Kit	16590	-0.27514	0.998356
Klc1	16593	-0.4883	0.998356
Klc2	16594	0.280311	0.998356
Klf12	16597	0.017912	0.998356
Klf2	16598	0.318181	0.998356
Klf3	16599	0.016896	0.998356
Klf9	16601	0.100529	0.998356
Kpna1	16646	-0.0095	0.998356
Kpna2	16647	-0.38387	0.998356
Kpna3	16648	-0.16823	0.998356
Kpna4	16649	0.044192	0.998356
Kpna6	16650	-0.0024	0.998356
Kras	16653	-0.01943	0.998356
Mafb	16658	0.270778	0.998356
Krt13	16663	-0.44476	0.998356

Krt15	16665	-0.15556	0.998356
Krt18	16668	-0.5417	0.998356
Krt19	16669	-0.14606	0.998356
Krt36	16673	0.293003	0.998356
Krt4	16682	-0.42278	0.998356
Krt8	16691	0.423465	0.998356
Ksr1	16706	0.452327	0.998356
Ktn1	16709	0.002585	0.998356
L1cam	16728	0.302278	0.998356
Lad1	16763	-0.24433	0.998356
Stmn1	16765	-0.09786	0.998356
Lama3	16774	0.616533	0.998356
Lama4	16775	0.398381	0.998356
Lama5	16776	0.332027	0.998356
Lamb1	16777	0.397782	0.998356
Lamb2	16779	0.603988	0.998356
Lamb3	16780	0.608136	0.998356
Lamc2	16782	0.719622	0.998356
Lamp1	16783	0.379538	0.998356
Lamp2	16784	0.431904	0.998356
Rpsa	16785	-0.06129	0.998356
Anpep	16790	0.204693	0.998356
Laptm5	16792	-0.24028	0.998356
Large	16795	-0.06758	0.998356
Lasp1	16796	0.239897	0.998356
Lats1	16798	0.050088	0.998356
Arhgef2	16800	1.45005	0.998356
Arhgef1	16801	-0.1761	0.998356
Lbp	16803	-0.6386	0.998356
Lcn2	16819	-0.79743	0.998356
Ldb1	16825	-0.27643	0.998356
Ldha	16828	-0.0677	0.998356
Ldhb	16832	0.421581	0.998356
Cog1	16834	0.076032	0.998356
Ldlr	16835	-0.07988	0.998356
Lef1	16842	-0.21563	0.998356
Lfng	16848	0.009114	0.998356
Lgals1	16852	-0.97708	0.998356
Lgals3	16854	0.351511	0.998356
Lgals4	16855	0.415291	0.998356
Lgals9	16859	0.216347	0.998356
Eif2d	16865	-0.37044	0.998356
Lif	16878	-0.82006	0.998356
Lifr	16880	0.22289	0.998356
Lig1	16881	-0.45258	0.998356
Lig3	16882	-0.22189	0.998356
Limk1	16885	0.025529	0.998356

Limk2	16886	-0.30702	0.998356
Lipa	16889	0.35525	0.998356
Llgl1	16897	0.266049	0.998356
Rps2	16898	0.341645	0.998356
Lmna	16905	0.356676	0.998356
Lmnb1	16906	-0.19498	0.998356
Lmnb2	16907	-0.05834	0.998356
Lmo2	16909	0.74523	0.998356
Lmo4	16911	0.7686	0.998356
Psmb9	16912	-0.44457	0.998356
Psmb8	16913	-0.52806	0.998356
Mycl1	16918	0.10956	0.998356
Phyh	16922	-0.01767	0.998356
Sh2b3	16923	0.199107	0.998356
Lnx1	16924	-0.15655	0.998356
Loxl1	16949	0.733746	0.998356
Anxa1	16952	-0.37244	0.998356
Zbtb7a	16969	0.068175	0.998356
Lrp1	16971	0.225841	0.998356
Lrp5	16973	0.259723	0.998356
Lrp6	16974	0.012586	0.998356
Lrp8	16975	-0.2543	0.998356
Lrpap1	16976	0.186862	0.998356
Lrrfip1	16978	0.112737	0.998356
Lrrn3	16981	-0.09425	0.998356
Lsp1	16985	-0.96017	0.998356
Lss	16987	0.161505	0.998356
Lst1	16988	-0.79957	0.998356
Lta4h	16993	-0.19527	0.998356
Ltb	16994	-0.5607	0.998356
Ltbp3	16998	0.179731	0.998356
Ltbr	17000	0.468786	0.998356
Lxn	17035	0.736479	0.998356
Blnk	17060	0.301822	0.998356
Muc13	17063	0.454752	0.998356
Cd93	17064	0.053033	0.998356
Ly6c1	17067	-0.42187	0.998356
Ly6e	17069	0.144528	0.998356
Epcam	17075	0.073216	0.998356
Ly75	17076	-0.07433	0.998356
Il1rl1	17082	-0.87917	0.998356
Tmed1	17083	0.42689	0.998356
Ly86	17084	0.299968	0.998356
Ly96	17087	-0.17631	0.998356
Lyar	17089	-0.33957	0.998356
Lyn	17096	-0.3732	0.998356
Lyst	17101	0.144567	0.998356

Lyz2	17105	0.129821	0.998356
Lyz1	17110	0.594935	0.998356
Tm4sf1	17112	-0.01738	0.998356
M6pr	17113	-0.17454	0.998356
Amacr	17117	-0.23551	0.998356
Marcks	17118	0.017594	0.998356
Mad1l1	17120	-0.11322	0.998356
Mxd3	17121	0.238801	0.998356
Mxd4	17122	-0.0257	0.998356
Smad1	17125	-0.07261	0.998356
Smad2	17126	-0.18121	0.998356
Smad3	17127	-0.04896	0.998356
Smad4	17128	0.273568	0.998356
Smad5	17129	-1.75159	0.998356
Smad6	17130	0.126593	0.998356
Maff	17133	0.281893	0.998356
Mafg	17134	0.044105	0.998356
Mafk	17135	0.316577	0.998356
Magoh	17149	-0.34053	0.998356
Ccndbp1	17151	0.424857	0.998356
Mal	17153	0.200621	0.998356
Man1a	17155	0.181484	0.998356
Man1a2	17156	0.017434	0.998356
Man2a1	17158	0.372635	0.998356
Man2b1	17159	-0.07283	0.998356
Man2b2	17160	0.295854	0.998356
Maoa	17161	0.067457	0.998356
Mapkapk2	17164	0.246122	0.998356
Mapkapk5	17165	0.598831	0.998356
Nprl3	17168	0.595418	0.998356
Mark3	17169	1.25578	0.998356
Ascl2	17173	-0.17225	0.998356
Matr3	17184	-0.17326	0.998356
Max	17187	0.047433	0.998356
Maz	17188	0.273644	0.998356
Mbd1	17190	-0.21081	0.998356
Mbd2	17191	0.485356	0.998356
Mbd3	17192	0.121647	0.998356
Mbd4	17193	-0.65628	0.998356
Mbp	17196	0.217157	0.998356
Mcf2l	17207	0.459305	0.998356
Mcl1	17210	0.182855	0.998356
Mcm3	17215	-0.27394	0.998356
Mcm2	17216	-0.33207	0.998356
Mcm4	17217	-0.17207	0.998356
Mcm5	17218	-0.21324	0.998356
Mcm6	17219	-0.1435	0.998356

Mcm7	17220	0.203183	0.998356
Anapc1	17222	-0.00935	0.998356
Mcpt4	17227	-1.0527	0.998356
Cma1	17228	-0.27515	0.998356
Tpsb2	17229	-0.77788	0.998356
Mgrn1	17237	0.415284	0.998356
Mdfi	17240	0.495485	0.998356
Mdk	17242	-0.41361	0.998356
Mdm2	17246	0.011958	0.998356
Mdm4	17248	-0.08526	0.998356
Abcc1	17250	0.248616	0.998356
Rdh11	17252	0.233734	0.998356
Slc3a2	17254	-0.26659	0.998356
Mea1	17256	0.094393	0.998356
Mecp2	17257	0.416867	0.998356
Mef2a	17258	-0.1397	0.998356
Mef2d	17261	0.34995	0.998356
Rab8a	17274	0.112059	0.998356
Melk	17279	0.066406	0.998356
Fyco1	17281	0.198089	0.998356
Men1	17283	0.698596	0.998356
Mep1a	17287	0.606903	0.998356
Mep1b	17288	0.599088	0.998356
Met	17295	0.137539	0.998356
Mettl1	17299	-0.35504	0.998356
Mfge8	17304	0.163873	0.998356
Mgat1	17308	-0.2185	0.998356
Mgat3	17309	0.193425	0.998356
Mgp	17313	0.551198	0.998356
Mid1	17318	0.502284	0.998356
Mif	17319	0.267251	0.998356
Minpp1	17330	0.056978	0.998356
Pias2	17344	0.301096	0.998356
Mki67	17345	-0.20792	0.998356
Mknk1	17346	-0.04795	0.998356
Mknk2	17347	-0.06429	0.998356
Mlh1	17350	0.003198	0.998356
Mllt10	17354	-1.62374	0.998356
Aff1	17355	0.142035	0.998356
Mllt4	17356	0.131334	0.998356
Marcksl1	17357	0.500199	0.998356
Mmp12	17381	-0.41994	0.998356
Mmp10	17384	-0.36748	0.998356
Mmp11	17385	0.277837	0.998356
Mmp14	17387	-0.1144	0.998356
Mmp15	17388	0.335335	0.998356
Mmp2	17390	0.430104	0.998356

Mmp7	17393	0.128867	0.998356
Mmp9	17395	0.250335	0.998356
Mnat1	17420	0.437608	0.998356
Ndst2	17423	0.051059	0.998356
Foxk1	17425	-0.12813	0.998356
Mns1	17427	-0.25334	0.998356
Mnt	17428	-0.07749	0.998356
Mocs2	17434	0.082755	0.998356
Me1	17436	0.498836	0.998356
Mdh2	17448	0.074453	0.998356
Mdh1	17449	-0.26521	0.998356
Mov10	17454	0.116959	0.998356
Psmd7	17463	-0.11613	0.998356
Cd200	17470	0.296322	0.998356
Gbp4	17472	-2.14391	0.998356
Clec4d	17474	0.729575	0.998356
Mpeg1	17476	0.272038	0.998356
Mpp1	17524	0.369375	0.998356
Mpv17	17527	0.401461	0.998356
Mrc1	17533	0.389172	0.998356
Mre11a	17535	-0.44278	0.998356
Cited2	17684	-0.2783	0.998356
Msh2	17685	-0.0313	0.998356
Msh3	17686	0.060274	0.998356
Msh6	17688	-0.0575	0.998356
Msi1	17690	0.185553	0.998356
Sik1	17691	0.122473	0.998356
Msl3	17692	-0.21075	0.998356
Msn	17698	-0.16464	0.998356
Msx1	17701	0.168292	0.998356
Grpel1	17713	-0.30891	0.998356
Grpel2	17714	0.126208	0.998356
Mt1	17748	0.246616	0.998356
Polr2k	17749	-0.6158	0.998356
Mt3	17751	0.682921	0.998356
Mtap4	17758	-0.00399	0.998356
Mtap6	17760	-0.21594	0.998356
Mtap7	17761	-0.17735	0.998356
Mtcp1	17763	-0.38103	0.998356
Mtf1	17764	0.069698	0.998356
Mtf2	17765	0.308288	0.998356
Nudt1	17766	-0.05849	0.998356
Mthfd2	17768	-0.08195	0.998356
Mtm1	17772	0.593235	0.998356
Laptm4a	17775	0.200806	0.998356
Mast2	17776	-0.00224	0.998356
Fam89b	17826	0.653124	0.998356

Mtx1	17827	-0.09821	0.998356
Muted	17828	-0.07502	0.998356
Muc1	17829	0.098753	0.998356
Commd1	17846	0.454166	0.998356
Usp34	17847	0.188474	0.998356
Mut	17850	0.120754	0.998356
Mvk	17855	0.212216	0.998356
Mxi1	17859	0.590689	0.998356
Myb	17863	-0.28011	0.998356
Mybl2	17865	-0.12936	0.998356
Myc	17869	-0.64091	0.998356
Ppp1r15a	17872	-0.20666	0.998356
Gadd45b	17873	0.308953	0.998356
Myd88	17874	0.132291	0.998356
Myef2	17876	-1.21431	0.998356
Myh9	17886	0.072493	0.998356
Myl7	17898	0.163637	0.998356
Myl6	17904	0.569591	0.998356
Myo10	17909	-0.13049	0.998356
Myo1b	17912	-0.40199	0.998356
Myo1c	17913	0.176029	0.998356
Myo5b	17919	0.182942	0.998356
Myo6	17920	0.332738	0.998356
Myo7a	17921	0.116575	0.998356
Myo7b	17922	-0.16675	0.998356
Myo9b	17925	0.134073	0.998356
Ppp1r12a	17931	-0.17226	0.998356
Nab1	17936	0.194795	0.998356
Naca	17938	0.046826	0.998356
Naga	17939	0.33147	0.998356
Naip6	17952	0.326755	0.998356
Nap1l4	17955	-0.00457	0.998356
Nbr1	17966	0.043805	0.998356
Ncf1	17969	-0.08153	0.998356
Ncf2	17970	-0.36764	0.998356
Nck1	17973	-0.02516	0.998356
Nck2	17974	0.106889	0.998356
Ncl	17975	-0.68678	0.998356
Ncoa1	17977	0.238762	0.998356
Ncoa2	17978	0.473202	0.998356
Ncoa3	17979	0.133087	0.998356
Ndrg1	17988	0.055126	0.998356
Ndufv1	17995	0.068062	0.998356
Nedd1	17997	-0.16282	0.998356
Nedd4	17999	-0.15155	0.998356
Nedd8	18002	-0.01894	0.998356
Nedd9	18003	-0.04782	0.998356

Nek1	18004	0.111108	0.998356
Nek2	18005	-0.18889	0.998356
Neo1	18007	0.221818	0.998356
Neu1	18010	-0.09062	0.998356
Neurl1a	18011	1.06092	0.998356
Nf1	18015	0.141285	0.998356
Nf2	18016	10	0.998356
Nfatc1	18018	-0.49459	0.998356
Nfatc3	18021	-0.1161	0.998356
Nfe2l1	18023	0.480415	0.998356
Nfe2l2	18024	0.100849	0.998356
Nfe2l3	18025	-0.07092	0.998356
Nfia	18027	-0.22598	0.998356
Nfib	18028	0.21933	0.998356
Nfic	18029	1.28482	0.998356
Nfil3	18030	-0.13963	0.998356
Nfix	18032	-0.66152	0.998356
Nfkb1	18033	0.332306	0.998356
Nfkb2	18034	0.217398	0.998356
Nfkbia	18035	-0.29211	0.998356
Nfkbib	18036	-0.08392	0.998356
Nfkbie	18037	0.285125	0.998356
Nfkbil1	18038	0.15349	0.998356
Nfs1	18041	0.365888	0.998356
Nfya	18044	-0.34267	0.998356
Nfyb	18045	-0.01965	0.998356
Nfyc	18046	-0.06919	0.998356
Nid1	18073	0.567237	0.998356
Nin	18080	0.225775	0.998356
Ninj1	18081	-0.1883	0.998356
Nipsnap1	18082	-0.15834	0.998356
Nktr	18087	-0.14167	0.998356
Nlk	18099	0.220862	0.998356
Mrpl40	18100	-0.14904	0.998356
Nme1	18102	-0.26163	0.998356
Nqo1	18104	0.642354	0.998356
Nqo2	18105	-0.49662	0.998356
Cd244	18106	0.440494	0.998356
Nmt1	18107	0.21519	0.998356
Nmt2	18108	-0.04651	0.998356
Mycn	18109	0.245702	0.998356
Rrp1	18114	0.115367	0.998356
Cox4nb	18117	0.22079	0.998356
Mrpl49	18120	-0.12599	0.998356
Nos2	18126	-0.24242	0.998356
Notch1	18128	0.077551	0.998356
Notch2	18129	-0.00759	0.998356

Ints6	18130	0.018157	0.998356
Notch4	18132	-0.06089	0.998356
Nov	18133	-0.93373	0.998356
Zfml	18139	-0.65504	0.998356
Uhrf1	18140	2.73757	0.998356
Nup50	18141	-0.14211	0.998356
Npas2	18143	-0.3773	0.998356
Npc1	18145	0.39627	0.998356
Npdc1	18146	-0.3759	0.998356
Npm1	18148	-0.43266	0.998356
Npm3	18150	0.008513	0.998356
Nptx1	18164	0.321054	0.998356
Nr1i2	18171	0.37294	0.998356
Slc11a1	18173	-0.01035	0.998356
Slc11a2	18174	0.053881	0.998356
Nras	18176	0.165298	0.998356
Nrf1	18181	-0.87212	0.998356
Nrp1	18186	-0.03969	0.998356
Nrp2	18187	-0.23055	0.998356
Nrxn1	18189	0.110541	0.998356
Nsd1	18193	-0.04364	0.998356
Nsdhl	18194	0.243015	0.998356
Nsf	18195	0.322076	0.998356
Nsmaf	18201	0.020365	0.998356
Ntan1	18203	-0.04206	0.998356
Nthl1	18207	-0.00561	0.998356
Nucb1	18220	0.245712	0.998356
Numb	18222	-0.1072	0.998356
Nup62	18226	-0.35138	0.998356
Nr4a2	18227	-1.66133	0.998356
Nxn	18230	0.078042	0.998356
Oaz1	18245	0.965707	0.998356
Oaz2	18247	0.255958	0.998356
Ocln	18260	0.222724	0.998356
Odc1	18263	0.11954	0.998356
Odf2	18286	-0.22495	0.998356
Ogdh	18293	-0.58221	0.998356
Ogg1	18294	-0.24639	0.998356
Ogn	18295	-1.18478	0.998356
Oit1	18300	0.243133	0.998356
Fxyd5	18301	-0.63278	0.998356
Omp	18378	0.216661	0.998356
Tnfrsf11b	18383	-0.18261	0.998356
Sigmar1	18391	0.191448	0.998356
Orc2	18393	-0.18228	0.998356
Slc22a18	18400	-0.45907	0.998356
Slc25a15	18408	0.322563	0.998356

Sqstm1	18412	0.472394	0.998356
Osm	18413	-0.8269	0.998356
Osmr	18414	0.247147	0.998356
Hspa4l	18415	0.100509	0.998356
Ovol1	18426	-0.00874	0.998356
Mybbp1a	18432	-0.28797	0.998356
P2rx4	18438	0.352177	0.998356
P2rx7	18439	-1.16961	0.998356
P2ry1	18441	-0.0472	0.998356
P2ry2	18442	-0.18219	0.998356
P4ha1	18451	-0.09549	0.998356
P4ha2	18452	-1.92236	0.998356
P4hb	18453	0.352464	0.998356
Pldn	18457	-0.0432	0.998356
Pabpc1	18458	0.209158	0.998356
Pafah1b1	18472	0.180546	0.998356
Pafah1b2	18475	-0.05316	0.998356
Prdx1	18477	-0.29437	0.998356
Pak1	18479	-0.00927	0.998356
Pax8	18510	-0.30572	0.998356
Pbx1	18514	0.072781	0.998356
Pbx2	18515	-0.17105	0.998356
Pbx3	18516	0.740949	0.998356
Igbp1	18518	-0.10795	0.998356
Kat2b	18519	0.112389	0.998356
Pcbp2	18521	0.378432	0.998356
Pcm1	18536	-0.13208	0.998356
Pcmt1	18537	-0.12661	0.998356
Pcna	18538	0.372022	0.998356
Pcnt	18541	-0.03261	0.998356
Pcolce	18542	0.476573	0.998356
Furin	18550	0.156005	0.998356
Pcsk6	18553	0.579402	0.998356
Pcsk7	18554	0.144023	0.998356
Cdk16	18555	0.192859	0.998356
Cdk18	18557	-0.18371	0.998356
Pctp	18559	-0.19705	0.998356
Pcx	18563	0.607391	0.998356
Pdcd2	18567	0.612675	0.998356
Pdcd4	18569	0.034028	0.998356
Pdcd6	18570	0.473635	0.998356
Pdcd6ip	18571	1.07628	0.998356
Pdcd11	18572	-0.19455	0.998356
Pde3b	18576	-0.11133	0.998356
Pde4b	18578	0.130807	0.998356
Pde6d	18582	-0.08391	0.998356
Pde7a	18583	-0.41709	0.998356

Pde9a	18585	0.201767	0.998356
Pdgfb	18591	-0.3024	0.998356
Pdgfra	18595	0.639997	0.998356
Pdgfrb	18596	0.526349	0.998356
Pdha1	18597	-0.26202	0.998356
Padi4	18602	0.064436	0.998356
Pdk2	18604	0.512982	0.998356
Enpp1	18605	0.298817	0.998356
Pdpk1	18607	-0.12347	0.998356
Pea15a	18611	0.049572	0.998356
Etv4	18612	-0.12233	0.998356
Pecam1	18613	-0.02668	0.998356
Pepd	18624	-0.12888	0.998356
Per1	18626	0.624111	0.998356
Per2	18627	-0.0686	0.998356
Pex11b	18632	0.350274	0.998356
Pex16	18633	0.149592	0.998356
Pex7	18634	0.294909	0.998356
Cfp	18636	0.0562	0.998356
Pfdn2	18637	-0.34961	0.998356
Pfkfb2	18640	-0.10913	0.998356
Pfkl	18641	0.052289	0.998356
Pfn1	18643	0.075038	0.998356
Pfn2	18645	-0.30966	0.998356
Pgam1	18648	0.097042	0.998356
Pgk1	18655	-0.02507	0.998356
Abcb1a	18671	0.582118	0.998356
Phb	18673	-0.05211	0.998356
Slc25a3	18674	0.212615	0.998356
Phf2	18676	-0.08224	0.998356
Phka1	18679	0.753262	0.998356
Phtf1	18685	-0.8657	0.998356
Pick1	18693	-0.08095	0.998356
Piga	18700	-0.01845	0.998356
Pigf	18701	0.481531	0.998356
Pigr	18703	0.088254	0.998356
Pik3c2a	18704	-0.12122	0.998356
Pik3ca	18706	0.281511	0.998356
Pik3r1	18708	0.034894	0.998356
Pik3r2	18709	0.34026	0.998356
Pik3r3	18710	-0.5352	0.998356
Pikfyve	18711	-0.13684	0.998356
Pim1	18712	0.395567	0.998356
Pip5k1c	18717	0.058762	0.998356
Pip5k1b	18719	0.206116	0.998356
Pip5k1a	18720	0.217627	0.998356
Lilrb3	18733	-0.42354	0.998356

Pitpna	18738	0.290231	0.998356
Pitpnm1	18739	0.688701	0.998356
Pitx2	18741	-0.91347	0.998356
Pja1	18744	-0.19536	0.998356
Pkm2	18746	0.080733	0.998356
Prkaca	18747	0.275887	0.998356
Prkacb	18749	0.157046	0.998356
Prkca	18750	0.013498	0.998356
Prkcd	18753	0.265843	0.998356
Prkch	18755	0.422566	0.998356
Prkcz	18762	0.248985	0.998356
Pkd1	18763	0.28769	0.998356
Pkd2	18764	0.260589	0.998356
Pkig	18769	-0.37648	0.998356
Pklr	18770	-0.17757	0.998356
Pknox1	18771	0.685807	0.998356
Pkp1	18772	-0.22926	0.998356
Lypla1	18777	0.164334	0.998356
Pla2g2a	18780	0.094749	0.998356
Pla2g2d	18782	-0.59113	0.998356
Pla2g4a	18783	0.171395	0.998356
Plaa	18786	0.060036	0.998356
Serpine1	18787	-0.41414	0.998356
Serpinb2	18788	-9.21717	0.998356
Papola	18789	0.0299	0.998356
Plat	18791	0.119976	0.998356
Plau	18792	-0.32165	0.998356
Plaur	18793	-0.07727	0.998356
Plcb3	18797	0.414434	0.998356
Plcb4	18798	-0.5199	0.998356
Plcd1	18799	0.505352	0.998356
Plcg1	18803	0.125744	0.998356
Pld1	18805	0.037881	0.998356
Pld2	18806	0.019749	0.998356
Pld3	18807	-0.02885	0.998356
Plec	18810	-0.37674	0.998356
Pa2g4	18813	-0.34883	0.998356
Plk1	18817	-0.37911	0.998356
Plod1	18822	0.21075	0.998356
Plp2	18824	-0.2245	0.998356
Lcp1	18826	0.26734	0.998356
Pltp	18830	0.954849	0.998356
Plxna1	18844	0.099271	0.998356
Plxna2	18845	0.069846	0.998356
Pml	18854	1.37548	0.998356
Pms2	18861	0.075303	0.998356
Ppp1r14b	18938	0.294953	0.998356

Pnn	18949	-0.39669	0.998356
Pnp	18950	-0.07107	0.998356
Pola1	18968	-0.18602	0.998356
Pola2	18969	-0.21901	0.998356
Polb	18970	0.771597	0.998356
Pold1	18971	0.038308	0.998356
Pold2	18972	-0.16899	0.998356
Pole	18973	-0.52821	0.998356
Pole2	18974	0.198078	0.998356
Polg	18975	0.002404	0.998356
Cnot7	18983	0.162175	0.998356
Por	18984	0.535118	0.998356
Pou2f1	18986	0.136346	0.998356
Pou6f1	19009	-0.02203	0.998356
Ppap2a	19012	0.118946	0.998356
Med1	19014	-0.13742	0.998356
Ppard	19015	-0.07054	0.998356
Pparg	19016	0.369604	0.998356
Ppargc1a	19017	0.205186	0.998356
Scand1	19018	0.157416	0.998356
Ppfibp2	19024	0.352467	0.998356
Ctsa	19025	-0.17683	0.998356
Sypl	19027	0.016623	0.998356
Ppib	19035	0.09491	0.998356
Ppic	19038	-0.19741	0.998356
Lgals3bp	19039	0.524303	0.998356
Ppl	19041	0.228114	0.998356
Ppm1a	19042	0.450002	0.998356
Ppm1b	19043	-0.11324	0.998356
Ppox	19044	-0.17813	0.998356
Ppp1ca	19045	-0.03049	0.998356
Ppp1cb	19046	0.374622	0.998356
Ppp2ca	19052	0.316546	0.998356
Ppp2cb	19053	0.048664	0.998356
Ppp2r3d	19054	0.404337	0.998356
Ppp3ca	19055	0.084922	0.998356
Ppp3cb	19056	0.026207	0.998356
Ppp3r1	19058	0.015041	0.998356
Ppp5c	19060	-0.33346	0.998356
Inpp5k	19062	0.357647	0.998356
Ppt1	19063	-0.25444	0.998356
Nup88	19069	0.008406	0.998356
Mob4	19070	0.363225	0.998356
Prep	19072	0.107872	0.998356
Srgn	19073	-0.32352	0.998356
Prim1	19075	0.169384	0.998356
Prim2	19076	0.375553	0.998356

Prkab1	19079	0.244018	0.998356
Prkag1	19082	0.158014	0.998356
Prkar1a	19084	-0.12514	0.998356
Prkar1b	19085	-0.17576	0.998356
Prkar2a	19087	-0.07056	0.998356
Prkcsh	19089	-0.19573	0.998356
Prkdc	19090	0.098611	0.998356
Prkg2	19092	0.557038	0.998356
Eif2ak2	19106	0.355167	0.998356
Prkx	19108	0.358203	0.998356
Procr	19124	-0.23116	0.998356
Prodh	19125	0.474523	0.998356
Prom1	19126	-0.11298	0.998356
Pros1	19128	0.002272	0.998356
Prox1	19130	0.18553	0.998356
Prpf4b	19134	-0.17341	0.998356
Prps1	19139	0.227999	0.998356
Lgmn	19141	0.352366	0.998356
Prss12	19142	0.268968	0.998356
St14	19143	0.380537	0.998356
Npepps	19155	0.225837	0.998356
Psap	19156	0.052375	0.998356
Cyth1	19157	0.035833	0.998356
Cyth2	19158	-0.60051	0.998356
Cyth3	19159	0.226459	0.998356
Psen1	19164	0.267536	0.998356
Psen2	19165	0.39989	0.998356
Psma2	19166	-0.07608	0.998356
Psma3	19167	-0.03256	0.998356
Psmb1	19170	0.166495	0.998356
Psmb10	19171	-0.22626	0.998356
Psmb5	19173	0.139239	0.998356
Psmb6	19175	-0.18182	0.998356
Psmb7	19177	0.260887	0.998356
Psmc1	19179	-0.30227	0.998356
Psmc2	19181	0.543994	0.998356
Psmc3	19182	-0.20988	0.998356
Psmc3ip	19183	0.523929	0.998356
Psmc5	19184	-0.21216	0.998356
Psmd4	19185	-0.10185	0.998356
Psme1	19186	-0.45554	0.998356
Psme2	19188	-0.09695	0.998356
Psme3	19192	-0.0819	0.998356
Pstpip2	19201	0.485613	0.998356
Ptafr	19204	0.031322	0.998356
Ptbp1	19205	-0.32533	0.998356
Ptch1	19206	0.074249	0.998356

Ptdss1	19210	0.266432	0.998356
Pten	19211	-0.0458	0.998356
Pter	19212	-0.19175	0.998356
Ptgds	19215	1.33541	0.998356
Ptger1	19216	0.096761	0.998356
Ptger4	19219	0.850098	0.998356
Ptgfrn	19221	0.123578	0.998356
Ptgs1	19224	0.631212	0.998356
Ptgs2	19225	-0.52811	0.998356
Ptk2b	19229	0.083407	0.998356
Twf1	19230	0.226736	0.998356
Ptma	19231	-0.57481	0.998356
Tmsb10	19240	0.420069	0.998356
Tmsb4x	19241	0.454913	0.998356
Ptn	19242	-0.63373	0.998356
Ptp4a1	19243	0.061778	0.998356
Ptp4a2	19244	0.287852	0.998356
Ptp4a3	19245	-0.68249	0.998356
Ptpn1	19246	-0.25458	0.998356
Ptpn11	19247	0.083888	0.998356
Ptpn12	19248	0.123016	0.998356
Ptpn13	19249	-0.41037	0.998356
Ptpn14	19250	0.287578	0.998356
Dusp1	19252	-0.36746	0.998356
Ptpn18	19253	-0.00869	0.998356
Ptpn2	19255	0.209639	0.998356
Ptpn4	19258	-0.07201	0.998356
Sirpa	19261	0.44311	0.998356
Ptpra	19262	0.55586	0.998356
Ptprc	19264	0.144181	0.998356
Ptprcap	19265	-0.88579	0.998356
Ptprd	19266	-0.81353	0.998356
Ptpre	19267	0.223126	0.998356
Ptprf	19268	-0.10903	0.998356
Ptprg	19270	0.327107	0.998356
Ptprk	19272	-0.10659	0.998356
Ptpru	19273	0.442385	0.998356
Ptprn2	19276	0.057307	0.998356
Ptpro	19277	0.227625	0.998356
Ptprr	19279	0.02934	0.998356
Ptprs	19280	0.132094	0.998356
Ptprz1	19283	-0.10208	0.998356
Ptrf	19285	-0.13059	0.998356
Pts	19286	0.339907	0.998356
Pura	19290	0.37623	0.998356
Purb	19291	0.001664	0.998356
Pvrl2	19294	0.670119	0.998356

Pvt1	19296	-0.44399	0.998356
Pex19	19298	-0.09735	0.998356
Abcd3	19299	0.075278	0.998356
Abcd4	19300	0.666039	0.998356
Pxmp2	19301	-0.00448	0.998356
Pxmp3	19302	-1.03214	0.998356
Pxn	19303	0.108537	0.998356
Pex5	19305	0.508268	0.998356
Qk	19317	-0.48716	0.998356
Rab10	19325	0.23409	0.998356
Rab11b	19326	-0.17684	0.998356
Rab12	19328	-0.06407	0.998356
Rab18	19330	0.021954	0.998356
Rab19	19331	-0.23897	0.998356
Rab20	19332	0.378878	0.998356
Rab22a	19334	0.066781	0.998356
Rab23	19335	-0.15776	0.998356
Rab24	19336	0.105034	0.998356
Rab33b	19338	-0.11484	0.998356
Rab3a	19339	0.165972	0.998356
Rab3d	19340	-0.07133	0.998356
Rab4a	19341	0.255231	0.998356
Rab4b	19342	-0.12122	0.998356
Rab5b	19344	0.236838	0.998356
Rab5c	19345	0.497461	0.998356
Rab6a	19346	0.328508	0.998356
Dennd5a	19347	0.018288	0.998356
Kif20a	19348	-1.01151	0.998356
Rab7	19349	0.013484	0.998356
Rabggtb	19352	0.755742	0.998356
Rac1	19353	0.229778	0.998356
Rac2	19354	-0.2925	0.998356
Rad1	19355	-0.34341	0.998356
Rad17	19356	0.163926	0.998356
Rad21	19357	-0.01686	0.998356
Rad23a	19358	-0.17448	0.998356
Rad23b	19359	0.140204	0.998356
Rad50	19360	-0.36515	0.998356
Rad51	19361	0.047892	0.998356
Rad51ap1	19362	-0.13914	0.998356
Rad51l3	19364	-0.03225	0.998356
Rad52	19365	-0.14739	0.998356
Rad9	19367	0.142027	0.998356
Rai1	19377	0.058383	0.998356
Raly	19383	-1.59017	0.998356
Ran	19384	-0.31315	0.998356
Ranbp1	19385	0.559432	0.998356

Ranbp2	19386	-0.13032	0.998356
Rangap1	19387	-0.28518	0.998356
Rara	19401	0.402471	0.998356
Rarg	19411	0.344321	0.998356
Rasa3	19414	0.294252	0.998356
Rasd1	19416	0.014784	0.998356
Rb1	19645	0.30493	0.998356
Rbbp4	19646	0.391845	0.998356
Rbbp6	19647	-0.11802	0.998356
Rbl1	19650	-0.13332	0.998356
Rbl2	19651	-0.00469	0.998356
Rbm3	19652	-0.21704	0.998356
Rbm4	19653	0.150774	0.998356
Rbm6	19654	-0.13312	0.998356
Rbmx	19655	-1.93312	0.998356
Rbmxl1	19656	-0.52878	0.998356
Rbp1	19659	0.534755	0.998356
Rbp2	19660	0.767581	0.998356
Rbp4	19662	-0.44313	0.998356
Rbpms	19663	-0.25686	0.998356
Rbpj	19664	0.757651	0.998356
Rce1	19671	-0.00278	0.998356
Rcn1	19672	0.215841	0.998356
Rdx	19684	0.385915	0.998356
Rfc1	19687	-0.10406	0.998356
Recql	19691	1.14402	0.998356
Reg3g	19695	0.189953	0.998356
Rel	19696	0.288807	0.998356
Rela	19697	-0.0748	0.998356
Relb	19698	0.302345	0.998356
Renbp	19703	0.182445	0.998356
Upf1	19704	-2.03686	0.998356
Reps1	19707	1.0356	0.998356
Dpf2	19708	-0.25653	0.998356
Rest	19712	-0.13774	0.998356
Rev3l	19714	-0.04817	0.998356
Bex1	19716	0.58307	0.998356
Rfc2	19718	-0.13878	0.998356
Rfng	19719	0.014333	0.998356
Trim27	19720	-0.08583	0.998356
Rfx1	19724	0.101229	0.998356
Rfx3	19726	0.748124	0.998356
Rfxank	19727	-0.16781	0.998356
Slc50a1	19729	-0.08484	0.998356
Ralgds	19730	0.753034	0.998356
Rgl2	19732	0.031381	0.998356
Rgs16	19734	-0.16664	0.998356

Rgs2	19735	-0.55443	0.998356
Rgs5	19737	0.19524	0.998356
Rheb	19744	-0.05729	0.998356
Rnase1	19752	-0.59814	0.998356
Ring1	19763	-0.00913	0.998356
Ralbp1	19765	0.084545	0.998356
Ripk1	19766	0.107717	0.998356
Rit1	19769	0.485022	0.998356
Xpr1	19775	-0.3742	0.998356
C80913	19777	0.040108	0.998356
Rnaseh1	19819	-0.58392	0.998356
Rlim	19820	-0.14448	0.998356
Rnf2	19821	0.067876	0.998356
Rnf4	19822	-0.07699	0.998356
Rnf7	19823	0.050858	0.998356
Trim10	19824	0.622064	0.998356
Rnps1	19826	-0.49903	0.998356
Robo1	19876	-0.19746	0.998356
Rock1	19877	0.066113	0.998356
Rock2	19878	0.024527	0.998356
Mst1r	19882	0.439083	0.998356
Rorc	19885	-0.36485	0.998356
Rp2h	19889	0.49929	0.998356
Rpa2	19891	-0.23424	0.998356
Rpia	19895	-0.04474	0.998356
Rpl18	19899	-0.13134	0.998356
Rpl19	19921	-0.49693	0.998356
Rpl21	19933	-0.04307	0.998356
Rpl22	19934	-0.17173	0.998356
Mrpl23	19935	0.449359	0.998356
Rpl26	19941	-0.41953	0.998356
Rpl27	19942	0.129483	0.998356
Rpl28	19943	0.240016	0.998356
Rpl29	19944	0.098208	0.998356
Rpl30	19946	0.321613	0.998356
Rpl32	19951	-0.32418	0.998356
Rpl37a	19981	0.410384	0.998356
Rpl36a	19982	0.210244	0.998356
Rpl6	19988	-0.23871	0.998356
Rpl7	19989	0.080423	0.998356
Rpl9	20005	0.250472	0.998356
Rpn2	20014	0.091239	0.998356
Polr1c	20016	0.093826	0.998356
Polr1b	20017	-0.37136	0.998356
Polr1d	20018	-0.06159	0.998356
Polr1a	20019	-0.20976	0.998356
Polr2a	20020	-0.02299	0.998356

Polr2c	20021	0.016898	0.998356
Polr2j	20022	0.255134	0.998356
Sub1	20024	0.34397	0.998356
Rps12	20042	0.12965	0.998356
Rps14	20044	0.522605	0.998356
Rps15	20054	0.747734	0.998356
Rps16	20055	-0.19471	0.998356
Rps17	20068	0.552092	0.998356
Rps18	20084	0.816806	0.998356
Rps19	20085	-0.03662	0.998356
Rps24	20088	0.433579	0.998356
Rps3a	20091	-0.24675	0.998356
Rps4x	20102	-0.53482	0.998356
Rps5	20103	0.338162	0.998356
Rps6	20104	-0.18901	0.998356
Rps6ka1	20111	0.117188	0.998356
Rps6ka2	20112	0.314078	0.998356
Rps7	20115	0.370973	0.998356
Rps8	20116	-0.0876	0.998356
Trim30a	20128	0.827077	0.998356
Rras	20130	0.448303	0.998356
Rrm1	20133	-0.24793	0.998356
Rrm2	20135	-0.24795	0.998356
Dhrs3	20148	-0.07147	0.998356
Rsu1	20163	0.01714	0.998356
Rtkn	20166	-0.10876	0.998356
Rtn3	20168	0.460399	0.998356
Hps6	20170	0.186513	0.998356
Ruvbl2	20174	-0.51103	0.998356
Rxra	20181	0.273303	0.998356
Rxrb	20182	1.49403	0.998356
Uimc1	20184	0.219235	0.998356
Ncor1	20185	-0.1903	0.998356
Nr1h4	20186	0.9699	0.998356
Ryk	20187	0.199268	0.998356
S100a10	20194	0.351598	0.998356
S100a11	20195	0.1165	0.998356
S100a13	20196	0.244959	0.998356
S100a3	20197	-0.4171	0.998356
S100a4	20198	0.457081	0.998356
S100a6	20200	0.794317	0.998356
S100a8	20201	0.079554	0.998356
S100a9	20202	-0.41051	0.998356
Saa3	20210	-0.32027	0.998356
Acsm3	20216	1.23858	0.998356
Khdrbs1	20218	0.07094	0.998356
Sap18	20220	0.073815	0.998356

Sf3a2	20222	-0.40254	0.998356
Sar1a	20224	-0.11834	0.998356
Sars	20226	-0.58552	0.998356
Sart1	20227	-0.04508	0.998356
Atxn1	20238	0.189386	0.998356
Atxn2	20239	0.086635	0.998356
Scd1	20249	0.163316	0.998356
Scd2	20250	-0.26032	0.998356
Clec11a	20256	0.131977	0.998356
Scnn1b	20277	0.470299	0.998356
Scp2	20280	-0.26141	0.998356
Zc3h7b	20286	-0.1186	0.998356
Msr1	20288	-0.98953	0.998356
Ccl12	20293	-0.01556	0.998356
Ccl17	20295	-0.08387	0.998356
Ccl20	20297	-1.22827	0.998356
Ccl25	20300	0.625486	0.998356
Ccl27a	20301	-2.15242	0.998356
Ccl3	20302	-0.68254	0.998356
Ccl5	20304	-0.37161	0.998356
Ccl6	20305	-0.01102	0.998356
Ccl7	20306	-0.10041	0.998356
Ccl8	20307	0.666601	0.998356
Ccl9	20308	-0.63974	0.998356
Cxcl5	20311	-0.88042	0.998356
Cx3cl1	20312	0.170123	0.998356
Sdf2	20316	0.389018	0.998356
Sdf4	20318	0.344258	0.998356
Nptn	20320	0.514927	0.998356
Frrs1	20321	-0.82768	0.998356
Sord	20322	0.352621	0.998356
Sec22b	20333	0.032876	0.998356
Sec23a	20334	0.158885	0.998356
Sec61g	20335	0.458613	0.998356
Exoc4	20336	-0.11772	0.998356
Sel1l	20338	-0.1329	0.998356
Glg1	20340	0.265299	0.998356
Selenbp1	20341	-0.82264	0.998356
Sell	20343	-0.39608	0.998356
Selplg	20345	-0.09798	0.998356
Sema3b	20347	0.164447	0.998356
Sema3c	20348	0.500634	0.998356
Sema3e	20349	-0.26261	0.998356
Sema3f	20350	0.277587	0.998356
Sema4a	20351	0.112906	0.998356
Sema4b	20352	-0.14818	0.998356
Sema4c	20353	0.290459	0.998356

Sema4d	20354	-0.20864	0.998356
Sema5a	20356	-0.42302	0.998356
Sema6a	20358	0.426628	0.998356
Sema7a	20361	-0.05541	0.998356
Sepp1	20363	0.571777	0.998356
Sepw1	20364	-0.25822	0.998356
Serf1	20365	0.400286	0.998356
Sfpi1	20375	0.152144	0.998356
Srsf2	20382	0.286258	0.998356
Srsf3	20383	-0.3544	0.998356
Srsf5	20384	0.197274	0.998356
Sgk1	20393	-0.21426	0.998356
Sgpl1	20397	0.244269	0.998356
Sh2b1	20399	-0.23269	0.998356
Sh3bp1	20401	-0.71623	0.998356
Zfp106	20402	-0.00409	0.998356
Itsn2	20403	-0.43879	0.998356
Sh3gl1	20405	-0.35725	0.998356
Ostf1	20409	0.542178	0.998356
Sorbs3	20410	0.183055	0.998356
Shc1	20416	0.550121	0.998356
Shcbp1	20419	-0.11848	0.998356
Shfm1	20422	0.275045	0.998356
Shh	20423	1.0328	0.998356
Shmt1	20425	-0.36461	0.998356
Cyfip1	20430	1.59147	0.998356
Siah1a	20437	0.194079	0.998356
Siah1b	20438	0.124407	0.998356
Siah2	20439	0.293153	0.998356
St6gal1	20440	-1.51001	0.998356
St3gal3	20441	0.612981	0.998356
St3gal4	20443	-0.01095	0.998356
St6galnac2	20446	0.056125	0.998356
St6galnac4	20448	-0.17294	0.998356
Ptk6	20459	0.660506	0.998356
Stil	20460	-0.08674	0.998356
Tra2b	20462	0.148354	0.998356
Cox7a2l	20463	-0.01294	0.998356
Sin3a	20466	-1.37984	0.998356
Sin3b	20467	-0.22764	0.998356
Six4	20474	0.390651	0.998356
Vps4b	20479	0.055497	0.998356
Clpb	20480	-0.07466	0.998356
Ski	20481	0.021094	0.998356
Skil	20482	0.31016	0.998356
Sla	20491	0.280639	0.998356
Slbp	20492	-0.27909	0.998356

Slc10a2	20494	0.6864	0.998356
Slc12a2	20496	-0.38712	0.998356
Slc12a4	20498	-7.16168	0.998356
Slc12a7	20499	-0.06463	0.998356
Slc16a1	20501	0.088193	0.998356
Slc19a1	20509	-0.53468	0.998356
Slc1a1	20510	-0.81236	0.998356
Slc1a5	20514	-0.11299	0.998356
Slc20a1	20515	-0.50484	0.998356
Slc20a2	20516	0.139318	0.998356
Slc22a1	20517	-0.18469	0.998356
Slc22a5	20520	0.228477	0.998356
Slc25a14	20523	0.669854	0.998356
Slc25a17	20524	0.326044	0.998356
Slc2a1	20525	0.501375	0.998356
Slc31a1	20529	-0.22101	0.998356
Slc31a2	20530	0.284515	0.998356
Slc4a1ap	20534	0.017697	0.998356
Slc4a2	20535	8.76221	0.998356
Slc5a1	20537	-0.0624	0.998356
Slc7a5	20539	0.117651	0.998356
Slc7a7	20540	-0.4577	0.998356
Slc9a1	20544	-0.01316	0.998356
Slfn1	20555	-0.013	0.998356
Slfn2	20556	0.498	0.998356
Smarca4	20586	0.728496	0.998356
Smarcb1	20587	0.01277	0.998356
Smarcc1	20588	-0.27307	0.998356
Ighmbp2	20589	0.050716	0.998356
Kdm5c	20591	-0.14166	0.998356
Kdm5d	20592	0.216336	0.998356
Smn1	20595	-0.35232	0.998356
Smpd1	20597	-0.07367	0.998356
Smpd2	20598	-0.09135	0.998356
Ncor2	20602	-0.70818	0.998356
Sms	20603	0.005205	0.998356
Sumo3	20610	-0.0028	0.998356
Snapin	20615	-0.14267	0.998356
Snca	20617	-0.81962	0.998356
Sncg	20618	0.293292	0.998356
Snap23	20619	-0.103	0.998356
Plk2	20620	-0.45661	0.998356
Snn	20621	-0.02354	0.998356
Snrk	20623	0.298395	0.998356
Eftud2	20624	0.120891	0.998356
Snrpc	20630	-0.04108	0.998356
Snrnp70	20637	-0.38817	0.998356

Snrpb	20638	-0.2308	0.998356
Snrpb2	20639	0.286405	0.998356
Snrpd1	20641	0.035602	0.998356
Snrpe	20643	-0.32453	0.998356
Snta1	20648	0.782321	0.998356
Sntb1	20649	-0.03608	0.998356
Sntb2	20650	0.245132	0.998356
Soat1	20652	-0.0671	0.998356
Sod1	20655	0.496505	0.998356
Sod2	20656	-0.10093	0.998356
Sod3	20657	0.154904	0.998356
Son	20658	0.126442	0.998356
Sorl1	20660	0.503104	0.998356
Sort1	20661	-0.07026	0.998356
Sos1	20662	-0.17982	0.998356
Sos2	20663	0.184542	0.998356
Sox13	20668	-0.43	0.998356
Sox17	20671	-0.45304	0.998356
Sox18	20672	0.26385	0.998356
Sox4	20677	-0.14667	0.998356
Sox9	20682	-0.02067	0.998356
Sp1	20683	0.052219	0.998356
Sp100	20684	0.638457	0.998356
Spa17	20686	0.113335	0.998356
Sp3	20687	-0.10592	0.998356
Sp4	20688	-0.05735	0.998356
Sparc	20692	0.313601	0.998356
Serpinb6b	20708	0.451533	0.998356
Serpina3g	20715	0.535157	0.998356
Serpina3n	20716	-0.1503	0.998356
Serpinb6a	20719	1.56598	0.998356
Serpine2	20720	-0.31262	0.998356
Serpinb9	20723	0.30784	0.998356
Serpinb5	20724	0.273667	0.998356
Spin1	20729	0.057573	0.998356
Spint1	20732	0.448326	0.998356
Spint2	20733	0.040492	0.998356
Spna2	20740	0.153298	0.998356
Spnb1	20741	0.316267	0.998356
Spnb2	20742	0.035383	0.998356
Spnb3	20743	0.404241	0.998356
Strbp	20744	0.028901	0.998356
Spop	20747	0.031546	0.998356
Spp1	20750	-1.07492	0.998356
Spr	20751	0.390141	0.998356
Sprr2b	20756	0.509262	0.998356
Sprr2d	20758	0.273618	0.998356

Sprr2e	20759	1.97599	0.998356
Sprr2f	20760	1.64487	0.998356
Sprr2g	20761	2.24253	0.998356
Sephs2	20768	0.074857	0.998356
Sptlc2	20773	0.23905	0.998356
Sqle	20775	-0.33032	0.998356
Scarb1	20778	0.011511	0.998356
Src	20779	0.485856	0.998356
Srebf1	20787	0.136183	0.998356
Srebf2	20788	-0.01488	0.998356
Srf	20807	-0.03533	0.998356
Srm	20810	-0.77102	0.998356
Srp14	20813	0.068457	0.998356
Srpk1	20815	-0.30895	0.998356
Srpk2	20817	0.192587	0.998356
Srprb	20818	0.124438	0.998356
Trim21	20821	-0.01162	0.998356
Trove2	20822	0.046194	0.998356
Ssb	20823	0.22976	0.998356
Nhp2l1	20826	-0.27695	0.998356
Ssrp1	20833	2.56218	0.998356
Zfp143	20841	-0.17518	0.998356
Stag1	20842	-0.18104	0.998356
Stag2	20843	0.057849	0.998356
Stam	20844	0.11923	0.998356
Stat1	20846	0.03734	0.998356
Stat2	20847	0.126879	0.998356
Stat3	20848	-0.26565	0.998356
Stat5a	20850	0.858207	0.998356
Stat5b	20851	0.208355	0.998356
Stat6	20852	-0.07156	0.998356
Stau1	20853	-0.6467	0.998356
Stc1	20855	0.12267	0.998356
Stc2	20856	-0.00251	0.998356
Stim1	20866	0.241365	0.998356
Stip1	20867	-0.45227	0.998356
Stk10	20868	-0.08925	0.998356
Stk11	20869	0.021875	0.998356
Stk16	20872	-0.37721	0.998356
Plk4	20873	-0.07037	0.998356
Slk	20874	0.043843	0.998356
Aurkb	20877	0.076153	0.998356
Aurka	20878	-0.36875	0.998356
Bhlhe40	20893	0.273717	0.998356
Stra6	20897	-0.97996	0.998356
Strap	20901	-0.02045	0.998356
Stx1a	20907	0.054648	0.998356

Stx3	20908	0.359781	0.998356
Stx4a	20909	0.065646	0.998356
Stxbp1	20910	0.097721	0.998356
Stxbp2	20911	0.003174	0.998356
Sucla2	20916	-0.07239	0.998356
Suclg2	20917	0.315318	0.998356
Eif1	20918	-0.00719	0.998356
Supt4h1	20922	0.572746	0.998356
Supt5h	20924	-0.13986	0.998356
Supt6h	20926	-0.08211	0.998356
Surf1	20930	-0.0278	0.998356
Surf2	20931	-0.13938	0.998356
Surf4	20932	0.10596	0.998356
Med22	20933	0.932519	0.998356
Surf6	20935	0.015235	0.998356
Suv39h1	20937	-0.16188	0.998356
Swap70	20947	-0.33796	0.998356
Syk	20963	0.287024	0.998356
Sdc1	20969	-0.05238	0.998356
Sdc4	20971	0.030841	0.998356
Syngr1	20972	0.467319	0.998356
Syngr2	20973	0.134666	0.998356
Synj2	20975	-0.02644	0.998356
T	20998	0.012516	0.998356
Tac1	21333	-1.07465	0.998356
Tacc3	21335	0.125528	0.998356
Taf1a	21339	-0.1727	0.998356
Taf1b	21340	0.719706	0.998356
Taf1c	21341	-0.41231	0.998356
Taf6	21343	-0.01144	0.998356
Tagln	21345	-1.4595	0.998356
Tagln2	21346	-0.35136	0.998356
Taldo1	21351	0.025567	0.998356
Tank	21353	0.280023	0.998356
Tap1	21354	-0.42403	0.998356
Tap2	21355	0.06728	0.998356
Tapbp	21356	0.405242	0.998356
Tarbp2	21357	0.440164	0.998356
Slc6a6	21366	-0.01144	0.998356
Tbca	21371	0.60661	0.998356
Tbl1x	21372	0.120123	0.998356
Tbp	21374	0.080374	0.998356
Tbrg1	21376	-0.01098	0.998356
Tbrg3	21378	-0.1417	0.998356
Tbrg4	21379	-0.00246	0.998356
Tbx3	21386	-0.2914	0.998356
Tcea1	21399	-0.26483	0.998356

Tcea2	21400	0.713613	0.998356
Tcea3	21401	-0.11496	0.998356
Skp1a	21402	0.006565	0.998356
Hnf1a	21405	-0.21351	0.998356
Tcf12	21406	0.473706	0.998356
Hnf1b	21410	-0.2077	0.998356
Tcf20	21411	0.135104	0.998356
Tcf21	21412	0.531095	0.998356
Tcf4	21413	-0.32049	0.998356
Tcf7l1	21415	0.036652	0.998356
Tcf7l2	21416	1.14221	0.998356
Tfcp2	21422	-0.15075	0.998356
Tcf3	21423	-0.51272	0.998356
Tfeb	21425	0.023291	0.998356
Vps72	21427	-0.19712	0.998356
Mlx	21428	2.82665	0.998356
Ubtf	21429	0.609549	0.998356
Tcn2	21452	-0.07288	0.998356
Tcof1	21453	-0.01349	0.998356
Tcp1	21454	-0.16612	0.998356
Phf1	21652	-0.02822	0.998356
Phlda1	21664	0.118712	0.998356
Tdg	21665	-0.10408	0.998356
Prdx2	21672	-0.09019	0.998356
Tead1	21676	0.324959	0.998356
Tead2	21677	0.006405	0.998356
Tead3	21678	-1.0544	0.998356
Alyref	21681	0.132441	0.998356
Tec	21682	-0.98494	0.998356
Tef	21685	0.53747	0.998356
Tep1	21745	0.382119	0.998356
Terf1	21749	-0.09537	0.998356
Terf2	21750	0.342438	0.998356
Tes	21753	0.466216	0.998356
Tesk1	21754	0.372188	0.998356
Morf4l1	21761	-0.10878	0.998356
Psmd2	21762	0.002119	0.998356
Tex2	21763	0.087616	0.998356
Tex261	21766	-0.22319	0.998356
Tex264	21767	-0.0715	0.998356
Zfand3	21769	0.170754	0.998356
Ppp2r5d	21770	0.363079	0.998356
Cirh1a	21771	-0.08558	0.998356
Tfam	21780	0.092957	0.998356
Tfdp1	21781	0.121313	0.998356
Tff3	21786	0.487372	0.998356
Tfg	21787	-0.10543	0.998356

Tfpi	21788	-0.07109	0.998356
Tgfa	21802	0.334344	0.998356
Tgfb1	21803	-0.60124	0.998356
Tsc22d1	21807	0.160398	0.998356
Tgfbi	21810	0.143276	0.998356
Tgfbr1	21812	0.190958	0.998356
Tgfbr2	21813	0.18033	0.998356
Tgif1	21815	-0.38717	0.998356
Tgm1	21816	-1.20357	0.998356
Tgm2	21817	0.215171	0.998356
Ift88	21821	0.200717	0.998356
Tgtp1	21822	-0.68402	0.998356
Thbd	21824	0.494897	0.998356
Thbs1	21825	0.279481	0.998356
Thbs4	21828	-0.45797	0.998356
Thra	21833	0.51257	0.998356
Thy1	21838	0.822636	0.998356
Tia1	21841	0.095657	0.998356
Tial1	21843	-0.12488	0.998356
Tiam1	21844	-0.25928	0.998356
Klf10	21847	0.217745	0.998356
Trim24	21848	0.178617	0.998356
Trim28	21849	-0.15524	0.998356
Timeless	21853	-1.72642	0.998356
Timm17a	21854	-0.18305	0.998356
Timm17b	21855	0.335217	0.998356
Timm44	21856	-0.01152	0.998356
Timp1	21857	0.467151	0.998356
Timp2	21858	0.250022	0.998356
Timp3	21859	0.360867	0.998356
Atp6v0a2	21871	-0.01482	0.998356
Tjp1	21872	0.084057	0.998356
Tjp2	21873	1.14493	0.998356
Tk1	21877	0.702779	0.998356
Tkt	21881	-0.05144	0.998356
Tle1	21885	0.237666	0.998356
Tle3	21887	0.315024	0.998356
Tle4	21888	0.416444	0.998356
Tln1	21894	0.343584	0.998356
Tlr1	21897	0.109526	0.998356
Tlr4	21898	-0.18023	0.998356
Otop1	21906	0.552979	0.998356
Tspan7	21912	-0.09987	0.998356
Dtymk	21915	0.552626	0.998356
Tmpo	21917	0.188539	0.998356
Tnc	21923	-0.70451	0.998356
Tnf	21926	-0.17314	0.998356

Tnfaip1	21927	0.298323	0.998356
Tnfaip2	21928	-0.26879	0.998356
Tnfaip3	21929	0.45123	0.998356
Tnfrsf10b	21933	-0.15893	0.998356
Tnfrsf11a	21934	0.151303	0.998356
Tnfrsf18	21936	-0.47801	0.998356
Tnfrsf1a	21937	0.208182	0.998356
Tnfrsf1b	21938	0.249627	0.998356
Tnfrsf9	21942	-0.62838	0.998356
Dedd	21945	-0.34871	0.998356
Pglyrp1	21946	0.623915	0.998356
Tnfsf9	21950	-0.12424	0.998356
Tnks	21951	0.028073	0.998356
Tnni1	21952	-0.23454	0.998356
Tnnt2	21956	-0.24937	0.998356
Tom1	21968	0.149253	0.998356
Top1	21969	-0.21488	0.998356
Top2a	21973	-0.22869	0.998356
Top2b	21974	0.002073	0.998356
Top3a	21975	0.011165	0.998356
Top3b	21976	-0.28324	0.998356
Ppp1r13b	21981	-0.16202	0.998356
Tmem165	21982	0.40292	0.998356
Tpbg	21983	0.029496	0.998356
Tpd52	21985	-0.18296	0.998356
Tpd52l1	21987	0.184117	0.998356
Tpi1	21991	0.138824	0.998356
Tpm1	22003	-1.96467	0.998356
Tpmt	22017	-0.53792	0.998356
Tpp2	22019	-0.21337	0.998356
Tpst2	22022	-0.27346	0.998356
Nr2c1	22025	-0.22569	0.998356
Nr2c2	22026	0.079666	0.998356
Hsp90b1	22027	-0.21432	0.998356
Traf2	22030	0.126545	0.998356
Traf3	22031	0.151454	0.998356
Traf4	22032	0.092661	0.998356
Traf6	22034	0.217892	0.998356
Tnfsf10	22035	-0.43697	0.998356
Traip	22036	0.225806	0.998356
Plscr1	22038	0.132912	0.998356
Trex1	22040	0.327401	0.998356
Trf	22041	0.149288	0.998356
Tfrc	22042	-0.16318	0.998356
Trip6	22051	0.147715	0.998356
Tob1	22057	0.134994	0.998356
Trp53	22059	-0.85465	0.998356

Trp63	22061	-2.2833	0.998356
Tpt1	22070	-0.08667	0.998356
Ctr9	22083	0.092102	0.998356
Tsc2	22084	-0.53193	0.998356
Tsg101	22088	-0.36062	0.998356
Rsph1	22092	-1.02485	0.998356
Tsn	22099	0.130438	0.998356
Tspyl1	22110	0.059716	0.998356
Phlda2	22113	-0.73281	0.998356
Tst	22117	0.067878	0.998356
Rpl13a	22121	0.344854	0.998356
Tsta3	22122	0.388813	0.998356
Psmd3	22123	-0.31024	0.998356
Ttc3	22129	-0.03203	0.998356
Ttf1	22130	0.07886	0.998356
Tgoln1	22134	0.478131	0.998356
Tgoln2	22135	-0.57069	0.998356
Ttk	22137	-0.47287	0.998356
Ttr	22139	0.58097	0.998356
Tuba1a	22142	-0.24292	0.998356
Tuba1b	22143	-0.29381	0.998356
Tuba4a	22145	0.198778	0.998356
Tuba1c	22146	-0.13423	0.998356
Tubb2a	22151	0.511361	0.998356
Tubb3	22152	-1.29737	0.998356
Tubb4a	22153	-0.11464	0.998356
Tubb5	22154	-0.08156	0.998356
Tuft1	22156	0.256603	0.998356
Tulp3	22158	0.032284	0.998356
Tnfrsf4	22163	0.511188	0.998356
Txn1	22166	0.032243	0.998356
Cmpk2	22169	0.318035	0.998356
Tyms	22171	0.276098	0.998356
Tyrobp	22177	0.268363	0.998356
Zrsr1	22183	0.246904	0.998356
Zrsr2	22184	0.473633	0.998356
U2af2	22185	0.364928	0.998356
Uba52	22186	0.225376	0.998356
Ubb	22187	-0.53994	0.998356
Ubc	22190	-0.23285	0.998356
Ube2m	22192	-1.18801	0.998356
Ube2e3	22193	0.421236	0.998356
Ube2e1	22194	0.292938	0.998356
Ube2l3	22195	0.203907	0.998356
Ube2i	22196	-0.11674	0.998356
Uba3	22200	1.02127	0.998356
Uba1	22201	0.159548	0.998356

Ube2a	22209	0.076955	0.998356
Ube2b	22210	0.267329	0.998356
Ube2g2	22213	-0.0967	0.998356
Ube2h	22214	0.110706	0.998356
Ube3a	22215	-0.17413	0.998356
Usp12	22217	0.058005	0.998356
Sumo1	22218	0.01395	0.998356
Ubp1	22221	0.474369	0.998356
Ubr1	22222	0.204163	0.998356
Uchl1	22223	-0.01997	0.998356
Usp10	22224	-0.41449	0.998356
Usp5	22225	0.14621	0.998356
Ucp2	22228	-0.2446	0.998356
Ufd1l	22230	0.318109	0.998356
Slc35a2	22232	0.137086	0.998356
Ugcg	22234	0.218533	0.998356
Ugdh	22235	0.079245	0.998356
Dpysl3	22240	0.328265	0.998356
Ulk1	22241	0.127345	0.998356
Uck1	22245	0.262397	0.998356
Umps	22247	-0.34447	0.998356
Unc13b	22249	0.550222	0.998356
Ung	22256	-0.45895	0.998356
Usp4	22258	0.148394	0.998356
Nr1h3	22259	-0.02101	0.998356
Nr1h2	22260	-0.19659	0.998356
Prap1	22264	2.37444	0.998356
Upk3a	22270	-0.00075	0.998356
Upp1	22271	0.378214	0.998356
Uqcrq	22272	-0.1616	0.998356
Uqcrc1	22273	0.031471	0.998356
Urod	22275	0.004791	0.998356
Uros	22276	0.102687	0.998356
Usf1	22278	0.135189	0.998356
Usf2	22282	0.285546	0.998356
Usp9x	22284	0.217562	0.998356
Utrn	22288	0.135884	0.998356
Kdm6a	22289	0.092311	0.998356
Uty	22290	0.329802	0.998356
Uxt	22294	0.152274	0.998356
Vamp2	22318	0.054008	0.998356
Vamp3	22319	0.053623	0.998356
Vamp8	22320	0.469614	0.998356
Vars	22321	-0.06845	0.998356
Vasp	22323	0.208383	0.998356
Vav1	22324	-0.59397	0.998356
Vav2	22325	0.096749	0.998356

Vbp1	22327	0.01137	0.998356
Vcam1	22329	0.575291	0.998356
Vcl	22330	-0.08923	0.998356
Vdac1	22333	0.223875	0.998356
Vdac2	22334	0.384984	0.998356
Vdac3	22335	-0.11016	0.998356
Vdr	22337	-0.32982	0.998356
Vegfa	22339	-0.85921	0.998356
Vegfb	22340	0.140842	0.998356
Lin7c	22343	-0.20332	0.998356
Vezf1	22344	-0.03794	0.998356
Vhl	22346	0.076057	0.998356
Vil1	22349	0.290045	0.998356
Ezr	22350	0.198403	0.998356
Vill	22351	0.397236	0.998356
Vim	22352	0.271217	0.998356
Vipr1	22354	0.167784	0.998356
Vldlr	22359	0.354743	0.998356
Vnn1	22361	-0.54801	0.998356
Vps45	22365	0.167087	0.998356
Vrk1	22367	0.854394	0.998356
Vwf	22371	0.302087	0.998356
Wars	22375	-0.04696	0.998356
Wbp1	22377	0.214474	0.998356
Wbp2	22378	-0.23259	0.998356
Fmnl3	22379	0.22802	0.998356
Wbp4	22380	-0.21759	0.998356
Wbp5	22381	-0.34064	0.998356
Eif4h	22384	0.012079	0.998356
Baz1b	22385	0.0427	0.998356
Wdr1	22388	0.48828	0.998356
Wee1	22390	-0.25296	0.998356
Wfs1	22393	-0.09855	0.998356
Zmat3	22401	-0.32491	0.998356
Wiz	22404	0.168383	0.998356
Wnt10a	22409	0.381207	0.998356
Wnt5a	22418	0.206893	0.998356
Wnt5b	22419	1.13398	0.998356
Wnt6	22420	0.577017	0.998356
Wrn	22427	-0.17545	0.998356
Dctn6	22428	0.246346	0.998356
Xbp1	22433	-0.1795	0.998356
Xdh	22436	0.025654	0.998356
Xk	22439	-0.3157	0.998356
Atrx	22589	-0.06875	0.998356
Xpc	22591	-0.2889	0.998356
Ercc5	22592	-0.17219	0.998356

Xrcc1	22594	-0.30761	0.998356
Xrcc5	22596	-0.0417	0.998356
Yap1	22601	0.389424	0.998356
Ybx1	22608	0.461008	0.998356
Yes1	22612	-0.26076	0.998356
Siae	22619	-0.39673	0.998356
Slc23a3	22626	1.25681	0.998356
Ywhae	22627	-0.01988	0.998356
Ywhag	22628	-0.47262	0.998356
Ywhah	22629	0.052154	0.998356
Ywhaq	22630	-0.38104	0.998356
Ywhaz	22631	-0.09033	0.998356
Yy1	22632	0.129135	0.998356
Plagl1	22634	0.202748	0.998356
Zap70	22637	0.258197	0.998356
Zfp1	22640	-0.304	0.998356
Zbtb17	22642	-0.08813	0.998356
Zfp101	22643	-0.10873	0.998356
Rnf103	22644	0.509835	0.998356
Zfp13	22654	0.323061	0.998356
Pcgf2	22658	0.358738	0.998356
Zfp148	22661	-0.05321	0.998356
Zfp161	22666	0.016863	0.998356
Sf1	22668	-0.18022	0.998356
Trim26	22670	-0.02295	0.998356
Zfp185	22673	0.115146	0.998356
Zfp207	22680	-0.28992	0.998356
Zfand5	22682	0.273118	0.998356
Zfp239	22685	0.051843	0.998356
Zfp259	22687	-0.37021	0.998356
Zfp26	22688	-0.2505	0.998356
Zscan2	22691	-0.38471	0.998356
Zfp35	22694	0.147863	0.998356
Zfp36	22695	0.068996	0.998356
Zfp37	22696	0.723944	0.998356
Zfp46	22704	-0.05247	0.998356
Zfp51	22709	0.288237	0.998356
Zfp52	22710	-0.168	0.998356
Zfp54	22712	-0.02068	0.998356
Zfp59	22717	-0.4356	0.998356
Zfp60	22718	0.24257	0.998356
Zfp61	22719	0.329495	0.998356
Zfp62	22720	-0.05912	0.998356
Zfp64	22722	-0.28624	0.998356
Zbtb7b	22724	0.004462	0.998356
Zfp90	22751	0.555916	0.998356
Zfp93	22755	-0.22871	0.998356

Zscan12	22758	-0.08064	0.998356
Zfp97	22759	-0.29799	0.998356
Zfpm1	22761	0.073277	0.998356
Zfr	22763	0.038822	0.998356
Zfx	22764	0.131955	0.998356
Zhx1	22770	0.391022	0.998356
Ikzf2	22779	0.239139	0.998356
Slc30a1	22782	0.344285	0.998356
Slc30a4	22785	0.400022	0.998356
Dnajc2	22791	-0.20638	0.998356
Zyx	22793	-0.24193	0.998356
Coro1b	23789	0.01768	0.998356
Coro1c	23790	0.103334	0.998356
Agr2	23795	-0.30093	0.998356
Akt3	23797	-0.33201	0.998356
Amfr	23802	0.417047	0.998356
Arih1	23806	0.160955	0.998356
Arih2	23807	0.266583	0.998356
Ash2l	23808	1.20064	0.998356
Bace1	23821	1.23849	0.998356
Banf1	23825	-1.20245	0.998356
Bpnt1	23827	-0.07526	0.998356
Capn10	23830	0.307752	0.998356
Cd52	23833	0.071026	0.998356
Cdc6	23834	-0.21861	0.998356
Cfdp1	23837	0.404262	0.998356
Klf6	23849	0.629814	0.998356
Def8	23854	0.161065	0.998356
Dido1	23856	-0.07723	0.998356
Dmtf1	23857	-0.17423	0.998356
Dand5	23863	-0.45028	0.998356
Ets1	23871	-0.16886	0.998356
Ets2	23872	0.173345	0.998356
Faim	23873	-0.07195	0.998356
Farsb	23874	-0.01162	0.998356
Fiz1	23877	-0.25541	0.998356
Fxr2	23879	0.075363	0.998356
Fyb	23880	-0.11684	0.998356
G3bp2	23881	-0.20583	0.998356
Gadd45g	23882	0.554132	0.998356
Gmcl1	23885	-0.04859	0.998356
Gdf15	23886	-1.18107	0.998356
Grem1	23892	-0.74911	0.998356
Gtf2h2	23894	0.061525	0.998356
Hax1	23897	0.296204	0.998356
Hs2st1	23908	-0.18556	0.998356
Rhof	23912	0.56816	0.998356

Impdh1	23917	0.470846	0.998356
Impdh2	23918	-0.1789	0.998356
Insl5	23919	-1.69623	0.998356
Jtb	23922	0.104093	0.998356
Katna1	23924	0.18223	0.998356
Map2k5	23938	0.608986	0.998356
Mapk7	23939	0.14287	0.998356
Mta2	23942	-0.147	0.998356
Esyt1	23943	0.260228	0.998356
Mid2	23947	-0.07343	0.998356
Dnajb6	23950	-0.24903	0.998356
Nek3	23954	0.551406	0.998356
Nek4	23955	0.090366	0.998356
Oas1g	23960	0.47125	0.998356
Oasl2	23962	0.519822	0.998356
Odz4	23966	0.114215	0.998356
Pacsin2	23970	-0.16961	0.998356
Papss1	23971	0.087225	0.998356
Papss2	23972	0.21398	0.998356
Pebp1	23980	-0.09576	0.998356
Pcbp1	23983	0.036555	0.998356
Eci2	23986	0.778703	0.998356
Pin1	23988	-0.24342	0.998356
Med24	23989	0.065884	0.998356
Prkra	23992	-0.16354	0.998356
Dazap2	23994	-0.02461	0.998356
Psmc4	23996	0.187396	0.998356
Psmd13	23997	0.301945	0.998356
Twf2	23999	-0.19769	0.998356
Ptpn21	24000	1.39869	0.998356
Tiam2	24001	-0.46681	0.998356
Ik	24010	-0.0425	0.998356
Abce1	24015	-0.12021	0.998356
Rnf13	24017	0.466477	0.998356
Rngtt	24018	-0.24047	0.998356
Scamp2	24044	0.397514	0.998356
Scamp3	24045	0.103254	0.998356
Sgcb	24051	0.741798	0.998356
Sh3yl1	24057	0.478498	0.998356
Sigirr	24058	-0.10693	0.998356
Slco2a1	24059	-0.39759	0.998356
Slc35a1	24060	0.387443	0.998356
Smc1a	24061	-0.20676	0.998356
Spry1	24063	0.006034	0.998356
Spry2	24064	0.373384	0.998356
Spry4	24066	0.076116	0.998356
Srp54a	24067	-0.5895	0.998356

Sra1	24068	-0.0621	0.998356
Sufu	24069	-0.6161	0.998356
Mpdu1	24070	0.193608	0.998356
Synj2bp	24071	0.009972	0.998356
Taf7	24074	-0.45296	0.998356
Taf10	24075	0.672953	0.998356
Gm16515	24083	-0.16046	0.998356
Tlk2	24086	-0.09268	0.998356
Tlr2	24088	-0.21014	0.998356
Tnfsf13b	24099	0.418856	0.998356
Tpra1	24100	0.099888	0.998356
Trex2	24102	0.413662	0.998356
Rbck1	24105	-0.03323	0.998356
Ubl3	24109	0.246481	0.998356
Usp18	24110	0.066909	0.998356
Whsc2	24116	-0.08345	0.998356
Wif1	24117	0.490793	0.998356
Xrn1	24127	0.046527	0.998356
Xrn2	24128	0.019277	0.998356
Ldb3	24131	-0.59595	0.998356
Zfp53	24132	-0.29613	0.998356
Zfp68	24135	0.323775	0.998356
Zeb2	24136	0.191323	0.998356
Ing1	26356	0.49264	0.998356
Angptl2	26360	0.01697	0.998356
Axl	26362	0.279352	0.998356
Btd	26363	0.457558	0.998356
Cd97	26364	-0.36307	0.998356
Ceacam1	26365	0.588885	0.998356
Ceacam10	26366	-0.54602	0.998356
Ceacam2	26367	1.21673	0.998356
Cetn2	26370	0.586729	0.998356
Ciao1	26371	-0.04244	0.998356
Clcn7	26373	0.019616	0.998356
Rfwd2	26374	0.335677	0.998356
Dapp1	26377	0.224511	0.998356
Decr2	26378	0.173277	0.998356
Esrra	26379	0.331865	0.998356
Fgd2	26382	0.679418	0.998356
Fto	26383	-0.03385	0.998356
Gnpda1	26384	0.078241	0.998356
Grk6	26385	-0.01579	0.998356
Ifi202b	26388	-4.63771	0.998356
Lypla2	26394	0.182623	0.998356
Map2k1	26395	0.242265	0.998356
Map2k2	26396	0.340653	0.998356
Map2k3	26397	0.049345	0.998356

Map2k4	26398	0.298151	0.998356
Map2k6	26399	0.043155	0.998356
Map2k7	26400	0.242199	0.998356
Map3k1	26401	0.069595	0.998356
Map3k11	26403	-0.07758	0.998356
Map3k3	26406	0.183794	0.998356
Map3k4	26407	-0.1438	0.998356
Map3k5	26408	-0.00278	0.998356
Map3k7	26409	0.016395	0.998356
Map3k8	26410	-0.05231	0.998356
Mapk1	26413	0.035949	0.998356
Mapk13	26415	0.291768	0.998356
Mapk14	26416	-0.1642	0.998356
Mapk3	26417	0.345339	0.998356
Mapk8	26419	-0.14119	0.998356
Mapk9	26420	3.27644	0.998356
Nbea	26422	0.374256	0.998356
Nubp1	26425	-0.12658	0.998356
Nubp2	26426	-0.04809	0.998356
Creb3l1	26427	-0.53219	0.998356
Orc4	26428	0.256453	0.998356
Orc5	26429	0.159619	0.998356
Parg	26430	0.065634	0.998356
Git2	26431	-0.18873	0.998356
Plod3	26433	-0.11429	0.998356
Prnd	26434	1.08978	0.998356
Psma1	26440	0.077866	0.998356
Psma4	26441	0.051486	0.998356
Psma5	26442	-0.11986	0.998356
Psma6	26443	0.117988	0.998356
Psma7	26444	0.391597	0.998356
Psmb2	26445	-0.11783	0.998356
Poli	26447	-0.47155	0.998356
Rbbp9	26450	-0.07134	0.998356
Rpl27a	26451	-0.38781	0.998356
Sema4g	26456	0.044165	0.998356
Slc27a1	26457	0.168558	0.998356
Zfp146	26465	-0.09606	0.998356
Cul3	26554	0.042179	0.998356
Homer1	26556	-0.04975	0.998356
Homer2	26557	-1.60652	0.998356
Homer3	26558	-0.14423	0.998356
Hunk	26559	0.084147	0.998356
Ncdn	26562	0.094304	0.998356
Ror2	26564	0.053229	0.998356
Pla2g10	26565	0.593075	0.998356
Slc27a4	26569	0.293077	0.998356

Slc7a11	26570	-0.51571	0.998356
Rcn2	26611	-0.11212	0.998356
Cops5	26754	0.51975	0.998356
B3galt2	26878	-0.28778	0.998356
Casp8ap2	26885	0.310394	0.998356
Cenph	26886	-0.30909	0.998356
Chst4	26887	0.170444	0.998356
Cln8	26889	-0.12263	0.998356
Cops4	26891	0.015676	0.998356
Cops6	26893	-0.5208	0.998356
Cops7a	26894	-0.54217	0.998356
Cops7b	26895	0.224813	0.998356
Med14	26896	0.077426	0.998356
Acot1	26897	-0.45466	0.998356
Ddx3y	26900	0.055604	0.998356
Deb1	26901	-0.29358	0.998356
Eif2s3x	26905	0.297943	0.998356
Eif2s3y	26908	-0.25473	0.998356
Exo1	26909	-0.2218	0.998356
Gcat	26912	-3.40843	0.998356
H2afy	26914	0.113017	0.998356
Ern2	26918	-0.22157	0.998356
Zfp346	26919	0.234158	0.998356
Cep110	26920	-0.15598	0.998356
Map4k4	26921	0.240366	0.998356
Mecr	26922	-0.40648	0.998356
Aifm1	26926	0.151246	0.998356
Ppp2r5c	26931	-0.18534	0.998356
Ppp2r5e	26932	0.056048	0.998356
Racgap1	26934	0.057822	0.998356
Mprip	26936	-0.31126	0.998356
Polr3e	26939	-0.14294	0.998356
Ecsit	26940	-0.84802	0.998356
Slc9a3r1	26941	-0.02017	0.998356
Serinc3	26943	0.395574	0.998356
Tinag	26944	0.061177	0.998356
Tpsg1	26945	-0.1531	0.998356
Vat1	26949	0.143415	0.998356
Zw10	26951	0.115207	0.998356
Rpl8	26961	0.171154	0.998356
Cul1	26965	0.253253	0.998356
Pla2g2e	26970	-0.38998	0.998356
Pla2g2f	26971	-0.01034	0.998356
Eif4e2	26987	0.159677	0.998356
Brd7	26992	0.120131	0.998356
Micall1	27008	-0.17626	0.998356
Polk	27015	-0.05891	0.998356

Tspan32	27027	-0.11037	0.998356
Sgsh	27029	0.061334	0.998356
G3bp1	27041	-0.23547	0.998356
Nit1	27045	0.205563	0.998356
Etv3	27049	7.55463	0.998356
Rps3	27050	0.273186	0.998356
Asns	27053	-0.22001	0.998356
Sec23b	27054	0.666387	0.998356
Fkbp9	27055	-0.10889	0.998356
Irf5	27056	10	0.998356
Ncoa4	27057	-0.26673	0.998356
Srp9	27058	0.239955	0.998356
Sh3d19	27059	-0.05958	0.998356
Tcirg1	27060	0.078465	0.998356
Bcap31	27061	-0.00325	0.998356
Cadps	27062	-0.45497	0.998356
B9d1	27078	0.495744	0.998356
Zfp275	27081	0.102139	0.998356
Trappc3	27096	0.062615	0.998356
Eif2ak4	27103	-0.06751	0.998356
Rpl7a	27176	-0.23113	0.998356
Podxl	27205	0.071811	0.998356
Rps11	27207	0.49114	0.998356
Dbf4	27214	0.292236	0.998356
Azi2	27215	0.001542	0.998356
Sgk2	27219	0.382253	0.998356
Chaf1a	27221	-0.32255	0.998356
Trp53bp1	27223	-0.10042	0.998356
Tceb3	27224	0.245489	0.998356
Ddx24	27225	0.006752	0.998356
Pla2g7	27226	-0.02935	0.998356
Plek2	27260	0.497334	0.998356
Cars	27267	0.137061	0.998356
Nufip1	27275	-0.30735	0.998356
Golga5	27277	-0.49847	0.998356
Tnfrsf12a	27279	-0.01552	0.998356
Phlda3	27280	-0.49885	0.998356
Nbn	27354	0.073624	0.998356
Insl6	27356	-0.09419	0.998356
Gyg	27357	-0.36399	0.998356
Defb3	27358	1.22084	0.998356
Add3	27360	0.219838	0.998356
Sepx1	27361	0.143819	0.998356
Dnajb9	27362	-0.45309	0.998356
Srr	27364	0.268401	0.998356
Txnl4a	27366	-3.20819	0.998356
Rpl3	27367	0.194967	0.998356

Tbl2	27368	-0.1699	0.998356
Dguok	27369	0.242477	0.998356
Rps26	27370	0.312947	0.998356
Csnk1e	27373	-0.13771	0.998356
Prmt5	27374	-0.06604	0.998356
Tjp3	27375	-0.10329	0.998356
Slc25a10	27376	0.130983	0.998356
Yme1l1	27377	0.006106	0.998356
Akr1c13	27384	0.373758	0.998356
Ptdss2	27388	-0.00335	0.998356
Pign	27392	0.266322	0.998356
Mrpl39	27393	0.301538	0.998356
Mrpl15	27395	-0.04429	0.998356
Mrpl2	27398	-0.13982	0.998356
Ip6k1	27399	0.195023	0.998356
Skp2	27401	-0.75464	0.998356
Pdhx	27402	-0.37276	0.998356
Abca7	27403	0.187724	0.998356
Abcf3	27406	-0.05724	0.998356
Abcf2	27407	0.608494	0.998356
Abcg5	27409	0.542401	0.998356
Abca3	27410	0.352818	0.998356
Sergef	27414	1.06662	0.998356
Abcc5	27416	0.752966	0.998356
Mkln1	27418	0.123002	0.998356
Naglu	27419	0.532226	0.998356
Atp5l	27425	0.128786	0.998356
Nagpa	27426	0.326745	0.998356
Shroom3	27428	-0.02197	0.998356
Amot	27494	-0.0465	0.998356
D0H4S114	27528	-1.11629	0.998356
Rdbp	27632	-0.48657	0.998356
Ubl4	27643	0.15664	0.998356
1700088E04Rik	27660	0.230838	0.998356
Snf8	27681	0.439859	0.998356
Lsm2	27756	-0.51553	0.998356
Commd8	27784	0.169597	0.998356
Zdhhc8	27801	0.070154	0.998356
Tada1	27878	0.341417	0.998356
D16H22S680E	27883	0.416715	0.998356
Dgcr14	27886	-0.43999	0.998356
Spg21	27965	0.02257	0.998356
Rrp9	27966	-0.44231	0.998356
Cherp	27967	-0.13784	0.998356
Vkorc1	27973	-0.81947	0.998356
Eif3b	27979	-0.24455	0.998356
D4Wsu53e	27981	-0.5485	0.998356

Efhd2	27984	0.474488	0.998356
Imp4	27993	-0.38777	0.998356
Exosc5	27998	-0.12193	0.998356
Fam3c	27999	0.341049	0.998356
Prpf19	28000	0.202903	0.998356
D6Wsu116e	28006	-0.08261	0.998356
Miip	28010	-0.23342	0.998356
Polr2m	28015	-3.04958	0.998356
Ubfd1	28018	-0.21531	0.998356
Ing4	28019	-0.07939	0.998356
Mrpl50	28028	-0.09708	0.998356
Gfm1	28030	-0.18908	0.998356
Usp39	28035	-0.06217	0.998356
Larp7	28036	-0.11014	0.998356
D6Wsu163e	28040	0.091581	0.998356
Ept1	28042	0.343264	0.998356
Yipf3	28064	-0.31446	0.998356
Twistnb	28071	0.065449	0.998356
Pppde2	28075	-0.3239	0.998356
Med10	28077	0.110917	0.998356
Atp5o	28080	0.009675	0.998356
D11Wsu99e	28081	-0.15743	0.998356
Vps25	28084	-0.0093	0.998356
D10Wsu52e	28088	0.097099	0.998356
Trim36	28105	-0.09584	0.998356
D17Wsu104e	28106	0.249873	0.998356
D10Wsu102e	28109	-0.23474	0.998356
Tinf2	28113	0.099346	0.998356
Nsun2	28114	-0.15195	0.998356
Cep63	28135	0.14598	0.998356
Serp1	28146	-0.2099	0.998356
Agpat3	28169	-0.15664	0.998356
Tomm70a	28185	-0.52623	0.998356
Reep3	28193	0.314016	0.998356
Dcaf11	28199	-0.11627	0.998356
Dhrs4	28200	-0.4249	0.998356
D10Jhu81e	28295	-0.00805	0.998356
Znhit2-ps	29805	-0.03306	0.998356
Limd1	29806	-0.0161	0.998356
Tpk1	29807	0.171008	0.998356
Mga	29808	-0.10538	0.998356
Rabgap1l	29809	-0.11173	0.998356
Bag3	29810	-0.3977	0.998356
Ndrg2	29811	-0.12397	0.998356
Ndrg3	29812	0.140249	0.998356
Bcar3	29815	0.361914	0.998356
Hip1r	29816	0.162966	0.998356

Igfbp7	29817	0.733117	0.998356
Stau2	29819	-0.16441	0.998356
Tnfrsf19	29820	0.197999	0.998356
Smtn	29856	-0.47941	0.998356
Rnf11	29864	0.132208	0.998356
Ulk2	29869	0.378407	0.998356
Gtse1	29870	-9.47192	0.998356
Scmh1	29871	-0.28933	0.998356
Iqgap1	29875	-0.03482	0.998356
Clic4	29876	0.027475	0.998356
Dnajc12	30045	0.005285	0.998356
Zfp292	30046	0.079346	0.998356
Fbxw2	30050	-0.39146	0.998356
Spdef	30051	-0.31196	0.998356
Timm13	30055	0.165946	0.998356
Timm9	30056	0.652941	0.998356
Timm8b	30057	0.513005	0.998356
Timm8a1	30058	-0.07412	0.998356
Timm10	30059	-0.22421	0.998356
Cttnbp2	30785	0.13497	0.998356
Slc39a1	30791	0.053879	0.998356
Pdlim4	30794	0.04229	0.998356
Fkbp3	30795	0.250835	0.998356
Fbxw4	30838	0.439766	0.998356
Fbxw5	30839	0.029088	0.998356
Fbxl6	30840	0.564871	0.998356
Kdm2b	30841	-0.2919	0.998356
Mlf2	30853	-0.2746	0.998356
Gnl3	30877	-0.42382	0.998356
Snai3	30927	-0.22951	0.998356
Zfp238	30928	0.078613	0.998356
Vps26a	30930	-0.06544	0.998356
Tor1a	30931	0.231766	0.998356
Zfp330	30932	0.275035	0.998356
Tor2a	30933	0.436446	0.998356
Tor1b	30934	0.23114	0.998356
Tor3a	30935	0.13584	0.998356
Lmcd1	30937	-0.9704	0.998356
Fgd3	30938	-0.10921	0.998356
Pttg1	30939	-0.1669	0.998356
Usp25	30940	0.311914	0.998356
Usp21	30941	-0.29932	0.998356
Hnf4g	30942	-0.14495	0.998356
Prss30	30943	0.235574	0.998356
Rnf19a	30945	0.379081	0.998356
Abt1	30946	-0.19566	0.998356
Bin1	30948	-0.86775	0.998356

Lcmt1	30949	0.230213	0.998356
Cbx8	30951	-0.29116	0.998356
Siva1	30954	-0.48982	0.998356
Pik3cg	30955	0.921514	0.998356
Mapk8ip3	30957	1.46779	0.998356
Vapa	30960	0.114927	0.998356
Ptpla	30963	-0.08493	0.998356
Thop1	50492	-0.28111	0.998356
Txnrd1	50493	-0.41889	0.998356
Hspa14	50497	0.137894	0.998356
Ercc4	50505	-0.12975	0.998356
Lats2	50523	0.141905	0.998356
Ero1l	50527	0.29268	0.998356
Tmprss2	50528	0.243558	0.998356
Mrps7	50529	-0.18001	0.998356
Postn	50706	0.380099	0.998356
Hist1h1c	50708	-0.07428	0.998356
Hist1h1e	50709	-0.60618	0.998356
Sirt6	50721	-0.76217	0.998356
Icosl	50723	0.411911	0.998356
Sap30l	50724	0.37596	0.998356
Fbxo8	50753	0.268313	0.998356
Fbxw7	50754	0.288887	0.998356
Fbxo18	50755	0.043291	0.998356
Fbxl17	50758	0.121423	0.998356
Fbxo6	50762	1.3989	0.998356
Crim1	50766	0.58236	0.998356
Pnpla6	50767	0.067494	0.998356
Atp8a2	50769	0.45598	0.998356
Atp11a	50770	0.0254	0.998356
Atp9b	50771	0.074171	0.998356
Mapk6	50772	-0.21419	0.998356
Nt5c	50773	0.577672	0.998356
Rgs1	50778	-0.36349	0.998356
Rgs3	50780	-0.16798	0.998356
Lsm4	50783	0.050849	0.998356
Ppap2c	50784	0.078879	0.998356
Hs6st1	50785	0.127044	0.998356
Fbxl8	50788	0.602397	0.998356
Fbxl3	50789	0.236704	0.998356
Acsl4	50790	0.62795	0.998356
Orc3	50793	-0.88598	0.998356
Klf13	50794	-0.06011	0.998356
Sh3bgr	50795	-0.83377	0.998356
Copb2	50797	-0.09377	0.998356
Gne	50798	0.120507	0.998356
Slc25a13	50799	-0.13319	0.998356

Solh	50817	-0.19571	0.998356
Rnf10	50849	0.051436	0.998356
Spast	50850	0.007032	0.998356
Keap1	50868	-0.18326	0.998356
Tmod3	50875	-0.11559	0.998356
Tmod2	50876	0.028904	0.998356
Scly	50880	-0.4164	0.998356
Chek2	50883	-0.20443	0.998356
Nckap1	50884	0.264209	0.998356
Hmgn5	50887	0.390174	0.998356
Preb	50907	-0.26352	0.998356
C1s	50908	0.304181	0.998356
Exosc9	50911	-0.0737	0.998356
Exosc10	50912	-0.26992	0.998356
Grb14	50915	0.244683	0.998356
Irx4	50916	-0.47781	0.998356
Galns	50917	-10	0.998356
Myadm	50918	-0.08912	0.998356
Hnrpdl	50926	0.04149	0.998356
Nasp	50927	-0.25898	0.998356
Mink1	50932	-0.13481	0.998356
Uchl3	50933	-0.11108	0.998356
Slc7a8	50934	0.260762	0.998356
St6galnac6	50935	0.363687	0.998356
Uba2	50995	0.140401	0.998356
Pdcd7	50996	-0.21431	0.998356
Cpsf2	51786	0.007975	0.998356
H2afz	51788	-0.36517	0.998356
Tnk2	51789	-0.04036	0.998356
Ppp2r1a	51792	0.16981	0.998356
Ddah2	51793	0.037302	0.998356
Srrm1	51796	-0.18055	0.998356
Ctps	51797	-0.12088	0.998356
Ech1	51798	-0.07123	0.998356
Bok	51800	0.289749	0.998356
Ramp1	51801	0.007337	0.998356
Hnrnpu	51810	-0.10823	0.998356
Mcrs1	51812	1.45982	0.998356
Ccnc	51813	0.22518	0.998356
Rif1	51869	-0.12148	0.998356
Tmem141	51875	0.360769	0.998356
Tubgcp4	51885	0.02407	0.998356
Fubp1	51886	-0.29304	0.998356
Atg13	51897	-0.28853	0.998356
Rnf24	51902	0.424978	0.998356
D2Ertd750e	51944	-0.27491	0.998356
Kctd18	51960	0.974425	0.998356

Cdk2ap2	52004	0.555657	0.998356
Hn1l	52009	-0.13759	0.998356
D19Ertd386e	52013	-0.16106	0.998356
Nus1	52014	-0.06509	0.998356
Pibf1	52023	-0.42795	0.998356
Ankrd22	52024	0.079008	0.998356
Pbk	52033	0.154918	0.998356
Ppp6r3	52036	0.389897	0.998356
Ppp1r10	52040	-0.43611	0.998356
Rab11fip5	52055	0.038528	0.998356
Coq5	52064	-0.02607	0.998356
Mfhas1	52065	-0.14647	0.998356
Pvr	52118	-0.10267	0.998356
Hgsnat	52120	0.393371	0.998356
Agpat5	52123	-0.21841	0.998356
Ccdc97	52132	-0.17428	0.998356
Kcnk6	52150	0.277654	0.998356
Camk1	52163	0.252827	0.998356
Tmem222	52174	0.291703	0.998356
Odf2l	52184	-2.20398	0.998356
Rbm34	52202	0.054467	0.998356
Anapc4	52206	0.078598	0.998356
Ankzf1	52231	-0.03015	0.998356
Commd2	52245	0.685423	0.998356
Cdca8	52276	-0.36597	0.998356
Klhl7	52323	0.479843	0.998356
Atxn1l	52335	-0.01694	0.998356
Vps37a	52348	-0.01443	0.998356
Rcn3	52377	0.100284	0.998356
D1Ertd622e	52392	0.133398	0.998356
Zfp644	52397	-0.00586	0.998356
Rhpn2	52428	0.520427	0.998356
Echdc2	52430	-0.22724	0.998356
Ppp2r2d	52432	0.279687	0.998356
Tax1bp1	52440	0.27397	0.998356
Mrpl48	52443	-0.41405	0.998356
Ctdsp2	52468	0.982391	0.998356
Ccdc56	52469	0.232839	0.998356
Angel2	52477	-0.19792	0.998356
Carhsp1	52502	-0.18444	0.998356
Cenpo	52504	-0.13675	0.998356
Ddx56	52513	-0.24822	0.998356
Zfp622	52521	0.033167	0.998356
Nhp2	52530	0.053219	0.998356
Mettl17	52535	-0.34341	0.998356
Acaa2	52538	-0.12089	0.998356
Sgta	52551	-0.23251	0.998356

Parp8	52552	-0.44639	0.998356
Cdc23	52563	0.068406	0.998356
Rg9mtd1	52575	-0.07393	0.998356
Dhrs1	52585	0.097587	0.998356
Tspan14	52588	-0.08713	0.998356
Ncald	52589	0.10427	0.998356
Brms1l	52592	0.251869	0.998356
Cbx7	52609	0.049766	0.998356
Suz12	52615	-1.18169	0.998356
Cdkn2aipnl	52626	0.066307	0.998356
Nit2	52633	-0.12847	0.998356
Esyt2	52635	0.253723	0.998356
Cisd1	52637	0.310194	0.998356
Wipi1	52639	0.225233	0.998356
Nudcd2	52653	-0.2662	0.998356
D18Ertd653e	52662	0.26045	0.998356
Echdc1	52665	-0.23839	0.998356
Ifi27l1	52668	-0.21138	0.998356
E2f7	52679	-0.33775	0.998356
Ncaph2	52683	0.13145	0.998356
Mettl2	52686	-0.17762	0.998356
Setd3	52690	0.035452	0.998356
Zwint	52696	-0.00201	0.998356
Txndc17	52700	0.329422	0.998356
Krr1	52705	-0.15408	0.998356
Zfp410	52708	-0.48458	0.998356
Slc52a2	52710	0.006652	0.998356
Zkscan6	52712	0.10287	0.998356
Ccdc59	52713	-0.51476	0.998356
Ccdc43	52715	-0.04522	0.998356
Anapc16	52717	0.142532	0.998356
Tspyl2	52808	-0.27204	0.998356
Ldhd	52815	-0.18147	0.998356
D4Bwg0951e	52829	0.325953	0.998356
Pnrc2	52830	0.02244	0.998356
Tmx4	52837	0.173442	0.998356
Dnlz	52838	1.08753	0.998356
Dbndd2	52840	1.22341	0.998356
D1Bwg0212e	52846	0.095696	0.998356
Sgsm1	52850	-0.26559	0.998356
Gtpbp5	52856	-0.04344	0.998356
Gramd1a	52857	-0.01173	0.998356
Cdipt	52858	-0.37819	0.998356
Slx4	52864	-0.25503	0.998356
D19Bwg1357e	52874	-0.19615	0.998356
Sco1	52892	0.107896	0.998356
Rnasek	52898	-0.00705	0.998356

Ahi1	52906	-0.03291	0.998356
Zmiz2	52915	0.297799	0.998356
Dlg3	53310	0.366049	0.998356
Nub1	53312	0.042895	0.998356
Atp2a3	53313	0.200591	0.998356
Sult1d1	53315	0.23698	0.998356
Plrg1	53317	0.231624	0.998356
Pdlim3	53318	0.081277	0.998356
Nxf1	53319	0.282851	0.998356
Nucb2	53322	0.540394	0.998356
Ube2k	53323	-0.0748	0.998356
Banp	53325	-0.53237	0.998356
Pgrmc1	53328	0.096555	0.998356
Vamp4	53330	-0.18819	0.998356
Stx7	53331	0.091611	0.998356
Mtmr1	53332	0.047018	0.998356
Tomm40	53333	0.833427	0.998356
Gosr1	53334	0.113729	0.998356
Eif3g	53356	-0.27494	0.998356
Pla2g6	53357	-0.99256	0.998356
Mtx2	53375	-0.34239	0.998356
Sdcbp	53378	-0.11978	0.998356
Hnrnpa2b1	53379	-0.19701	0.998356
Psmd10	53380	0.329027	0.998356
Prdx4	53381	0.283788	0.998356
Txnl1	53382	-0.07122	0.998356
Exoc7	53413	0.154687	0.998356
Bysl	53414	-0.19946	0.998356
Htatip2	53415	0.075041	0.998356
Stk39	53416	-0.43588	0.998356
Sec61a1	53421	0.156803	0.998356
Tsnax	53424	-0.19808	0.998356
Dctn3	53598	0.278591	0.998356
Cd164	53599	-0.00193	0.998356
Timm23	53600	0.584413	0.998356
Hpcal1	53602	-0.1969	0.998356
Nap1l1	53605	0.218108	0.998356
Snrpa	53607	-0.87394	0.998356
Clasrp	53609	-0.20719	0.998356
Nono	53610	-0.28308	0.998356
Vti1a	53611	0.154297	0.998356
Vti1b	53612	0.243198	0.998356
Fut8	53618	0.289138	0.998356
Blcap	53619	0.239981	0.998356
Cnot4	53621	-0.34242	0.998356
Cldn7	53624	1.34807	0.998356
B3gnt2	53625	0.236027	0.998356

Prrc2a	53761	-0.4855	0.998356
Ddx39b	53817	-0.15932	0.998356
Rwdd2b	53858	-0.1326	0.998356
Map3k14	53859	-0.26194	0.998356
Zranb2	53861	-0.29947	0.998356
Col5a3	53867	-0.19339	0.998356
Rab25	53868	-0.04981	0.998356
Rab11a	53869	0.050476	0.998356
Caprin1	53872	-0.12633	0.998356
Naip7	53880	0.628414	0.998356
Slc5a3	53881	-0.04169	0.998356
Cdkl2	53886	0.119063	0.998356
Sart3	53890	-0.23469	0.998356
Ppm1d	53892	0.219653	0.998356
Nudt5	53893	-0.29671	0.998356
Clpp	53895	-0.06955	0.998356
Rcan2	53901	0.227464	0.998356
Rcan3	53902	0.447685	0.998356
Slc40a1	53945	0.256466	0.998356
Ccdc75	53951	-0.30209	0.998356
Rfx5	53970	-0.27657	0.998356
Ngef	53972	0.169051	0.998356
Ddx20	53975	0.041131	0.998356
Diap2	54004	-0.0065	0.998356
Deaf1	54006	-0.01692	0.998356
Uevld	54122	0.298713	0.998356
Cks1b	54124	0.149032	0.998356
Polm	54125	0.00404	0.998356
Arhgef7	54126	-0.03952	0.998356
Pmm2	54128	0.005657	0.998356
Actr1a	54130	0.116117	0.998356
Irf3	54131	-0.18296	0.998356
Pdlim1	54132	0.215864	0.998356
Lsr	54135	0.105003	0.998356
Atxn10	54138	-0.19034	0.998356
Irf6	54139	0.064425	0.998356
Avpr1a	54140	0.282244	0.998356
Spag5	54141	0.123391	0.998356
Cyhr1	54151	0.531259	0.998356
Dnalc4	54152	0.159662	0.998356
Copg2	54160	0.178659	0.998356
Copg	54161	0.239875	0.998356
Myst4	54169	-0.67675	0.998356
Rragc	54170	0.472271	0.998356
Cpsf4	54188	-0.30402	0.998356
Rabep1	54189	-0.2499	0.998356
Akap8l	54194	0.282486	0.998356

Pabpn1	54196	-0.05983	0.998356
Rnf5	54197	-0.38143	0.998356
Snx3	54198	-0.31953	0.998356
Sult2b1	54200	-0.31161	0.998356
Arl6ip1	54208	-0.00547	0.998356
Golga4	54214	0.055418	0.998356
Pcdh7	54216	0.183315	0.998356
B3galt4	54218	0.226462	0.998356
Cd320	54219	-0.08429	0.998356
Arhgef5	54324	0.107606	0.998356
Elovl1	54325	0.823379	0.998356
Slc23a2	54338	0.334183	0.998356
Gnpnat1	54342	-0.14827	0.998356
Atf7ip	54343	-0.41613	0.998356
Rai12	54351	-0.34977	0.998356
Irx5	54352	0.521323	0.998356
Skap2	54353	-0.01487	0.998356
Rpp30	54364	0.552802	0.998356
Ctnnal1	54366	-0.10244	0.998356
Zfp326	54367	-0.27876	0.998356
Nme6	54369	0.04579	0.998356
Azin1	54375	0.272148	0.998356
Pgcp	54381	0.141022	0.998356
Phc2	54383	0.647523	0.998356
Mtmr7	54384	0.795496	0.998356
Mcm3ap	54387	-0.1701	0.998356
Rfk	54391	0.490047	0.998356
Ncapg	54392	-0.29596	0.998356
Crlf3	54394	-0.02065	0.998356
Irgm2	54396	0.277445	0.998356
Ppt2	54397	0.071595	0.998356
Bet1l	54399	-0.1411	0.998356
Ywhab	54401	-0.08269	0.998356
Stk19	54402	0.488658	0.998356
Slc4a4	54403	-0.15588	0.998356
Ndufa1	54405	0.297412	0.998356
Ramp2	54409	0.136075	0.998356
Atp6ap1	54411	0.037865	0.998356
Cldn6	54419	-0.18194	0.998356
Cldn8	54420	-0.40069	0.998356
Hgfac	54426	0.017329	0.998356
Unc93b1	54445	0.732293	0.998356
Nfat5	54446	-0.04403	0.998356
Cpsf3	54451	0.124693	0.998356
Tollip	54473	0.147454	0.998356
Mkrn1	54484	0.294665	0.998356
Dll4	54485	-0.12133	0.998356

Hpgds	54486	-0.32942	0.998356
Nup210	54563	0.016688	0.998356
Foxo4	54601	-0.05791	0.998356
Pcnx	54604	0.171973	0.998356
Socs6	54607	0.168816	0.998356
Abhd2	54608	0.148417	0.998356
Ubqln2	54609	0.007067	0.998356
Tbc1d8	54610	0.195353	0.998356
Pde3a	54611	-0.15874	0.998356
St3gal6	54613	0.149435	0.998356
Prpf40b	54614	-0.59121	0.998356
Extl3	54616	0.094857	0.998356
Paf1	54624	0.092734	0.998356
Prickle3	54630	0.128457	0.998356
Ftsj1	54632	-0.01716	0.998356
Pqbp1	54633	-0.66518	0.998356
Pdgfc	54635	0.339885	0.998356
Wdr45	54636	0.087753	0.998356
Praf2	54637	-0.28674	0.998356
Ccdc22	54638	0.114325	0.998356
Otud5	54644	0.176348	0.998356
Gripap1	54645	-0.29954	0.998356
Ccdc120	54648	0.566877	0.998356
Sfmbt1	54650	0.153686	0.998356
Atp8b2	54667	-0.09814	0.998356
Atp8b1	54670	0.134088	0.998356
Sh3glb1	54673	0.36816	0.998356
Prdx5	54683	0.075575	0.998356
Eif3i	54709	0.188555	0.998356
Hs3st3b1	54710	0.200753	0.998356
Plagl2	54711	-0.01715	0.998356
Rcan1	54720	0.444239	0.998356
Tyk2	54721	-0.05572	0.998356
Tfip11	54723	0.157944	0.998356
Cadm1	54725	-0.20786	0.998356
Syt8	55925	0.574833	0.998356
Hes6	55927	0.461544	0.998356
Gbp3	55932	-0.34147	0.998356
Rp9	55934	0.508681	0.998356
Fnbp4	55935	-0.03673	0.998356
Ctps2	55936	-0.27335	0.998356
Sertad1	55942	0.531934	0.998356
Stx8	55943	-0.17115	0.998356
Eif3d	55944	-0.10409	0.998356
Ap3m1	55946	0.19664	0.998356
Dclre1a	55947	-0.06943	0.998356
Sfn	55948	-0.17666	0.998356

Eef1b2	55949	0.11547	0.998356
Bri3	55950	1.67979	0.998356
Brp44l	55951	-0.20299	0.998356
Ebag9	55960	0.387097	0.998356
Slc1a4	55963	0.174683	0.998356
Ift20	55978	0.141796	0.998356
Agpat1	55979	-0.1692	0.998356
Impa1	55980	-0.09927	0.998356
Pigb	55981	0.052777	0.998356
Paxip1	55982	0.099028	0.998356
Pdzrn3	55983	-0.05975	0.998356
Cpxm2	55987	-0.22434	0.998356
Snx12	55988	-0.17248	0.998356
Nop58	55989	-0.45887	0.998356
Panx1	55991	-0.22166	0.998356
Trim3	55992	0.278468	0.998356
Alyref2	56009	0.404436	0.998356
Hebp2	56016	-0.09226	0.998356
Stard10	56018	0.30388	0.998356
Tmem131	56030	0.360265	0.998356
Ppie	56031	0.269523	0.998356
Nprl2	56032	0.022482	0.998356
Ccnl2	56036	-0.11872	0.998356
Rplp1	56040	0.268905	0.998356
Uso1	56041	0.009803	0.998356
Akr1e1	56043	0.037908	0.998356
Rala	56044	0.155795	0.998356
Samhd1	56045	0.218943	0.998356
Uqcc	56046	-0.07802	0.998356
Lgals8	56048	0.57914	0.998356
Cyp39a1	56050	-0.46823	0.998356
Ammecr1	56068	-0.27488	0.998356
Tcerg1	56070	0.027679	0.998356
Lgals12	56072	-0.5252	0.998356
Pdss1	56075	-0.08684	0.998356
Dgke	56077	0.068693	0.998356
Ubqln1	56085	0.434501	0.998356
Set	56086	0.22867	0.998356
Psmg1	56088	0.063165	0.998356
Ramp3	56089	0.209092	0.998356
Ftsj3	56095	-0.37202	0.998356
Grasp	56149	0.183304	0.998356
Mad2l1	56150	0.066359	0.998356
Nagk	56174	0.017899	0.998356
Bace2	56175	0.288173	0.998356
Pigp	56176	0.127434	0.998356
Rabggta	56187	-0.12824	0.998356

Rbm38	56190	-0.47569	0.998356
Plek	56193	-0.17228	0.998356
Prpf40a	56194	0.047638	0.998356
Ptbp2	56195	0.223767	0.998356
Tdp2	56196	-0.03697	0.998356
Abcb10	56199	0.116256	0.998356
Ddx21	56200	-0.15837	0.998356
Ensa	56205	0.18243	0.998356
Uchl5	56207	-0.24575	0.998356
Becn1	56208	0.288392	0.998356
Gde1	56209	0.362285	0.998356
Rev1	56210	-0.07956	0.998356
Rhog	56212	0.233496	0.998356
Htra1	56213	0.28799	0.998356
Scamp4	56214	-0.30866	0.998356
Acin1	56215	0.835545	0.998356
Mpp5	56217	0.158293	0.998356
Patz1	56218	-0.21743	0.998356
Extl1	56219	0.615764	0.998356
Zfp386	56220	0.16048	0.998356
Cited4	56222	-0.01951	0.998356
Tspan5	56224	0.090389	0.998356
Espn	56226	0.013975	0.998356
Ube2j1	56228	0.062407	0.998356
Thsd1	56229	-0.73764	0.998356
Ak3	56248	0.24372	0.998356
Actr8	56249	0.32912	0.998356
Hnrnph2	56258	-0.06709	0.998356
Pex14	56273	-0.06932	0.998356
Stk3	56274	-0.23725	0.998356
Rbm14	56275	-0.39201	0.998356
Mrpl37	56280	0.34679	0.998356
Mrpl12	56282	-0.10389	0.998356
Mrpl19	56284	0.094208	0.998356
Rassf1	56289	0.175131	0.998356
Naa10	56292	-0.08047	0.998356
Ptpn9	56294	0.224451	0.998356
Arl6	56297	0.437908	0.998356
Atl2	56298	0.251587	0.998356
Fkbpl	56299	0.117443	0.998356
Pitpnb	56305	-0.29323	0.998356
Fam60a	56306	0.166484	0.998356
Metap2	56307	-0.08663	0.998356
Mycbp	56309	0.126884	0.998356
Gps2	56310	-0.2267	0.998356
Nupr1	56312	-0.52129	0.998356
Zfp113	56314	-0.24326	0.998356

Ggcx	56316	-0.07798	0.998356
Anapc7	56317	0.008297	0.998356
Acpp	56318	0.25003	0.998356
Dbn1	56320	-0.78464	0.998356
Aatf	56321	-0.46155	0.998356
Timm22	56322	-0.18517	0.998356
Dnajb5	56323	-0.13119	0.998356
Stam2	56324	0.424622	0.998356
Abcb9	56325	0.046251	0.998356
Arl2	56327	-0.3475	0.998356
Pdcd5	56330	-0.02258	0.998356
Amotl2	56332	-0.51353	0.998356
Tmed2	56334	0.005687	0.998356
Mettl3	56335	-0.24162	0.998356
Txnip	56338	0.298361	0.998356
Eif3c	56347	-0.07803	0.998356
Hsd17b12	56348	-0.15502	0.998356
Net1	56349	0.213099	0.998356
Arl3	56350	0.008672	0.998356
Ptges3	56351	0.1773	0.998356
Rybp	56353	-0.25459	0.998356
Dnajc7	56354	-0.29974	0.998356
Gltp	56356	-0.15196	0.998356
Ivd	56357	-0.22195	0.998356
Copz2	56358	-1.40918	0.998356
Acot9	56360	0.089708	0.998356
Pus1	56361	-1.37731	0.998356
Zmym3	56364	-0.14794	0.998356
Scoc	56367	-1.25172	0.998356
Cyb561d2	56368	-0.08454	0.998356
Apip	56369	-0.496	0.998356
Fzr1	56371	0.114141	0.998356
1110004F10Rik	56372	0.096277	0.998356
Tmem59	56374	0.429153	0.998356
B4galt4	56375	-0.28489	0.998356
Pdlim5	56376	0.112179	0.998356
Arpc3	56378	-0.15254	0.998356
Arid3b	56380	-0.22842	0.998356
Spen	56381	-0.17249	0.998356
Rab9	56382	-0.09694	0.998356
Letm1	56384	0.026851	0.998356
B4galt6	56386	0.081397	0.998356
Stx5a	56389	0.217075	0.998356
Sssca1	56390	-0.15228	0.998356
Shoc2	56392	-2.57071	0.998356
Tmem115	56395	-0.13823	0.998356
Morf4l2	56397	-1.92363	0.998356

1500003O03Rik	56398	0.268738	0.998356
Akap8	56399	-0.11028	0.998356
Syncrip	56403	-0.41318	0.998356
Trip4	56404	-0.10643	0.998356
Dusp14	56405	0.45789	0.998356
Ncoa6	56406	0.222996	0.998356
Trpc4ap	56407	0.274097	0.998356
Nudt3	56409	0.046282	0.998356
Noa1	56412	0.174926	0.998356
Adar	56417	0.646738	0.998356
Ykt6	56418	-0.01942	0.998356
Diap3	56419	-0.41935	0.998356
Ppp4c	56420	0.112967	0.998356
Pfkp	56421	-0.04506	0.998356
Hbs1l	56422	-0.05168	0.998356
Stub1	56424	0.073356	0.998356
Tubd1	56427	0.803882	0.998356
Mtch2	56428	-0.07597	0.998356
Dpt	56429	0.321056	0.998356
Clip1	56430	-0.18973	0.998356
Dstn	56431	-0.11995	0.998356
Tspan3	56434	0.148421	0.998356
Adrm1	56436	-0.5652	0.998356
Rbx1	56438	0.31861	0.998356
Snx1	56440	0.402968	0.998356
Nat6	56441	0.099165	0.998356
Serinc1	56442	0.258327	0.998356
Arpc1a	56443	0.256397	0.998356
Actr10	56444	0.019469	0.998356
Dnaja2	56445	0.177226	0.998356
Copz1	56447	-0.30414	0.998356
Csda	56449	0.17596	0.998356
Suclg1	56451	-0.04125	0.998356
Orc6	56452	-1.43035	0.998356
Mbtps1	56453	0.091973	0.998356
Aldh18a1	56454	-0.7479	0.998356
Dynll1	56455	-0.24352	0.998356
Actl6a	56456	-0.17547	0.998356
Clptm1	56457	0.436055	0.998356
Foxo1	56458	-0.20472	0.998356
Sae1	56459	-0.10714	0.998356
Pkp3	56460	-0.17807	0.998356
Mtch1	56462	0.261271	0.998356
Snd1	56463	-0.14873	0.998356
Ctsf	56464	-0.03482	0.998356
Socs5	56468	-0.14171	0.998356
Rgs19	56470	-0.17394	0.998356

Fads2	56473	-0.21733	0.998356
Tbk1	56480	0.163768	0.998356
Foxo3	56484	0.089887	0.998356
Gabarap	56486	0.257682	0.998356
Nxt1	56488	0.138719	0.998356
Ikbke	56489	-0.07995	0.998356
Zbtb20	56490	-0.47042	0.998356
Vapb	56491	0.180986	0.998356
Gosr2	56494	-0.07693	0.998356
Asna1	56495	0.209764	0.998356
Elf4	56501	-0.06396	0.998356
Ankrd49	56503	0.037767	0.998356
Ruvbl1	56505	-0.4429	0.998356
Cib2	56506	0.189321	0.998356
Rnf138	56515	-0.07598	0.998356
Rbms2	56516	0.182254	0.998356
Nme4	56520	0.518567	0.998356
Mpp6	56524	0.267045	0.998356
Zfp235	56525	0.191523	0.998356
Sec11a	56529	0.079423	0.998356
Cnpy2	56530	-0.14125	0.998356
Ylpm1	56531	-0.03602	0.998356
Ripk3	56532	-0.46396	0.998356
Pex3	56535	-0.99609	0.998356
Habp4	56541	0.228605	0.998356
Ick	56542	-0.05552	0.998356
Ube2d2	56550	0.154154	0.998356
Txn2	56551	-0.00703	0.998356
Pfdn5	56612	0.153418	0.998356
Rps6ka4	56613	0.092915	0.998356
Mgst1	56615	0.397462	0.998356
Clec4n	56620	-0.40641	0.998356
Poll	56626	-0.39222	0.998356
Sphk2	56632	-0.34743	0.998356
Gsk3b	56637	0.011025	0.998356
Slc15a1	56643	0.700825	0.998356
Clec7a	56644	0.423817	0.998356
Mlycd	56690	0.92536	0.998356
Lamtor3	56692	0.472437	0.998356
Crtap	56693	-0.22416	0.998356
Pnkd	56695	-0.57418	0.998356
Akap10	56697	0.156489	0.998356
Phax	56698	-0.3376	0.998356
Cdc42ep4	56699	0.031216	0.998356
0610031J06Rik	56700	0.035708	0.998356
Hist1h1b	56702	-0.40619	0.998356
Pigo	56703	-0.0083	0.998356

Ranbp9	56705	0.598688	0.998356
Ccnl1	56706	0.207775	0.998356
Zfp111	56707	-0.29327	0.998356
Clcf1	56708	-0.47391	0.998356
Dnajb12	56709	0.127088	0.998356
Rabgef1	56715	0.021403	0.998356
Mlst8	56716	1.47216	0.998356
Mtor	56717	0.301086	0.998356
Litaf	56722	0.533674	0.998356
Cript	56724	0.433231	0.998356
Sh3bgrl	56726	-0.04584	0.998356
Rnf14	56736	0.189686	0.998356
Alg2	56737	-0.17751	0.998356
Mocs1	56738	0.013013	0.998356
Rec8	56739	0.124143	0.998356
Psrc1	56742	-0.62965	0.998356
Pf4	56744	-0.31784	0.998356
Nfu1	56748	-1.75257	0.998356
Dhodh	56749	-0.00272	0.998356
Aldh9a1	56752	-0.38858	0.998356
Tacstd2	56753	0.595114	0.998356
Mbnl1	56758	1.91584	0.998356
Med20	56771	0.436376	0.998356
Mllt11	56772	-0.19389	0.998356
Slc6a14	56774	-0.02799	0.998356
Fam48a	56790	0.048714	0.998356
Ube2l6	56791	-0.09928	0.998356
Arl10	56795	-0.68074	0.998356
Zbtb33	56805	-0.46061	0.998356
Scamp5	56807	0.181082	0.998356
Dkk2	56811	0.184404	0.998356
Dnajb2	56812	-0.67607	0.998356
Ccl28	56838	-0.00803	0.998356
Tssc4	56844	0.255657	0.998356
Aldh1a3	56847	0.57547	0.998356
Zfp109	56869	0.037155	0.998356
Lmbr1	56873	0.156945	0.998356
Rnf32	56874	0.16415	0.998356
Nelf	56876	1.46183	0.998356
Rbms1	56878	0.219169	0.998356
Gtf2ird1	57080	-0.22098	0.998356
Dolpp1	57170	0.277261	0.998356
Sap30bp	57230	-0.17886	0.998356
Zfp276	57247	0.413368	0.998356
Vav3	57257	-1.8158	0.998356
Xpo4	57258	0.003259	0.998356
Tob2	57259	-0.1638	0.998356

Brd4	57261	0.33605	0.998356
Retnlb	57263	0.491088	0.998356
Apba3	57267	-0.12376	0.998356
Bcam	57278	-0.21503	0.998356
Rps27	57294	-0.17592	0.998356
Icmt	57295	0.113191	0.998356
Psmd8	57296	0.114788	0.998356
Mrps31	57312	-0.27427	0.998356
Th1l	57314	0.173766	0.998356
Wdr46	57315	-0.01898	0.998356
C1d	57316	-0.12828	0.998356
Srsf4	57317	-0.07209	0.998356
Park7	57320	-0.4158	0.998356
Terf2ip	57321	0.090198	0.998356
Gigyf1	57330	0.142597	0.998356
Parva	57342	0.407751	0.998356
As3mt	57344	0.196739	0.998356
Cramp1l	57354	-0.10379	0.998356
Srd5a3	57357	0.264409	0.998356
B4galt3	57370	0.01779	0.998356
D930014E17Rik	57373	-0.12855	0.998356
Smarce1	57376	0.050338	0.998356
Mogs	57377	-0.00067	0.998356
Psors1c2	57390	1.79922	0.998356
Atp5j2	57423	0.568457	0.998356
U90926	57425	-1.16618	0.998356
Dnajc4	57431	0.027232	0.998356
Zc3h8	57432	-0.01353	0.998356
Xrcc2	57434	-0.12122	0.998356
Gabarapl1	57436	-0.29362	0.998356
Golga7	57437	0.237681	0.998356
Tmem183a	57439	-0.19843	0.998356
Ehd3	57440	-0.15129	0.998356
Gmnn	57441	-0.15726	0.998356
Kcne3	57442	-0.55666	0.998356
Fbxo3	57443	-0.13308	0.998356
Isg20	57444	-0.01147	0.998356
Noc2l	57741	-0.15287	0.998356
Sec61a2	57743	0.289006	0.998356
Jmy	57748	-0.00513	0.998356
Wdr12	57750	-0.96721	0.998356
Rnf25	57751	0.405778	0.998356
Tacc2	57752	0.103965	0.998356
Noc3l	57753	-0.43648	0.998356
Wdr4	57773	0.092504	0.998356
Ttyh1	57776	0.140285	0.998356
Tnip1	57783	-0.41579	0.998356

Bin3	57784	-0.03417	0.998356
Rangrf	57785	1.03961	0.998356
Rpl35a	57808	-1.05151	0.998356
Tk2	57813	0.332677	0.998356
Spata5	57815	0.019682	0.998356
Tesc	57816	0.255261	0.998356
Eral1	57837	-0.1855	0.998356
Adck2	57869	0.053411	0.998356
Ptplad1	57874	0.035176	0.998356
Krcc1	57896	0.071914	0.998356
Isy1	57905	0.202678	0.998356
Zfp318	57908	0.087149	0.998356
Gsdma	57911	-0.15769	0.998356
Cdc42se1	57912	0.055497	0.998356
Lrdd	57913	-0.68454	0.998356
Tbc1d1	57915	-0.17305	0.998356
Sertad2	58172	0.074756	0.998356
Rqcd1	58184	-0.13435	0.998356
Rsad2	58185	0.761983	0.998356
Rad18	58186	0.112687	0.998356
Extl2	58193	0.444076	0.998356
Sh3kbp1	58194	-0.28019	0.998356
Cobra1	58202	0.227023	0.998356
Zbp1	58203	0.255255	0.998356
Bcl11b	58208	-0.47765	0.998356
Sectm1b	58210	0.075628	0.998356
Trem1	58217	-0.32404	0.998356
Pard6b	58220	0.556416	0.998356
Mmp19	58223	-0.35303	0.998356
Rnf8	58230	0.035198	0.998356
Stk4	58231	0.167368	0.998356
Dnaja4	58233	-0.11088	0.998356
Hs1bp3	58240	-0.7231	0.998356
Stx6	58244	-0.04695	0.998356
Gpr180	58245	-0.06517	0.998356
Slc35b4	58246	0.108748	0.998356
1700123O20Rik	58248	0.015942	0.998356
Fibp	58249	0.884423	0.998356
Chst11	58250	-0.08762	0.998356
0610007P14Rik	58520	0.237957	0.998356
Eid1	58521	0.071702	0.998356
Elp2	58523	0.372884	0.998356
Crbn	58799	0.23118	0.998356
Trpm7	58800	-1.1083	0.998356
Pmaip1	58801	0.134825	0.998356
Kcnmb4	58802	0.024459	0.998356
Cdc42ep5	58804	0.408448	0.998356

Rnase4	58809	-0.30558	0.998356
Akr1a1	58810	0.088623	0.998356
Efemp2	58859	0.091825	0.998356
Adamdec1	58860	0.50069	0.998356
Hibadh	58875	-0.13292	0.998356
Repin1	58887	1.59907	0.998356
Fam13a	58909	0.271144	0.998356
Sumf1	58911	0.241303	0.998356
Rps6kb2	58988	0.181206	0.998356
Smpd3	58994	0.020847	0.998356
Pole3	59001	0.195836	0.998356
Wdr8	59002	-0.13532	0.998356
Maea	59003	-0.12341	0.998356
Pias4	59004	-0.46282	0.998356
Trappc2l	59005	0.53159	0.998356
Ngly1	59007	0.198893	0.998356
Anapc5	59008	-0.34558	0.998356
Sh3rf1	59009	0.06667	0.998356
Sqrdl	59010	-0.33766	0.998356
Moxd1	59012	-0.50146	0.998356
Hnrnph1	59013	-0.10505	0.998356
Rrs1	59014	-0.22192	0.998356
Nup160	59015	-0.30021	0.998356
Thap11	59016	-0.15625	0.998356
Rab2a	59021	0.129948	0.998356
Edf1	59022	-0.1492	0.998356
Med12	59024	0.057586	0.998356
Usp14	59025	-0.14669	0.998356
Huwe1	59026	-0.02273	0.998356
Nampt	59027	0.261102	0.998356
Rcl1	59028	-0.37081	0.998356
Psmd14	59029	0.060307	0.998356
Mkks	59030	-0.0139	0.998356
Chst12	59031	-0.4391	0.998356
Ppp2r3c	59032	-0.05535	0.998356
Carm1	59035	-0.20018	0.998356
Pxmp4	59038	0.40348	0.998356
Rhot1	59040	-0.0893	0.998356
Stk25	59041	0.589791	0.998356
Cope	59042	-0.26965	0.998356
Wsb2	59043	0.450765	0.998356
Rnf130	59044	0.381737	0.998356
Stard3	59045	0.285749	0.998356
Arpp19	59046	-0.42536	0.998356
Pnkp	59047	-0.18825	0.998356
C1galt1c1	59048	0.074618	0.998356
Nsa2	59050	-0.06942	0.998356

Mettl9	59052	0.360393	0.998356
Fam203a	59053	-0.3928	0.998356
Mrps30	59054	0.101465	0.998356
Zfp191	59057	-0.1638	0.998356
Tpm3	59069	-0.20008	0.998356
Erbb2ip	59079	0.396623	0.998356
Fetub	59083	1.82612	0.998356
Midn	59090	-0.11989	0.998356
Pcbp4	59092	0.254863	0.998356
Fxyd6	59095	-0.16993	0.998356
Nek7	59125	0.358309	0.998356
Nek6	59126	-4.34788	0.998356
Ncstn	59287	0.18004	0.998356
Dctn5	59288	-0.23341	0.998356
Gpa33	59290	0.146087	0.998356
Myg1	60315	0.245279	0.998356
Wbp11	60321	-0.50532	0.998356
Cldn15	60363	-0.39684	0.998356
Donson	60364	0.703259	0.998356
Sap30	60406	0.085274	0.998356
Trappc4	60409	0.384229	0.998356
Cenpk	60411	0.088826	0.998356
Mrpl38	60441	-0.34426	0.998356
Tmem8	60455	0.279505	0.998356
Qtrt1	60507	-0.24955	0.998356
Acss2	60525	-0.53006	0.998356
Fads3	60527	-0.07858	0.998356
Fignl1	60530	-0.35052	0.998356
Wtap	60532	0.341596	0.998356
Cd274	60533	-0.17759	0.998356
Actn4	60595	0.154795	0.998356
Trp53inp1	60599	-0.12167	0.998356
Foxj2	60611	0.066683	0.998356
Kcnq1ot1	63830	0.092996	0.998356
Taf8	63856	-0.36438	0.998356
Fam129a	63913	0.107577	0.998356
Dusp10	63953	0.226691	0.998356
Ube4b	63958	0.006242	0.998356
Slc29a1	63959	-0.02346	0.998356
Gmfb	63985	-0.19918	0.998356
Gmfg	63986	0.023528	0.998356
Syne1	64009	0.200419	0.998356
Sav1	64010	0.031074	0.998356
Yeats4	64050	0.299517	0.998356
Perp	64058	0.640383	0.998356
Smoc2	64074	0.060848	0.998356
Smoc1	64075	0.876693	0.998356

Clstn2	64085	0.31417	0.998356
Gpr35	64095	-0.49724	0.998356
Sdf2l1	64136	-0.07508	0.998356
Ctsz	64138	-0.09685	0.998356
Ralb	64143	0.175736	0.998356
Mllt1	64144	0.218225	0.998356
Trpv6	64177	-0.21026	0.998356
Herpud1	64209	-0.19195	0.998356
St7	64213	0.445173	0.998356
Ptges	64292	0.339866	0.998356
Itm2c	64294	0.294156	0.998356
Tmub1	64295	0.663779	0.998356
Abhd8	64296	0.03254	0.998356
Dhx38	64340	0.081235	0.998356
Sirt2	64383	0.121893	0.998356
Sp5	64406	0.156891	0.998356
Polr1e	64424	-0.65356	0.998356
Inpp5e	64436	0.210704	0.998356
Dip2a	64451	0.256368	0.998356
Zfp280b	64453	-0.34125	0.998356
Tspan4	64540	1.03639	0.998356
Ireb2	64602	0.054555	0.998356
Nisch	64652	-0.12414	0.998356
Mrps22	64655	0.546968	0.998356
Mrps23	64656	-0.13123	0.998356
Mrps10	64657	0.789771	0.998356
Mrps25	64658	-0.3567	0.998356
Mrps14	64659	-0.26148	0.998356
Mrps24	64660	-0.31613	0.998356
Krtdap	64661	0.075488	0.998356
Nmi	64685	0.085793	0.998356
Htra2	64704	-0.19141	0.998356
Scube1	64706	0.645085	0.998356
Svep1	64817	0.536231	0.998356
Krt81	64818	5.51822	0.998356
Lpin2	64898	0.272557	0.998356
Lpin3	64899	0.499169	0.998356
Tsc1	64930	-0.19565	0.998356
Pes1	64934	-0.22868	0.998356
Cldn12	64945	0.381646	0.998356
Rpl23	65019	-0.19787	0.998356
Zfp110	65020	-0.15832	0.998356
Zfand6	65098	0.005079	0.998356
Nif3l1	65102	-0.33107	0.998356
Arl6ip6	65103	-0.28926	0.998356
Arl6ip4	65105	-0.33526	0.998356
Arl6ip5	65106	0.195323	0.998356

Lrp10	65107	0.390089	0.998356
Dap3	65111	0.041929	0.998356
Pmepa1	65112	0.245233	0.998356
Ndfip1	65113	0.17675	0.998356
Vps35	65114	0.063917	0.998356
Prrg2	65116	-0.05621	0.998356
Slc15a3	65221	0.118848	0.998356
Xpo7	65246	0.029469	0.998356
Asb3	65257	-0.26378	0.998356
Clstn1	65945	0.053381	0.998356
Twsg1	65960	0.08778	0.998356
Utp3	65961	-0.47549	0.998356
Slc9a3r2	65962	-0.19691	0.998356
Tmem176b	65963	0.115416	0.998356
B230120H23Rik	65964	-0.3075	0.998356
Eefsec	65967	-0.24321	0.998356
Cubn	65969	-0.04521	0.998356
Lima1	65970	0.131396	0.998356
Asph	65973	-1.2285	0.998356
Atp5d	66043	0.250632	0.998356
Dtd1	66044	-0.0444	0.998356
Ndufb5	66046	-0.26475	0.998356
Tmem93	66048	-1.21673	0.998356
Rogdi	66049	-0.01248	0.998356
0610009B22Rik	66050	0.089157	0.998356
Sdhc	66052	0.30221	0.998356
Ppil2	66053	0.262262	0.998356
Cndp2	66054	-0.12694	0.998356
0610009D07Rik	66055	0.416245	0.998356
Zfp524	66056	-0.05215	0.998356
Tmem176a	66058	0.952522	0.998356
Krtcap2	66059	-0.14049	0.998356
0610010O12Rik	66060	-0.43689	0.998356
Tctex1d2	66061	0.535964	0.998356
Gng11	66066	0.294352	0.998356
Gtpbp8	66067	0.462857	0.998356
Snupn	66069	-0.09838	0.998356
Cwc15	66070	0.255639	0.998356
Ethe1	66071	0.442162	0.998356
Sdhaf2	66072	-0.05876	0.998356
Txndc12	66073	-0.15743	0.998356
Tmem167	66074	0.025206	0.998356
Chchd3	66075	0.042135	0.998356
Aurkaip1	66077	-0.06469	0.998356
Tsen34	66078	0.122436	0.998356
Tmem42	66079	-2.40066	0.998356
Abhd6	66082	-0.08642	0.998356

Setd6	66083	-0.23302	0.998356
Rmnd1	66084	-0.09449	0.998356
Eif3f	66085	0.330366	0.998356
0610037P05Rik	66086	0.625454	0.998356
Tmem111	66087	0.420379	0.998356
Rmnd5b	66089	0.139369	0.998356
Ypel3	66090	0.216964	0.998356
Ndufa3	66091	-0.77145	0.998356
Ghitm	66092	0.417327	0.998356
Lsm7	66094	-0.77813	0.998356
0910001L09Rik	66096	-0.46645	0.998356
Chchd6	66098	-0.82741	0.998356
Ppih	66101	0.53801	0.998356
Cxcl16	66102	0.421127	0.998356
Ube2d3	66105	0.207934	0.998356
Smpx	66106	0.349097	0.998356
Ndufa9	66108	0.052668	0.998356
Tspan13	66109	-0.36046	0.998356
Tmed3	66111	0.019379	0.998356
Dnajc30	66114	0.046894	0.998356
1110001J03Rik	66117	0.466429	0.998356
Sarnp	66118	0.347396	0.998356
Tomm6	66119	-0.21078	0.998356
Fkbp11	66120	-0.28015	0.998356
Chchd1	66121	0.573682	0.998356
1110006O24Rik	66123	0.151085	0.998356
Josd2	66124	-0.37111	0.998356
Sf3b5	66125	0.257816	0.998356
Elof1	66126	0.434884	0.998356
Mrps36	66128	0.54894	0.998356
1110018J18Rik	66129	-0.09862	0.998356
Tipin	66131	-0.17296	0.998356
1110008L16Rik	66132	-0.36557	0.998356
Znrd1	66136	-0.01213	0.998356
Wbscr22	66138	-0.00027	0.998356
Fam33a	66140	-0.17782	0.998356
Ifitm3	66141	-0.16681	0.998356
Cox7b	66142	0.381051	0.998356
Eef1e1	66143	0.116443	0.998356
Atp6v1f	66144	0.129052	0.998356
Tmem57	66146	0.142283	0.998356
Necap2	66147	0.461436	0.998356
Dnajc15	66148	0.234948	0.998356
Prr13	66151	0.618325	0.998356
Uqcr10	66152	0.39685	0.998356
Fbxo36	66153	-0.23456	0.998356
Tmem14c	66154	-0.44144	0.998356

Ufc1	66155	0.394737	0.998356
Anapc11	66156	-0.10344	0.998356
Pop4	66161	0.128667	0.998356
Bola2	66162	-0.40633	0.998356
Mrpl4	66163	-0.33419	0.998356
Nip7	66164	-0.14636	0.998356
Bccip	66165	0.1802	0.998356
Ccdc72	66167	-0.20282	0.998356
Grina	66168	0.029207	0.998356
Tomm7	66169	0.201285	0.998356
Chchd5	66170	-0.15787	0.998356
Pgls	66171	-0.01814	0.998356
Med11	66172	0.061261	0.998356
Nudt14	66174	-0.33574	0.998356
Mustn1	66175	-0.06535	0.998356
Nat9	66176	-0.2727	0.998356
Ubl5	66177	-0.17725	0.998356
1110031I02Rik	66179	0.225224	0.998356
Leprel4	66180	-0.4381	0.998356
Nop10	66181	-0.11195	0.998356
Rps4y2	66184	0.299627	0.998356
1110037F02Rik	66185	0.044398	0.998356
Acer3	66190	0.406601	0.998356
Ier3ip1	66191	-0.33269	0.998356
Lage3	66192	0.398021	0.998356
1110049F12Rik	66193	0.642814	0.998356
Pycrl	66194	0.157882	0.998356
Myo19	66196	-0.15078	0.998356
Cks2	66197	0.240947	0.998356
Commd4	66199	0.227169	0.998356
Commd6	66200	0.591068	0.998356
Vta1	66201	0.324401	0.998356
Acyp1	66204	0.53601	0.998356
Cd302	66205	0.598016	0.998356
1110059E24Rik	66206	0.202341	0.998356
Nenf	66208	0.150921	0.998356
1110054O05Rik	66209	-0.23775	0.998356
Sec61b	66212	0.470499	0.998356
Med7	66213	-0.97537	0.998356
1190002H23Rik	66214	0.374428	0.998356
Ndufb9	66218	0.336445	0.998356
Zdhhc12	66220	-0.31205	0.998356
Mrpl35	66223	-0.21767	0.998356
Trappc2	66226	0.3903	0.998356
Rpl7l1	66229	-0.20085	0.998356
Mrps28	66230	-0.33176	0.998356
Dmap1	66233	-0.11401	0.998356

Sc4mol	66234	0.285822	0.998356
Eif1ax	66235	-0.08936	0.998356
1500011B03Rik	66236	-0.27371	0.998356
Atp6v1g2	66237	0.44607	0.998356
Tmem9	66241	0.3924	0.998356
Mrps16	66242	0.563631	0.998356
Nemf	66244	0.00125	0.998356
Hspbp1	66245	0.195741	0.998356
Osgep	66246	0.099822	0.998356
Alg5	66248	0.27732	0.998356
Pno1	66249	-0.26547	0.998356
Arfgap3	66251	0.264967	0.998356
Aig1	66253	-0.16041	0.998356
Dimt1	66254	-0.21405	0.998356
Hsbp1l1	66255	0.073328	0.998356
Ssr2	66256	-0.06481	0.998356
Nicn1	66257	0.198226	0.998356
Mrps17	66258	-0.08163	0.998356
Camk2n1	66259	0.072411	0.998356
Tmem54	66260	0.344029	0.998356
Tm4sf20	66261	0.134337	0.998356
Ing5	66262	0.283899	0.998356
Ccdc28b	66264	0.942266	0.998356
Eapp	66266	0.167714	0.998356
Pigyl	66268	0.473764	0.998356
Tmed6	66269	0.310879	0.998356
Fam134b	66270	-0.12496	0.998356
Cox16	66272	0.229122	0.998356
1810020D17Rik	66273	-0.70273	0.998356
1810012P15Rik	66274	0.07201	0.998356
1810009A15Rik	66276	-0.29795	0.998356
1810013D10Rik	66278	-0.12095	0.998356
Tmem218	66279	0.232901	0.998356
1810029B16Rik	66282	0.836345	0.998356
Sec11c	66286	0.123665	0.998356
Atp6v1g1	66290	0.087897	0.998356
1810030N24Rik	66291	0.408772	0.998356
Mrps21	66292	-0.0546	0.998356
Fam3a	66294	0.415024	0.998356
Haus2	66296	-0.05768	0.998356
Defa21	66298	-0.6356	0.998356
Fam82b	66302	0.183052	0.998356
Fam53c	66306	-0.3017	0.998356
Isoc1	66307	0.010895	0.998356
2810021B07Rik	66308	-0.10845	0.998356
Tmem128	66309	0.216525	0.998356
Dpy30	66310	0.370185	0.998356

Cenpw	66311	0.758766	0.998356
Smurf2	66313	-0.02733	0.998356
Tpd52l2	66314	0.233493	0.998356
Senp7	66315	-0.92836	0.998356
Wdr61	66317	-0.85317	0.998356
1700020L24Rik	66330	0.946998	0.998356
Aqp11	66333	0.242475	0.998356
Atp6v1c1	66335	0.132366	0.998356
Cenpp	66336	0.346029	0.998356
Psenen	66340	0.069556	0.998356
Eid3	66341	0.065313	0.998356
Atp5sl	66349	0.032658	0.998356
Pla2g12a	66350	0.104059	0.998356
Blzf1	66352	-0.13699	0.998356
Snw1	66354	-0.10843	0.998356
Gmpr	66355	-0.43408	0.998356
2310008H09Rik	66356	-0.28947	0.998356
Ostc	66357	0.119486	0.998356
2310004I24Rik	66358	0.409484	0.998356
Fam36a	66359	-0.47626	0.998356
Zfand1	66361	-0.18637	0.998356
Exosc3	66362	0.469481	0.998356
2310009A05Rik	66364	0.276623	0.998356
Ccdc90b	66365	-0.09529	0.998356
Ergic3	66366	0.284975	0.998356
Rtcd1	66368	0.271086	0.998356
Dus2l	66369	0.048521	0.998356
Chmp4c	66371	0.263186	0.998356
Lsm5	66373	1.54594	0.998356
2310011J03Rik	66374	0.813075	0.998356
Dhrs7	66375	-0.17865	0.998356
Ndufc1	66377	0.478375	0.998356
2310016M24Rik	66379	0.087487	0.998356
Rnf113a2	66381	-0.3509	0.998356
Iscu	66383	-0.12884	0.998356
Srp19	66384	-0.12923	0.998356
Ppp1r7	66385	-0.31892	0.998356
Nudt8	66387	0.222313	0.998356
Cutc	66388	-0.33264	0.998356
Slmo2	66390	0.132568	0.998356
Nosip	66394	-0.18585	0.998356
Ahnak	66395	1.07029	0.998356
Ccdc82	66396	0.161641	0.998356
Sar1b	66397	0.093004	0.998356
Commd5	66398	0.266318	0.998356
Tsfm	66399	0.163493	0.998356
Alkbh7	66400	0.467135	0.998356

Nudt2	66401	0.114889	0.998356
Asf1a	66403	-0.33879	0.998356
2410001C21Rik	66404	0.060889	0.998356
Mcts2	66405	-0.0947	0.998356
Sac3d1	66406	-0.40819	0.998356
Mrps15	66407	-0.2946	0.998356
Aptx	66408	0.391092	0.998356
Rsl1d1	66409	-0.2045	0.998356
Mterfd1	66410	-0.33862	0.998356
Tbcb	66411	0.265879	0.998356
Arrdc4	66412	0.35951	0.998356
Psmd6	66413	0.032247	0.998356
Ndufa12	66414	0.119063	0.998356
Ndufa7	66416	0.254037	0.998356
Mrpl11	66419	0.000946	0.998356
Polr2e	66420	-0.19095	0.998356
2410004B18Rik	66421	0.686324	0.998356
Dctpp1	66422	0.272775	0.998356
2410022L05Rik	66423	-0.03065	0.998356
Pcp4l1	66425	-0.33284	0.998356
Cyb5b	66427	-0.08569	0.998356
1810049H13Rik	66431	0.362034	0.998356
Slc7a6os	66432	-0.35147	0.998356
Chchd7	66433	-1.60944	0.998356
Fis1	66437	-0.38044	0.998356
Hamp2	66438	0.392218	0.998356
2010012O05Rik	66439	0.215473	0.998356
Cdc26	66440	-0.17859	0.998356
Magohb	66441	-0.16908	0.998356
Spc25	66442	-0.0008	0.998356
Tnfaip8l1	66443	-0.44791	0.998356
Cyc1	66445	-0.51653	0.998356
Exosc7	66446	-0.77009	0.998356
Pam16	66449	0.04387	0.998356
2610528J11Rik	66451	0.023251	0.998356
Nmnat1	66454	-0.41373	0.998356
Cnpy4	66455	0.256066	0.998356
2810001G20Rik	66456	-0.09404	0.998356
2810002D19Rik	66457	-0.21525	0.998356
Sys1	66460	0.514142	0.998356
Ptpmt1	66461	0.066898	0.998356
2810428I15Rik	66462	0.248319	0.998356
Taf12	66464	-0.16838	0.998356
Gtf2h5	66467	0.14289	0.998356
2810405K02Rik	66469	0.27349	0.998356
Anp32e	66471	-0.67648	0.998356
Rps23	66475	-0.21327	0.998356

Rpl15	66480	-0.20565	0.998356
Exoc2	66482	-0.07955	0.998356
Rpl36al	66483	0.244968	0.998356
2010107H07Rik	66487	0.817182	0.998356
Fam136a	66488	-0.48559	0.998356
Rpl35	66489	-0.19091	0.998356
Polr2l	66491	0.100409	0.998356
Zmat2	66492	-0.03727	0.998356
Mrpl51	66493	-0.25765	0.998356
Prelid1	66494	0.411863	0.998356
Ndufb3	66495	-0.46351	0.998356
Ppdpf	66496	-0.03131	0.998356
2610528E23Rik	66497	0.326724	0.998356
Dda1	66498	-0.0901	0.998356
Slc30a7	66500	-0.05875	0.998356
Zmynd11	66505	0.120911	0.998356
Psmg3	66506	-0.09791	0.998356
2400001E08Rik	66508	0.15505	0.998356
Rnf181	66510	0.171406	0.998356
2500003M10Rik	66511	-0.27429	0.998356
Tab1	66513	0.158231	0.998356
Cul7	66515	0.49035	0.998356
2610001J05Rik	66520	0.18264	0.998356
Rwdd1	66521	-0.14311	0.998356
Pgpep1	66522	0.009687	0.998356
2810004N23Rik	66523	0.067997	0.998356
Timm50	66525	0.03093	0.998356
Ubxn6	66530	0.530783	0.998356
2310061C15Rik	66531	0.116612	0.998356
Rep15	66532	-0.4901	0.998356
Nipsnap3b	66536	0.040331	0.998356
Pomp	66537	0.487422	0.998356
Rps19bp1	66538	0.093632	0.998356
Fam107b	66540	0.08596	0.998356
Immp1l	66541	0.34982	0.998356
Aggf1	66549	0.112056	0.998356
2010106G01Rik	66552	0.471702	0.998356
Drap1	66556	-0.21663	0.998356
Metap1d	66559	-0.14413	0.998356
Ntpcr	66566	-0.57441	0.998356
Gdpd1	66569	-0.35696	0.998356
Cenpm	66570	3.28058	0.998356
Uqcrh	66576	0.527597	0.998356
2610039C10Rik	66578	0.282588	0.998356
Esf1	66580	-0.25191	0.998356
Exosc1	66583	0.231817	0.998356
Snrnp40	66585	0.096909	0.998356

Crls1	66586	0.022576	0.998356
Fastk	66587	0.12423	0.998356
Cmpk1	66588	0.162969	0.998356
Ube2v1	66589	0.531848	0.998356
Mad2l1bp	66591	0.067478	0.998356
Stoml2	66592	-0.17101	0.998356
Diablo	66593	0.447778	0.998356
Aste1	66595	-0.23494	0.998356
Gtf3a	66596	-0.09575	0.998356
Trim13	66597	0.097565	0.998356
3110001I22Rik	66598	0.168191	0.998356
Rdm1	66599	0.487613	0.998356
1700020I14Rik	66602	0.010746	0.998356
Lrrc57	66606	0.015392	0.998356
Cryzl1	66609	-0.29917	0.998356
Abi3	66610	0.153718	0.998356
Ribc1	66611	0.00126	0.998356
Ormdl3	66612	0.26946	0.998356
Gpatch4	66614	-0.20764	0.998356
Atg4b	66615	-0.10375	0.998356
Snx9	66616	0.298748	0.998356
Mettl11a	66617	-0.34351	0.998356
Snrnp27	66618	0.128534	0.998356
Ubr7	66622	-0.00416	0.998356
Spcs2	66624	-0.34637	0.998356
Sfrs18	66625	0.067278	0.998356
5730403B10Rik	66626	-0.19157	0.998356
Ogfod2	66627	-0.43608	0.998356
Thg1l	66628	0.430173	0.998356
Golph3	66629	-0.2441	0.998356
Hiatl1	66631	0.323328	0.998356
Atpbd4	66632	0.008498	0.998356
Tsen15	66637	-0.1823	0.998356
Sike1	66641	0.051603	0.998356
Ctnnbl1	66642	0.042479	0.998356
Lix1	66643	0.736074	0.998356
Pspc1	66645	0.278334	0.998356
Rpe	66646	-0.39876	0.998356
5730494M16Rik	66648	-0.19133	0.998356
Brf2	66653	-0.15197	0.998356
Eef1d	66656	-0.48927	0.998356
Ccdc51	66658	-0.28432	0.998356
Acp6	66659	0.086553	0.998356
Sltm	66660	0.062772	0.998356
Srp72	66661	-0.16555	0.998356
Uba5	66663	-0.04572	0.998356
Tmem41a	66664	-0.02566	0.998356

5730528L13Rik	66665	-0.33669	0.998356
Hspbap1	66667	-0.00189	0.998356
Ccnh	66671	0.071063	0.998356
6330409N04Rik	66674	0.077257	0.998356
Tmed7	66676	0.121848	0.998356
Rae1	66679	-0.45439	0.998356
3230401D17Rik	66680	0.163779	0.998356
Pgm1	66681	0.227371	0.998356
Trappc5	66682	0.050913	0.998356
Tceal8	66684	0.271213	0.998356
Dcbld1	66686	-0.06733	0.998356
Tbc1d15	66687	0.205563	0.998356
Klhl28	66689	-0.09195	0.998356
Tmem186	66690	0.018635	0.998356
Gapvd1	66691	0.123682	0.998356
Uqcrfs1	66694	0.099809	0.998356
Chmp3	66700	0.362647	0.998356
Spryd4	66701	-0.11091	0.998356
Rbm4b	66704	-0.44747	0.998356
Ndufaf3	66706	0.228962	0.998356
Sbds	66711	-0.1046	0.998356
Actr2	66713	-0.19718	0.998356
4921524J17Rik	66714	-0.18852	0.998356
Tab3	66724	0.170167	0.998356
Map1lc3a	66734	0.323565	0.998356
Ttc35	66736	-0.18671	0.998356
Rnf220	66743	0.134212	0.998356
Erlec1	66753	0.003773	0.998356
4933411K20Rik	66756	0.082244	0.998356
Adat2	66757	-0.03639	0.998356
Pacrgl	66768	0.146613	0.998356
4933439F18Rik	66771	-0.12805	0.998356
Ptplad2	66775	0.678164	0.998356
4933433P14Rik	66787	0.11499	0.998356
Alg14	66789	-0.33945	0.998356
Grtp1	66790	0.075632	0.998356
Atg10	66795	0.061188	0.998356
Ube2w	66799	-0.04501	0.998356
Prkrip1	66801	0.451047	0.998356
Tspan1	66805	0.827623	0.998356
Rbm22	66810	-0.37177	0.998356
Duoxa2	66811	0.592869	0.998356
Ppcdc	66812	0.046787	0.998356
Bcl2l14	66813	-0.08301	0.998356
Ccdc109b	66815	-0.06385	0.998356
Thap2	66816	-0.11567	0.998356
Tmem170	66817	0.534436	0.998356

9130011J15Rik	66818	0.07377	0.998356
Bcs1l	66821	-0.33988	0.998356
Fbxo25	66822	0.230498	0.998356
Pycard	66824	0.596474	0.998356
Taz	66826	2.48549	0.998356
Ttc1	66827	0.163206	0.998356
Nacc1	66830	-0.11918	0.998356
Rsph3a	66832	-0.619	0.998356
Acot13	66834	0.283206	0.998356
Tmem223	66836	0.291258	0.998356
0610009O20Rik	66839	0.10195	0.998356
Wdr45l	66840	0.39302	0.998356
Etfdh	66841	0.118282	0.998356
Ormdl2	66844	0.49392	0.998356
Mrpl33	66845	-0.09136	0.998356
Hint3	66847	0.233671	0.998356
Fuca2	66848	-0.00751	0.998356
Ppp1r2	66849	0.150036	0.998356
Pnpla2	66853	-0.22888	0.998356
Trim35	66854	0.028688	0.998356
Tcf25	66855	-0.47002	0.998356
Plbd1	66857	-0.10095	0.998356
Tanc1	66860	0.17112	0.998356
Dnajc10	66861	-0.028	0.998356
Lztr1	66863	0.110667	0.998356
Pmpca	66865	-0.01048	0.998356
Nhlrc2	66866	-0.0602	0.998356
Hmg20a	66867	-0.17527	0.998356
Mfsd1	66868	-0.18376	0.998356
Zfp869	66869	-0.46079	0.998356
Serbp1	66870	0.06312	0.998356
Cpne8	66871	0.592371	0.998356
1200014J11Rik	66874	-0.16194	0.998356
1200016B10Rik	66875	0.183318	0.998356
Crnkl1	66877	0.139843	0.998356
Riok3	66878	0.14245	0.998356
Rsrc1	66880	-0.18663	0.998356
Pcyox1	66881	0.053982	0.998356
Bzw1	66882	-0.29724	0.998356
Appbp2	66884	0.09867	0.998356
Acadsb	66885	-0.07342	0.998356
Lonp2	66887	0.032576	0.998356
Lman2	66890	-0.06599	0.998356
Eif4e3	66892	-0.00783	0.998356
Wwp2	66894	0.059455	0.998356
1300014I06Rik	66895	1.0285	0.998356
Naa16	66897	-0.3104	0.998356

Baiap2l1	66898	0.255627	0.998356
Fip1l1	66899	0.017377	0.998356
Mtap	66902	-0.45208	0.998356
Pccb	66904	0.288618	0.998356
Plin3	66905	-0.03755	0.998356
Tmem107	66910	-0.77791	0.998356
Nudt16l1	66911	-0.44783	0.998356
Bzw2	66912	-0.48533	0.998356
Kdelr2	66913	0.04503	0.998356
Vps28	66914	0.087587	0.998356
Myeov2	66915	0.540458	0.998356
Ndufb7	66916	-0.24966	0.998356
Chordc1	66917	-0.27708	0.998356
Prpf38b	66921	-0.02411	0.998356
Rras2	66922	0.206477	0.998356
Pbrm1	66923	-0.01122	0.998356
Sdhd	66925	0.082273	0.998356
Trmt6	66926	0.008107	0.998356
3110001D03Rik	66928	0.227637	0.998356
Asf1b	66929	-0.25105	0.998356
Rexo1	66932	-0.34034	0.998356
Dsn1	66934	-0.35167	0.998356
Cir1	66935	0.399234	0.998356
1700029G01Rik	66938	-0.06853	0.998356
Aagab	66939	0.236049	0.998356
Shisa5	66940	-0.19841	0.998356
Ddx18	66942	-0.29959	0.998356
Pqlc1	66943	0.051944	0.998356
Sdha	66945	0.067456	0.998356
Acad8	66948	-0.10378	0.998356
Trim59	66949	-0.25802	0.998356
Tmem206	66950	-0.19448	0.998356
2310030G06Rik	66952	0.131443	0.998356
Cdca7	66953	-0.20275	0.998356
Tmx2	66958	0.024402	0.998356
Fam188a	66960	0.396077	0.998356
Neat1	66961	0.308477	0.998356
2310047B19Rik	66962	-0.05135	0.998356
Golt1b	66964	0.045626	0.998356
Ctu2	66965	-0.00851	0.998356
Trit1	66966	-0.03823	0.998356
Edem3	66967	0.018408	0.998356
Slc25a23	66972	-0.09677	0.998356
Mrps18b	66973	-0.22893	0.998356
2410002O22Rik	66975	0.381485	0.998356
Nuf2	66977	-0.17609	0.998356
Luc7l	66978	0.051093	0.998356

Pole4	66979	-0.3638	0.998356
Zdhhc6	66980	-0.53503	0.998356
Zfp830	66983	0.073872	0.998356
Rassf7	66985	0.165158	0.998356
Lap3	66988	0.387068	0.998356
Kctd20	66989	-0.24782	0.998356
Tmem134	66990	-0.01238	0.998356
1500031L02Rik	66994	-0.14593	0.998356
Psmd12	66997	-0.17264	0.998356
Psmd5	66998	0.474563	0.998356
Med28	66999	-0.24276	0.998356
Uqcrc2	67003	0.165675	0.998356
Polr3k	67005	-0.10203	0.998356
Cisd2	67006	0.238066	0.998356
1600012F09Rik	67008	0.114532	0.998356
Ttc23	67009	-0.44121	0.998356
Rbm7	67010	0.100956	0.998356
Mettl6	67011	0.120194	0.998356
Oma1	67013	0.28782	0.998356
Mina	67014	-0.63455	0.998356
Ccdc91	67015	-0.28474	0.998356
Tbc1d2b	67016	0.295146	0.998356
2010011I20Rik	67017	-0.22897	0.998356
Actr6	67019	0.034249	0.998356
Tmem88	67020	-0.50325	0.998356
Use1	67023	0.211139	0.998356
Rpl11	67025	0.177452	0.998356
Thap4	67026	-0.09074	0.998356
Mkrn2	67027	-0.17054	0.998356
2610002M06Rik	67028	-0.08617	0.998356
Fancl	67030	0.014631	0.998356
Upf3a	67031	-0.28048	0.998356
Dnajb4	67035	0.017292	0.998356
Mrpl45	67036	0.154049	0.998356
Pmf1	67037	0.725117	0.998356
Rbm25	67039	-0.00771	0.998356
Ddx17	67040	-0.0795	0.998356
Oxct1	67041	0.289889	0.998356
Ift27	67042	0.289384	0.998356
Syap1	67043	0.469368	0.998356
Higd2a	67044	-0.08994	0.998356
Riok2	67045	0.312878	0.998356
Tbc1d7	67046	-0.10166	0.998356
Vma21	67048	-0.10138	0.998356
Pus3	67049	-0.46022	0.998356
Nkap	67050	-0.24081	0.998356
Ndc80	67052	0.148045	0.998356

Rpp14	67053	-0.23606	0.998356
Paics	67054	0.090743	0.998356
Yaf2	67057	-0.23961	0.998356
Ola1	67059	-0.27449	0.998356
Chmp1b	67064	0.309723	0.998356
Polr3d	67065	0.063511	0.998356
Romo1	67067	-0.46927	0.998356
Dynlrb1	67068	0.011889	0.998356
Lsm14a	67070	0.024696	0.998356
Cdc37l1	67072	-0.0127	0.998356
Pi4k2b	67073	0.495507	0.998356
Mon2	67074	0.27147	0.998356
Magt1	67075	-0.16876	0.998356
Pgp	67078	-0.12084	0.998356
1700019D03Rik	67080	0.313092	0.998356
Ctnnbip1	67087	-1.1679	0.998356
Cand2	67088	-0.01182	0.998356
Psmc6	67089	-0.13455	0.998356
Trappc6a	67091	-0.66212	0.998356
Gatm	67092	0.13497	0.998356
Trak1	67095	0.305087	0.998356
Mmachc	67096	-0.24349	0.998356
Rps10	67097	0.413646	0.998356
Mettl21a	67099	0.14036	0.998356
2310039H08Rik	67101	0.463402	0.998356
D16Ertd472e	67102	-0.64453	0.998356
Ptgr1	67103	0.086961	0.998356
1700034H14Rik	67105	-0.07023	0.998356
Zbtb8os	67106	0.503266	0.998356
Zfp787	67109	0.550382	0.998356
Naaa	67111	0.546152	0.998356
Rpl14	67115	-0.14559	0.998356
Cuedc2	67116	0.137927	0.998356
Dynlt3	67117	0.255736	0.998356
Bfar	67118	-1.51903	0.998356
Ttc14	67120	-0.12095	0.998356
Mastl	67121	0.010869	0.998356
Nrarp	67122	-0.23329	0.998356
Ubap1	67123	-0.0318	0.998356
Tspan31	67125	0.102518	0.998356
Ube2g1	67128	-0.16025	0.998356
Ndufa6	67130	0.071411	0.998356
Acbd4	67131	-0.35701	0.998356
Nop56	67134	-0.6063	0.998356
Kbtbd4	67136	-0.01303	0.998356
Herc6	67138	0.764519	0.998356
Mis12	67139	-0.35148	0.998356

Fbxo5	67141	-0.29282	0.998356
Ikzf5	67143	-0.09453	0.998356
Lrrc40	67144	-0.1496	0.998356
Tomm34	67145	0.116313	0.998356
Fam103a1	67148	0.41153	0.998356
Rnf141	67150	-0.12077	0.998356
Psmd9	67151	0.079224	0.998356
Rnaseh2b	67153	-0.01671	0.998356
Mtdh	67154	0.032488	0.998356
Smarca2	67155	0.322703	0.998356
2610301B20Rik	67157	-0.59386	0.998356
Eef1g	67160	0.010922	0.998356
Ccdc47	67163	-0.27188	0.998356
Lipt2	67164	0.258855	0.998356
Arl8b	67166	0.155824	0.998356
Lpar6	67168	0.080394	0.998356
Nradd	67169	-0.34665	0.998356
2610306M01Rik	67170	0.458677	0.998356
Dram2	67171	-0.71681	0.998356
Cdt1	67177	0.010215	0.998356
Zmat5	67178	0.184469	0.998356
Ccdc25	67179	0.106924	0.998356
Yipf5	67180	0.050777	0.998356
Ctdnep1	67181	0.01833	0.998356
Pdzk1ip1	67182	1.47137	0.998356
Ndufa13	67184	-0.14584	0.998356
Rplp2	67186	-0.42174	0.998356
Zmynd19	67187	0.150965	0.998356
Ube2t	67196	0.626608	0.998356
Zcrb1	67197	-0.05408	0.998356
Spats2l	67198	0.487875	0.998356
Pfdn1	67199	-0.50557	0.998356
Glod4	67201	0.356887	0.998356
Nde1	67203	-0.0331	0.998356
Eif2s2	67204	0.54088	0.998356
Utp11l	67205	-0.17765	0.998356
Lsm1	67207	0.408278	0.998356
Gatad1	67210	0.314445	0.998356
Armc10	67211	0.045932	0.998356
Mrpl55	67212	-0.28875	0.998356
Cmtm6	67213	0.166913	0.998356
Mboat2	67216	-0.58768	0.998356
Med18	67219	-0.09261	0.998356
Plekho1	67220	0.093912	0.998356
Srfbp1	67222	-0.40965	0.998356
Rrp15	67223	-0.10785	0.998356
Med29	67224	0.048937	0.998356

Rnpc3	67225	0.230876	0.998356
Tmem19	67226	0.201242	0.998356
Wdr85	67228	-0.48116	0.998356
Prpf18	67229	0.134759	0.998356
Zfp329	67230	-0.16231	0.998356
Tbc1d20	67231	0.487135	0.998356
Zkscan14	67235	0.225952	0.998356
Cinp	67236	0.067604	0.998356
2810453I06Rik	67238	0.029912	0.998356
Smc6	67241	-0.10619	0.998356
Gemin6	67242	-0.40233	0.998356
Peli1	67245	0.078559	0.998356
2810474O19Rik	67246	0.089608	0.998356
Mosc2	67247	0.058542	0.998356
Rpl39	67248	0.301845	0.998356
Tbc1d19	67249	0.077488	0.998356
Cap2	67252	-0.37727	0.998356
Zfp422	67255	-0.23562	0.998356
Lass4	67260	0.226311	0.998356
Zswim6	67263	0.529948	0.998356
Fam69a	67266	0.070519	0.998356
2900010M23Rik	67267	0.373617	0.998356
Myl12a	67268	0.251415	0.998356
Mrpl42	67270	-0.2734	0.998356
Ndufa10	67273	0.123734	0.998356
Eri1	67276	-0.13192	0.998356
2900092E17Rik	67278	-0.16203	0.998356
Med31	67279	0.403136	0.998356
Rpl37	67281	-0.12512	0.998356
Ccdc53	67282	0.388457	0.998356
Slc25a19	67283	-0.68178	0.998356
Cwc27	67285	0.175774	0.998356
Rabl5	67286	-0.28834	0.998356
Parp6	67287	-0.12185	0.998356
Srek1ip1	67288	-0.17706	0.998356
3110040N11Rik	67290	-0.23451	0.998356
Ccdc137	67291	-0.55946	0.998356
Pigc	67292	0.816384	0.998356
Socs4	67296	0.099679	0.998356
Gprasp1	67298	0.298297	0.998356
Dock7	67299	0.297068	0.998356
Cltc	67300	0.245207	0.998356
Zc3h13	67302	0.115008	0.998356
3110045C21Rik	67303	-0.20666	0.998356
3110070M22Rik	67304	-1.61747	0.998356
Gpx7	67305	0.277642	0.998356
Fam164a	67306	-0.5984	0.998356

Mrpl46	67308	-0.128	0.998356
5730559C18Rik	67313	-0.02637	0.998356
Ceacam12	67315	0.477733	0.998356
1700037H04Rik	67326	-0.15841	0.998356
1700018L02Rik	67329	-0.63059	0.998356
Snrpd3	67332	-0.62175	0.998356
Stk35	67333	-0.1204	0.998356
Cstf1	67337	-0.22022	0.998356
Rffl	67338	-0.47867	0.998356
Herc4	67345	0.084954	0.998356
Gcfc1	67367	0.030134	0.998356
Qpctl	67369	0.004961	0.998356
Zfp606	67370	-0.21498	0.998356
Gtf3c6	67371	0.10648	0.998356
Bbs2	67378	-0.02011	0.998356
Dedd2	67379	0.247685	0.998356
Med4	67381	0.034299	0.998356
Brd3	67382	-0.29605	0.998356
2410127L17Rik	67383	0.285329	0.998356
Bag4	67384	-0.00628	0.998356
Unc50	67387	-0.06862	0.998356
1110008F13Rik	67388	0.348697	0.998356
Rnmtl1	67390	0.076168	0.998356
Fundc2	67391	-0.11072	0.998356
4833420G17Rik	67392	-0.38364	0.998356
Cxxc5	67393	0.297727	0.998356
Erp29	67397	0.106402	0.998356
Srpr	67398	-0.00613	0.998356
Pdlim7	67399	0.323521	0.998356
Mfn1	67414	0.086143	0.998356
Ears2	67417	-0.07676	0.998356
Ppil4	67418	0.020724	0.998356
Far1	67420	-0.88205	0.998356
Dhdds	67422	0.052614	0.998356
Eps8l1	67425	0.16922	0.998356
Rps20	67427	0.013725	0.998356
Nudcd1	67429	-0.62216	0.998356
Hoga1	67432	0.811051	0.998356
Ccdc127	67433	-1.77418	0.998356
Ssr3	67437	0.003702	0.998356
Xab2	67439	0.230518	0.998356
Mtpap	67440	0.040785	0.998356
Isoc2b	67441	-0.53113	0.998356
Retsat	67442	-0.13379	0.998356
Map1lc3b	67443	-0.07216	0.998356
Ilkap	67444	-0.15451	0.998356
Dusp28	67446	0.323522	0.998356

Pkp2	67451	0.205851	0.998356
Pnpla8	67452	0.075424	0.998356
Slc25a46	67453	0.156714	0.998356
Ikbip	67454	0.526655	0.998356
Ergic2	67456	0.035262	0.998356
Frmd8	67457	0.013496	0.998356
Ergic1	67458	-0.44457	0.998356
Nvl	67459	0.041657	0.998356
Decr1	67460	-0.1741	0.998356
Poc5	67463	0.238662	0.998356
Sf3a1	67465	-0.12222	0.998356
Pdcl	67466	-0.03047	0.998356
1200011I18Rik	67467	-0.00382	0.998356
Mmd	67468	-0.69343	0.998356
Abhd5	67469	0.231196	0.998356
Gpatch1	67471	0.083888	0.998356
Mtfr1	67472	0.983846	0.998356
Snap29	67474	0.044089	0.998356
Cwc25	67480	-0.10514	0.998356
Eepd1	67484	0.342379	0.998356
Polr3g	67486	-0.20601	0.998356
Dhx40	67487	0.098637	0.998356
Calcoco1	67488	0.043608	0.998356
Ap4b1	67489	0.068085	0.998356
1810074P20Rik	67490	0.222372	0.998356
Mettl16	67493	-0.25714	0.998356
Tmem167b	67495	-0.18729	0.998356
Ccar1	67500	-0.03267	0.998356
Ccdc50	67501	-0.11512	0.998356
1810063B07Rik	67509	0.160362	0.998356
Fam18b	67510	0.016139	0.998356
Tmed9	67511	-0.10566	0.998356
Agpat2	67512	0.416314	0.998356
2610002J02Rik	67513	-0.11823	0.998356
Ttc33	67515	-0.46073	0.998356
1700097N02Rik	67522	0.429336	0.998356
Trim62	67525	0.182032	0.998356
Atg12	67526	0.366595	0.998356
Nudt7	67528	0.40109	0.998356
Fgfr1op2	67529	-0.14415	0.998356
Uqcrb	67530	0.574518	0.998356
5730408K05Rik	67531	0.757129	0.998356
Mfap1a	67532	0.067126	0.998356
Ppfibp1	67533	-0.24324	0.998356
Ttll4	67534	-0.05004	0.998356
Cog6	67542	0.183587	0.998356
Fam120b	67544	-0.48604	0.998356

Slc39a8	67547	0.196071	0.998356
Gpr89	67549	0.072468	0.998356
Gstcd	67553	0.006179	0.998356
Slc25a30	67554	-0.75062	0.998356
Pigm	67556	-0.05215	0.998356
Wdr48	67561	0.199112	0.998356
Narfl	67563	0.198127	0.998356
Mrfap1	67568	0.076703	0.998356
Alg13	67574	0.51277	0.998356
Cpeb4	67579	-0.00722	0.998356
Tbc1d23	67581	0.284213	0.998356
Slc25a26	67582	-0.13464	0.998356
Rnf41	67588	-0.12559	0.998356
Necap1	67602	0.077637	0.998356
Dusp6	67603	0.356793	0.998356
Get4	67604	1.31137	0.998356
Akt1s1	67605	-0.85327	0.998356
Narf	67608	0.08233	0.998356
4930453N24Rik	67609	0.12543	0.998356
Rspry1	67610	-0.17185	0.998356
Ube2r2	67615	0.251785	0.998356
Aasdhppt	67618	-0.23281	0.998356
Nob1	67619	-0.13418	0.998356
Tm7sf3	67623	0.132583	0.998356
Anp32b	67628	-0.24747	0.998356
Spc24	67629	-0.34353	0.998356
Samd8	67630	0.159093	0.998356
Lyrm5	67636	-0.01696	0.998356
4930523C07Rik	67647	0.059106	0.998356
Ctdp1	67655	0.244736	0.998356
Rabl3	67657	-0.31402	0.998356
Ift172	67661	0.222402	0.998356
Rnf125	67664	-0.03731	0.998356
Dctn4	67665	0.03807	0.998356
Alkbh8	67667	-0.10222	0.998356
l7Rn6	67669	0.096544	0.998356
Rpl38	67671	-3.96639	0.998356
0610040B10Rik	67672	0.0738	0.998356
Tceb2	67673	-0.19452	0.998356
Trmt112	67674	-0.50408	0.998356
Cuta	67675	-0.30634	0.998356
Lsm3	67678	0.116166	0.998356
Sdhb	67680	0.104995	0.998356
Mrpl18	67681	-0.13924	0.998356
2610029G23Rik	67683	-0.55399	0.998356
Luc7l3	67684	-0.0564	0.998356
Aldh3b1	67689	0.084248	0.998356

2310003F16Rik	67693	0.253674	0.998356
Ift74	67694	-0.03409	0.998356
Ost4	67695	0.256448	0.998356
Fam174a	67698	0.63099	0.998356
Wfdc2	67701	0.483252	0.998356
Rnf149	67702	0.408702	0.998356
1810037I17Rik	67704	0.522194	0.998356
1810058I24Rik	67705	0.361627	0.998356
Tmem179b	67706	-1.00579	0.998356
Mrpl24	67707	-0.38648	0.998356
Reg4	67709	-0.05747	0.998356
Polr2g	67710	-0.11353	0.998356
Nsmce1	67711	0.356376	0.998356
Slc25a37	67712	-0.27226	0.998356
Dnajc19	67713	0.343059	0.998356
Pop1	67724	-0.00984	0.998356
Fam114a2	67726	0.609971	0.998356
Stx17	67727	0.124658	0.998356
Dph2	67728	-0.1856	0.998356
Mansc1	67729	-0.02642	0.998356
Fbxo32	67731	-0.16108	0.998356
Iah1	67732	0.192541	0.998356
Ccdc130	67736	0.369859	0.998356
Slc48a1	67739	-0.04897	0.998356
Samsn1	67742	0.176626	0.998356
Ddx47	67755	-0.46095	0.998356
5033414D02Rik	67759	0.421819	0.998356
Slc38a2	67760	0.045086	0.998356
Prpsap1	67763	-0.09872	0.998356
N6amt1	67768	-0.0553	0.998356
Gpatch2	67769	-0.14056	0.998356
5830433M19Rik	67770	-0.09117	0.998356
Arpc5	67771	0.502405	0.998356
Chd8	67772	-0.13546	0.998356
Myst1	67773	0.045095	0.998356
Loh12cr1	67774	0.022622	0.998356
Rtp4	67775	0.475017	0.998356
Vwa5a	67776	0.107607	0.998356
Zfp639	67778	0.236664	0.998356
Ilf2	67781	-0.00344	0.998356
Plxnd1	67784	0.368588	0.998356
Zmym4	67785	0.083864	0.998356
Sfr1	67788	0.174765	0.998356
Dalrd3	67789	-0.18372	0.998356
Rnls	67795	1.22143	0.998356
Snrnp48	67797	0.111355	0.998356
Dgat2	67800	0.60584	0.998356

Limd2	67803	-0.20403	0.998356
Snx2	67804	-0.08191	0.998356
Tprgl	67808	0.243763	0.998356
Fam82a2	67809	-0.10956	0.998356
Poldip2	67811	0.06817	0.998356
Ubxn4	67812	-0.036	0.998356
Derl1	67819	0.263546	0.998356
Nmral1	67824	-0.24665	0.998356
Snap47	67826	-0.20971	0.998356
Brix1	67832	-0.1953	0.998356
Idh3a	67834	-0.22001	0.998356
Wdr83	67836	-0.38789	0.998356
Dnajb11	67838	0.188122	0.998356
Gpsm1	67839	-0.30435	0.998356
Mrp63	67840	0.198266	0.998356
Atg3	67841	-0.04058	0.998356
2610027L16Rik	67842	-0.48701	0.998356
Slc35a4	67843	-0.59167	0.998356
Rab32	67844	0.04677	0.998356
Rnf115	67845	-0.0554	0.998356
Tmem39a	67846	-0.42529	0.998356
Ddx55	67848	-0.12005	0.998356
Cdca5	67849	-0.16934	0.998356
1700021F05Rik	67851	0.129732	0.998356
Asprv1	67855	-0.29887	0.998356
Ppp6c	67857	0.408496	0.998356
S100a16	67860	0.227116	0.998356
Akr1b10	67861	-0.46317	0.998356
2310033P09Rik	67862	0.084832	0.998356
Slc25a11	67863	-0.13222	0.998356
Yipf4	67864	0.104163	0.998356
Rgs10	67865	0.288103	0.998356
Wfdc1	67866	-1.03929	0.998356
Lrrc28	67867	-0.20652	0.998356
Paip2	67869	-0.26991	0.998356
Mrrf	67871	-0.43376	0.998356
Nsmce4a	67872	-0.04543	0.998356
Mri1	67873	-0.622	0.998356
Coq10b	67876	0.158632	0.998356
Naa20	67877	0.520012	0.998356
Tmem33	67878	-0.05575	0.998356
Dcxr	67880	-0.38265	0.998356
Mdp1	67881	0.539489	0.998356
Uxs1	67883	0.725451	0.998356
1810043G02Rik	67884	-0.29599	0.998356
1500011K16Rik	67885	0.333061	0.998356
Camsap2	67886	-0.08281	0.998356

Tmem66	67887	-0.06935	0.998356
Rbm18	67889	-0.07612	0.998356
Ufm1	67890	-0.11262	0.998356
Rpl4	67891	-0.36595	0.998356
Tmem86a	67893	0.525475	0.998356
Fam45a	67894	0.450846	0.998356
Ppa1	67895	-0.49928	0.998356
Ccdc80	67896	-0.19155	0.998356
Rnmt	67897	0.110579	0.998356
Pef1	67898	0.054158	0.998356
Cmc1	67899	-0.48882	0.998356
Mtfp1	67900	-0.23525	0.998356
Sumf2	67902	-0.59886	0.998356
Gipc1	67903	-0.18494	0.998356
Ppm1m	67905	-0.26653	0.998356
Zfp169	67911	-0.40942	0.998356
1600012H06Rik	67912	-0.6691	0.998356
Coq9	67914	0.013513	0.998356
Ppap2b	67916	0.273012	0.998356
Mak16	67920	0.013271	0.998356
Ube2f	67921	-0.02942	0.998356
Fam32a	67922	0.324639	0.998356
Tceb1	67923	0.200729	0.998356
Hcfc2	67933	0.060301	0.998356
Wdr55	67936	0.14401	0.998356
Myl12b	67938	0.22147	0.998356
Prorsd1	67939	-0.80542	0.998356
Rps27l	67941	-0.10025	0.998356
Atp5g2	67942	0.313819	0.998356
Mesdc2	67943	-0.3609	0.998356
Rpl41	67945	0.077558	0.998356
Spata6	67946	0.607801	0.998356
Fbxo28	67948	0.014984	0.998356
Mki67ip	67949	-0.48219	0.998356
Tubb6	67951	-0.04003	0.998356
Tomm20	67952	-0.16513	0.998356
Sugt1	67955	-0.18029	0.998356
Setd8	67956	0.041879	0.998356
2610101N10Rik	67958	-0.15768	0.998356
Puf60	67959	0.03565	0.998356
Npc2	67963	-0.14777	0.998356
Zcchc10	67966	-0.03533	0.998356
Pold3	67967	-0.10958	0.998356
Atp2b1	67972	0.182734	0.998356
Mphosph10	67973	-0.27795	0.998356
Ccny	67974	0.433194	0.998356
Trabd	67976	-0.01332	0.998356

Tctn2	67978	-0.14462	0.998356
Atad1	67979	-0.12191	0.998356
Gnpda2	67980	0.549391	0.998356
Tmx3	67988	-0.0201	0.998356
Nacc2	67991	0.101623	0.998356
Nudt12	67993	-0.09491	0.998356
Mrps11	67994	-0.02597	0.998356
Srsf6	67996	0.045176	0.998356
Ddx59	67997	0.068235	0.998356
Fam134c	67998	0.436073	0.998356
1110004E09Rik	68001	0.074771	0.998356
1110058L19Rik	68002	0.338077	0.998356
Defa20	68009	-0.23621	0.998356
Bambi	68010	0.405651	0.998356
Snrpg	68011	-0.32518	0.998356
Zwilch	68014	0.140762	0.998356
Trap1	68015	0.008691	0.998356
Ftsj2	68017	0.019761	0.998356
Col4a3bp	68018	0.373899	0.998356
2810002N01Rik	68020	0.881398	0.998356
Bphl	68021	0.291782	0.998356
Pdf	68023	0.024755	0.998356
Hist1h2bc	68024	0.356204	0.998356
2810417H13Rik	68026	-0.60168	0.998356
Rpl22l1	68028	-0.4072	0.998356
Rnf146	68031	-0.26417	0.998356
Tmem85	68032	-0.11398	0.998356
Cox19	68033	-0.28459	0.998356
Fam122a	68034	-0.12132	0.998356
Rbm42	68035	-0.02572	0.998356
Zfp706	68036	-0.10157	0.998356
Chid1	68038	-0.22108	0.998356
Zfp593	68040	-0.0345	0.998356
Mid1ip1	68041	0.134734	0.998356
N6amt2	68043	0.467745	0.998356
Chac2	68044	0.14938	0.998356
2700060E02Rik	68045	0.125252	0.998356
2700062C07Rik	68046	-0.18194	0.998356
Mpnd	68047	-0.1948	0.998356
Aen	68048	-0.08864	0.998356
Akirin1	68050	0.296329	0.998356
Nutf2	68051	-0.16294	0.998356
Rps13	68052	-0.44226	0.998356
Ubxn2b	68053	-0.1104	0.998356
Atp5s	68055	0.291918	0.998356
Chd1l	68058	-0.04875	0.998356
Tm9sf2	68059	0.363333	0.998356

Slc25a39	68066	-0.40771	0.998356
3010026O09Rik	68067	0.203367	0.998356
Fam173b	68073	0.000605	0.998356
Gltscr2	68077	-0.30099	0.998356
Gpn3	68080	0.243881	0.998356
Dusp19	68082	0.071642	0.998356
Pak1ip1	68083	-0.08644	0.998356
Dcakd	68087	-0.48398	0.998356
Arpc4	68089	10	0.998356
Yif1a	68090	-0.28476	0.998356
Ncbp2	68092	-0.27549	0.998356
Smarcc2	68094	2.90855	0.998356
Ociad1	68095	-3.4407	0.998356
Dynll2	68097	0.003267	0.998356
Rchy1	68098	0.084014	0.998356
Fam92a	68099	0.079558	0.998356
Nt5c3l	68106	-0.29511	0.998356
9430008C03Rik	68108	-0.6269	0.998356
Sdccag3	68112	0.303977	0.998356
Mum1	68114	-0.09326	0.998356
9430016H08Rik	68115	-0.02437	0.998356
Apool	68117	-0.42396	0.998356
9430023L20Rik	68118	0.332804	0.998356
Cmtm3	68119	-0.20529	0.998356
Cep70	68121	-0.44685	0.998356
Fahd2a	68126	0.782569	0.998356
C030044B11Rik	68128	-0.07532	0.998356
Gcsh	68133	0.156492	0.998356
Upf3b	68134	-0.04118	0.998356
Eif3h	68135	0.366754	0.998356
Kdelr1	68137	-0.07086	0.998356
Tigd2	68140	-0.14308	0.998356
Ino80	68142	0.152484	0.998356
Etaa1	68145	-0.18124	0.998356
Arl13b	68146	0.019421	0.998356
Gar1	68147	0.020587	0.998356
Fam133b	68152	-0.10067	0.998356
Gtf2e2	68153	8.71039	0.998356
Stx19	68159	0.959678	0.998356
A930005H10Rik	68161	0.463357	0.998356
Fdx1l	68165	-1.04011	0.998356
Ebpl	68177	-0.10338	0.998356
Cgnl1	68178	0.441729	0.998356
Hyi	68180	0.49321	0.998356
Bcas2	68183	-0.04085	0.998356
Denr	68184	-0.06595	0.998356
Chchd8	68185	-0.41117	0.998356

Fam135a	68187	0.101845	0.998356
Sympk	68188	0.006326	0.998356
Leprotl1	68192	0.039822	0.998356
Rpl24	68193	0.207533	0.998356
Ndufb4	68194	0.259974	0.998356
Rnaset2b	68195	-0.11335	0.998356
Hsbp1	68196	0.099265	0.998356
Ndufc2	68197	0.285404	0.998356
Ndufb2	68198	-0.34953	0.998356
Ccdc34	68201	-0.32904	0.998356
Ndufa5	68202	-0.18743	0.998356
Urm1	68205	0.194109	0.998356
Rnaseh2c	68209	-0.33314	0.998356
Tmbim4	68212	0.156002	0.998356
Fam98b	68215	-0.45522	0.998356
AI846148	68229	1.01576	0.998356
1700102H20Rik	68230	-0.41568	0.998356
Rpa3	68240	0.284135	0.998356
Fam195a	68241	0.239689	0.998356
Fam96a	68250	0.153664	0.998356
5430437P03Rik	68251	0.123083	0.998356
Ift80	68259	-0.30741	0.998356
Trmt12	68260	-0.33851	0.998356
Agpat4	68262	0.40439	0.998356
Pdhb	68263	0.100054	0.998356
Slc25a22	68267	0.10681	0.998356
Zdhhc21	68268	0.147808	0.998356
Rbm28	68272	-0.18293	0.998356
Pomgnt1	68273	-0.01509	0.998356
Rpa1	68275	0.082351	0.998356
Toe1	68276	-0.00735	0.998356
2310057M21Rik	68277	-0.11285	0.998356
Ddx39	68278	-0.08744	0.998356
Mcoln2	68279	0.287965	0.998356
C630043F03Rik	68285	-0.22567	0.998356
Mto1	68291	-0.70963	0.998356
Stt3b	68292	0.10905	0.998356
Mfsd10	68294	0.381853	0.998356
0610011L14Rik	68295	-0.01981	0.998356
Ncapd2	68298	-0.13764	0.998356
Vps53	68299	-0.07308	0.998356
Fam114a1	68303	-0.09483	0.998356
Kdelc2	68304	-0.23633	0.998356
4930565N06Rik	68306	-0.58844	0.998356
Zmym1	68310	-0.51864	0.998356
Gstm7	68312	-0.41306	0.998356
Apoo	68316	-0.55931	0.998356

Nudt22	68323	0.283877	0.998356
0610007P22Rik	68327	-0.24709	0.998356
Rab13	68328	0.122927	0.998356
Sdhaf1	68332	0.112957	0.998356
Golt1a	68338	-0.11103	0.998356
Ccdc88c	68339	0.197378	0.998356
Sirt5	68346	0.092702	0.998356
0610011F06Rik	68347	0.127091	0.998356
Ndufs3	68349	0.44988	0.998356
Mul1	68350	-0.08261	0.998356
2010204K13Rik	68355	1.57063	0.998356
0610030E20Rik	68364	0.07605	0.998356
Rab14	68365	-0.11939	0.998356
Tmem129	68366	-0.11077	0.998356
Ndufa8	68375	-0.38904	0.998356
Ciz1	68379	-0.26279	0.998356
Tlcd1	68385	-0.12745	0.998356
G6pc3	68401	-0.13152	0.998356
Sycn	68416	0.010089	0.998356
Ankrd13a	68420	0.332145	0.998356
Lmbrd1	68421	0.368557	0.998356
Slc39a13	68427	0.119626	0.998356
Fbxl15	68431	0.788891	0.998356
Rpl34	68436	-0.58509	0.998356
Rraga	68441	0.108365	0.998356
Tbc1d10b	68449	0.231854	0.998356
Ppp1r14a	68458	0.70495	0.998356
Mrpl14	68463	-0.13263	0.998356
Adipor2	68465	0.31529	0.998356
Ly6g6c	68468	0.049362	0.998356
Tmem126b	68472	-0.16255	0.998356
Mob1b	68473	-0.09744	0.998356
Ssna1	68475	0.027115	0.998356
Rmnd5a	68477	0.207743	0.998356
Phf5a	68479	-0.43072	0.998356
1110007C09Rik	68480	0.556968	0.998356
Mpzl1	68481	-0.29901	0.998356
Zfp579	68490	0.250745	0.998356
Ndufaf4	68493	-0.28356	0.998356
1110018G07Rik	68497	-0.09352	0.998356
Mrpl53	68499	-0.11488	0.998356
Nsmce2	68501	0.080083	0.998356
1110014N23Rik	68505	0.316124	0.998356
Ints1	68510	0.138262	0.998356
Tomm5	68512	0.316214	0.998356
Efha1	68514	0.26136	0.998356
Eml1	68519	0.299395	0.998356

Zfyve21	68520	-0.17196	0.998356
Fam189b	68521	-0.6577	0.998356
Fam96b	68523	0.369748	0.998356
Wipf2	68524	0.276724	0.998356
1110017F19Rik	68528	0.66546	0.998356
Mphosph6	68533	-0.026	0.998356
Tmem109	68539	-0.17903	0.998356
2310036O22Rik	68544	0.423882	0.998356
Sgol2	68549	-0.21434	0.998356
1110002N22Rik	68550	0.299213	0.998356
1110003E01Rik	68552	0.134055	0.998356
1110001A16Rik	68554	1.70426	0.998356
Uckl1	68556	-0.10239	0.998356
Ankra2	68558	-0.00595	0.998356
Pdrg1	68559	-0.35161	0.998356
Dpm3	68563	-0.44858	0.998356
Nufip2	68564	0.222345	0.998356
Mrps18a	68565	0.103291	0.998356
Cgref1	68567	-0.33082	0.998356
Ict1	68572	-0.46732	0.998356
Hbxip	68576	-0.18854	0.998356
Tmed10	68581	0.137482	0.998356
Rtn4	68585	0.670317	0.998356
Mocos	68591	0.40271	0.998356
Syf2	68592	-0.1446	0.998356
1110021J02Rik	68597	-0.11318	0.998356
Dnajc8	68598	-0.00661	0.998356
Pmvk	68603	-0.14315	0.998356
Ppm1f	68606	-0.29482	0.998356
Serhl	68607	0.442369	0.998356
Mrpl28	68611	0.534932	0.998356
Ube2c	68612	0.105978	0.998356
Letmd1	68614	-0.24063	0.998356
1110012J17Rik	68617	-0.27893	0.998356
1110012L19Rik	68618	-0.237	0.998356
Elac2	68626	-0.45639	0.998356
Fbxw9	68628	0.256914	0.998356
Cryl1	68631	0.351514	0.998356
Tm2d3	68634	0.285015	0.998356
Fahd1	68636	-0.17323	0.998356
Tmem216	68642	-0.19451	0.998356
Abhd14a	68644	-0.19993	0.998356
Tab2	68652	0.062672	0.998356
Samm50	68653	-0.10165	0.998356
Fndc1	68655	0.565778	0.998356
Trpm4	68667	0.325671	0.998356
Pcyt2	68671	0.287256	0.998356

Fam172a	68675	0.243949	0.998356
Slc44a2	68682	1.21637	0.998356
1110028F11Rik	68690	0.098069	0.998356
1110028C15Rik	68691	-0.10174	0.998356
Hnrnpul2	68693	-0.18983	0.998356
Hddc3	68695	-0.81788	0.998356
Rere	68703	-0.0995	0.998356
Gtf2f2	68705	0.042048	0.998356
Rabl2	68708	-0.08395	0.998356
Ifitm1	68713	-0.21101	0.998356
Rnf166	68718	0.091418	0.998356
1110032A03Rik	68721	0.061882	0.998356
Arl8a	68724	0.260002	0.998356
Trp53inp2	68728	0.049585	0.998356
Trim37	68729	-0.25867	0.998356
Dus1l	68730	0.181486	0.998356
Rbfa	68731	0.161269	0.998356
Lrrc16a	68732	0.173823	0.998356
Smek1	68734	1.20142	0.998356
Mrps18c	68735	-0.45686	0.998356
1110034B05Rik	68736	-0.65632	0.998356
Acss1	68738	-0.05189	0.998356
Tmem219	68742	0.070745	0.998356
Anln	68743	-0.12928	0.998356
Zfp740	68744	0.348831	0.998356
Rreb1	68750	-0.34146	0.998356
Cgrrf1	68755	0.361189	0.998356
Abhd11	68758	-0.32112	0.998356
1110038B12Rik	68763	0.483861	0.998356
Wash	68767	0.238353	0.998356
Phtf2	68770	-0.06925	0.998356
Ms4a6d	68774	0.282601	0.998356
Taf11	68776	0.059382	0.998356
Tmem53	68777	0.131118	0.998356
1110038D17Rik	68778	0.463532	0.998356
Trmt61b	68789	-0.31781	0.998356
Tmem214	68796	-0.16778	0.998356
Pdgfrl	68797	0.245083	0.998356
Rgmb	68799	0.004317	0.998356
Elovl5	68801	0.135373	0.998356
Dock5	68813	0.200403	0.998356
Btbd10	68815	0.556528	0.998356
Ppil1	68816	0.05836	0.998356
Ddi2	68817	-0.1359	0.998356
Zfand2b	68818	-0.49987	0.998356
1110057K04Rik	68832	0.135069	0.998356
Pdcl3	68833	-0.03182	0.998356

Mrpl52	68836	0.011961	0.998356
Foxk2	68837	0.202216	0.998356
Ankrd46	68839	-0.56698	0.998356
1110054M08Rik	68841	-0.39329	0.998356
Tulp4	68842	0.075789	0.998356
Pih1d1	68845	-0.1909	0.998356
1190002N15Rik	68861	0.654859	0.998356
Arv1	68865	0.412807	0.998356
Rnf122	68867	-0.70922	0.998356
Klhdc9	68874	0.21167	0.998356
Maf1	68877	-1.08529	0.998356
Prpf6	68879	0.310219	0.998356
Gkn3	68888	-1.07984	0.998356
Ubac2	68889	-0.24783	0.998356
Cd177	68891	-0.17579	0.998356
Disp1	68897	0.034825	0.998356
Abhd13	68904	0.610968	0.998356
Pygo2	68911	0.068276	0.998356
Vars2	68915	0.060434	0.998356
Cdkal1	68916	0.049495	0.998356
Hint2	68917	0.455821	0.998356
1190005I06Rik	68918	1.35825	0.998356
1110065P20Rik	68920	0.739724	0.998356
Rpap1	68925	-0.95379	0.998356
Ubap2	68926	-0.48313	0.998356
Ptcd2	68927	-0.16179	0.998356
Mospd3	68929	0.108434	0.998356
Fam165b	68936	0.530891	0.998356
Aspscr1	68938	-0.26325	0.998356
Rasl11b	68939	0.871676	0.998356
Chmp2b	68942	0.459849	0.998356
Tmco1	68944	0.283458	0.998356
1500011H22Rik	68948	0.018289	0.998356
1500012F01Rik	68949	0.674392	0.998356
Chmp2a	68953	0.227756	0.998356
Phkg2	68961	0.5342	0.998356
1500010J02Rik	68964	-0.40933	0.998356
Ngdn	68966	-0.35877	0.998356
Eif1b	68969	-0.3231	0.998356
Dcaf12	68970	0.110915	0.998356
1500001M20Rik	68971	0.110386	0.998356
Tatdn3	68972	1.11581	0.998356
Med27	68975	-0.0999	0.998356
Haghl	68977	-0.14464	0.998356
Nol11	68979	1.07115	0.998356
Wdr53	68980	-0.41415	0.998356
Snrpa1	68981	-0.4928	0.998356

1500015A07Rik	68982	-0.05416	0.998356
Prpf31	68988	0.021219	0.998356
Ssu72	68991	0.260921	0.998356
Mcts1	68995	-0.08149	0.998356
Anapc10	68999	-0.227	0.998356
6330418K02Rik	69004	0.182363	0.998356
Cab39l	69008	0.171024	0.998356
Thap7	69009	0.099398	0.998356
Anapc13	69010	-0.08662	0.998356
Spcs1	69019	-0.03453	0.998356
Zfp707	69020	-0.19368	0.998356
Snx15	69024	-0.24962	0.998356
Mitd1	69028	0.073922	0.998356
1500032L24Rik	69029	0.144412	0.998356
Zdhhc3	69035	0.297942	0.998356
Zg16	69036	0.42546	0.998356
Isca1	69046	0.11288	0.998356
Atp2c2	69047	-0.18181	0.998356
Slc30a5	69048	0.182566	0.998356
Pycr2	69051	-0.15776	0.998356
1810013L24Rik	69053	0.213244	0.998356
Chac1	69065	0.175991	0.998356
1810011O10Rik	69068	-0.0689	0.998356
Tmem97	69071	-0.13678	0.998356
Ebna1bp2	69072	-0.42662	0.998356
1810019J16Rik	69073	-0.2299	0.998356
Triap1	69076	-0.12768	0.998356
Psmd11	69077	-0.15831	0.998356
Gmppa	69080	-0.03325	0.998356
Zc3h15	69082	-0.11447	0.998356
Zcchc9	69085	0.089399	0.998356
Oxa1l	69089	-0.34859	0.998356
Ascc1	69090	-0.10087	0.998356
Vps26b	69091	0.097106	0.998356
Tmem160	69094	0.059497	0.998356
Trim15	69097	0.520499	0.998356
Ydjc	69101	0.84819	0.998356
Stoml1	69106	1.00404	0.998356
Fam58b	69109	-0.23255	0.998356
Alkbh3	69113	0.032672	0.998356
Ubr4	69116	0.155846	0.998356
Cnot8	69125	-0.33122	0.998356
1810022K09Rik	69126	0.673089	0.998356
Pex11c	69129	0.641664	0.998356
Cdk12	69131	-0.31568	0.998356
Tusc1	69136	0.682543	0.998356
Gsdmd	69146	0.161429	0.998356

Snx4	69150	0.050757	0.998356
Lzic	69151	-0.45637	0.998356
1810030O07Rik	69155	0.264064	0.998356
Comtd1	69156	0.012757	0.998356
Manbal	69161	0.254099	0.998356
Sec31a	69162	-0.33221	0.998356
Mrpl44	69163	0.033165	0.998356
1810026B05Rik	69170	-0.26101	0.998356
1810031K17Rik	69171	0.42499	0.998356
Snx5	69178	-0.4123	0.998356
Tmem110	69179	0.204545	0.998356
Dyrk2	69181	0.047028	0.998356
Dtwd1	69185	0.125983	0.998356
1810027O10Rik	69186	0.722536	0.998356
Erp27	69187	0.018713	0.998356
Mll5	69188	-0.01041	0.998356
Dym	69190	0.113502	0.998356
Dhx16	69192	-0.17713	0.998356
Ptms	69202	0.250353	0.998356
2010016I18Rik	69206	0.00031	0.998356
Srsf11	69207	0.271803	0.998356
Ccdc23	69216	0.413733	0.998356
Carkd	69225	-0.2212	0.998356
Snx24	69226	-0.07625	0.998356
2810407C02Rik	69227	0.166009	0.998356
Zfp746	69228	0.035773	0.998356
Qrich1	69232	0.079467	0.998356
Zfp688	69234	0.479305	0.998356
Gtpbp4	69237	-0.40745	0.998356
Polr2d	69241	0.247178	0.998356
Zfp397	69256	-0.37101	0.998356
Elf2	69257	0.055132	0.998356
Kctd5	69259	0.261882	0.998356
Ing2	69260	0.252508	0.998356
Rfc3	69263	0.329287	0.998356
Scnm1	69269	0.391071	0.998356
Gins1	69270	-0.05318	0.998356
Ctdspl	69274	0.113116	0.998356
Sec62	69276	-0.20056	0.998356
3300002I08Rik	69277	-0.34683	0.998356
1700001L05Rik	69291	-0.17537	0.998356
Dcps	69305	0.562359	0.998356
Slc16a13	69309	0.136056	0.998356
Slc38a4	69354	-0.44263	0.998356
Glrx2	69367	-0.28639	0.998356
Mocs3	69372	0.022824	0.998356
Hist1h4h	69386	0.54338	0.998356

Dnajc17	69408	-0.3206	0.998356
Snhg10	69434	0.125005	0.998356
Prss56	69453	0.525344	0.998356
Clic3	69454	-0.77266	0.998356
Commd10	69456	-0.10804	0.998356
Ubl7	69459	0.814274	0.998356
Tmem127	69470	0.357169	0.998356
2300009A05Rik	69478	0.695643	0.998356
Ttc9	69480	0.245132	0.998356
Nup35	69482	-0.04658	0.998356
2310003L22Rik	69487	0.42822	0.998356
Zfp932	69504	0.589419	0.998356
Esam	69524	-0.29238	0.998356
Mrps9	69527	0.087034	0.998356
Avpi1	69534	0.18008	0.998356
2310004N24Rik	69535	0.181369	0.998356
Dnase1l1	69537	0.276348	0.998356
Antxr1	69538	-0.31287	0.998356
Wdr5b	69544	0.092176	0.998356
Mapk1ip1	69546	-0.73067	0.998356
2310009B15Rik	69549	0.951813	0.998356
Bst2	69550	-0.25864	0.998356
2310022B05Rik	69551	-0.02549	0.998356
Klhdc2	69554	0.400993	0.998356
Bod1	69556	0.306748	0.998356
Cdk13	69562	0.216285	0.998356
Vkorc1l1	69568	0.934672	0.998356
Hilpda	69573	0.165607	0.998356
Cmbl	69574	0.163055	0.998356
Fastkd3	69577	-0.05399	0.998356
Rhou	69581	0.267898	0.998356
Plekhm2	69582	0.267769	0.998356
Tnfsf13	69583	-0.45105	0.998356
Pcgf3	69587	0.006301	0.998356
Gpx8	69590	-0.17187	0.998356
Odam	69592	0.35928	0.998356
2310035K24Rik	69596	0.268534	0.998356
Afg3l2	69597	0.022048	0.998356
Dab2ip	69601	-0.08513	0.998356
Mtfmt	69606	-0.0664	0.998356
Sec24d	69608	0.042633	0.998356
2310037I24Rik	69612	-0.07232	0.998356
Pitrm1	69617	0.132602	0.998356
Fam89a	69627	0.234491	0.998356
Arhgef12	69632	-0.05609	0.998356
Clybl	69634	-0.70721	0.998356
Dapk1	69635	-0.19494	0.998356

Exosc8	69639	-1.92736	0.998356
Fam83g	69640	-0.34678	0.998356
Wdr20a	69641	-0.10206	0.998356
2310069B03Rik	69652	0.029703	0.998356
Dctn2	69654	0.199881	0.998356
Pir	69656	-0.07054	0.998356
Tmbim1	69660	0.454539	0.998356
2310061I04Rik	69662	0.059859	0.998356
Ddx51	69663	-0.39227	0.998356
2310043J07Rik	69665	-0.04687	0.998356
Psmg4	69666	-1.04059	0.998356
Ccdc115	69668	-0.1031	0.998356
Txndc15	69672	0.262444	0.998356
Mif4gd	69674	0.123068	0.998356
Pxdn	69675	-0.47753	0.998356
2310044H10Rik	69683	-0.05845	0.998356
Aarsd1	69684	-0.08905	0.998356
Hddc2	69692	0.833142	0.998356
Pof1b	69693	0.3441	0.998356
Camsap3	69697	-0.15935	0.998356
Slc52a3	69698	0.410636	0.998356
Ndufaf1	69702	-0.23948	0.998356
2410017P09Rik	69709	0.18877	0.998356
Arap1	69710	-0.25876	0.998356
Pin4	69713	-0.27087	0.998356
Tfpt	69714	0.283811	0.998356
Trip13	69716	0.01372	0.998356
Cad	69719	-0.33798	0.998356
Nkiras1	69721	0.121972	0.998356
Rpain	69723	0.068237	0.998356
Rnaseh2a	69724	0.138241	0.998356
Smyd3	69726	-0.28344	0.998356
Usp46	69727	0.124519	0.998356
Gemin7	69731	-0.13028	0.998356
Nup37	69736	0.022919	0.998356
Ttl	69737	-0.19124	0.998356
Dph5	69740	0.287039	0.998356
Tm2d2	69742	-0.0839	0.998356
Casz1	69743	0.205267	0.998356
Pold4	69745	0.062121	0.998356
Zswim7	69747	0.076259	0.998356
Aldh16a1	69748	0.193539	0.998356
2410004N09Rik	69749	0.06217	0.998356
Zfp511	69752	0.145901	0.998356
Fbxo7	69754	0.073245	0.998356
Leng1	69757	-0.01835	0.998356
Tnfaip8l2	69769	-0.57967	0.998356

1600002K03Rik	69770	-0.40745	0.998356
1810019D21Rik	69771	-0.14459	0.998356
1810026J23Rik	69773	-0.21845	0.998356
Smap2	69780	-0.00182	0.998356
1500009L16Rik	69784	0.141632	0.998356
Tprkb	69786	0.366677	0.998356
Med30	69790	-0.01352	0.998356
Med6	69792	0.356621	0.998356
Cox11	69802	0.002	0.998356
Tmem147	69804	0.26642	0.998356
Slc39a11	69806	-0.45742	0.998356
Trim32	69807	-1.01635	0.998356
2010001M06Rik	69812	1.01603	0.998356
Prss32	69814	0.27511	0.998356
Krtcap3	69815	-0.19482	0.998356
Mterfd2	69821	0.258738	0.998356
Fyttd1	69823	0.028992	0.998356
Glod5	69824	-0.59012	0.998356
Ms4a10	69826	0.253134	0.998356
Polr2f	69833	-0.27255	0.998356
Rab43	69834	-0.28099	0.998356
Pcgf1	69837	0.42124	0.998356
Wnk4	69847	0.362332	0.998356
Eif1ad	69860	-0.35393	0.998356
2010003K11Rik	69861	0.275506	0.998356
Ttc39b	69863	-0.0732	0.998356
1810065E05Rik	69864	-0.42103	0.998356
A1cf	69865	-0.14177	0.998356
Polr3gl	69870	-0.34124	0.998356
Ppp1r35	69871	0.132019	0.998356
Ndufa11	69875	0.247671	0.998356
Thap3	69876	0.222297	0.998356
Snrpf	69878	-0.11295	0.998356
2010321M09Rik	69882	0.495593	0.998356
2610002D18Rik	69885	-0.32181	0.998356
Zfp219	69890	-0.51359	0.998356
2010305A19Rik	69893	-0.13231	0.998356
2010107G23Rik	69894	0.336782	0.998356
Snhg8	69895	0.179011	0.998356
Mfsd11	69900	0.09405	0.998356
Mrto4	69902	0.131183	0.998356
Slc25a32	69906	-0.00729	0.998356
Nup43	69912	-0.38738	0.998356
Obfc2b	69917	0.165951	0.998356
Polr2i	69920	0.577978	0.998356
Vrk2	69922	0.320767	0.998356
Agk	69923	0.009444	0.998356

Apitd1	69928	-0.21583	0.998356
Zfp715	69930	0.089623	0.998356
Rg9mtd3	69934	-0.36198	0.998356
Exoc1	69940	-0.28164	0.998356
2810408I11Rik	69941	-0.96299	0.998356
Rnf113a1	69942	0.027518	0.998356
2810025M15Rik	69953	0.249458	0.998356
Fars2	69955	0.149444	0.998356
Ptcd3	69956	0.275667	0.998356
Cdc16	69957	-0.18216	0.998356
2810432D09Rik	69961	0.004549	0.998356
2810422O20Rik	69962	-0.22578	0.998356
Galk2	69976	-0.20412	0.998356
Tmem30a	69981	0.079214	0.998356
Chn2	69993	-0.1195	0.998356
Rsc1a1	69994	0.48601	0.998356
Ccdc21	70012	0.066126	0.998356
Ino80b	70020	-0.57001	0.998356
Mcm10	70024	-0.48344	0.998356
Acot7	70025	0.099735	0.998356
Dopey2	70028	-3.04304	0.998356
Cmtm8	70031	0.235668	0.998356
2700023E23Rik	70036	0.112188	0.998356
Tut1	70044	-0.2205	0.998356
2610528A11Rik	70045	0.494693	0.998356
Trnt1	70047	-1.02463	0.998356
Prpf4	70052	-0.18396	0.998356
Degs2	70059	0.184777	0.998356
Nol7	70078	0.030032	0.998356
2210010C17Rik	70080	0.224214	0.998356
2210404O09Rik	70081	0.493967	0.998356
Lysmd2	70082	-0.06367	0.998356
Meaf6	70088	-0.5594	0.998356
Ube2q1	70093	0.210299	0.998356
Sash1	70097	0.200515	0.998356
Smc4	70099	-0.34761	0.998356
Cyp4f16	70101	0.268532	0.998356
Znhit1	70103	-0.17157	0.998356
Ifi35	70110	0.121563	0.998356
Yars2	70120	0.026476	0.998356
Mllt3	70122	0.024752	0.998356
2210013O21Rik	70123	-0.49029	0.998356
Slc44a4	70129	0.522521	0.998356
2210417A02Rik	70138	-0.47044	0.998356
Lrch3	70144	0.122087	0.998356
Mettl7a1	70152	0.272978	0.998356
Vps36	70160	-0.22501	0.998356

2210415F13Rik	70163	0.730442	0.998356
Fam108c	70178	0.509363	0.998356
Fam162a	70186	-0.26667	0.998356
Taco1	70207	-0.5249	0.998356
Med23	70208	0.590757	0.998356
Tmem143	70209	-0.1796	0.998356
Kif18b	70218	-0.11428	0.998356
Nars	70223	-0.52251	0.998356
Ppil3	70225	0.498498	0.998356
Gorasp2	70231	0.023611	0.998356
Cd2bp2	70233	0.073111	0.998356
Poc1a	70235	0.01742	0.998356
Rnf168	70238	-0.1795	0.998356
Gtf3c5	70239	0.105592	0.998356
Ufsp1	70240	-0.40756	0.998356
Psmd1	70247	-0.11271	0.998356
Dazap1	70248	0.075244	0.998356
2010107E04Rik	70257	0.659939	0.998356
2010110P09Rik	70261	0.305521	0.998356
Ccbl1	70266	-0.25576	0.998356
Rpf1	70285	0.156432	0.998356
Afap1	70292	-0.18524	0.998356
Rnf126	70294	0.252595	0.998356
Tbc1d13	70296	0.039171	0.998356
Gcc2	70297	0.009296	0.998356
Fuz	70300	-0.30624	0.998356
Plscr3	70310	0.963082	0.998356
2510012J08Rik	70312	-0.33134	0.998356
Rabep2	70314	0.007288	0.998356
Hdac8	70315	0.442375	0.998356
Arl16	70317	0.183349	0.998356
Pigw	70325	0.916488	0.998356
Cd3eap	70333	0.049371	0.998356
Reep6	70335	-0.19039	0.998356
Ube2cbp	70348	-0.64548	0.998356
Copb1	70349	0.211029	0.998356
Basp1	70350	0.35709	0.998356
Ppp4r1	70351	0.09172	0.998356
Secisbp2l	70354	0.371017	0.998356
Gprc5c	70355	-0.4533	0.998356
St13	70356	0.301144	0.998356
Steap1	70358	-0.55387	0.998356
Gtpbp3	70359	-0.0619	0.998356
Lman1	70361	0.008677	0.998356
Bag5	70369	-0.19667	0.998356
1700020O03Rik	70373	0.062991	0.998356
Mospd1	70380	0.012014	0.998356

Tecpr1	70381	0.215383	0.998356
Cox10	70383	-0.20132	0.998356
Ccdc99	70385	0.116865	0.998356
Ttc9c	70387	-0.24779	0.998356
Kptn	70394	-0.00169	0.998356
Asnsd1	70396	-0.04768	0.998356
Tmem70	70397	-2.69059	0.998356
Polr3f	70408	0.170869	0.998356
2810408A11Rik	70419	0.634573	0.998356
2610034B18Rik	70420	-0.03067	0.998356
Ints2	70422	-0.13638	0.998356
Csnk1g3	70425	0.262749	0.998356
Mier2	70427	0.126892	0.998356
Polr3b	70428	0.12963	0.998356
Tbce	70430	0.333548	0.998356
Rufy2	70432	0.229163	0.998356
Inf2	70435	-0.26517	0.998356
Taf15	70439	-0.47843	0.998356
Unc13d	70450	0.336139	0.998356
Dhrs13	70451	0.059833	0.998356
Cenpl	70454	-0.77522	0.998356
Brp44	70456	0.05304	0.998356
Crtc3	70461	0.163571	0.998356
Wdr77	70465	-0.18155	0.998356
Ckap2l	70466	-0.20786	0.998356
Rprd1b	70470	-0.04252	0.998356
Atad2	70472	-0.17059	0.998356
Mipep	70478	-0.15792	0.998356
Slc35d2	70484	0.748205	0.998356
Atp6ap2	70495	-0.19618	0.998356
Arhgap17	70497	0.246245	0.998356
Bbx	70508	0.109551	0.998356
Rnf167	70510	0.234638	0.998356
Fam86	70511	0.007683	0.998356
Stambp	70527	0.181308	0.998356
Btf3l4	70533	0.273589	0.998356
Zdhhc2	70546	0.104944	0.998356
Tln2	70549	0.2583	0.998356
Tmtc4	70551	0.187078	0.998356
Slc25a33	70556	0.72588	0.998356
Wars2	70560	0.134655	0.998356
Txndc16	70561	0.019356	0.998356
5730469M10Rik	70564	-0.03966	0.998356
5730455O13Rik	70567	-0.04835	0.998356
Cpne3	70568	-0.06108	0.998356
Ipo5	70572	0.022803	0.998356
Tbccd1	70573	0.057086	0.998356

Cpm	70574	0.291076	0.998356
Gfod2	70575	-0.03803	0.998356
Zc3h11a	70579	-0.12354	0.998356
Pak4	70584	-0.06661	0.998356
5730455P16Rik	70591	0.393464	0.998356
5730457N03Rik	70593	0.637405	0.998356
Ssfa2	70599	0.052916	0.998356
Ecd	70601	-0.25054	0.998356
Mutyh	70603	-0.70004	0.998356
Dnajb14	70604	0.357111	0.998356
Zdhhc24	70605	-0.67157	0.998356
Fbxo33	70611	0.303733	0.998356
5730494N06Rik	70612	0.188169	0.998356
Sugp1	70616	-0.1343	0.998356
5730508B09Rik	70617	0.441243	0.998356
Ube2v2	70620	-0.54209	0.998356
Med26	70625	-0.08917	0.998356
Dcp2	70640	-0.21178	0.998356
Oip5	70645	0.40649	0.998356
Naa30	70646	0.361388	0.998356
Zcchc8	70650	0.055602	0.998356
Sik3	70661	-0.15298	0.998356
Vcpip1	70675	0.032945	0.998356
Fam175a	70681	-0.27921	0.998356
Utp20	70683	-0.16912	0.998356
Dusp16	70686	-0.09345	0.998356
3830403N18Rik	70691	-4.59962	0.998356
Gpr125	70693	0.346754	0.998356
Nup205	70699	-0.15166	0.998356
Nipal1	70701	0.580945	0.998356
Cgn	70737	0.003113	0.998356
Kdsr	70750	0.039066	0.998356
Ptplb	70757	0.135178	0.998356
Prpf3	70767	-0.28985	0.998356
Nolc1	70769	-0.51511	0.998356
Dennd1c	70785	-0.24261	0.998356
Ubr5	70790	0.285059	0.998356
Hars2	70791	0.07944	0.998356
Zdhhc1	70796	-0.5676	0.998356
Ankib1	70797	0.054056	0.998356
Cep192	70799	-0.09415	0.998356
Pwwp2a	70802	0.130068	0.998356
Pgrmc2	70804	0.183306	0.998356
Arrdc2	70807	0.215028	0.998356
4632415L05Rik	70808	-0.18605	0.998356
Hmgxb4	70823	-0.02291	0.998356
Trak2	70827	0.0114	0.998356

Ccdc93	70829	0.272594	0.998356
Spag9	70834	-0.30109	0.998356
Prss22	70835	-0.28473	0.998356
4921506M07Rik	70846	0.335195	0.998356
Ints10	70885	0.163447	0.998356
Ttll7	70892	-0.01638	0.998356
Cdkn2aip	70925	-0.24083	0.998356
Nol8	70930	-0.41276	0.998356
Pgm2l1	70974	-0.25366	0.998356
4931406C07Rik	70984	0.183288	0.998356
Phf6	70998	-0.37691	0.998356
Naa40	70999	-0.29483	0.998356
Pcgf6	71041	-0.28888	0.998356
Zfp597	71063	0.004103	0.998356
Arhgap19	71085	-0.1774	0.998356
Atoh8	71093	0.230271	0.998356
4933407H18Rik	71101	0.037459	0.998356
Gpr39	71111	0.535575	0.998356
Stx18	71116	0.075904	0.998356
Zfp689	71131	-0.43849	0.998356
Oxsm	71147	0.051639	0.998356
Mier1	71148	0.145468	0.998356
Eri2	71151	-0.04865	0.998356
Nbas	71169	0.089034	0.998356
Nipbl	71175	-0.27048	0.998356
4933424B01Rik	71177	-0.21081	0.998356
Dlg5	71228	-0.2952	0.998356
Acn9	71238	-0.18799	0.998356
Osbpl7	71240	0.158949	0.998356
Spata24	71242	0.448398	0.998356
Lrrfip2	71268	2.2278	0.998356
Slc29a3	71279	-0.16387	0.998356
Arhgap26	71302	-0.16103	0.998356
Wbscr25	71304	1.04583	0.998356
Mfap3l	71306	-0.48652	0.998356
Tbc1d9	71310	-0.5225	0.998356
Rcbtb1	71330	-0.07661	0.998356
Rbks	71336	0.507466	0.998356
Riok1	71340	-0.28532	0.998356
Ano9	71345	0.259124	0.998356
Aifm2	71361	0.751814	0.998356
Pdss2	71365	-0.01201	0.998356
Arid5b	71371	-0.24846	0.998356
Foxn3	71375	0.180613	0.998356
Pex1	71382	-0.35268	0.998356
Chd6	71389	0.037884	0.998356
Kctd6	71393	1.00013	0.998356

Fmnl2	71409	-0.01333	0.998356
Arhgap21	71435	0.069005	0.998356
Flrt3	71436	0.337225	0.998356
Wrb	71446	0.00798	0.998356
Tmem80	71448	-0.19119	0.998356
Mettl13	71449	-0.44165	0.998356
Ankrd40	71452	0.264982	0.998356
Bcor	71458	-0.04845	0.998356
Ptk7	71461	0.367388	0.998356
Usp19	71472	-0.47366	0.998356
Ppp6r2	71474	0.379702	0.998356
Alpk1	71481	0.014225	0.998356
Zfp935	71508	0.078726	0.998356
Sfpq	71514	-0.1427	0.998356
9030624J02Rik	71517	-0.08113	0.998356
Grap	71520	-0.12333	0.998356
Pds5a	71521	0.02674	0.998356
Ggt6	71522	-0.18048	0.998356
8430429K09Rik	71523	0.827887	0.998356
Kazn	71529	-0.37153	0.998356
Fbxo9	71538	1.02962	0.998356
Arhgap42	71544	0.287403	0.998356
Izumo4	71564	0.434938	0.998356
9030425E11Rik	71566	0.669902	0.998356
Mcm9	71567	0.008297	0.998356
Sval1	71578	0.554511	0.998356
9130008F23Rik	71583	0.105402	0.998356
Ifih1	71586	0.501732	0.998356
Zfp251	71591	-0.37769	0.998356
Pogk	71592	-0.18917	0.998356
Myo1e	71602	0.270501	0.998356
Snx20	71607	0.025666	0.998356
Tradd	71609	-0.03966	0.998356
9130011E15Rik	71617	0.282418	0.998356
Arl14	71619	0.33979	0.998356
4930413F20Rik	71637	-0.46989	0.998356
Zfp949	71640	0.249075	0.998356
Optn	71648	0.184446	0.998356
4930506M07Rik	71653	0.0677	0.998356
Rarres2	71660	1.8851	0.998356
Mettl7b	71664	0.197269	0.998356
Fuca1	71665	0.224448	0.998356
0610007L01Rik	71667	0.186069	0.998356
Acy3	71670	0.190944	0.998356
Rnf215	71673	0.338096	0.998356
0610010F05Rik	71675	0.00817	0.998356
Brox	71678	0.214484	0.998356

Atp5h	71679	-0.07451	0.998356
Rbm43	71684	0.194117	0.998356
Pnpt1	71701	-0.11578	0.998356
Cdc5l	71702	-0.11037	0.998356
Armcx3	71703	-0.65225	0.998356
Arhgef3	71704	0.103451	0.998356
Ubiad1	71707	-0.05178	0.998356
Lrrcc1	71710	-0.10153	0.998356
Mus81	71711	0.547012	0.998356
Cdc40	71713	-0.08587	0.998356
Dhx35	71715	0.371467	0.998356
Telo2	71718	-0.27381	0.998356
Osbpl3	71720	0.154049	0.998356
Cic	71722	-0.74384	0.998356
Dhx34	71723	-0.15188	0.998356
Smug1	71726	0.013398	0.998356
Stk11ip	71728	0.261532	0.998356
Rgs12	71729	0.067179	0.998356
Vps11	71732	0.381753	0.998356
Lrwd1	71735	-0.16973	0.998356
Pvrl4	71740	1.23172	0.998356
Ulk3	71742	0.006674	0.998356
Coasy	71743	-0.13299	0.998356
Cul2	71745	0.132071	0.998356
R3hdm2	71750	0.502138	0.998356
Map3k13	71751	-0.08591	0.998356
Gtf3c2	71752	-0.12788	0.998356
C2cd2l	71764	0.081327	0.998356
Klhdc3	71765	-0.33609	0.998356
Raver1	71766	-0.53091	0.998356
Tysnd1	71767	0.15368	0.998356
Ap2b1	71770	-0.01286	0.998356
Plbd2	71772	0.234509	0.998356
Shroom1	71774	0.060847	0.998356
Tha1	71776	-0.16046	0.998356
Ing3	71777	-0.07546	0.998356
Klhl5	71778	0.05026	0.998356
Isyna1	71780	-0.27294	0.998356
Ankle2	71782	0.028164	0.998356
Pdgfd	71785	0.360234	0.998356
Trnau1ap	71787	0.178328	0.998356
Ints12	71793	-0.17063	0.998356
Pitpnc1	71795	-0.40416	0.998356
Ptcd1	71799	-0.03958	0.998356
Plekhf2	71801	0.421361	0.998356
Nup93	71805	-0.15934	0.998356
Tars2	71807	0.422417	0.998356

Ranbp3	71810	-0.42275	0.998356
Rnf180	71816	-0.14092	0.998356
Tmem50a	71817	0.14833	0.998356
Wdr34	71820	0.311849	0.998356
Dcaf7	71833	-0.06615	0.998356
Zbtb43	71834	-0.52218	0.998356
Lancl2	71835	0.388928	0.998356
Phf7	71838	0.018357	0.998356
Osgin1	71839	-0.30576	0.998356
R3hcc1	71843	-0.02535	0.998356
Nupl1	71844	0.023626	0.998356
Syce2	71846	-0.78224	0.998356
Pdia6	71853	-0.25463	0.998356
Gpr160	71862	-1.08776	0.998356
Fbxo30	71865	-0.12498	0.998356
2310007B03Rik	71874	-5.50625	0.998356
Mlf1ip	71876	-0.10823	0.998356
Fam83d	71878	-0.04804	0.998356
2310001A20Rik	71881	-0.04898	0.998356
Coq2	71883	0.059495	0.998356
Chit1	71884	0.790478	0.998356
2310003H01Rik	71885	-0.24894	0.998356
Ppm1j	71887	0.795301	0.998356
Epn3	71889	-0.44445	0.998356
Mad2l2	71890	-0.25053	0.998356
Cdadc1	71891	0.781526	0.998356
Noxo1	71893	-0.24296	0.998356
Lypd6b	71897	0.267296	0.998356
Tmem106b	71900	0.048891	0.998356
Cand1	71902	-0.00753	0.998356
Ces2f	71903	-0.06707	0.998356
Cldn23	71908	0.435511	0.998356
Haus5	71909	-0.40727	0.998356
Ppapdc1b	71910	0.531494	0.998356
Bdh1	71911	-0.46483	0.998356
Tmem79	71913	-0.31691	0.998356
Antxr2	71914	0.41354	0.998356
Dus4l	71916	-0.41431	0.998356
Zcchc24	71918	0.078499	0.998356
Rpap3	71919	0.011223	0.998356
2310047M10Rik	71923	0.351516	0.998356
Itfg1	71927	0.158151	0.998356
Tmem123	71929	0.401838	0.998356
Car13	71934	0.203076	0.998356
Tom1l1	71943	0.581561	0.998356
2310067B10Rik	71947	-0.13634	0.998356
Lass5	71949	0.098654	0.998356

2410016O06Rik	71952	0.105149	0.998356
Suds3	71954	0.090986	0.998356
2400003C14Rik	71955	0.268657	0.998356
Rnf135	71956	-0.38075	0.998356
Cpsf3l	71957	-0.48561	0.998356
Myh14	71960	0.10597	0.998356
Cdca4	71963	0.037055	0.998356
Nkiras2	71966	0.072395	0.998356
Wdr73	71968	-0.15682	0.998356
Zswim1	71971	-0.20977	0.998356
Dnmbp	71972	0.059105	0.998356
Prmt3	71974	-0.39674	0.998356
Ppp2r2a	71978	0.069495	0.998356
Snx10	71982	-0.2203	0.998356
Tmco6	71983	-0.00495	0.998356
Sars2	71984	0.31318	0.998356
Ddx28	71986	0.02755	0.998356
Esco2	71988	-0.56357	0.998356
Rpusd4	71989	0.126798	0.998356
Ddx54	71990	-0.37271	0.998356
Cnn3	71994	-0.25744	0.998356
1500002O20Rik	71997	0.010598	0.998356
Slc25a35	71998	-0.14614	0.998356
Fbxo22	71999	-0.02018	0.998356
Fndc3b	72007	-0.10912	0.998356
Zfyve19	72008	0.37412	0.998356
1600002H07Rik	72016	-0.2245	0.998356
Cyb5r1	72017	-0.4497	0.998356
Fundc1	72018	0.153829	0.998356
Zfp654	72020	-0.09485	0.998356
Slc35f2	72022	0.41383	0.998356
Trmu	72026	0.13457	0.998356
Slc39a4	72027	0.54777	0.998356
Cnpy3	72029	-0.02645	0.998356
Tsc22d2	72033	-0.25254	0.998356
Mccc1	72039	0.047748	0.998356
Cdhr5	72040	0.825585	0.998356
Alkbh4	72041	-0.22177	0.998356
Cotl1	72042	-0.39727	0.998356
Sulf2	72043	-0.94266	0.998356
Urgcp	72046	-0.3067	0.998356
Ddx42	72047	-0.18093	0.998356
Kdelc1	72050	-0.22006	0.998356
Tmub2	72053	0.276392	0.998356
Cyp4f18	72054	0.587856	0.998356
Slc38a10	72055	-0.11268	0.998356
1810055G02Rik	72056	-0.42136	0.998356

Phf10	72057	-0.11874	0.998356
2010111I01Rik	72061	-0.08171	0.998356
Rap2c	72065	0.237931	0.998356
Cnot2	72068	0.425957	0.998356
Anks4b	72074	0.240995	0.998356
Ogfr	72075	0.122216	0.998356
Gcnt3	72077	0.397009	0.998356
2010317E24Rik	72080	-0.11711	0.998356
Mzt2	72083	0.544557	0.998356
Pigx	72084	0.194339	0.998356
Osgepl1	72085	-0.10864	0.998356
Ush1c	72088	-0.12392	0.998356
Entpd8	72090	0.303859	0.998356
Snhg7	72091	-0.17786	0.998356
2010320M18Rik	72093	0.059772	0.998356
Mettl10	72096	-0.03564	0.998356
2010300C02Rik	72097	-0.05949	0.998356
Tmem68	72098	-0.36701	0.998356
Dusp11	72102	0.02883	0.998356
Aplf	72103	0.090649	0.998356
Jmjd8	72106	0.050917	0.998356
Dscc1	72107	-0.67872	0.998356
Ddhd2	72108	-0.02987	0.998356
Ppp1r14d	72112	0.296141	0.998356
Adck1	72113	0.394211	0.998356
Zbed3	72114	-0.18857	0.998356
Naa50	72117	-0.41277	0.998356
Tpx2	72119	0.21642	0.998356
Dennd2d	72121	0.711078	0.998356
Seh1l	72124	0.113125	0.998356
Fam123a	72125	-0.95238	0.998356
2610008E11Rik	72128	0.14598	0.998356
Pex13	72129	-0.05239	0.998356
Wdsub1	72137	-1.5757	0.998356
2610044O15Rik	72139	0.918614	0.998356
Ccdc123	72140	-0.26339	0.998356
Adpgk	72141	-0.51192	0.998356
Slc37a3	72144	0.021574	0.998356
Wdfy3	72145	0.066648	0.998356
Rfc5	72151	-0.24306	0.998356
Zfp157	72154	0.171283	0.998356
Cenpn	72155	-0.79246	0.998356
Pgm2	72157	-0.14846	0.998356
Dhx36	72162	0.152954	0.998356
Thumpd2	72167	-0.53071	0.998356
Trim29	72169	0.148972	0.998356
Chchd4	72170	0.112574	0.998356

Shq1	72171	0.292424	0.998356
Nsun4	72181	-0.08872	0.998356
Snx6	72183	-0.24609	0.998356
2510009E07Rik	72190	0.03871	0.998356
Scaf11	72193	0.018059	0.998356
Fbxl20	72194	-0.0712	0.998356
Supt7l	72195	0.048745	0.998356
Skiv2l2	72198	0.208356	0.998356
Mms19	72199	0.16747	0.998356
Otud6b	72201	-0.07928	0.998356
2610507I01Rik	72203	0.120129	0.998356
Eml2	72205	0.285624	0.998356
Tbc1d5	72238	0.144085	0.998356
1700030K09Rik	72254	-0.12547	0.998356
Tram1	72265	0.1803	0.998356
Lrrc8e	72267	0.286043	0.998356
Cda	72269	0.091586	0.998356
2210404O07Rik	72273	0.497729	0.998356
2200002D01Rik	72275	0.907497	0.998356
Ccpg1	72278	-0.11327	0.998356
Sh2d4a	72281	0.187168	0.998356
Oraov1	72284	0.145195	0.998356
Plekhf1	72287	0.154776	0.998356
Malat1	72289	-0.08141	0.998356
Rusc1	72296	0.173262	0.998356
B3gnt3	72297	0.406533	0.998356
Zfp777	72306	0.095502	0.998356
2510002D24Rik	72307	-0.14676	0.998356
Brf1	72308	-0.0118	0.998356
Fryl	72313	0.023523	0.998356
Cyth4	72318	-0.37403	0.998356
2510003E04Rik	72320	0.035935	0.998356
Xpo5	72322	-0.05099	0.998356
Asb6	72323	-0.12997	0.998356
1300018I17Rik	72325	-0.14971	0.998356
Palld	72333	-0.62631	0.998356
Wdr89	72338	-0.50125	0.998356
2610002I17Rik	72341	-0.06427	0.998356
Usp36	72344	-0.25519	0.998356
Fam123b	72345	0.008351	0.998356
Dusp3	72349	-0.06074	0.998356
Ptar1	72351	-0.09894	0.998356
Ttc4	72354	-0.08495	0.998356
2210021J22Rik	72355	-1.13106	0.998356
2210016L21Rik	72357	-0.24488	0.998356
Ces2g	72361	-0.02651	0.998356
2310045N01Rik	72368	0.240034	0.998356

2610035D17Rik	72386	-0.34968	0.998356
Ripk4	72388	0.520366	0.998356
Cdkn3	72391	-0.1135	0.998356
Tmem175	72392	-0.11289	0.998356
Faim2	72393	-0.14884	0.998356
Brap	72399	0.351473	0.998356
Pinx1	72400	-0.35269	0.998356
Wdr44	72404	0.099164	0.998356
Sgol1	72415	-0.1468	0.998356
Lrpprc	72416	-0.15774	0.998356
Ttc30b	72421	0.299112	0.998356
2410042D21Rik	72425	-0.16046	0.998356
Dnajc25	72429	0.569442	0.998356
Ceacam18	72431	0.414514	0.998356
Rab38	72433	0.514849	0.998356
Lypd3	72434	-0.70927	0.998356
5930416I19Rik	72440	-0.4248	0.998356
Ccdc71	72454	-0.13372	0.998356
Htatsf1	72459	0.384714	0.998356
Prcp	72461	-0.04246	0.998356
Zfp131	72465	0.05808	0.998356
Ssbp3	72475	0.020609	0.998356
Tmem87b	72477	0.044006	0.998356
Hsdl2	72479	0.03993	0.998356
Acbd6	72482	-0.21527	0.998356
Rnf219	72486	0.015871	0.998356
Ier5l	72500	0.382808	0.998356
Cwf19l1	72502	-0.19046	0.998356
2610507B11Rik	72503	0.124112	0.998356
Taf4b	72504	-0.25092	0.998356
Rps6kb1	72508	-0.17075	0.998356
Tmem173	72512	-0.07223	0.998356
Wdr43	72515	-0.58373	0.998356
2610307P16Rik	72518	-0.19291	0.998356
Tmem55a	72519	0.285877	0.998356
Aldh1b1	72535	-0.42449	0.998356
Pgam5	72542	-0.38397	0.998356
Fam125b	72543	-0.00826	0.998356
Exosc6	72544	0.182483	0.998356
Reep4	72549	-0.04023	0.998356
Hsdl1	72552	-0.01214	0.998356
Utp14a	72554	-0.15408	0.998356
Zfp566	72556	-0.73546	0.998356
Pcbd2	72562	0.112038	0.998356
Uaca	72565	0.36915	0.998356
Bclaf1	72567	-0.39316	0.998356
Lin9	72568	0.437724	0.998356

Bbs5	72569	0.086409	0.998356
Spats2	72572	-0.02856	0.998356
Zufsp	72580	-0.11016	0.998356
Cul4b	72584	-0.0414	0.998356
Pan3	72587	0.101225	0.998356
Ppme1	72590	0.412318	0.998356
Pdia5	72599	0.473299	0.998356
Zfp655	72611	0.068929	0.998356
2700029M09Rik	72612	-0.0396	0.998356
Pih1d2	72614	-0.09702	0.998356
Anks3	72615	0.090504	0.998356
Pdzd11	72621	-0.39201	0.998356
2700086A05Rik	72628	0.173512	0.998356
Mex3a	72640	-0.07691	0.998356
Tmem209	72649	0.137316	0.998356
2810006K23Rik	72650	-0.10678	0.998356
Ccdc12	72654	0.056154	0.998356
Ints8	72656	0.018414	0.998356
2700094K13Rik	72657	0.306805	0.998356
2700097O09Rik	72658	0.31143	0.998356
Dis3	72662	-0.41785	0.998356
Zfp444	72667	0.058342	0.998356
Zfp518a	72672	-0.15199	0.998356
Adipor1	72674	0.292518	0.998356
Dnajc6	72685	-0.19192	0.998356
Hnrpll	72692	0.024948	0.998356
Lime1	72699	0.390083	0.998356
Zfp618	72701	-0.08315	0.998356
C1qtnf6	72709	0.115644	0.998356
Fam98a	72722	-0.44986	0.998356
Zfp74	72723	-0.52681	0.998356
Tbcc	72726	0.073037	0.998356
B3gat3	72727	0.062873	0.998356
Cdc42se2	72729	0.135379	0.998356
Tmx1	72736	-0.15267	0.998356
Zkscan3	72739	-0.16725	0.998356
Tmem161b	72745	1.07071	0.998356
Hdhd3	72748	-0.21209	0.998356
Tonsl	72749	-0.26164	0.998356
Fam117b	72750	-0.02053	0.998356
Arhgef10l	72754	-0.07632	0.998356
Tmem135	72759	0.13856	0.998356
Rint1	72772	-0.01561	0.998356
Neil1	72774	0.462392	0.998356
Fance	72775	0.38503	0.998356
Sass6	72776	-0.19834	0.998356
Dnajc22	72778	0.307419	0.998356

Tmem48	72787	-0.00181	0.998356
Ttc19	72795	0.007852	0.998356
Zfp429	72807	0.736382	0.998356
Scn2b	72821	0.021528	0.998356
Pard3b	72823	0.018534	0.998356
Mon1a	72825	-0.01367	0.998356
Fam76b	72826	-0.05857	0.998356
Ubash3b	72828	0.422428	0.998356
Dhx30	72831	0.055582	0.998356
Pot1b	72836	-0.08421	0.998356
Prdm4	72843	0.39269	0.998356
Kctd17	72844	-0.45818	0.998356
Zdhhc4	72881	0.504741	0.998356
Ccdc94	72886	-0.28713	0.998356
Setd5	72895	0.028791	0.998356
Ndufv2	72900	0.470438	0.998356
Swi5	72931	-0.02995	0.998356
Ddx41	72935	0.010956	0.998356
Hspb11	72938	-0.32482	0.998356
Lrrc47	72946	-0.20981	0.998356
Agxt2l2	72947	0.321518	0.998356
Tppp	72948	-0.17349	0.998356
Ccnt2	72949	-0.10124	0.998356
Top1mt	72960	-0.28155	0.998356
Gcap14	72972	0.338643	0.998356
Prkrir	72981	-0.07731	0.998356
Tmem138	72982	0.334736	0.998356
Appl1	72993	0.242368	0.998356
Insig2	72999	0.298323	0.998356
Kremen2	73016	0.666236	0.998356
2900064A13Rik	73024	-0.1066	0.998356
Glrx5	73046	-0.05588	0.998356
Ppp1r16a	73062	-0.13883	0.998356
Tmem192	73067	-0.1391	0.998356
Fut11	73068	-0.20904	0.998356
Pmpcb	73078	0.240787	0.998356
Slc22a23	73102	0.171957	0.998356
3110009E18Rik	73103	-0.33112	0.998356
3110003A17Rik	73112	0.357341	0.998356
Tgfbrap1	73122	0.125558	0.998356
Golim4	73124	-0.29507	0.998356
Tmed5	73130	-0.24506	0.998356
Slc25a16	73132	0.228536	0.998356
Prrc1	73137	-0.16074	0.998356
Cenpv	73139	0.378561	0.998356
Larp1	73158	0.040286	0.998356
Otud3	73162	-0.00175	0.998356

Tm7sf2	73166	-0.31132	0.998356
Arhgap8	73167	-0.25732	0.998356
Dem1	73172	1.28816	0.998356
Wasl	73178	0.041805	0.998356
Xpot	73192	0.047801	0.998356
3110043O21Rik	73205	0.089429	0.998356
3110082I17Rik	73212	0.074631	0.998356
3110056O03Rik	73218	0.049428	0.998356
Fam118a	73225	0.225954	0.998356
Zfp942	73233	0.017105	0.998356
Atat1	73242	0.687894	0.998356
Rassf6	73246	0.015303	0.998356
1600027N09Rik	73247	-0.18352	0.998356
Setd7	73251	-0.27297	0.998356
1700037C18Rik	73261	-0.1221	0.998356
Gpbp1	73274	-0.24391	0.998356
Ccdc132	73288	0.227894	0.998356
Rhobtb3	73296	-0.44919	0.998356
1700040I03Rik	73327	0.07515	0.998356
1700034P13Rik	73331	-0.80646	0.998356
Itpripl1	73338	-0.5059	0.998356
Phospho2	73373	-0.14016	0.998356
Dcbld2	73379	-0.39755	0.998356
Hbp1	73389	-0.09276	0.998356
1700052N19Rik	73419	-0.16595	0.998356
1700052K11Rik	73431	0.128186	0.998356
Hspa12a	73442	0.701815	0.998356
Wdr13	73447	-0.75218	0.998356
1700066B19Rik	73449	-0.33673	0.998356
Zfp763	73451	-0.41598	0.998356
1700066M21Rik	73467	-0.12486	0.998356
Rnf38	73469	-1.19011	0.998356
Iws1	73473	-0.22267	0.998356
Mipol1	73490	-0.24904	0.998356
1700086O06Rik	73516	0.54018	0.998356
1700096K18Rik	73571	1.18063	0.998356
1700106N22Rik	73582	-0.33435	0.998356
Marveld3	73608	0.270746	0.998356
Ms4a6c	73656	0.406147	0.998356
Spns1	73658	-0.23722	0.998356
Thoc3	73666	0.111531	0.998356
Ttc21b	73668	0.049219	0.998356
Wdr75	73674	0.027231	0.998356
Zbtb8a	73680	-0.0203	0.998356
Trmt11	73681	-0.06952	0.998356
Glipr1	73690	-0.1458	0.998356
2410091C18Rik	73694	-0.26426	0.998356

Ppp2r1b	73699	-0.56908	0.998356
Tubb2b	73710	0.237504	0.998356
Fam125a	73711	-0.10427	0.998356
Dmkn	73712	0.700689	0.998356
Rbm20	73713	0.280743	0.998356
Sh3bgrl3	73723	0.118079	0.998356
Mcee	73724	-0.43581	0.998356
Fcf1	73736	-0.24949	0.998356
1110008P14Rik	73737	-0.11003	0.998356
Haus7	73738	0.184062	0.998356
Cby1	73739	0.179749	0.998356
Man2c1	73744	-0.30671	0.998356
1110034G24Rik	73747	0.096325	0.998356
Thap1	73754	-0.45686	0.998356
Kif2c	73804	0.062602	0.998356
Fam83e	73813	0.102949	0.998356
Mfsd12	73822	-0.03069	0.998356
Snhg6	73824	2.04359	0.998356
Klraq1	73825	0.254797	0.998356
Poldip3	73826	-0.04176	0.998356
Tmem198b	73827	-0.11109	0.998356
Dcaf4	73828	0.524453	0.998356
Eif3k	73830	0.238977	0.998356
Fam98c	73833	0.148791	0.998356
Atp6v1d	73834	-0.19315	0.998356
Slc35b2	73836	-0.02055	0.998356
Ankrd42	73845	0.199339	0.998356
Fam110a	73847	0.12462	0.998356
D3Ertd751e	73852	-0.24228	0.998356
Arhgap18	73910	0.043349	0.998356
Ift57	73916	0.179725	0.998356
Lyrm1	73919	0.345258	0.998356
Otud4	73945	-0.53744	0.998356
4930413G21Rik	73951	0.228896	0.998356
Herc3	73998	0.455913	0.998356
Jakmip3	74004	0.44942	0.998356
Dnm1l	74006	0.033522	0.998356
Rap2b	74012	-0.10425	0.998356
Fcho1	74015	-0.14225	0.998356
Als2	74018	-0.12204	0.998356
Traf3ip1	74019	-0.25014	0.998356
Glyr1	74022	-0.6793	0.998356
Msl1	74026	-0.02355	0.998356
Rin2	74030	0.15051	0.998356
Sdr42e1	74032	0.042929	0.998356
Nol9	74035	-0.14407	0.998356
Pex26	74043	0.132383	0.998356

Ttf2	74044	-0.24714	0.998356
4921525O09Rik	74050	0.367938	0.998356
Steap2	74051	-0.137	0.998356
Plce1	74055	-0.00969	0.998356
4933406C10Rik	74076	0.277372	0.998356
Nmnat3	74080	-0.1451	0.998356
Cep350	74081	0.035489	0.998356
Npl	74091	0.592224	0.998356
Tjap1	74094	-0.16948	0.998356
Hvcn1	74096	-0.23254	0.998356
Pop7	74097	0.471146	0.998356
0610037L13Rik	74098	0.106069	0.998356
Slc35a5	74102	0.211878	0.998356
Nebl	74103	0.015057	0.998356
Abcb6	74104	0.350602	0.998356
Dcaf6	74106	0.237767	0.998356
Cep55	74107	-0.28849	0.998356
Parn	74108	0.225464	0.998356
Rbm19	74111	-0.44035	0.998356
Usp16	74112	0.043273	0.998356
Crot	74114	0.510846	0.998356
Actr3	74117	0.182105	0.998356
Zfp263	74120	0.01757	0.998356
Tmem43	74122	0.260917	0.998356
Foxp4	74123	0.186849	0.998356
Armc8	74125	0.365702	0.998356
Syvn1	74126	-0.36548	0.998356
Rnf6	74132	-0.17145	0.998356
1200011M11Rik	74133	-0.05244	0.998356
Cyp2s1	74134	0.447725	0.998356
Sec14l1	74136	0.164348	0.998356
Nuak2	74137	-0.14429	0.998356
Tm9sf1	74140	0.035528	0.998356
Lonp1	74142	0.178377	0.998356
Opa1	74143	-0.64996	0.998356
F13a1	74145	0.607327	0.998356
Ehhadh	74147	-0.28097	0.998356
1300001I01Rik	74148	-0.01363	0.998356
Zfp946	74149	0.505618	0.998356
Slc35f5	74150	0.324954	0.998356
1300002K09Rik	74152	-0.423	0.998356
Uba7	74153	0.437892	0.998356
Errfi1	74155	0.336848	0.998356
Ftsjd2	74157	0.136778	0.998356
Josd1	74158	0.159264	0.998356
Acbd5	74159	0.553333	0.998356
Nfx1	74164	-0.43607	0.998356

Nudt9	74167	-0.1133	0.998356
Zdhhc16	74168	0.441928	0.998356
1700012B15Rik	74173	0.221286	0.998356
Stk40	74178	0.193931	0.998356
Gbe1	74185	0.09535	0.998356
Ccdc3	74186	0.175361	0.998356
Katnb1	74187	0.069483	0.998356
Arpc5l	74192	-0.25592	0.998356
Rnd3	74194	0.240049	0.998356
Elp3	74195	-0.24015	0.998356
Ttc27	74196	-0.01808	0.998356
Gtf2e1	74197	-0.48243	0.998356
Dtx2	74198	-1.12722	0.998356
2810403A07Rik	74200	0.012497	0.998356
Cep97	74201	2.67489	0.998356
Fblim1	74202	-0.17518	0.998356
Eif4enif1	74203	-0.24471	0.998356
Xpo6	74204	-0.1169	0.998356
Acsl3	74205	0.379553	0.998356
Sipa1l3	74206	-0.0208	0.998356
1700017B05Rik	74211	0.017895	0.998356
Rbm26	74213	-0.19597	0.998356
Paqr8	74229	0.574584	0.998356
Tubgcp2	74237	0.060958	0.998356
Mterfd3	74238	0.359765	0.998356
Iqce	74239	-0.38916	0.998356
Chpf	74241	-0.51028	0.998356
2210009G21Rik	74243	-0.28093	0.998356
Atg7	74244	0.256902	0.998356
Ctbs	74245	0.10284	0.998356
Gale	74246	-0.06202	0.998356
Ankrd9	74251	-0.03805	0.998356
Armc1	74252	-0.07684	0.998356
Gpn1	74254	0.115882	0.998356
Smu1	74255	-0.03749	0.998356
Cyld	74256	-0.26382	0.998356
Aven	74268	0.588012	0.998356
Usp20	74270	0.269633	0.998356
Chic2	74277	0.231592	0.998356
Kcmf1	74287	0.205378	0.998356
Mtmr3	74302	0.26433	0.998356
Rnf145	74315	-0.20898	0.998356
Isca2	74316	0.190049	0.998356
Hopx	74318	0.265157	0.998356
1110005A03Rik	74319	-0.13344	0.998356
Wdr33	74320	-0.05007	0.998356
Cxxc1	74322	-0.06191	0.998356

Cltb	74325	-0.10297	0.998356
Hnrnpr	74326	-0.27519	0.998356
Dnajc14	74330	-0.23804	0.998356
Ranbp10	74334	0.114077	0.998356
Xrcc3	74335	-0.30326	0.998356
Ahcyl2	74340	-0.30029	0.998356
G630025P09Rik	74341	-0.83308	0.998356
Crtc2	74343	0.020997	0.998356
4632415K11Rik	74347	-0.00508	0.998356
Fam160a2	74349	-0.39554	0.998356
Ddx23	74351	-0.42983	0.998356
Zfp84	74352	-0.28067	0.998356
Smchd1	74355	-0.07002	0.998356
4931414P19Rik	74359	-0.14322	0.998356
Cep57	74360	-0.07082	0.998356
Lonrf3	74365	0.127643	0.998356
Rptor	74370	0.08939	0.998356
Clec16a	74374	0.287679	0.998356
Gcc1	74375	-0.21294	0.998356
Ubap2l	74383	-0.99739	0.998356
Mudeng	74385	0.344364	0.998356
Rmi1	74386	0.018444	0.998356
Dpp8	74388	-0.12806	0.998356
Specc1l	74392	0.102018	0.998356
Ppapdc2	74411	0.278888	0.998356
Gle1	74412	0.067105	0.998356
Tc2n	74413	0.325884	0.998356
Polr3c	74414	0.029699	0.998356
Tmc5	74424	-0.29524	0.998356
Eaf1	74427	0.116776	0.998356
4933407C03Rik	74440	0.06429	0.998356
Sgms2	74442	0.005173	0.998356
Pank2	74450	0.125034	0.998356
Pgs1	74451	0.11093	0.998356
Nsun6	74455	0.110986	0.998356
Pus10	74467	0.070264	0.998356
Cep72	74470	0.120081	0.998356
4933439C10Rik	74476	-0.14796	0.998356
4933427D14Rik	74477	-0.20113	0.998356
Snx29	74478	0.40407	0.998356
Snx11	74479	0.911761	0.998356
Batf2	74481	-0.03656	0.998356
Osbpl10	74486	0.175778	0.998356
5430405H02Rik	74487	1.01851	0.998356
Tnks2	74493	0.041378	0.998356
Gorasp1	74498	0.279821	0.998356
Fam53a	74504	0.037975	0.998356

Neto2	74513	0.120477	0.998356
Morc2a	74522	-0.30885	0.998356
8430406I07Rik	74528	-0.67125	0.998356
Gzf1	74533	0.053697	0.998356
Gsdmc4	74548	0.521826	0.998356
Mau2	74549	-1.78641	0.998356
Pck2	74551	-0.04869	0.998356
Nipal3	74552	0.428979	0.998356
9130404H23Rik	74556	-0.05093	0.998356
Elovl7	74559	0.128164	0.998356
Mlkl	74568	0.088418	0.998356
Ttc17	74569	0.296013	0.998356
Zkscan1	74570	1.14672	0.998356
Sppl3	74585	0.288967	0.998356
Cds1	74596	0.537665	0.998356
Mrpl47	74600	0.051586	0.998356
Abcb8	74610	0.133987	0.998356
Scrn3	74616	-0.36557	0.998356
Scpep1	74617	0.003268	0.998356
Fam46c	74645	0.255214	0.998356
Spsb1	74646	-0.16785	0.998356
S100pbp	74648	-0.70458	0.998356
4930444A02Rik	74653	-0.24825	0.998356
Zfp943	74670	-0.00125	0.998356
Wdr35	74682	-0.23094	0.998356
Tbc1d30	74694	-0.20943	0.998356
Snx16	74718	0.522979	0.998356
Trim14	74735	0.375824	0.998356
Pcf11	74737	0.293139	0.998356
5730419I09Rik	74741	1.07849	0.998356
Ddit4	74747	-0.08961	0.998356
5830415F09Rik	74753	-0.34492	0.998356
Dhcr24	74754	-0.03593	0.998356
Mxra8	74761	0.386341	0.998356
Nat15	74763	0.252608	0.998356
Klc4	74764	0.152619	0.998356
Yipf2	74766	-0.17024	0.998356
Pik3cb	74769	0.259218	0.998356
Lmbr1l	74775	0.117857	0.998356
Ppa2	74776	0.491481	0.998356
Sepn1	74777	-0.32305	0.998356
Rrp7a	74778	0.173821	0.998356
Wipi2	74781	0.024146	0.998356
Naa15	74838	-0.07509	0.998356
Manf	74840	-0.1258	0.998356
Usp38	74841	0.091829	0.998356
Tmem65	74868	0.151212	0.998356

4930461G14Rik	74879	0.525085	0.998356
4930455F23Rik	74895	-0.22043	0.998356
4930471M23Rik	74919	0.252117	0.998356
Usp47	74996	0.469719	0.998356
Rab11fip2	74998	-0.33279	0.998356
Fam63a	75007	0.298971	0.998356
Sf3a3	75062	-0.2515	0.998356
Zbtb49	75079	-0.6743	0.998356
Uhrf1bp1l	75089	0.117695	0.998356
Lysmd4	75099	-0.5056	0.998356
4930526I15Rik	75135	-0.38558	0.998356
Rprd2	75137	-0.03489	0.998356
Tmem180	75146	0.029345	0.998356
Rnf121	75212	0.533056	0.998356
4930534B04Rik	75216	0.32018	0.998356
Dusp18	75219	0.325409	0.998356
Dpp3	75221	-0.03522	0.998356
Pelp1	75273	-0.12903	0.998356
Bcdin3d	75284	0.155599	0.998356
Prkd3	75292	-0.31779	0.998356
Fgfr1op	75296	-0.02502	0.998356
Asxl2	75302	-0.0166	0.998356
Taf1d	75316	0.120454	0.998356
4930547N16Rik	75317	0.269463	0.998356
Etnk1	75320	-0.02926	0.998356
Mphosph8	75339	-0.35331	0.998356
Sirt4	75387	-0.00036	0.998356
0610040F04Rik	75394	1.64484	0.998356
Mrpl32	75398	-0.14608	0.998356
Ndufs7	75406	0.238092	0.998356
Wbp7	75410	0.106483	0.998356
Arhgap12	75415	0.548718	0.998356
Nop14	75416	0.042452	0.998356
Secisbp2	75420	-0.1531	0.998356
Mettl5	75422	-0.04828	0.998356
Arl5a	75423	-0.11113	0.998356
Tti1	75425	0.297938	0.998356
3200002M19Rik	75430	0.156194	0.998356
Ascc2	75452	-0.26356	0.998356
Phpt1	75454	0.585098	0.998356
Cklf	75458	-0.23347	0.998356
Oplah	75475	0.238206	0.998356
Ttc32	75516	-0.04211	0.998356
Fpgt	75540	-0.0602	0.998356
1700019G17Rik	75541	-0.18877	0.998356
Akap13	75547	0.208838	0.998356
Zc3h14	75553	1.66587	0.998356

Ep400	75560	0.045233	0.998356
Rsph9	75564	-0.38987	0.998356
Ccdc101	75565	-0.64014	0.998356
Capsl	75568	-0.18943	0.998356
Acyp2	75572	0.884147	0.998356
2310002L13Rik	75577	0.183549	0.998356
Fggy	75578	0.097964	0.998356
2310034G01Rik	75579	0.689177	0.998356
Zbtb4	75580	-0.02224	0.998356
2410003K15Rik	75593	0.297474	0.998356
Ndufaf2	75597	0.447205	0.998356
Pcdh1	75599	0.286554	0.998356
Calml4	75600	-0.01835	0.998356
Kdm5b	75605	-0.09273	0.998356
Wnk2	75607	0.002267	0.998356
Chmp4b	75608	0.15173	0.998356
Gns	75612	0.127481	0.998356
Med25	75613	-0.08152	0.998356
2810008M24Rik	75616	0.011199	0.998356
Rps25	75617	-0.03698	0.998356
Fastkd2	75619	-0.09607	0.998356
2810422J05Rik	75620	-0.2879	0.998356
1700029F09Rik	75623	0.238871	0.998356
Metap1	75624	0.249475	0.998356
Snapc1	75627	-0.41274	0.998356
Rai14	75646	0.285815	0.998356
Lin37	75660	-0.31246	0.998356
Ccdc64	75665	0.662546	0.998356
Pik3r4	75669	0.035149	0.998356
Ippk	75678	0.003973	0.998356
Nudt16	75686	0.074882	0.998356
Fam65a	75687	0.065868	0.998356
Nr2c2ap	75692	-1.45697	0.998356
Fam35a	75698	-0.17329	0.998356
Eif4b	75705	-0.05882	0.998356
Rbm12	75710	-0.21967	0.998356
Tmem14a	75712	-0.24532	0.998356
Cul5	75717	-0.20609	0.998356
Amotl1	75723	-0.04561	0.998356
Phf14	75725	-0.06805	0.998356
5133401N09Rik	75731	-0.24734	0.998356
Mff	75734	-0.10696	0.998356
Pank1	75735	0.108084	0.998356
Bcl2l12	75736	-0.06876	0.998356
Mpp7	75739	-0.00498	0.998356
Svip	75744	-0.21879	0.998356
Morc4	75746	0.379535	0.998356

Sesn3	75747	-0.24316	0.998356
Ipo4	75751	-0.01169	0.998356
9130401M01Rik	75758	0.204539	0.998356
Apol7a	75761	0.918643	0.998356
Dcaf17	75763	0.08397	0.998356
Slx1b	75764	0.71034	0.998356
Rab11fip1	75767	-0.16013	0.998356
Them4	75778	-0.42016	0.998356
Lca5	75782	-0.45286	0.998356
Klhl24	75785	0.323105	0.998356
Ckap5	75786	0.054757	0.998356
Smurf1	75788	0.531841	0.998356
Nln	75805	-0.05504	0.998356
Senp2	75826	0.204544	0.998356
Rnf139	75841	-0.02177	0.998356
Arl5b	75869	-0.12898	0.998356
Zfp821	75871	0.775512	0.998356
4930579K19Rik	75881	0.741301	0.998356
Adal	75894	-0.01487	0.998356
Dcp1a	75901	-0.04597	0.998356
Vmp1	75909	0.038813	0.998356
Exoc6b	75914	0.136337	0.998356
4930579G24Rik	75939	-0.24747	0.998356
Srrm2	75956	-0.13963	0.998356
Mir17hg	75957	0.14684	0.998356
Trappc8	75964	0.144412	0.998356
Zdhhc20	75965	0.219759	0.998356
Dock11	75974	0.267701	0.998356
Slain2	75991	-0.05486	0.998356
Zc3h18	76014	-0.76882	0.998356
Gon4l	76022	0.692756	0.998356
Cant1	76025	-0.43772	0.998356
Ccdc125	76041	-0.11038	0.998356
Ncapg2	76044	-0.02462	0.998356
Ganc	76051	-0.02082	0.998356
Mgea5	76055	0.246544	0.998356
Jakmip1	76071	-0.03061	0.998356
Rnf183	76072	-0.10801	0.998356
Pcgf5	76073	0.122216	0.998356
Ttpal	76080	-2.79564	0.998356
Dock8	76088	0.286248	0.998356
Rapgef2	76089	0.170496	0.998356
Lpo	76113	-0.63137	0.998356
Gpsm2	76123	0.218453	0.998356
Las1l	76130	-0.04561	0.998356
Depdc1a	76131	0.022315	0.998356
Ccdc90a	76137	0.430156	0.998356

Snrnp35	76167	0.15292	0.998356
6330578E17Rik	76178	-0.01524	0.998356
Usp31	76179	-0.33423	0.998356
Med13l	76199	-0.19605	0.998356
Stard3nl	76205	-0.06248	0.998356
Dnttip1	76233	0.274927	0.998356
Grhpr	76238	-0.24064	0.998356
Rtf1	76246	-0.05452	0.998356
0610007P08Rik	76251	0.204791	0.998356
Atp6v0e2	76252	-0.12554	0.998356
Ttc8	76260	0.288113	0.998356
0610040J01Rik	76261	0.051783	0.998356
Gstk1	76263	0.637901	0.998356
Tsen54	76265	-0.19494	0.998356
Fads1	76267	-0.11594	0.998356
Ndfip2	76273	0.626766	0.998356
Tax1bp3	76281	0.162519	0.998356
Atp11b	76295	0.219176	0.998356
Erp44	76299	0.047836	0.998356
Pcnp	76302	-0.05941	0.998356
Osbp	76303	0.041561	0.998356
1110021L09Rik	76306	-0.174	0.998356
Rab1b	76308	0.494654	0.998356
1110019D14Rik	76311	-0.469	0.998356
Cog2	76332	0.006304	0.998356
Tgds	76355	0.586695	0.998356
Trmt5	76357	-0.43919	0.998356
Mtif3	76366	0.718688	0.998356
Det1	76375	-0.06989	0.998356
Ccdc46	76380	0.297083	0.998356
Abcc3	76408	0.485686	0.998356
Fam187b	76415	0.239744	0.998356
Znrd1as	76416	0.150018	0.998356
2310003C23Rik	76425	-0.16102	0.998356
Lhpp	76429	0.066153	0.998356
Ppp1r18	76448	-0.08899	0.998356
Ccdc134	76457	-0.28133	0.998356
Casc5	76464	-0.32288	0.998356
Msrb2	76467	0.539266	0.998356
Haus8	76478	-0.44758	0.998356
Smndc1	76479	-0.03123	0.998356
3110002H16Rik	76482	-0.01706	0.998356
Lmf1	76483	0.049497	0.998356
Glt8d1	76485	0.127973	0.998356
Abhd14b	76491	0.107704	0.998356
Ppp1r11	76497	-0.00702	0.998356
Paqr4	76498	0.189759	0.998356

Clasp2	76499	0.102753	0.998356
Ip6k2	76500	0.346706	0.998356
Commd9	76501	-0.03244	0.998356
1500009C09Rik	76505	-1.41554	0.998356
2210015D19Rik	76508	-0.26662	0.998356
1600029D21Rik	76509	0.153504	0.998356
Trappc9	76510	-0.02826	0.998356
Cln6	76524	-0.02765	0.998356
Il34	76527	0.363816	0.998356
D19Ertd737e	76539	0.209106	0.998356
Tmem101	76547	0.220991	0.998356
Ccdc6	76551	-0.12284	0.998356
Atg2b	76559	0.021794	0.998356
Prss8	76560	0.593542	0.998356
Snx7	76561	0.224807	0.998356
Qrsl1	76563	0.409032	0.998356
Ift46	76568	0.489561	0.998356
Mfsd2a	76574	-0.2639	0.998356
Faf2	76577	0.17668	0.998356
Ipo11	76582	-0.17178	0.998356
Unc5cl	76589	-0.36879	0.998356
Dnajc18	76594	-0.11357	0.998356
Hectd3	76608	0.437935	0.998356
Immt	76614	-0.14779	0.998356
Msi2	76626	-0.36248	0.998356
Stambpl1	76630	-0.39419	0.998356
1700113A16Rik	76642	0.32321	0.998356
Srxn1	76650	0.471529	0.998356
Trim12a	76681	0.346092	0.998356
Spcs3	76687	-0.03246	0.998356
Arfrp1	76688	-0.17011	0.998356
Clasp1	76707	0.381667	0.998356
Arpc2	76709	-0.03274	0.998356
1700081L11Rik	76719	-0.1099	0.998356
Creld2	76737	-0.1829	0.998356
Efr3a	76740	0.130061	0.998356
Snx27	76742	-0.0116	0.998356
Mospd2	76763	0.528811	0.998356
Wdyhv1	76773	-0.09084	0.998356
Slc10a7	76775	-0.09872	0.998356
Cluap1	76779	-0.0184	0.998356
Mettl4	76781	-0.28894	0.998356
Mtif2	76784	-0.04884	0.998356
Klhdc10	76788	-0.05962	0.998356
Mzt1	76789	0.047742	0.998356
2410131K14Rik	76792	0.079044	0.998356
Snip1	76793	0.067802	0.998356

Tbc1d9b	76795	0.035277	0.998356
2510006D16Rik	76799	-0.11104	0.998356
Usp42	76800	0.012778	0.998356
Kdm4c	76804	-0.1928	0.998356
Rpl18a	76808	-0.47343	0.998356
Bri3bp	76809	-0.30643	0.998356
Armc6	76813	-0.07507	0.998356
Sdccag8	76816	-0.3223	0.998356
Fam54b	76824	0.914289	0.998356
Nubpl	76826	-0.55606	0.998356
Hyls1	76832	-0.43185	0.998356
Dtl	76843	0.005675	0.998356
Rps9	76846	-0.18137	0.998356
Spopl	76857	-0.32821	0.998356
Dcun1d5	76863	0.276978	0.998356
Rhbdd1	76867	0.283737	0.998356
Adck4	76889	-0.14681	0.998356
Memo1	76890	0.476185	0.998356
Rnft1	76892	-0.07686	0.998356
Lass2	76893	0.040402	0.998356
Mettl15	76894	-0.16456	0.998356
Bicd2	76895	-0.44245	0.998356
Golga1	76899	-0.07577	0.998356
Ssbp4	76900	0.199832	0.998356
Lrg1	76905	0.027964	0.998356
Mnd1	76915	-0.16603	0.998356
4930455C21Rik	76916	-0.01893	0.998356
Flywch2	76917	0.25084	0.998356
Arfip2	76932	-0.28513	0.998356
Ifi27l2a	76933	0.812521	0.998356
Hnrnpm	76936	-0.51964	0.998356
Rbm17	76938	0.062818	0.998356
Psapl1	76943	0.089933	0.998356
2310030N02Rik	76947	0.256022	0.998356
Nt5c2	76952	-0.42771	0.998356
St5	76954	0.533082	0.998356
Chmp5	76959	-0.12648	0.998356
Bcas1	76960	0.094751	0.998356
2700049A03Rik	76967	0.311761	0.998356
2810008D09Rik	76972	-0.24385	0.998356
Scfd1	76983	0.095309	0.998356
Hdhd2	76987	-0.53224	0.998356
Ddrgk1	77006	0.053868	0.998356
2700099C18Rik	77022	0.34339	0.998356
Slc9a8	77031	0.092787	0.998356
2610029I01Rik	77032	0.083126	0.998356
2510039O18Rik	77034	0.491592	0.998356

Jmjd5	77035	-0.04983	0.998356
Arfgap2	77038	-1.04214	0.998356
Atg16l1	77040	-0.12332	0.998356
Arsk	77041	0.292666	0.998356
Arid2	77044	0.021981	0.998356
Bcl7a	77045	-0.38604	0.998356
Ccdc41	77048	0.105227	0.998356
Sun1	77053	0.172718	0.998356
Tmco4	77056	0.153641	0.998356
Ints7	77065	-0.07277	0.998356
Ankrd11	77087	0.062812	0.998356
Ocel1	77090	-0.19589	0.998356
Tanc2	77097	-0.00354	0.998356
Tmem181a	77106	0.403405	0.998356
Gpbp1l1	77110	0.158177	0.998356
Klhl2	77113	0.26243	0.998356
Mtmr2	77116	0.237931	0.998356
9130221H12Rik	77124	-0.44727	0.998356
Il33	77125	-0.10922	0.998356
A930001N09Rik	77128	0.198146	0.998356
Hnrnpa0	77134	0.247844	0.998356
Ptgr2	77219	-0.75402	0.998356
9430038I01Rik	77252	0.569284	0.998356
Yif1b	77254	-0.75938	0.998356
Zfp142	77264	-0.19096	0.998356
Nkrf	77286	-0.23415	0.998356
Raph1	77300	0.18001	0.998356
Wdr82	77305	-0.11391	0.998356
Sec24a	77371	-0.19263	0.998356
Rab35	77407	0.191283	0.998356
Esrp2	77411	-0.2445	0.998356
C330018D20Rik	77422	-0.5952	0.998356
Heg1	77446	-0.09157	0.998356
Kidins220	77480	0.212874	0.998356
Zfp266	77519	-0.10303	0.998356
Jrkl	77532	-0.07299	0.998356
C030034I22Rik	77533	-0.31178	0.998356
Shisa4	77552	-0.13942	0.998356
Agl	77559	0.090217	0.998356
Limch1	77569	0.401809	0.998356
Vps33a	77573	0.125736	0.998356
Fam115a	77574	0.138228	0.998356
Bcl9	77578	0.068741	0.998356
Myh10	77579	0.670267	0.998356
Mboat7	77582	-0.24025	0.998356
Notum	77583	0.454554	0.998356
Chst15	77590	0.218154	0.998356

Ddx10	77591	-0.05159	0.998356
Usp45	77593	0.031015	0.998356
C430048L16Rik	77604	-0.18955	0.998356
H2afv	77605	0.153437	0.998356
Prelid2	77619	0.624677	0.998356
Apex2	77622	-0.0516	0.998356
Smpd4	77626	-0.34325	0.998356
4930594C11Rik	77633	0.039181	0.998356
Snapc3	77634	-0.1406	0.998356
C330007P06Rik	77644	0.21384	0.998356
Zfp955a	77652	-0.12713	0.998356
Arhgef38	77669	-0.37825	0.998356
5033406O09Rik	77675	0.094335	0.998356
Mmab	77697	-0.41595	0.998356
Mrps5	77721	-0.4101	0.998356
Rnf170	77733	0.160699	0.998356
6720463M24Rik	77744	-0.2562	0.998356
Epm2aip1	77781	-0.11817	0.998356
Polq	77782	-0.44608	0.998356
Esco1	77805	0.150016	0.998356
Lrrc42	77809	0.123563	0.998356
A930015D03Rik	77810	0.444955	0.998356
Tchp	77832	-0.20052	0.998356
Msl2	77853	-0.06226	0.998356
Thyn1	77862	-0.33843	0.998356
Ypel2	77864	-0.15829	0.998356
6030458C11Rik	77877	-0.47896	0.998356
Lbh	77889	0.003528	0.998356
Ube2s	77891	0.207739	0.998356
Yipf6	77929	-0.00372	0.998356
Fam53b	77938	-0.17503	0.998356
Cyp20a1	77951	0.450323	0.998356
Hook1	77963	-0.00366	0.998356
Tmem50b	77975	0.159187	0.998356
Nuak1	77976	0.208235	0.998356
Sbf1	77980	0.186663	0.998356
Ascc3	77987	-0.04791	0.998356
D730039F16Rik	77996	0.599795	0.998356
Prr15	78004	-0.18342	0.998356
Mccc2	78038	0.176621	0.998356
Sowahb	78088	0.354575	0.998356
8430410K20Rik	78100	-0.17195	0.998356
4930414L22Rik	78108	0.262167	0.998356
Trappc6b	78232	0.061325	0.998356
Dnajc21	78244	-0.07912	0.998356
Phf23	78246	-0.07254	0.998356
Fam55b	78252	-0.43706	0.998356

Ralgps2	78255	0.210764	0.998356
Zfp687	78266	0.16956	0.998356
Creb3l4	78284	-0.19828	0.998356
Nav2	78286	0.13247	0.998356
Zfyve20	78287	-0.13612	0.998356
Lsmd1	78304	0.056758	0.998356
Gpr108	78308	0.006708	0.998356
Ccdc88b	78317	-0.33795	0.998356
2310046O06Rik	78323	0.21369	0.998356
Ndufv3	78330	0.414543	0.998356
Cdk19	78334	0.179881	0.998356
Ttyh3	78339	-0.08995	0.998356
Snrnp25	78372	0.609318	0.998356
Mvp	78388	0.273763	0.998356
Ddx52	78394	0.074349	0.998356
Wibg	78428	-9.62164	0.998356
2700046A07Rik	78449	0.133252	0.998356
Helz	78455	-0.04756	0.998356
B230219D22Rik	78521	0.191585	0.998356
Mrpl9	78523	0.087639	0.998356
Asb8	78541	0.040976	0.998356
Utp23	78581	-0.0331	0.998356
Srbd1	78586	-0.30472	0.998356
6430706D22Rik	78605	-0.0611	0.998356
Uvrag	78610	0.183909	0.998356
Btbd19	78611	-0.74496	0.998356
Acap2	78618	0.318996	0.998356
Lsm6	78651	-0.62982	0.998356
Bola3	78653	-0.65713	0.998356
Ncapd3	78658	-0.19301	0.998356
Plekhj1	78670	-0.41088	0.998356
Naa35	78689	0.121754	0.998356
Pus7	78697	-0.17478	0.998356
Troap	78733	-0.25232	0.998356
Rassf10	78748	0.225352	0.998356
Filip1l	78749	-0.14677	0.998356
Csgalnact2	78752	0.205632	0.998356
Rictor	78757	0.064156	0.998356
2410002I01Rik	78777	0.13247	0.998356
Spata2l	78779	0.100528	0.998356
Zc3hav1	78781	-0.03549	0.998356
Brpf1	78783	0.030199	0.998356
Usp54	78787	0.103138	0.998356
Armc9	78795	-0.26375	0.998356
Zcchc4	78796	-0.05352	0.998356
Ndor1	78797	-0.08474	0.998356
Eml4	78798	0.0138	0.998356

Stxbp5	78808	-0.01627	0.998356
Gmip	78816	0.539509	0.998356
Pppde1	78825	0.533713	0.998356
Tsc22d4	78829	-0.24867	0.998356
Slc25a12	78830	0.057816	0.998356
2700078E11Rik	78832	0.097827	0.998356
Gins3	78833	-0.00711	0.998356
Zfp623	78834	-0.11852	0.998356
B430010I23Rik	78849	0.125116	0.998356
Coro7	78885	-0.00526	0.998356
Sfi1	78887	0.5286	0.998356
Wsb1	78889	-0.58966	0.998356
2310079F23Rik	78890	-0.09993	0.998356
Scyl1	78891	-0.22701	0.998356
Cnot10	78893	0.1176	0.998356
Aacs	78894	0.021013	0.998356
Wrnip1	78903	0.195445	0.998356
9130017N09Rik	78906	0.099545	0.998356
Igsf3	78908	-0.14597	0.998356
Sp2	78912	0.284815	0.998356
Ltn1	78913	0.178498	0.998356
Nadsyn1	78914	0.072327	0.998356
Dlst	78920	0.050311	0.998356
9130019O22Rik	78921	0.245824	0.998356
Gas2l1	78926	0.176449	0.998356
Pigt	78928	-0.02554	0.998356
Polr3h	78929	-0.23166	0.998356
Saal1	78935	-0.05188	0.998356
Avl9	78937	0.068677	0.998356
Fbxo34	78938	0.917186	0.998356
Ern1	78943	-0.18365	0.998356
Spsb3	79043	0.036713	0.998356
Mrps34	79044	-0.0505	0.998356
Nme3	79059	0.497349	0.998356
Osbpl5	79196	0.031256	0.998356
Tnfrsf23	79201	0.797015	0.998356
Zfp319	79233	0.010917	0.998356
Trim39	79263	-0.07684	0.998356
Krit1	79264	-0.33546	0.998356
Lias	79464	0.50899	0.998356
Gltpd1	79554	-0.04373	0.998356
BC005537	79555	0.121611	0.998356
Ublcp1	79560	0.242919	0.998356
Wbscr27	79565	0.220016	0.998356
Sh3bp5l	79566	-5.47831	0.998356
Cdk5rap3	80280	-0.17655	0.998356
Cttnbp2nl	80281	-0.25025	0.998356

Abtb1	80283	0.287559	0.998356
BC003266	80284	0.371745	0.998356
Parp9	80285	0.273649	0.998356
Tusc3	80286	-0.09083	0.998356
Apobec3	80287	-0.1727	0.998356
Bcl9l	80288	-0.06527	0.998356
Lysmd3	80289	0.096991	0.998356
Rilpl2	80291	-0.53366	0.998356
Zxdc	80292	0.202864	0.998356
Pofut2	80294	0.153248	0.998356
Tusc2	80385	0.458	0.998356
Med8	80509	0.267989	0.998356
Herpud2	80517	0.347585	0.998356
Wwox	80707	0.064629	0.998356
Pacsin3	80708	0.225914	0.998356
Rab27b	80718	-0.03843	0.998356
Igsf6	80719	-0.19495	0.998356
Mynn	80732	0.195117	0.998356
Vps16	80743	-0.28823	0.998356
Cwc22	80744	0.642857	0.998356
BC004004	80748	0.137958	0.998356
N4bp1	80750	-0.08298	0.998356
Rnf34	80751	0.330369	0.998356
Fam20c	80752	-0.81154	0.998356
Cblc	80794	2.41714	0.998356
Selk	80795	-0.06554	0.998356
Rhoj	80837	-0.1543	0.998356
Hist1h1a	80838	0.078828	0.998356
Nfkbiz	80859	-0.23876	0.998356
Ghdc	80860	0.563123	0.998356
Dhx58	80861	0.579803	0.998356
Ifitm2	80876	-0.38096	0.998356
Lrba	80877	0.512554	0.998356
Slc16a3	80879	0.001115	0.998356
Maged2	80884	-0.31721	0.998356
Niacr1	80885	-0.78763	0.998356
Senp3	80886	0.106966	0.998356
Hspb8	80888	0.03062	0.998356
Mesdc1	80889	0.328411	0.998356
Trim2	80890	0.118335	0.998356
Erap1	80898	-0.03173	0.998356
Cxcr6	80901	-0.37948	0.998356
Dtx3	80904	-0.18965	0.998356
Polh	80905	-0.20876	0.998356
Lactb	80907	0.316654	0.998356
Acox3	80911	0.262518	0.998356
Pum1	80912	-0.46299	0.998356

Pum2	80913	0.364073	0.998356
Uck2	80914	-0.2032	0.998356
Dusp12	80915	0.038072	0.998356
Syt13	80976	0.59405	0.998356
9930013L23Rik	80982	-0.40929	0.998356
Trim44	80985	0.073519	0.998356
Ckap2	80986	0.065619	0.998356
Nckipsd	80987	-0.2067	0.998356
Rad54l2	81000	-0.2581	0.998356
Trim23	81003	0.591028	0.998356
Tbl1xr1	81004	0.034006	0.998356
Rnf114	81018	-0.08162	0.998356
Dnajb1	81489	0.245704	0.998356
Btnl5	81497	0.665219	0.998356
Sil1	81500	0.161014	0.998356
Sgpp1	81535	-0.04547	0.998356
Kat5	81601	-0.07879	0.998356
Zbtb22	81630	0.049407	0.998356
Ankrd17	81702	0.518296	0.998356
Sorcs2	81840	0.117201	0.998356
Gpank1	81845	-0.15951	0.998356
Tfcp2l1	81879	-0.40841	0.998356
Ift122	81896	0.462924	0.998356
Sf3b1	81898	-0.00891	0.998356
Zfpl1	81909	-0.34705	0.998356
Rrbp1	81910	0.131291	0.998356
Tfap4	83383	-0.31485	0.998356
Glis2	83396	0.561667	0.998356
Akap12	83397	-0.48883	0.998356
Lamtor2	83409	-0.02557	0.998356
Cstf2t	83410	-0.14183	0.998356
Ctns	83429	0.178947	0.998356
Ndel1	83431	0.006707	0.998356
Trem2	83433	-0.33109	0.998356
Plekha3	83435	-0.16463	0.998356
Plekha2	83436	-0.05244	0.998356
Ngrn	83485	0.119619	0.998356
Rbm5	83486	-0.21408	0.998356
Pik3ap1	83490	-0.34325	0.998356
Sacm1l	83493	0.198825	0.998356
Fstl3	83554	0.229674	0.998356
Gtf2a1	83602	0.175125	0.998356
Wdr6	83669	-0.0049	0.998356
Sytl2	83671	0.301141	0.998356
Snhg1	83673	0.953449	0.998356
Pde4dip	83679	-0.32808	0.998356
Srrt	83701	-0.444	0.998356

Dbr1	83703	0.374566	0.998356
Slc12a9	83704	0.376464	0.998356
Dpp7	83768	0.106199	0.998356
Smarcd2	83796	-0.78771	0.998356
Smarcd1	83797	0.143243	0.998356
Tnk1	83813	0.32552	0.998356
Nedd4l	83814	0.212462	0.998356
Cenpq	83815	0.342524	0.998356
Tmem2	83921	0.214802	0.998356
Dnaja3	83945	-0.20937	0.998356
Phip	83946	-0.10367	0.998356
Btbd1	83962	0.50465	0.998356
Enpp5	83965	-10	0.998356
Slmap	83997	0.169825	0.998356
Mcam	84004	0.429785	0.998356
Kremen1	84035	0.287047	0.998356
Usp8	84092	-0.15655	0.998356
Plvap	84094	0.429078	0.998356
Pi4k2a	84095	-0.10002	0.998356
Ptov1	84113	0.055217	0.998356
Setdb1	84505	-0.40157	0.998356
Rnf123	84585	0.180284	0.998356
Snurf	84704	-0.04207	0.998356
Rpph1	85029	-0.15974	0.998356
Pla1a	85031	1.07853	0.998356
Kars	85305	-0.57481	0.998356
Fam158a	85308	0.880925	0.998356
Sec16b	89867	-0.29681	0.998356
Trim8	93679	-0.26072	0.998356
Zfp192	93681	-0.35196	0.998356
Glce	93683	0.159445	0.998356
Entpd7	93685	-0.02909	0.998356
Rbfox2	93686	-0.61541	0.998356
Csnk1a1	93687	0.063309	0.998356
Klf7	93691	0.053352	0.998356
Glrx	93692	0.135957	0.998356
Clec2d	93694	-0.03781	0.998356
Chrac1	93696	-0.0307	0.998356
Narg2	93697	-0.03287	0.998356
Pcdhgb6	93703	-0.21911	0.998356
Pcdhgc3	93706	-0.26271	0.998356
Cpn1	93721	-0.52063	0.998356
Lztfl1	93730	0.269838	0.998356
Wnt16	93735	0.906008	0.998356
Aff4	93736	0.184267	0.998356
Pard6g	93737	-0.25218	0.998356
Gabarapl2	93739	0.247849	0.998356

Pard3	93742	-0.24259	0.998356
Echs1	93747	0.028207	0.998356
Immp2l	93757	-0.80426	0.998356
Sirt1	93759	1.19523	0.998356
Arid1a	93760	-0.01424	0.998356
Smarca5	93762	-0.04794	0.998356
Ube2n	93765	-0.19935	0.998356
Nipa2	93790	-1.01839	0.998356
Rnf111	93836	-0.10562	0.998356
Dach2	93837	-1.35004	0.998356
Dqx1	93838	0.17045	0.998356
Vangl2	93840	0.078576	0.998356
Uchl4	93841	-0.19741	0.998356
Igsf9	93842	0.1447	0.998356
Brwd1	93871	0.330444	0.998356
Pcdhb7	93878	-1.20142	0.998356
Pcdhb9	93880	0.190657	0.998356
Pcdhb16	93887	-0.21077	0.998356
Pcdhb17	93888	-0.18079	0.998356
Fzd10	93897	0.609966	0.998356
Nkd1	93960	-0.4499	0.998356
B3galt5	93961	0.299848	0.998356
Clmn	94040	0.106494	0.998356
Bcl2l13	94044	-0.07706	0.998356
Mrpl1	94061	-0.44283	0.998356
Mrpl3	94062	-0.17365	0.998356
Mrpl16	94063	-0.05434	0.998356
Mrpl27	94064	-0.21149	0.998356
Mrpl36	94066	-0.11414	0.998356
Mrpl43	94067	0.106654	0.998356
Trim6	94088	0.69624	0.998356
Trim11	94091	-0.28606	0.998356
Trim16	94092	0.304331	0.998356
Trim33	94093	0.442459	0.998356
Trim34a	94094	0.511202	0.998356
Med15	94112	0.549106	0.998356
Mcoln1	94178	-0.27103	0.998356
Krt23	94179	-0.05151	0.998356
Nans	94181	0.001862	0.998356
Pdxdc1	94184	0.239558	0.998356
Tnfrsf21	94185	0.126669	0.998356
Strn3	94186	-0.06184	0.998356
Ophn1	94190	-0.05266	0.998356
C1galt1	94192	0.133447	0.998356
Pag1	94212	-0.53394	0.998356
Ddx50	94213	0.073488	0.998356
Spock2	94214	0.57423	0.998356

Cnnm3	94218	-0.08938	0.998356
Cnnm4	94220	0.549682	0.998356
Gopc	94221	-0.15717	0.998356
Dgcr8	94223	0.188863	0.998356
Srd5a2	94224	1.13588	0.998356
Cpsf1	94230	-0.11729	0.998356
Ubqln4	94232	-0.41996	0.998356
Tinagl1	94242	0.380408	0.998356
Dtnbp1	94245	-0.12064	0.998356
Arid4b	94246	-0.39855	0.998356
Wbscr16	94254	0.206257	0.998356
Maged1	94275	-0.51207	0.998356
Sfxn3	94280	-0.04767	0.998356
Prcc	94315	-0.29671	0.998356
Loxl2	94352	0.174601	0.998356
Tmem62	96957	0.874527	0.998356
Ptges2	96979	0.271895	0.998356
Tprn	97031	0.071049	0.998356
Wwtr1	97064	0.135416	0.998356
Nmd3	97112	0.360772	0.998356
Hist2h4	97122	0.116252	0.998356
C77080	97130	0.249285	0.998356
A430005L14Rik	97159	1.52988	0.998356
Hmgb2	97165	-0.62492	0.998356
Hadha	97212	0.456648	0.998356
Mtmr14	97287	0.253021	0.998356
Strn4	97387	-0.01605	0.998356
Cog8	97484	-0.22652	0.998356
Cmtm4	97487	0.094995	0.998356
Qars	97541	-0.0659	0.998356
Sgsm2	97761	0.033483	0.998356
D930048N14Rik	97775	0.320951	0.998356
4833439L19Rik	97820	-0.05562	0.998356
Exd2	97827	0.205323	0.998356
C78339	97863	0.176692	0.998356
B3galnt2	97884	-0.30103	0.998356
Hist1h3g	97908	-0.37902	0.998356
Nol12	97961	-0.54934	0.998356
Deptor	97998	-0.40012	0.998356
Gtf2f1	98053	-0.16999	0.998356
Tmem132a	98170	-0.16342	0.998356
Dcaf8	98193	-0.12612	0.998356
Eif3m	98221	-0.04736	0.998356
Lrrc59	98238	-0.32852	0.998356
Stk17b	98267	0.191836	0.998356
D2hgdh	98314	-0.20351	0.998356
Slamf9	98365	0.41456	0.998356

Smap1	98366	0.4207	0.998356
Gorab	98376	0.086657	0.998356
Lbr	98386	-0.16199	0.998356
Slc41a1	98396	-0.04487	0.998356
Sh3bp4	98402	-0.11946	0.998356
Zfp451	98403	-0.04063	0.998356
AI597479	98404	-0.01401	0.998356
Nucks1	98415	-0.30503	0.998356
Cnih4	98417	-0.31207	0.998356
Phlpp1	98432	0.132752	0.998356
Gtf3c3	98488	0.282164	0.998356
Pid1	98496	0.351288	0.998356
Mfsd6	98682	0.239009	0.998356
1190005F20Rik	98685	-0.2284	0.998356
Rabif	98710	0.099473	0.998356
Rdh10	98711	0.324498	0.998356
Rab3gap2	98732	0.047447	0.998356
Hnrnpf	98758	-0.1457	0.998356
Ubac1	98766	-0.40239	0.998356
Cdc123	98828	0.286323	0.998356
Eps8l2	98845	0.043571	0.998356
Ehd4	98878	0.20246	0.998356
Usp6nl	98910	-0.62994	0.998356
Fam102a	98952	0.098072	0.998356
Nat10	98956	-0.17696	0.998356
Clp1	98985	-0.0081	0.998356
Znfx1	98999	0.205139	0.998356
Qser1	99003	-0.13433	0.998356
Lpcat4	99010	0.155215	0.998356
Pomt1	99011	0.12266	0.998356
Mrps26	99045	-0.01913	0.998356
Cep152	99100	-0.08635	0.998356
Stard7	99138	-0.01176	0.998356
Anapc2	99152	-0.03625	0.998356
Ssx2ip	99167	-0.06724	0.998356
Tm9sf4	99237	-0.03252	0.998356
Commd7	99311	-0.19879	0.998356
Zscan29	99334	0.019839	0.998356
Dnajc24	99349	0.469483	0.998356
Arfgef2	99371	-0.0748	0.998356
Cul4a	99375	-0.05627	0.998356
Abtb2	99382	0.476418	0.998356
Golga2	99412	0.200056	0.998356
Magi3	99470	0.559476	0.998356
Dnttip2	99480	-0.0544	0.998356
Wdr47	99512	0.088536	0.998356
Usp53	99526	0.353325	0.998356

Olfml3	99543	0.6068	0.998356
Lphn2	99633	-0.13203	0.998356
4933434E20Rik	99650	-0.10627	0.998356
Eps8l3	99662	0.017195	0.998356
Sec24b	99683	-0.02723	0.998356
Ankrd50	99696	0.16327	0.998356
Cept1	99712	0.434819	0.998356
Taf13	99730	-0.03722	0.998356
Tmem56	99887	-5.1E-05	0.998356
Arfip1	99889	0.366503	0.998356
Prmt6	99890	-0.01696	0.998356
Tiparp	99929	0.016968	0.998356
Kdm1a	99982	-0.04621	0.998356
Ldlrap1	100017	0.014453	0.998356
Mdn1	100019	-0.3308	0.998356
Lrrc19	100061	0.478592	0.998356
Camta1	100072	-0.07632	0.998356
Kti12	100087	-0.44902	0.998356
Rcc1	100088	-2.06022	0.998356
Pcsk9	100102	-0.15078	0.998356
Tdrd7	100121	0.624257	0.998356
Pafah2	100163	-0.08726	0.998356
Phactr4	100169	-0.17228	0.998356
Zmym6	100177	-0.21011	0.998356
Akna	100182	-0.12013	0.998356
H6pd	100198	0.119847	0.998356
Tmem64	100201	-0.13954	0.998356
Adprhl2	100206	0.530479	0.998356
Gpn2	100210	0.47073	0.998356
Rusc2	100213	0.662492	0.998356
Stx12	100226	0.087482	0.998356
Osbpl9	100273	0.696437	0.998356
AU040320	100317	-0.44849	0.998356
Ppp1r8	100336	0.254465	0.998356
Smpdl3b	100340	-0.48566	0.998356
Fam46b	100342	0.67457	0.998356
Bsdc1	100383	0.023401	0.998356
Slc44a1	100434	0.825146	0.998356
Mob3c	100465	-0.04396	0.998356
Zfand2a	100494	1.92017	0.998356
Zfp518b	100515	-0.08625	0.998356
Rell1	100532	0.024863	0.998356
Slc15a4	100561	0.198457	0.998356
Lrrc8c	100604	-0.0567	0.998356
Noc4l	100608	-0.16426	0.998356
Nsun5	100609	-0.26976	0.998356
D930016D06Rik	100662	0.070255	0.998356

Psph	100678	0.162698	0.998356
Trrap	100683	-0.05562	0.998356
Spon2	100689	0.249689	0.998356
Gbp6	100702	-0.44147	0.998356
Pds5b	100710	-0.01297	0.998356
Papd4	100715	0.257494	0.998356
Ugt2b34	100727	0.128611	0.998356
Dcun1d4	100737	-0.39035	0.998356
Usp30	100756	0.105246	0.998356
Ube3c	100763	0.078747	0.998356
1110008J03Rik	100764	0.587506	0.998356
Tbc1d14	100855	-0.04584	0.998356
Hscb	100900	0.357668	0.998356
Chpf2	100910	-0.03246	0.998356
Tyw1	100929	-0.24157	0.998356
Emilin1	100952	0.588553	0.998356
Rab28	100972	0.051081	0.998356
Nfxl1	100978	0.193085	0.998356
Akap9	100986	0.123543	0.998356
Zfp282	101095	-0.29271	0.998356
Tmem168	101118	0.346602	0.998356
Rpusd3	101122	-0.10096	0.998356
Itfg2	101142	-0.16363	0.998356
B630005N14Rik	101148	0.477955	0.998356
Pot1a	101185	0.116483	0.998356
Parp11	101187	-0.00119	0.998356
Zfp956	101197	0.288174	0.998356
Hepacam2	101202	-0.39271	0.998356
Tada3	101206	-0.05159	0.998356
Tra2a	101214	-0.10013	0.998356
Wdr91	101240	0.04543	0.998356
6720456B07Rik	101314	0.113977	0.998356
A130022J15Rik	101351	0.128754	0.998356
Fbxl14	101358	0.070764	0.998356
Adamts9	101401	-0.42821	0.998356
Dhx32	101437	0.245369	0.998356
Phrf1	101471	-0.1697	0.998356
Plekha1	101476	0.129785	0.998356
Ric8	101489	0.406454	0.998356
Inpp5f	101490	0.414192	0.998356
Plekhg2	101497	1.45112	0.998356
Hsd3b7	101502	-1.66392	0.998356
Mob2	101513	0.296484	0.998356
Prkd2	101540	1.57341	0.998356
Wtip	101543	0.373979	0.998356
6330503K22Rik	101565	-0.24392	0.998356
Vrk3	101568	-0.15904	0.998356

Eftud1	101592	0.723606	0.998356
E430018J23Rik	101604	0.120842	0.998356
Grwd1	101612	-0.38389	0.998356
Pwwp2b	101631	-0.16608	0.998356
Spty2d1	101685	-0.17735	0.998356
Trim68	101700	-0.02378	0.998356
Numa1	101706	-0.09452	0.998356
Psip1	101739	-0.51138	0.998356
Ano1	101772	0.43563	0.998356
C230052I12Rik	101831	-0.70899	0.998356
AW146154	101835	0.042143	0.998356
Ints4	101861	0.018886	0.998356
Rrp8	101867	-0.64553	0.998356
Unc45a	101869	-0.09281	0.998356
Sf3b3	101943	-0.18115	0.998356
D8Ertd738e	101966	-0.16759	0.998356
AA960436	101985	0.121057	0.998356
Zfp828	101994	-0.28279	0.998356
AI316807	102032	1.33491	0.998356
Exoc8	102058	0.099024	0.998356
Gadd45gip1	102060	-0.06987	0.998356
Phkb	102093	0.317117	0.998356
Arhgef18	102098	-0.0556	0.998356
Mtus1	102103	0.010568	0.998356
Dohh	102115	-0.23257	0.998356
Fam192a	102122	0.321484	0.998356
E130303B06Rik	102124	-0.46839	0.998356
Snx25	102141	0.49462	0.998356
Taf5l	102162	0.508705	0.998356
Prmt10	102182	0.318411	0.998356
Zdhhc7	102193	0.229632	0.998356
Snapc2	102209	0.267173	0.998356
Agpat6	102247	-0.18093	0.998356
Cyp4v3	102294	0.360119	0.998356
Dcun1d2	102323	-0.09264	0.998356
Ankrd10	102334	-0.27908	0.998356
Cog4	102339	0.208067	0.998356
Myzap	102371	0.1341	0.998356
Clk3	102414	-0.21842	0.998356
Hinfp	102423	-0.10368	0.998356
Lars2	102436	-0.02285	0.998356
Dennd4a	102442	-0.28654	0.998356
Imp3	102462	-0.31116	0.998356
Pls1	102502	0.460744	0.998356
Cmtm7	102545	-1.06825	0.998356
Ano10	102566	0.272981	0.998356
Alg9	102580	0.010608	0.998356

Plekho2	102595	0.14044	0.998356
Snx19	102607	0.022749	0.998356
Rpp25	102614	-0.51088	0.998356
Mapkapk3	102626	0.347114	0.998356
Acad11	102632	0.319517	0.998356
Oaf	102644	0.080739	0.998356
Cd276	102657	-0.07218	0.998356
Phldb1	102693	-0.2598	0.998356
Bbs4	102774	-0.10742	0.998356
Tcta	102791	0.118072	0.998356
Slc6a8	102857	0.549526	0.998356
Pls3	102866	-0.14766	0.998356
Cenpi	102920	-0.0409	0.998356
6720401G13Rik	103012	0.15771	0.998356
Pan2	103135	0.256259	0.998356
Pwp1	103136	-0.12853	0.998356
Gstt3	103140	-0.14563	0.998356
Rdh9	103142	-0.16054	0.998356
Upb1	103149	0.575789	0.998356
Chchd10	103172	-0.10636	0.998356
Fig4	103199	0.080144	0.998356
Traf3ip2	103213	-0.25837	0.998356
Csnk1g2	103236	0.18937	0.998356
AI597468	103266	-0.3639	0.998356
Zc3h10	103284	0.060113	0.998356
Ncln	103425	-0.05772	0.998356
Nup107	103468	0.122755	0.998356
Mbtd1	103537	0.03337	0.998356
Psme4	103554	-0.04221	0.998356
Xpo1	103573	0.634262	0.998356
Fbxw11	103583	0.219378	0.998356
Smg6	103677	-0.00415	0.998356
Tmed4	103694	0.14922	0.998356
Slc35e4	103710	0.342355	0.998356
Pnpo	103711	-0.14276	0.998356
Tbc1d10a	103724	-0.09157	0.998356
Tubg1	103733	-0.29195	0.998356
Pex12	103737	-0.70714	0.998356
1810046J19Rik	103742	-0.08965	0.998356
Tmem98	103743	0.021741	0.998356
Wdr92	103784	-0.005	0.998356
Maml1	103806	0.109537	0.998356
Zfp692	103836	-0.4184	0.998356
Nt5m	103850	0.538408	0.998356
Rpn1	103963	-0.05141	0.998356
Qsox1	104009	0.018517	0.998356
Synj1	104015	0.00267	0.998356

Synpo	104027	-0.03812	0.998356
Wdr7	104082	-0.09796	0.998356
Cyp27a1	104086	-0.2665	0.998356
Airn	104103	-0.23049	0.998356
Acly	104112	-0.52181	0.998356
Ndufb11	104130	0.564396	0.998356
Etv5	104156	0.092456	0.998356
Sbk1	104175	-0.11499	0.998356
Blmh	104184	-0.17981	0.998356
Rhoq	104215	0.193447	0.998356
Cabin1	104248	-0.06133	0.998356
Cdc42ep2	104252	0.065366	0.998356
Kdm3a	104263	-0.11908	0.998356
Tex15	104271	0.130063	0.998356
Arl1	104303	-0.09349	0.998356
Csnk1d	104318	0.134459	0.998356
Gas8	104346	-0.02347	0.998356
Zfp120	104348	-0.56462	0.998356
Zfp119a	104349	-0.0271	0.998356
Rcor2	104383	-0.22846	0.998356
E2f4	104394	-0.29625	0.998356
Pcnxl3	104401	0.101287	0.998356
Bap1	104416	-0.02097	0.998356
Dgkz	104418	0.088224	0.998356
Rexo2	104444	-0.20445	0.998356
Cdc42ep1	104445	-0.49437	0.998356
0610010K14Rik	104457	0.419365	0.998356
Rars	104458	-0.00736	0.998356
Ccdc117	104479	-0.25758	0.998356
Smek2	104570	-0.05549	0.998356
Cnot6	104625	-0.2021	0.998356
Tsr1	104662	-0.3954	0.998356
Slc16a6	104681	-0.14884	0.998356
Ddx1	104721	0.32241	0.998356
1110002B05Rik	104725	-0.21916	0.998356
4930427A07Rik	104732	-0.33149	0.998356
Pld4	104759	-0.26293	0.998356
Jkamp	104771	-0.5457	0.998356
Aldh6a1	104776	0.293034	0.998356
Vipar	104799	0.125814	0.998356
Ptpn23	104831	0.019805	0.998356
Cbll1	104836	-0.39154	0.998356
Tecpr2	104859	-0.08193	0.998356
Spata7	104871	0.376307	0.998356
Tdp1	104884	-0.41487	0.998356
Rab15	104886	-0.30087	0.998356
Adi1	104923	-0.03613	0.998356

Fam110c	104943	0.101602	0.998356
Fam84a	105005	0.028818	0.998356
Rdh14	105014	0.304398	0.998356
Pelo	105083	-0.3814	0.998356
Iars	105148	-0.24668	0.998356
Arrdc3	105171	0.041181	0.998356
Rnf44	105239	-0.1567	0.998356
Slc9a3	105243	-0.0137	0.998356
Txndc5	105245	-0.012	0.998356
Brd9	105246	-0.1074	0.998356
Golm1	105348	0.40394	0.998356
AW209491	105351	-0.1396	0.998356
Utp15	105372	-0.11373	0.998356
Ankrd32	105377	0.022208	0.998356
Akr1c14	105387	-0.46036	0.998356
Fam149b	105428	-0.38107	0.998356
Slain1	105439	0.410332	0.998356
Dock9	105445	0.209361	0.998356
Gmpr2	105446	0.124508	0.998356
Abhd4	105501	0.458131	0.998356
Exoc5	105504	0.264974	0.998356
Chmp7	105513	0.101553	0.998356
Ankrd28	105522	0.227914	0.998356
Mbnl2	105559	-0.39334	0.998356
Dph3	105638	0.152229	0.998356
Thtpa	105663	0.065384	0.998356
Rcbtb2	105670	0.462049	0.998356
Ppif	105675	-0.20293	0.998356
Mycbp2	105689	0.070889	0.998356
Ano6	105722	-0.57631	0.998356
Slc38a1	105727	-0.23101	0.998356
Fam83h	105732	0.435138	0.998356
Scrib	105782	-0.02819	0.998356
Kdelr3	105785	-0.70138	0.998356
Prkaa1	105787	-0.12224	0.998356
Sgsm3	105835	0.134983	0.998356
Mtbp	105837	0.024885	0.998356
Dennd3	105841	-0.10911	0.998356
Card10	105844	-0.28419	0.998356
Lmf2	105847	-0.1293	0.998356
Mal2	105853	-0.01706	0.998356
9030619P08Rik	105892	0.201124	0.998356
Espl1	105988	-0.18938	0.998356
Fam19a5	106014	0.988341	0.998356
Topors	106021	0.083292	0.998356
Sharpin	106025	0.2892	0.998356
Gga1	106039	-0.23152	0.998356

Fbxo4	106052	0.510467	0.998356
AW549877	106064	-0.14434	0.998356
Slc45a4	106068	0.245836	0.998356
Mfsd5	106073	-0.25179	0.998356
Cggbp1	106143	0.099131	0.998356
Txndc11	106200	-0.17534	0.998356
Zc3h7a	106205	0.1008	0.998356
Qtrtd1	106248	0.036521	0.998356
0610012G03Rik	106264	0.244774	0.998356
Rrn3	106298	0.15618	0.998356
Osbpl11	106326	-0.04511	0.998356
Nsun3	106338	-0.08473	0.998356
Rfc4	106344	-0.01168	0.998356
Ildr1	106347	0.233925	0.998356
Ypel1	106369	0.385331	0.998356
Stk38	106504	-0.04468	0.998356
Gpsm3	106512	0.172729	0.998356
Tecr	106529	0.612958	0.998356
Ppcs	106564	0.02286	0.998356
Rab31	106572	0.456953	0.998356
Itfg3	106581	0.214569	0.998356
Nrm	106582	-0.40495	0.998356
Scaf8	106583	0.064462	0.998356
Ankrd12	106585	0.19312	0.998356
Trip10	106628	-0.66126	0.998356
Ift140	106633	0.011974	0.998356
Vmac	106639	0.661844	0.998356
AI413582	106672	0.029259	0.998356
Ticam1	106759	0.005785	0.998356
Stap2	106766	0.229073	0.998356
Dhx57	106794	0.212942	0.998356
Tcf19	106795	-0.11265	0.998356
AI314976	106821	0.302954	0.998356
Unc119b	106840	0.132516	0.998356
Tnfaip8	106869	0.184042	0.998356
Afap1l1	106877	-0.04751	0.998356
2010002N04Rik	106878	-0.04533	0.998356
Hmgxb3	106894	-0.47701	0.998356
Slc39a3	106947	-0.0623	0.998356
Slc39a6	106957	-0.23724	0.998356
Gramd3	107022	-0.35514	0.998356
Me2	107029	0.196764	0.998356
Fbxo38	107035	0.06712	0.998356
Lars	107045	0.275544	0.998356
Psmg2	107047	-0.02961	0.998356
Wdr74	107071	-0.2342	0.998356
Rrp12	107094	-0.24279	0.998356

Gpr137	107173	0.982086	0.998356
Btaf1	107182	-0.16925	0.998356
AI462493	107197	-0.07009	0.998356
O3far1	107221	-0.42897	0.998356
AI837181	107242	0.010486	0.998356
Otub1	107260	0.456765	0.998356
Yars	107271	-0.24789	0.998356
Psat1	107272	-0.30581	0.998356
Vps37c	107305	-0.14093	0.998356
Gbf1	107338	0.148852	0.998356
AW112010	107350	0.317732	0.998356
Kank1	107351	-0.14491	0.998356
Tm9sf3	107358	0.31212	0.998356
Pdzd8	107368	0.029328	0.998356
Exoc6	107371	-0.02755	0.998356
Fam111a	107373	-0.54486	0.998356
Slc25a45	107375	-0.24068	0.998356
Brms1	107392	-0.07873	0.998356
Hat1	107435	0.245751	0.998356
Acaca	107476	-0.08626	0.998356
Eprs	107508	-0.1849	0.998356
Ssr1	107513	-0.01659	0.998356
Lgr4	107515	0.412746	0.998356
Ece2	107522	-0.20505	0.998356
Gimap4	107526	-0.14772	0.998356
Il1rl2	107527	0.188844	0.998356
Arl2bp	107566	0.131998	0.998356
Wwp1	107568	0.157711	0.998356
Nt5c3	107569	-0.13544	0.998356
Col16a1	107581	0.092361	0.998356
Ovol2	107586	-0.09376	0.998356
Mylk	107589	-0.29902	0.998356
Rdh1	107605	0.408953	0.998356
Pi4kb	107650	0.131701	0.998356
Uap1	107652	-0.16311	0.998356
Snrpd2	107686	-0.08517	0.998356
Sf3b4	107701	-0.20108	0.998356
Rnh1	107702	0.826794	0.998356
Slc12a6	107723	0.347912	0.998356
Mrpl10	107732	-0.17168	0.998356
Mrpl41	107733	-0.28532	0.998356
Mrpl30	107734	0.406361	0.998356
Rapgef1	107746	0.104435	0.998356
Aldh1l1	107747	0.064668	0.998356
Lgals2	107753	0.398893	0.998356
Scamp1	107767	0.157552	0.998356
Tm6sf2	107770	0.276341	0.998356

Bmyc	107771	-0.42419	0.998356
Jmjd6	107817	-0.32566	0.998356
Whsc1	107823	0.072952	0.998356
Thoc5	107829	-0.04238	0.998356
Cth	107869	-0.18173	0.998356
Mthfs	107885	0.80086	0.998356
Mgat5	107895	0.046915	0.998356
Chd4	107932	-0.04336	0.998356
Pom121	107939	-0.08429	0.998356
Cdk9	107951	0.210059	0.998356
Pacs1	107975	-0.33308	0.998356
Bre	107976	-1.65076	0.998356
Ddb2	107986	0.606488	0.998356
Cdc20	107995	0.018719	0.998356
Gtpbp6	107999	0.475193	0.998356
Cenpf	108000	-0.10475	0.998356
Ap4e1	108011	-0.15124	0.998356
Ap1s2	108012	0.050319	0.998356
Srsf9	108014	0.121013	0.998356
Fxyd4	108017	0.146189	0.998356
Shmt2	108037	-0.75571	0.998356
Camk2d	108058	0.177071	0.998356
Cstf2	108062	-0.38564	0.998356
Eif2b3	108067	-0.24127	0.998356
Ltbp4	108075	-0.48843	0.998356
Skiv2l	108077	0.062019	0.998356
Olr1	108078	0.747867	0.998356
Prkaa2	108079	0.584391	0.998356
Pip4k2b	108083	-0.353	0.998356
Rnf216	108086	0.147731	0.998356
Prkab2	108097	0.216587	0.998356
Med21	108098	0.167404	0.998356
Prkag2	108099	0.362751	0.998356
Baiap2	108100	0.039071	0.998356
Fermt3	108101	0.278996	0.998356
B3gnt5	108105	0.485966	0.998356
Eif4ebp3	108112	-0.13956	0.998356
Slco4a1	108115	-0.87134	0.998356
U2af1	108121	-0.46246	0.998356
Napg	108123	0.07754	0.998356
Napa	108124	-0.09899	0.998356
Xrcc4	108138	-0.02222	0.998356
Taf9	108143	-0.58869	0.998356
Atic	108147	-0.27527	0.998356
Galnt7	108150	0.10816	0.998356
Ogt	108155	0.023023	0.998356
Mthfd1	108156	-0.19099	0.998356

Ubxn8	108159	-0.06576	0.998356
Fam50a	108160	-0.06463	0.998356
Mat2b	108645	-0.59762	0.998356
Slc35b3	108652	0.613797	0.998356
4933403F05Rik	108654	-0.17881	0.998356
Foxp1	108655	0.176253	0.998356
Rnpepl1	108657	0.25072	0.998356
Rnf187	108660	-0.03744	0.998356
Atp6v1h	108664	0.113343	0.998356
Epsti1	108670	0.598616	0.998356
Dnajc9	108671	-0.05375	0.998356
Zdhhc15	108672	-0.16543	0.998356
Ccdc86	108673	-0.28054	0.998356
Cops8	108679	0.119112	0.998356
Gpt2	108682	-0.10445	0.998356
Edem2	108687	-0.14582	0.998356
Obfc1	108689	-0.1255	0.998356
Pttg1ip	108705	0.179996	0.998356
1810008A18Rik	108707	-0.01191	0.998356
Sft2d2	108735	0.086474	0.998356
Oxsr1	108737	0.310039	0.998356
Lyrm2	108755	0.158478	0.998356
Pnrc1	108767	0.163205	0.998356
Mex3b	108797	0.007407	0.998356
Ston2	108800	0.026478	0.998356
Ccdc122	108811	0.338792	0.998356
Jmjd1c	108829	0.030039	0.998356
Ibtk	108837	0.083109	0.998356
Rdh13	108841	0.177624	0.998356
Mtrf1l	108853	0.009023	0.998356
Ankhd1	108857	-0.03132	0.998356
Atad3a	108888	-0.07635	0.998356
Aif1l	108897	-0.08597	0.998356
2700081O15Rik	108899	0.202055	0.998356
Tbcd	108903	0.339187	0.998356
Nusap1	108907	-0.32924	0.998356
Aida	108909	-0.0476	0.998356
Rcc2	108911	-0.19861	0.998356
Cdca2	108912	-0.36231	0.998356
Rnf169	108937	-0.03083	0.998356
Rg9mtd2	108943	-0.18213	0.998356
Zzz3	108946	0.305777	0.998356
Ppp1r15b	108954	0.011904	0.998356
Fam73b	108958	0.580891	0.998356
Irak2	108960	0.026541	0.998356
E2f8	108961	-0.3245	0.998356
Ciapin1	109006	-0.00478	0.998356

Obfc2a	109019	-0.02435	0.998356
Sp110	109032	0.585155	0.998356
Prkcdbp	109042	0.720141	0.998356
Pfdn4	109054	0.027848	0.998356
1110034A24Rik	109065	-0.23638	0.998356
Exosc4	109075	-0.02121	0.998356
Ints5	109077	-0.10022	0.998356
Sephs1	109079	-0.11007	0.998356
Fbxw17	109082	0.278021	0.998356
Rars2	109093	0.091823	0.998356
Rbm15b	109095	0.032962	0.998356
Slc30a9	109108	0.302246	0.998356
Uhrf2	109113	-0.12571	0.998356
Mmadhc	109129	0.02803	0.998356
Plekha5	109135	-0.09789	0.998356
Mmaa	109136	0.222249	0.998356
Gins4	109145	0.35037	0.998356
Chd9	109151	0.09199	0.998356
Mlec	109154	-0.23201	0.998356
Ube2q2	109161	-0.32465	0.998356
Atl3	109168	0.088788	0.998356
Trip11	109181	-0.09377	0.998356
Fam64a	109212	-0.25688	0.998356
Tmem139	109218	0.230928	0.998356
Ms4a7	109225	-0.27689	0.998356
Fam118b	109229	-0.11961	0.998356
Sccpdh	109232	0.360564	0.998356
Mbd5	109241	0.100561	0.998356
Tspan9	109246	-0.26438	0.998356
9530008L14Rik	109254	-0.24878	0.998356
Rlf	109263	0.118076	0.998356
Prr5	109270	-0.00573	0.998356
C030046I01Rik	109284	0.032068	0.998356
Prex2	109294	0.378404	0.998356
C330006A16Rik	109299	-0.33715	0.998356
Orai1	109305	0.281128	0.998356
Rnf20	109331	0.01621	0.998356
Cdcp1	109332	0.048519	0.998356
Pkn2	109333	0.066105	0.998356
Fam175b	109359	0.028109	0.998356
D730005E14Rik	109361	-0.27748	0.998356
Sri	109552	0.376634	0.998356
Dsp	109620	0.272511	0.998356
Cald1	109624	-0.24305	0.998356
Npy	109648	0.828587	0.998356
Acy1	109652	-0.46506	0.998356
Txlna	109658	-0.21121	0.998356

Cyb5	109672	0.057496	0.998356
Ampd2	109674	-0.26294	0.998356
Arrb1	109689	0.666103	0.998356
Itga1	109700	0.309545	0.998356
Actn1	109711	-0.04321	0.998356
Cyb5r3	109754	0.142314	0.998356
Blvra	109778	0.327259	0.998356
Pgm3	109785	0.014873	0.998356
Clps	109791	-0.00313	0.998356
Glo1	109801	-0.35928	0.998356
H47	109815	0.17235	0.998356
Braf	109880	-0.17632	0.998356
Asl	109900	0.171937	0.998356
Cela1	109901	0.175879	0.998356
Rap1a	109905	0.23504	0.998356
Zfp91	109910	0.045849	0.998356
Zbtb25	109929	0.202507	0.998356
Abr	109934	3.62675	0.998356
Gusb	110006	-0.10412	0.998356
Gm16517	110012	0.250203	0.998356
Kif22	110033	-0.09072	0.998356
Dek	110052	-0.17166	0.998356
Dut	110074	-0.36378	0.998356
Pygb	110078	-0.22882	0.998356
Phka2	110094	0.386573	0.998356
Pygl	110095	-0.42285	0.998356
Mpi	110119	0.387076	0.998356
Ehmt2	110147	0.649882	0.998356
Raf1	110157	0.0492	0.998356
Slc35b1	110172	-0.04155	0.998356
Manba	110173	0.060294	0.998356
Ggct	110175	0.157333	0.998356
Fdps	110196	-0.04196	0.998356
Akr7a5	110198	-0.35771	0.998356
Pgd	110208	0.451128	0.998356
Tmbim6	110213	2.77513	0.998356
Triobp	110253	-0.22231	0.998356
Hba-a2	110257	-9.07292	0.998356
Msra	110265	-0.21644	0.998356
Bcr	110279	0.16668	0.998356
Krt7	110310	-0.23292	0.998356
Cox6b1	110323	0.062187	0.998356
Rap1gap	110351	-0.14592	0.998356
Adrbk1	110355	-0.12616	0.998356
Sec13	110379	0.185053	0.998356
Shroom2	110380	0.265768	0.998356
Qdpr	110391	-0.55581	0.998356

Pigh	110417	0.093149	0.998356
Acat1	110446	0.24737	0.998356
Ly6a	110454	0.238686	0.998356
Acat2	110460	-0.29943	0.998356
Hivep1	110521	0.07164	0.998356
Dgkq	110524	0.392177	0.998356
H2-Q6	110557	-0.29224	0.998356
H2-Q9	110558	-0.64725	0.998356
Prdm2	110593	-0.26244	0.998356
Rgnef	110596	0.216582	0.998356
Fntb	110606	0.105328	0.998356
Hdlbp	110611	-0.11999	0.998356
Atxn3	110616	0.140258	0.998356
Prps2	110639	-0.15605	0.998356
Rps6ka3	110651	0.287906	0.998356
Aldh7a1	110695	-0.07735	0.998356
Chaf1b	110749	0.111347	0.998356
Cse1l	110750	-0.14629	0.998356
Nr3c2	110784	0.378955	0.998356
Tshz1	110796	0.425843	0.998356
Srsf1	110809	-0.24334	0.998356
Pwp2	110816	-0.28763	0.998356
Pcca	110821	0.00867	0.998356
Etfb	110826	0.158762	0.998356
Lims1	110829	-0.21979	0.998356
Etfa	110842	-0.13063	0.998356
Ppp2r4	110854	-0.25956	0.998356
Slc9a4	110895	0.308693	0.998356
Cds2	110911	0.172696	0.998356
Hspa13	110920	-0.00785	0.998356
Hlcs	110948	0.157199	0.998356
Rpl10	110954	-0.2144	0.998356
Nudt19	110959	0.124814	0.998356
Tars	110960	0.105629	0.998356
Mbd6	110962	0.093952	0.998356
Erc1	111173	-0.12728	0.998356
Pecr	111175	-0.11139	0.998356
Hmga1-rs1	111241	0.410981	0.998356
Dom3z	112403	-0.82842	0.998356
Egln1	112405	0.392308	0.998356
Egln2	112406	0.212853	0.998356
Egln3	112407	0.123678	0.998356
2010002M12Rik	112419	0.817769	0.998356
Acaa1a	113868	-0.25027	0.998356
Laptm4b	114128	0.347354	0.998356
Atp6v0b	114143	0.589063	0.998356
Npnt	114249	-0.4603	0.998356

Dok4	114255	0.275404	0.998356
Palmd	114301	-0.38291	0.998356
Slc28a3	114304	-0.08724	0.998356
Lyve1	114332	0.90148	0.998356
Zfp295	114565	-0.3113	0.998356
Clic1	114584	0.048373	0.998356
D17H6S53E	114585	0.055725	0.998356
Ehbp1l1	114601	1.18772	0.998356
Prdm15	114604	-0.07594	0.998356
Rpl31	114641	-1.5395	0.998356
Ly6g6d	114654	1.11052	0.998356
Impa2	114663	-0.27771	0.998356
Hsd17b11	114664	0.259365	0.998356
Selm	114679	-0.47354	0.998356
Rasa2	114713	0.206692	0.998356
Spred1	114715	-0.00952	0.998356
Spred2	114716	-0.30197	0.998356
Supt16h	114741	-0.10716	0.998356
Pawr	114774	0.314562	0.998356
Prosc	114863	-0.23592	0.998356
Ddhd1	114874	-0.17985	0.998356
Cygb	114886	0.664728	0.998356
Dcun1d1	114893	-0.0559	0.998356
Afg3l1	114896	0.063016	0.998356
Vps4a	116733	0.26713	0.998356
Lsm10	116748	3.13127	0.998356
Prelp	116847	-0.0299	0.998356
Baz2a	116848	0.074095	0.998356
Mta1	116870	-0.04625	0.998356
Mta3	116871	2.09592	0.998356
Stim2	116873	0.172946	0.998356
Derl2	116891	-0.14641	0.998356
Dph1	116905	0.238759	0.998356
Slc19a2	116914	-0.07689	0.998356
Tgs1	116940	-0.41596	0.998356
Pop5	117109	0.427856	0.998356
Ube3b	117146	0.043029	0.998356
Tirap	117149	0.91132	0.998356
Pip4k2c	117150	0.113784	0.998356
Ttyh2	117160	0.043135	0.998356
Steap4	117167	0.03057	0.998356
1110038F14Rik	117171	0.136399	0.998356
Cno	117197	0.309722	0.998356
Ivns1abp	117198	0.052448	0.998356
Asb7	117589	0.129065	0.998356
B3galt6	117592	-0.1505	0.998356
Helb	117599	0.059254	0.998356

Srgap1	117600	0.968703	0.998356
Klf16	118445	-0.27258	0.998356
Mrps2	118451	3.02748	0.998356
Mmp28	118453	1.84081	0.998356
Mrps6	121022	-0.02292	0.998356
Muc4	140474	0.734064	0.998356
Man2a2	140481	-0.00628	0.998356
Zfp358	140482	-0.22736	0.998356
Pofut1	140484	-0.00114	0.998356
Acap3	140500	-0.01906	0.998356
Eri3	140546	0.52107	0.998356
Igsf8	140559	0.122956	0.998356
Plxnb2	140570	0.335787	0.998356
Elmo2	140579	0.067578	0.998356
Elmo1	140580	-0.04832	0.998356
Ube4a	140630	-0.10447	0.998356
Caskin2	140721	-0.22622	0.998356
Sec63	140740	0.025984	0.998356
Rem2	140743	-0.53589	0.998356
Bmp2k	140780	-0.07979	0.998356
Wdr5	140858	-0.03415	0.998356
Nek8	140859	0.538934	0.998356
Lnx2	140887	-0.03974	0.998356
Dclre1b	140917	0.272212	0.998356
Zcchc14	142682	-0.19824	0.998356
Asb13	142688	-0.57258	0.998356
Tlr3	142980	0.114194	0.998356
Elovl6	170439	-0.06109	0.998356
Stard4	170459	-0.31258	0.998356
Stard5	170460	-0.08319	0.998356
Recql5	170472	-0.25152	0.998356
Snx18	170625	-0.03847	0.998356
Ubn1	170644	0.070992	0.998356
BC002163	170658	-0.0693	0.998356
Usp48	170707	0.056986	0.998356
Cyp4f13	170716	-0.00264	0.998356
Idh3b	170718	0.118219	0.998356
Oxr1	170719	-0.33567	0.998356
Card14	170720	0.585909	0.998356
Rtn4ip1	170728	-0.36291	0.998356
Mfn2	170731	0.196948	0.998356
Znrf1	170737	0.061293	0.998356
Pilrb1	170741	-0.45776	0.998356
Sertad3	170742	0.476625	0.998356
Mtmr4	170749	-0.11495	0.998356
Xpnpep1	170750	-0.15387	0.998356
Bco2	170752	0.682662	0.998356

Zfp704	170753	-0.09789	0.998356
Slc24a6	170756	0.718221	0.998356
Rac3	170758	0.660563	0.998356
Atp13a1	170759	0.379742	0.998356
Acbd3	170760	0.139815	0.998356
Nup155	170762	0.042045	0.998356
Zfp87	170763	0.142217	0.998356
Rfxap	170767	-0.07618	0.998356
Pfkfb3	170768	1.95685	0.998356
Bbc3	170770	0.430066	0.998356
Glcci1	170772	-0.29466	0.998356
Hdac10	170787	-0.02637	0.998356
Acot8	170789	0.501302	0.998356
Rbm39	170791	0.039409	0.998356
Usp33	170822	-0.04488	0.998356
Glmn	170823	0.124753	0.998356
Ppargc1b	170826	-0.25306	0.998356
Hook2	170833	-1.37256	0.998356
Inpp5j	170835	-0.5214	0.998356
Sumo2	170930	-0.09124	0.998356
Zfp369	170936	-0.01678	0.998356
Zfp617	170938	0.589132	0.998356
Il17rc	171095	-0.19621	0.998356
Mbnl3	171170	-0.08275	0.998356
Syt12	171180	0.055594	0.998356
Galnt10	171212	0.030408	0.998356
Havcr2	171285	0.129105	0.998356
Slc12a8	171286	-1.03243	0.998356
Il17rd	171463	-0.05463	0.998356
Cd99l2	171486	0.169384	0.998356
Apobr	171504	-0.419	0.998356
Creld1	171508	0.240451	0.998356
Mlph	171531	0.501934	0.998356
Bmf	171543	0.025392	0.998356
Nme7	171567	-0.28365	0.998356
Mical1	171580	0.884583	0.998356
Helq	191578	-0.13298	0.998356
Dicer1	192119	0.071833	0.998356
Bspry	192120	0.277852	0.998356
Mvd	192156	0.010841	0.998356
Socs7	192157	0.050213	0.998356
Prpf8	192159	0.025043	0.998356
Casc3	192160	-0.09623	0.998356
Ufsp2	192169	-0.26739	0.998356
Eif4a3	192170	0.041542	0.998356
Fam195b	192173	0.242533	0.998356
Rwdd4a	192174	-0.00273	0.998356

Flna	192176	-0.04031	0.998356
Nadk	192185	-0.01609	0.998356
Stab1	192187	0.205071	0.998356
Med9	192191	-0.22183	0.998356
Shkbp1	192192	-0.07784	0.998356
Edem1	192193	0.055958	0.998356
Ash1l	192195	-0.07629	0.998356
Luc7l2	192196	-0.0789	0.998356
Bcas3	192197	0.203876	0.998356
Lrrc4	192198	0.554394	0.998356
Hexim1	192231	-0.14189	0.998356
Hps4	192232	0.356534	0.998356
Hps1	192236	-0.15926	0.998356
Phf21a	192285	0.321466	0.998356
Slc25a36	192287	-0.27766	0.998356
Nrbp1	192292	0.160375	0.998356
Wdr81	192652	0.204801	0.998356
Ripk2	192656	0.122109	0.998356
Ell2	192657	0.071751	0.998356
Arhgdia	192662	0.225953	0.998356
Rassf3	192678	0.22251	0.998356
Rapgef6	192786	0.05844	0.998356
Itgb4	192897	0.13316	0.998356
Dhrs11	192970	-0.13267	0.998356
Cyb5d2	192986	-0.27825	0.998356
Gm53	193022	0.080993	0.998356
Zfp3	193043	-0.63245	0.998356
Slu7	193116	-0.06358	0.998356
Zfp184	193452	0.164829	0.998356
Rnf185	193670	-0.31559	0.998356
Abhd16a	193742	0.033504	0.998356
Kdm4b	193796	0.083356	0.998356
Mcfd2	193813	0.355957	0.998356
Eme2	193838	-0.07111	0.998356
Mtmr11	194126	0.321635	0.998356
Cnksr1	194231	0.42374	0.998356
9930104L06Rik	194268	-0.10817	0.998356
Tet3	194388	0.018717	0.998356
Mical3	194401	0.066643	0.998356
Reps2	194590	0.371957	0.998356
Klf11	194655	-0.10339	0.998356
Jmjd4	194952	0.718439	0.998356
Zzef1	195018	0.401319	0.998356
Tmem199	195040	-0.004	0.998356
Trim40	195359	0.65761	0.998356
Utp14b	195434	0.143274	0.998356
Zfp691	195522	0.264218	0.998356

Bptf	207165	0.019076	0.998356
Rbms3	207181	0.511094	0.998356
Arhgef17	207212	-0.14631	0.998356
Larp4	207214	-0.21922	0.998356
Zbtb7c	207259	0.149463	0.998356
Fchsd2	207278	-0.34631	0.998356
Hectd1	207304	0.092069	0.998356
Sec23ip	207352	0.048459	0.998356
Fam120c	207375	-0.00433	0.998356
Wdr11	207425	-0.04848	0.998356
Baiap2l2	207495	-0.27141	0.998356
Dtx4	207521	0.069515	0.998356
Tbc1d16	207592	-0.30252	0.998356
Wdr37	207615	0.022577	0.998356
Igsf11	207683	0.104392	0.998356
Gtpbp10	207704	0.303579	0.998356
Pde2a	207728	-1.11738	0.998356
Fam100a	207740	-0.21609	0.998356
Rnf43	207742	-0.09276	0.998356
Csrnp2	207785	-0.0136	0.998356
Gramd1c	207798	-0.5477	0.998356
Smagp	207818	0.180712	0.998356
Galnt6	207839	0.365679	0.998356
Esrp1	207920	0.186019	0.998356
Urb1	207932	-0.49375	0.998356
Alg11	207958	0.629321	0.998356
Mettl21d	207965	0.351822	0.998356
Pif1	208084	-0.28432	0.998356
Chmp6	208092	0.311789	0.998356
Mlxip	208104	0.007084	0.998356
Dhx37	208144	-0.2722	0.998356
Yeats2	208146	-1.10103	0.998356
Phldb2	208177	-0.158	0.998356
Btbd2	208198	-0.16746	0.998356
Alg1	208211	0.389421	0.998356
Mob3a	208228	0.044727	0.998356
Tor1aip1	208263	0.082016	0.998356
Dot1l	208266	-0.12219	0.998356
Zfp871	208292	-0.13641	0.998356
Dip2c	208440	0.209292	0.998356
Sgms1	208449	0.27738	0.998356
1810043H04Rik	208501	-0.02812	0.998356
Cep78	208518	0.179213	0.998356
Rsrc2	208606	-0.24392	0.998356
Etl4	208618	0.155694	0.998356
Alg3	208624	-0.64236	0.998356
Kntc1	208628	-0.08412	0.998356

Slc25a38	208638	0.056127	0.998356
Eif4g1	208643	-0.0963	0.998356
Creb3l2	208647	-0.19246	0.998356
Cblb	208650	-0.30249	0.998356
Fam20a	208659	0.196939	0.998356
Hmgcs1	208715	-0.20823	0.998356
Dis3l2	208718	-0.30817	0.998356
Hdac4	208727	0.008265	0.998356
Tmem63a	208795	-0.18581	0.998356
Daam1	208846	0.200479	0.998356
Zdhhc9	208884	0.27174	0.998356
Myo5c	208943	-0.04678	0.998356
Thnsl1	208967	-0.1431	0.998356
Zfp280c	208968	-0.16967	0.998356
Npb	208990	0.214749	0.998356
Fam83b	208994	0.006089	0.998356
Rbmx2	209003	-0.32514	0.998356
Sirt7	209011	0.451893	0.998356
Vps8	209018	-0.07403	0.998356
Pycr1	209027	0.608513	0.998356
Samd9l	209086	0.283134	0.998356
Clic6	209195	0.198524	0.998356
Dtx3l	209200	0.126375	0.998356
Osgin2	209212	-0.49927	0.998356
Enox2	209224	0.277837	0.998356
Zfp710	209225	0.301215	0.998356
Gan	209239	-0.17246	0.998356
Gps1	209318	0.113461	0.998356
Gen1	209334	-0.39106	0.998356
Eif2b1	209354	0.17078	0.998356
Gtf2h3	209357	-0.26757	0.998356
Taf3	209361	-0.08512	0.998356
Itih5	209378	0.309499	0.998356
Trim30d	209387	0.349095	0.998356
Gpkow	209416	-0.02651	0.998356
Tfe3	209446	-0.21875	0.998356
Trp53bp2	209456	-0.10977	0.998356
Hace1	209462	-0.06759	0.998356
Tbc1d12	209478	0.253825	0.998356
Tmem164	209497	-0.35586	0.998356
Nudcd3	209586	0.050466	0.998356
4922501L14Rik	209601	-0.06591	0.998356
Lcorl	209707	0.093636	0.998356
Kif15	209737	-0.33286	0.998356
Tmc7	209760	0.320037	0.998356
Dennd2a	209773	0.696401	0.998356
Tbc1d25	209815	0.427619	0.998356

B3gntl1	210004	-0.1139	0.998356
Mtrr	210009	-0.05809	0.998356
Metrnl	210029	0.264301	0.998356
Tmem194	210035	-0.38513	0.998356
Zfp719	210105	-0.43529	0.998356
Papd7	210106	0.546177	0.998356
Lpp	210126	-0.23306	0.998356
Zfp180	210135	0.024757	0.998356
Irgq	210146	0.034716	0.998356
Slc30a6	210148	0.435241	0.998356
Zfp526	210172	-0.05391	0.998356
Shank2	210274	-0.75471	0.998356
Mtmr9	210376	-8.3E-05	0.998356
Mettl14	210529	-0.17116	0.998356
Wdr67	210544	-0.25152	0.998356
Mcmbp	210711	0.18516	0.998356
Mkx	210719	-0.54771	0.998356
Brcc3	210766	0.052797	0.998356
9030625A04Rik	210808	-0.24867	0.998356
Ints9	210925	0.330897	0.998356
Kbtbd2	210973	0.00829	0.998356
D15Ertd621e	210998	0.223786	0.998356
Sepsecs	211006	0.068059	0.998356
Trim41	211007	0.291256	0.998356
Alkbh1	211064	0.120072	0.998356
D130040H23Rik	211135	-0.05839	0.998356
Churc1	211151	0.095531	0.998356
Lrrc25	211228	-0.6818	0.998356
Mtrf1	211253	0.058782	0.998356
Kbtbd7	211255	0.137602	0.998356
Cln5	211286	-0.09104	0.998356
Nrg1	211323	0.012986	0.998356
Pank3	211347	-0.0483	0.998356
Mtss1	211401	-0.12384	0.998356
Exoc3	211446	0.101232	0.998356
Ado	211488	-0.03741	0.998356
Tmem87a	211499	-1.24678	0.998356
Nomo1	211548	-0.23013	0.998356
Tifa	211550	0.073818	0.998356
Ap1ar	211556	-0.0631	0.998356
Tfdp2	211586	-0.01362	0.998356
Wwc1	211652	0.142631	0.998356
Cspp1	211660	0.043113	0.998356
Mgst2	211666	0.536315	0.998356
Arfgef1	211673	0.164751	0.998356
Trib1	211770	-0.19325	0.998356
Mfsd9	211798	0.235559	0.998356

Asap2	211914	-0.03337	0.998356
Fam116a	211922	0.122123	0.998356
Plekhh1	211945	-0.08684	0.998356
Pde12	211948	-0.1736	0.998356
Zfyve26	211978	0.18882	0.998356
Tmem18	211986	0.530859	0.998356
Tmem60	212090	-0.0599	0.998356
Inpp5a	212111	1.28156	0.998356
Nhlrc3	212114	0.272719	0.998356
Dcaf15	212123	0.334371	0.998356
2810046L04Rik	212127	-0.11615	0.998356
Cc2d1a	212139	0.108799	0.998356
2610015P09Rik	212153	-0.08524	0.998356
8030462N17Rik	212163	0.271783	0.998356
Pion	212167	0.27234	0.998356
Zswim4	212168	0.197236	0.998356
Ubxn10	212190	0.097993	0.998356
Zfp748	212276	0.182073	0.998356
A530054K11Rik	212281	0.206794	0.998356
Arap2	212285	0.28604	0.998356
Mapre2	212307	0.077229	0.998356
Mms22l	212377	-0.07206	0.998356
Lcor	212391	-0.33381	0.998356
A730008H23Rik	212427	-0.04318	0.998356
AA986860	212439	0.094709	0.998356
Fam193b	212483	-0.32311	0.998356
Paox	212503	-0.61598	0.998356
Mtg1	212508	-0.3248	0.998356
Spice1	212514	-0.55603	0.998356
Trmt1	212528	0.633566	0.998356
Sh3bgrl2	212531	0.307369	0.998356
Iffo2	212632	-0.16605	0.998356
Aldh4a1	212647	-0.23336	0.998356
Mars2	212679	-0.34844	0.998356
N4bp3	212706	0.089748	0.998356
Satb2	212712	0.260524	0.998356
Ccdc64b	212733	-0.23323	0.998356
2700007P21Rik	212772	0.055963	0.998356
Chpt1	212862	0.090034	0.998356
Ddx46	212880	-0.09733	0.998356
Fam46a	212943	-0.41339	0.998356
Athl1	212974	-0.14255	0.998356
Slc45a3	212980	-0.98101	0.998356
Best2	212989	-3.15844	0.998356
BC016579	212998	0.240637	0.998356
Tnpo2	212999	-0.30444	0.998356
Ifitm6	213002	-2.66759	0.998356

Abhd10	213012	-0.00733	0.998356
Pdlim2	213019	0.572076	0.998356
Evi5l	213027	0.201873	0.998356
Slc39a14	213053	0.17668	0.998356
Gabpb2	213054	-0.56097	0.998356
Fam126b	213056	0.30233	0.998356
Wdr19	213081	-0.09249	0.998356
Phf3	213109	0.181236	0.998356
Prss27	213171	-0.66562	0.998356
Rnf26	213211	-0.21251	0.998356
Tapbpl	213233	0.108709	0.998356
Scyl2	213326	-0.09251	0.998356
Pddc1	213350	0.459668	0.998356
Rassf4	213391	-0.60032	0.998356
8430408G22Rik	213393	-10	0.998356
Dstyk	213452	0.311677	0.998356
Rbbp5	213464	-0.39152	0.998356
D4Ertd22e	213491	0.019096	0.998356
Arhgef11	213498	0.136434	0.998356
Fbxo42	213499	-0.06865	0.998356
Plekhg6	213522	0.738371	0.998356
Bag2	213539	-0.2284	0.998356
Ythdf2	213541	-0.31941	0.998356
Dis3l	213550	-0.12194	0.998356
Plekhh2	213556	0.016707	0.998356
Efcab4a	213573	-0.28626	0.998356
Slc44a3	213603	0.02839	0.998356
Arhgef19	213649	-0.0582	0.998356
9530068E07Rik	213673	-0.16259	0.998356
Zfp598	213753	0.203076	0.998356
Prepl	213760	-0.22355	0.998356
Tbl3	213773	0.454533	0.998356
Plekhg1	213783	-0.29765	0.998356
Casd1	213819	-0.05311	0.998356
Arcn1	213827	0.008664	0.998356
Bms1	213895	0.036155	0.998356
Atg9b	213948	-0.01457	0.998356
Fam83f	213956	0.00206	0.998356
Tnrc6b	213988	0.374215	0.998356
Agap3	213990	0.010972	0.998356
A630007B06Rik	213993	0.38992	0.998356
Larp1b	214048	0.235409	0.998356
Dnajc16	214063	0.343071	0.998356
Tet2	214133	0.091045	0.998356
Arhgap29	214137	0.601048	0.998356
Eif2c3	214150	-0.34455	0.998356
Mll1	214162	-0.09623	0.998356

A430105I19Rik	214239	-0.41916	0.998356
Zcchc6	214290	0.28916	0.998356
Lrrc1	214345	-0.21294	0.998356
Tmem51	214359	-0.10275	0.998356
Parp16	214424	-0.07778	0.998356
Cdk5rap2	214444	-0.03097	0.998356
Fnbp1l	214459	-0.07669	0.998356
Fam168b	214469	-0.48346	0.998356
BC003965	214489	-0.386	0.998356
Cdc73	214498	0.101288	0.998356
Gnptg	214505	0.476282	0.998356
Tmprss4	214523	0.17757	0.998356
Tmprss13	214531	-0.19716	0.998356
Cep164	214552	-0.1008	0.998356
Prmt7	214572	0.15865	0.998356
Aldh5a1	214579	-0.02505	0.998356
Pstk	214580	-0.10106	0.998356
Spg11	214585	-0.02015	0.998356
Sidt2	214597	0.077316	0.998356
Slc10a3	214601	-0.08843	0.998356
Papd5	214627	0.948644	0.998356
L3mbtl2	214669	-0.1692	0.998356
Rcor3	214742	0.492179	0.998356
2700050L05Rik	214764	-0.47218	0.998356
Zfp609	214812	-0.18331	0.998356
Neurl3	214854	0.020449	0.998356
Arid5a	214855	0.03918	0.998356
Csnk1g1	214897	0.028804	0.998356
Kdm5a	214899	0.047851	0.998356
Chtf18	214901	-0.56258	0.998356
Fam173a	214917	2.66E-05	0.998356
Cecr5	214932	-0.07187	0.998356
Mob3b	214944	0.053218	0.998356
Rhot2	214952	-0.06527	0.998356
Chtf8	214987	-0.36609	0.998356
Vezt	215008	0.198534	0.998356
Fam20b	215015	0.157174	0.998356
Bud13	215051	-0.27771	0.998356
Rhbdd2	215160	-0.11581	0.998356
Kri1	215194	-0.50368	0.998356
Trmt2b	215201	0.484147	0.998356
Tmem120a	215210	0.772203	0.998356
Il1f9	215257	-0.07688	0.998356
Wipf1	215280	-0.11487	0.998356
Slc36a1	215335	-0.16378	0.998356
Senp6	215351	0.257613	0.998356
Fcgbp	215384	0.474579	0.998356

Ncaph	215387	-0.26043	0.998356
Csrnp1	215418	-0.06763	0.998356
Rab11fip3	215445	-1.11833	0.998356
Entpd3	215446	-0.06988	0.998356
Rap1b	215449	0.496594	0.998356
C330019G07Rik	215476	-0.0946	0.998356
C85492	215494	-0.47653	0.998356
Rnpep	215615	-0.2515	0.998356
Psd4	215632	0.240982	0.998356
Nav1	215690	-0.15112	0.998356
Arrdc1	215705	0.202043	0.998356
Ccdc92	215707	-0.45027	0.998356
Fam73a	215708	-0.96632	0.998356
Phactr2	215789	0.002481	0.998356
Ccdc28a	215814	0.561519	0.998356
Nhsl1	215819	0.052218	0.998356
D10Bwg1379e	215821	-0.32113	0.998356
Lace1	215951	0.099105	0.998356
Ccdc109a	215999	0.110594	0.998356
Micu1	216001	-0.07535	0.998356
Lrrc20	216011	-0.20659	0.998356
Ube2d1	216080	-0.2571	0.998356
Ybey	216119	-0.12556	0.998356
Trappc10	216131	0.344983	0.998356
Pdxk	216134	-0.43564	0.998356
Ilvbl	216136	0.222302	0.998356
Polrmt	216151	-0.21262	0.998356
Med16	216154	0.035362	0.998356
Wdr18	216156	-0.36495	0.998356
Sbno2	216161	0.302057	0.998356
Fam108a	216169	0.558381	0.998356
Appl2	216190	0.203405	0.998356
Ckap4	216197	-0.04611	0.998356
Tcp11l2	216198	0.757322	0.998356
Socs2	216233	0.899882	0.998356
Eea1	216238	0.251588	0.998356
Cep290	216274	-0.17069	0.998356
Rab21	216344	-0.06137	0.998356
Zfc3h1	216345	0.228847	0.998356
Tspan8	216350	0.499556	0.998356
Rab3ip	216363	-0.03359	0.998356
Tmem5	216395	0.393532	0.998356
Os9	216440	0.0757	0.998356
Mars	216443	-0.06989	0.998356
Gls2	216456	-0.56642	0.998356
Pik3ip1	216505	-0.11392	0.998356
Ccm2	216527	-0.24043	0.998356

Cep68	216543	-0.20419	0.998356
Aftph	216549	0.08813	0.998356
1110067D22Rik	216551	0.328792	0.998356
Ugp2	216558	0.06429	0.998356
Papolg	216578	0.21168	0.998356
Ccdc104	216618	-0.1156	0.998356
Clint1	216705	0.06863	0.998356
Rufy1	216724	0.208074	0.998356
Fnip1	216742	-0.01384	0.998356
Mfap3	216760	0.171549	0.998356
Gemin5	216766	-0.43329	0.998356
Mrpl22	216767	0.163136	0.998356
A230051G13Rik	216792	0.211347	0.998356
Nlrp3	216799	-0.42584	0.998356
Flcn	216805	0.138028	0.998356
Tom1l2	216810	1.78454	0.998356
Dhrs7b	216820	9.54155	0.998356
Tmem11	216821	-0.06663	0.998356
Usp22	216825	0.202009	0.998356
Mmgt2	216829	-0.15535	0.998356
Usp43	216835	0.245495	0.998356
Cntrob	216846	-0.11829	0.998356
Chd3	216848	0.03095	0.998356
Kdm6b	216850	-0.202	0.998356
Wrap53	216853	0.259616	0.998356
Nlgn2	216856	0.36031	0.998356
Kctd11	216858	0.006736	0.998356
Neurl4	216860	0.054153	0.998356
Arrb2	216869	-0.16847	0.998356
Spag7	216873	-0.32814	0.998356
Camta2	216874	-0.13965	0.998356
Dhx33	216877	-0.09626	0.998356
Git1	216963	0.177863	0.998356
Taok1	216965	0.017705	0.998356
Utp6	216987	-0.16158	0.998356
Nle1	217011	0.441666	0.998356
Heatr6	217026	-0.36937	0.998356
Synrg	217030	0.278503	0.998356
Tada2a	217031	-0.1074	0.998356
Ggnbp2	217039	0.07007	0.998356
Ptrh2	217057	0.285955	0.998356
Trim25	217069	0.03281	0.998356
Utp18	217109	-0.03764	0.998356
Xylt2	217119	0.129311	0.998356
Gm11545	217122	0.525298	0.998356
Ppp1r9b	217124	0.01207	0.998356
Myst2	217127	-0.44622	0.998356

Prr15l	217138	0.043966	0.998356
Scrn2	217140	0.091542	0.998356
Cisd3	217149	-0.39301	0.998356
Nr1d1	217166	0.325074	0.998356
Tns4	217169	-0.01271	0.998356
Klhl11	217194	-0.13543	0.998356
Plekhh3	217198	0.301244	0.998356
Rundc1	217201	-0.42031	0.998356
Tmem106a	217203	0.282692	0.998356
Dhx8	217207	-0.15701	0.998356
Pyy	217212	-1.02829	0.998356
BC030867	217216	-0.64584	0.998356
Atxn7l3	217218	-0.03753	0.998356
Cdc27	217232	-0.30659	0.998356
C630004H02Rik	217310	-0.04368	0.998356
Llgl2	217325	-0.10923	0.998356
Unk	217331	-0.07791	0.998356
Fbf1	217335	-0.18236	0.998356
Srp68	217337	-0.0164	0.998356
Ube2o	217342	-0.15826	0.998356
Rhbdf2	217344	-0.26746	0.998356
Tnrc6c	217351	0.121038	0.998356
Tmc6	217353	0.606187	0.998356
Engase	217364	0.106073	0.998356
Nploc4	217365	0.117488	0.998356
Lrrc45	217366	0.03288	0.998356
BC017643	217370	-0.09518	0.998356
Ubxn2a	217379	0.261992	0.998356
Trib2	217410	0.47391	0.998356
Pqlc3	217430	0.323248	0.998356
Nol10	217431	-0.40455	0.998356
Ttc15	217449	-0.17217	0.998356
Snx13	217463	0.094629	0.998356
Ankmy2	217473	0.394279	0.998356
Stxbp6	217517	-0.17194	0.998356
G2e3	217558	-0.03915	0.998356
Baz1a	217578	0.158143	0.998356
Mbip	217588	0.554296	0.998356
Ctage5	217615	-0.93204	0.998356
Mis18bp1	217653	-0.1807	0.998356
Mgat2	217664	0.100969	0.998356
L2hgdh	217666	-0.0256	0.998356
4933426M11Rik	217684	-0.22776	0.998356
Sipa1l1	217692	-0.34628	0.998356
Zfyve1	217695	0.18275	0.998356
Coq6	217707	0.097462	0.998356
Lin52	217708	-0.08649	0.998356

Eif2b2	217715	-0.29919	0.998356
Mlh3	217716	-0.37072	0.998356
Nek9	217718	0.206863	0.998356
2310044G17Rik	217732	0.09352	0.998356
Pomt2	217734	-0.44435	0.998356
Ahsa1	217737	-0.30812	0.998356
9030617O03Rik	217830	-0.33625	0.998356
Rin3	217835	2.59224	0.998356
Itpk1	217837	0.497051	0.998356
Rcor1	217864	-0.05478	0.998356
Cdc42bpb	217866	0.273884	0.998356
Eif5	217869	-0.07334	0.998356
AW555464	217882	-0.03567	0.998356
Pacs2	217893	0.062196	0.998356
Wdr60	217935	-0.03889	0.998356
Rapgef5	217944	-0.76805	0.998356
Cdca7l	217946	-0.48794	0.998356
Larp4b	217980	-0.01759	0.998356
Heatr1	217995	-0.3391	0.998356
Vps41	218035	0.138509	0.998356
Zfp322a	218100	-0.1008	0.998356
Gmds	218138	0.084876	0.998356
Mylip	218203	0.137058	0.998356
Nup153	218210	-0.0341	0.998356
Kdm1b	218214	-0.34007	0.998356
Fam120a	218236	-0.06052	0.998356
B4galt7	218271	-0.23139	0.998356
Cdc14b	218294	0.376682	0.998356
Zfp595	218314	-0.21507	0.998356
Clptm1l	218335	0.033304	0.998356
Ttc37	218343	-0.19737	0.998356
Rasa1	218397	0.099358	0.998356
Zfyve16	218441	0.072334	0.998356
Serinc5	218442	0.275919	0.998356
Wdr41	218460	-0.31505	0.998356
Pde8b	218461	-0.09993	0.998356
Btf3	218490	-0.01561	0.998356
Fcho2	218503	0.055561	0.998356
Mrps27	218506	0.211606	0.998356
Marveld2	218518	0.25516	0.998356
Srek1	218543	-0.07568	0.998356
Depdc1b	218581	0.416703	0.998356
Mier3	218613	0.146405	0.998356
Dhx29	218629	0.082699	0.998356
Arl15	218639	-0.12415	0.998356
Paip1	218693	0.164555	0.998356
Pxk	218699	0.127045	0.998356

3830406C13Rik	218734	0.090143	0.998356
Slc4a7	218756	-0.01994	0.998356
Rarb	218772	-0.66945	0.998356
Ube2e2	218793	0.388963	0.998356
Sec24c	218811	-0.04918	0.998356
Zfp503	218820	-0.22396	0.998356
Polr3a	218832	0.131401	0.998356
D14Abb1e	218850	0.347275	0.998356
Chdh	218865	0.086603	0.998356
Oxnad1	218885	-0.32559	0.998356
Wapal	218914	0.191589	0.998356
Fermt2	218952	0.244905	0.998356
Wdhd1	218973	-0.26027	0.998356
Mapk1ip1l	218975	-0.07638	0.998356
Dlgap5	218977	-0.25116	0.998356
6720456H20Rik	218989	0.066532	0.998356
Ttc5	219022	0.667561	0.998356
Tmem55b	219024	0.250597	0.998356
Ang4	219033	-0.20595	0.998356
Haus4	219072	0.106948	0.998356
Khnyn	219094	-0.00734	0.998356
Cenpj	219103	-0.06772	0.998356
Zmym5	219105	-2.1843	0.998356
F630043A04Rik	219114	-0.33176	0.998356
Shisa2	219134	0.440771	0.998356
Mtmr6	219135	0.313672	0.998356
Spata13	219140	-0.13017	0.998356
Fam167a	219148	-0.53392	0.998356
Hmbox1	219150	-0.1547	0.998356
Scara3	219151	0.534865	0.998356
2610301G19Rik	219158	-0.36665	0.998356
Akap11	219181	0.043363	0.998356
1300010F03Rik	219189	0.181928	0.998356
Tdrd3	219249	0.330594	0.998356
Sox21	223227	0.172441	0.998356
Farp1	223254	0.173532	0.998356
Stk24	223255	0.251098	0.998356
A2ld1	223267	-0.42968	0.998356
Trio	223435	0.207865	0.998356
Dap	223453	-0.02173	0.998356
Nipal2	223473	0.203202	0.998356
Dcaf13	223499	0.243101	0.998356
Eny2	223527	-0.2159	0.998356
E430025E21Rik	223593	0.090482	0.998356
Fam49b	223601	0.167327	0.998356
4930572J05Rik	223626	-0.396	0.998356
Zc3h3	223642	-0.00572	0.998356

Naprt1	223646	0.090874	0.998356
2410075B13Rik	223648	0.034473	0.998356
Nrbp2	223649	0.457445	0.998356
Eppk1	223650	-0.2543	0.998356
Heatr7a	223658	0.075149	0.998356
Lrrc14	223664	-0.18117	0.998356
Arhgap39	223666	-0.83156	0.998356
Zfp7	223669	-0.04631	0.998356
Ankrd54	223690	0.04942	0.998356
Eif3l	223691	-0.39647	0.998356
Tmem184b	223693	0.050698	0.998356
Tomm22	223696	-0.24833	0.998356
Sun2	223697	-0.84245	0.998356
Mkl1	223701	-0.12963	0.998356
Mcat	223722	0.328514	0.998356
Ttll12	223723	0.306592	0.998356
5031439G07Rik	223739	-0.25841	0.998356
Gramd4	223752	0.083415	0.998356
Cerk	223753	0.234269	0.998356
Tbc1d22a	223754	0.359719	0.998356
Brd1	223770	0.148736	0.998356
Zbed4	223773	-0.13156	0.998356
Alg12	223774	1.14077	0.998356
Pim3	223775	-0.06694	0.998356
1300018J18Rik	223776	0.525814	0.998356
Gxylt1	223827	0.025568	0.998356
Pphln1	223828	-0.29267	0.998356
Senp1	223870	-0.16012	0.998356
Spryd3	223918	-0.01246	0.998356
Aaas	223921	0.291202	0.998356
Atf7	223922	0.079994	0.998356
Cpped1	223978	-0.16163	0.998356
4921513D23Rik	223989	-0.20298	0.998356
2310008H04Rik	224008	-0.45276	0.998356
Fgd4	224014	0.462644	0.998356
Pi4ka	224020	0.050128	0.998356
Slc7a4	224022	0.251262	0.998356
Klhl22	224023	0.038281	0.998356
Eif2b5	224045	-0.07232	0.998356
Atp13a3	224088	-0.13696	0.998356
Lsg1	224092	-0.03027	0.998356
Fam43a	224093	-0.08317	0.998356
Pak2	224105	0.118237	0.998356
Ubxn7	224111	-0.15616	0.998356
Muc20	224116	0.059278	0.998356
Dirc2	224132	0.505164	0.998356
Poglut1	224143	-0.28788	0.998356

C330027C09Rik	224171	-0.08884	0.998356
Crybg3	224273	-0.21746	0.998356
Rbm11	224344	0.161854	0.998356
Scaf4	224432	0.185708	0.998356
Zdhhc14	224454	0.194651	0.998356
Tfb1m	224481	0.054608	0.998356
Zfp160	224585	0.104154	0.998356
Zfp758	224598	0.418962	0.998356
Flywch1	224613	0.028218	0.998356
Traf7	224619	-0.29009	0.998356
Rab40c	224624	-0.24083	0.998356
Bnip1	224630	-0.52808	0.998356
Lemd2	224640	0.055018	0.998356
D17Wsu92e	224647	0.045121	0.998356
Uhrf1bp1	224648	-0.18269	0.998356
Anks1	224650	-0.05088	0.998356
Zfp523	224656	0.037983	0.998356
Btbd9	224671	0.075542	0.998356
Slc37a1	224674	0.073881	0.998356
Zfp472	224691	0.451477	0.998356
Zfp81	224694	-0.09742	0.998356
Vps52	224705	-0.3896	0.998356
Bag6	224727	0.102432	0.998356
Abcf1	224742	-0.00487	0.998356
Trim31	224762	0.573951	0.998356
Enpp4	224794	-0.29693	0.998356
Clic5	224796	0.442456	0.998356
Aars2	224805	0.081912	0.998356
Tmem63b	224807	0.119919	0.998356
Rrp36	224823	-0.45029	0.998356
Pex6	224824	-0.11894	0.998356
Ubr2	224826	-0.82987	0.998356
AI661453	224833	0.143002	0.998356
Usp49	224836	-0.16046	0.998356
Plcl2	224860	0.243643	0.998356
Zfp959	224893	0.027047	0.998356
Dpp9	224897	0.159815	0.998356
Safb2	224902	-0.12078	0.998356
Safb	224903	-0.55344	0.998356
Dus3l	224907	-0.21856	0.998356
Crb3	224912	0.022627	0.998356
Pja2	224938	0.865968	0.998356
Lclat1	225010	0.174403	0.998356
Fez2	225020	0.230893	0.998356
Srsf7	225027	0.281072	0.998356
Map4k3	225028	0.126391	0.998356
Ttc7	225049	0.16344	0.998356

Fbxo11	225055	0.140772	0.998356
Svil	225115	-0.08774	0.998356
Wac	225131	0.536878	0.998356
Thoc1	225160	-0.43474	0.998356
Mib1	225164	0.186861	0.998356
Rbbp8	225182	-0.27357	0.998356
Ankrd29	225187	0.597939	0.998356
Rsl24d1	225215	0.011718	0.998356
Ino80c	225280	0.202936	0.998356
Rprd1a	225283	-0.31366	0.998356
Fhod3	225288	0.377233	0.998356
AW554918	225289	-0.17592	0.998356
Pik3c3	225326	-0.06179	0.998356
Ammecr1l	225339	0.234951	0.998356
Wdr36	225348	0.148591	0.998356
Fam13b	225358	-0.04162	0.998356
Etf1	225363	-0.02977	0.998356
Apbb3	225372	-0.274	0.998356
Rbm27	225432	-0.32942	0.998356
Pggt1b	225467	0.159309	0.998356
Cep120	225523	0.152765	0.998356
Sh3tc2	225608	0.343448	0.998356
Mppe1	225651	0.086281	0.998356
Mapk4	225724	-0.24259	0.998356
Haus1	225745	-0.25536	0.998356
Zadh2	225791	0.244056	0.998356
Pla2g16	225845	0.320955	0.998356
Ppp2r5b	225849	0.052314	0.998356
Lrfn4	225875	0.452079	0.998356
Kdm2a	225876	-0.12848	0.998356
Ndufs8	225887	-0.12154	0.998356
Suv420h1	225888	0.037433	0.998356
Taf6l	225895	-0.80951	0.998356
Ubxn1	225896	-0.12823	0.998356
Eml3	225898	0.432271	0.998356
Gm98	225908	-0.34261	0.998356
Cybasc3	225912	0.042047	0.998356
Dak	225913	-0.25094	0.998356
Patl1	225929	0.035488	0.998356
D030056L22Rik	225995	-0.21272	0.998356
Fam108b	226016	0.560985	0.998356
Smc5	226026	0.819858	0.998356
Cbwd1	226043	0.050282	0.998356
Glis3	226075	0.242189	0.998356
C030046E11Rik	226089	-0.01364	0.998356
Ermp1	226090	0.048712	0.998356
Myof	226101	0.166608	0.998356

Ubtd1	226122	-0.43811	0.998356
Cox15	226139	0.246803	0.998356
Fam178a	226151	-0.33256	0.998356
Peo1	226153	-0.28636	0.998356
Lzts2	226154	-0.02868	0.998356
Dpcd	226162	-0.1766	0.998356
Pprc1	226169	-0.25647	0.998356
D19Wsu162e	226178	-0.25878	0.998356
Habp2	226243	-0.63654	0.998356
9930023K05Rik	226245	-0.1353	0.998356
Ablim1	226251	-0.24669	0.998356
Fam160b1	226252	0.237485	0.998356
Atrnl1	226255	0.348137	0.998356
Tmem185b	226351	0.373641	0.998356
Rab3gap1	226407	0.292804	0.998356
R3hdm1	226412	-0.14903	0.998356
Dars	226414	-1.7631	0.998356
Yod1	226418	-0.30533	0.998356
Dyrk3	226419	0.405046	0.998356
Rab7l1	226422	-0.17847	0.998356
Ipo9	226432	-0.41918	0.998356
Zfp281	226442	-0.33145	0.998356
Zbtb41	226470	-0.24428	0.998356
BC003331	226499	0.210269	0.998356
Smg7	226517	0.323724	0.998356
Lamc1	226519	0.172383	0.998356
Rasal2	226525	0.029309	0.998356
Dars2	226539	-0.15586	0.998356
Klhl20	226541	0.017801	0.998356
Aph1a	226548	0.435549	0.998356
AI848100	226551	0.128207	0.998356
Prrc2c	226562	-0.09623	0.998356
Tiprl	226591	-0.16733	0.998356
Atf6	226641	0.192987	0.998356
Ndufs2	226646	0.284016	0.998356
Arhgap30	226652	-0.09282	0.998356
Tstd1	226654	0.681112	0.998356
Cnst	226744	0.052877	0.998356
Ahctf1	226747	-0.03217	0.998356
Cdc42bpa	226751	-0.2996	0.998356
Wdr26	226757	0.402896	0.998356
C130074G19Rik	226777	0.437731	0.998356
Mark1	226778	0.089978	0.998356
Lyplal1	226791	0.427486	0.998356
Kctd3	226823	0.053504	0.998356
Smyd2	226830	0.060433	0.998356
Vash2	226841	-0.80399	0.998356

Mfsd7b	226844	0.256537	0.998356
Ppp2r5a	226849	0.498163	0.998356
Lpgat1	226856	0.026318	0.998356
Gm106	226866	0.27372	0.998356
Plekhb2	226971	0.22969	0.998356
4632411B12Rik	226976	-0.16568	0.998356
Actr1b	226977	0.397804	0.998356
Eif5b	226982	-0.19862	0.998356
Slc39a10	227059	-0.4669	0.998356
Hibch	227095	0.010312	0.998356
Pms1	227099	-0.31948	0.998356
Ormdl1	227102	0.164678	0.998356
Stradb	227154	-0.24868	0.998356
Ino80d	227195	0.678373	0.998356
Ndufs1	227197	-0.2108	0.998356
Ccnyl1	227210	-0.25129	0.998356
Aamp	227290	-0.7877	0.998356
Ctdsp1	227292	0.372137	0.998356
Fam134a	227298	0.431563	0.998356
Gpr55	227326	-0.04589	0.998356
B3gnt7	227327	-0.14982	0.998356
Gigyf2	227331	-0.26023	0.998356
Dgkd	227333	-0.10615	0.998356
Usp40	227334	-0.18601	0.998356
Fam132b	227358	-0.40337	0.998356
Farp2	227377	-0.12354	0.998356
Ppip5k2	227399	-0.2006	0.998356
2310035C23Rik	227446	0.128729	0.998356
Zcchc2	227449	-0.24858	0.998356
Rpp38	227522	0.118202	0.998356
Dclre1c	227525	-0.19091	0.998356
Camk1d	227541	-0.54784	0.998356
5430407P10Rik	227545	1.58078	0.998356
A830007P12Rik	227612	0.075911	0.998356
Tubb4b	227613	0.152156	0.998356
Tmem203	227615	-0.02233	0.998356
Lrrc26	227618	-0.10027	0.998356
Man1b1	227619	0.336769	0.998356
Uap1l1	227620	0.366411	0.998356
BC029214	227622	0.451654	0.998356
B230208H17Rik	227624	-0.01026	0.998356
Camsap1	227634	-0.21769	0.998356
Qsox2	227638	-0.13676	0.998356
Snapc4	227644	0.396722	0.998356
Sec16a	227648	-0.05811	0.998356
Rexo4	227656	-0.34444	0.998356
Ddx31	227674	-0.57353	0.998356

Trub2	227682	0.467472	0.998356
Coq4	227683	-0.04751	0.998356
Zer1	227693	0.301952	0.998356
D2Wsu81e	227695	-0.08746	0.998356
Dolk	227697	-0.06204	0.998356
Nup188	227699	-0.22547	0.998356
Sh3glb2	227700	0.401122	0.998356
BC005624	227707	0.055671	0.998356
Exosc2	227715	-0.33004	0.998356
Nup214	227720	-0.17752	0.998356
Prrc2b	227723	-0.53071	0.998356
Slc25a25	227731	0.092759	0.998356
Fam129b	227737	0.225831	0.998356
Lrsam1	227738	0.19953	0.998356
Mapkap1	227743	0.263118	0.998356
Rabepk	227746	-0.08777	0.998356
Gsn	227753	-1.2496	0.998356
Rabgap1	227800	0.072212	0.998356
Dennd1a	227801	0.151365	0.998356
Gtdc1	227835	-0.17267	0.998356
Epc2	227867	0.14654	0.998356
Pkp4	227937	0.269608	0.998356
Gca	227960	-0.56367	0.998356
Ppig	228005	-0.17687	0.998356
Tlk1	228012	0.057699	0.998356
Mettl8	228019	0.128773	0.998356
Pdk1	228026	0.044546	0.998356
Atp5g3	228033	0.087455	0.998356
Agps	228061	-0.05137	0.998356
Sestd1	228071	-0.11597	0.998356
Zdhhc5	228136	0.143539	0.998356
Tnks1bp1	228140	0.005418	0.998356
Madd	228355	-0.36991	0.998356
1110051M20Rik	228356	0.215349	0.998356
Lrp4	228357	0.220496	0.998356
Arhgap1	228359	-0.18437	0.998356
Ambra1	228361	0.927103	0.998356
Gyltl1b	228366	0.046468	0.998356
Slc35c1	228368	0.148545	0.998356
Cstf3	228410	0.265152	0.998356
Prrg4	228413	-0.10881	0.998356
Kif18a	228421	0.166668	0.998356
Arhgap11a	228482	-0.26595	0.998356
Zfp770	228491	-0.46484	0.998356
Bahd1	228536	-0.12899	0.998356
Vps18	228545	0.130009	0.998356
Mall	228576	0.318629	0.998356

4930402H24Rik	228602	-0.02761	0.998356
Mavs	228607	-0.29121	0.998356
Smox	228608	-0.35434	0.998356
Btbd3	228662	-0.281	0.998356
Csrp2bp	228714	0.011673	0.998356
Gm561	228715	-0.1724	0.998356
Plk1s1	228730	0.164906	0.998356
Sdcbp2	228765	0.356078	0.998356
Psmf1	228769	0.222778	0.998356
Asxl1	228790	0.207445	0.998356
Pigu	228812	-0.03639	0.998356
Phf20	228829	-0.02744	0.998356
Dlgap4	228836	-0.25526	0.998356
Tgif2	228839	-0.51709	0.998356
Ralgapb	228850	0.094733	0.998356
Fitm2	228859	-0.4034	0.998356
Pcif1	228866	0.249046	0.998356
Ncoa5	228869	-0.07616	0.998356
Slc35c2	228875	-0.06418	0.998356
Zmynd8	228880	-0.05323	0.998356
Ddx27	228889	-0.21809	0.998356
Tshz2	228911	0.107803	0.998356
Zfp217	228913	-0.1193	0.998356
Stx16	228960	1.06937	0.998356
Npepl1	228961	-0.00921	0.998356
Taf4a	228980	-0.08747	0.998356
Osbpl2	228983	0.497759	0.998356
Slc17a9	228993	0.68332	0.998356
Ythdf1	228994	0.03182	0.998356
Arfgap1	228998	-0.85294	0.998356
Gmeb2	229004	0.139739	0.998356
Zgpat	229007	-0.24924	0.998356
Samd10	229011	0.219331	0.998356
Ythdf3	229096	0.104003	0.998356
Acad9	229211	-0.09282	0.998356
4932438A13Rik	229227	0.053577	0.998356
Nudt6	229228	0.35904	0.998356
Hnrnpa3	229279	-0.3023	0.998356
Spg20	229285	0.4308	0.998356
Tm4sf4	229302	-0.63362	0.998356
Eif2a	229317	0.120893	0.998356
Gmps	229363	-0.11792	0.998356
Ctso	229445	0.126072	0.998356
D930015E06Rik	229473	0.08002	0.998356
Fhdc1	229474	-0.06257	0.998356
Pet112l	229487	-0.25033	0.998356
Fam160a1	229488	0.325053	0.998356

Rrnad1	229503	0.176912	0.998356
Isg20l2	229504	0.152352	0.998356
Smg5	229512	0.201913	0.998356
Slc25a44	229517	1.49368	0.998356
Syt11	229521	0.282528	0.998356
Msto1	229524	0.360463	0.998356
Pbxip1	229534	-0.17983	0.998356
Gatad2b	229542	0.58894	0.998356
Ints3	229543	-0.26496	0.998356
Pogz	229584	-1.38863	0.998356
Prune	229589	0.182347	0.998356
Golph3l	229593	0.019646	0.998356
Adamtsl4	229595	-0.12093	0.998356
Otud7b	229603	0.142415	0.998356
Pias3	229615	1.39782	0.998356
Vangl1	229658	0.102115	0.998356
Csde1	229663	0.473757	0.998356
Bcl2l15	229672	0.689618	0.998356
Rsbn1	229675	-0.27624	0.998356
St7l	229681	-0.33973	0.998356
Rbm15	229700	-0.00038	0.998356
Fam40a	229707	0.293718	0.998356
Ahcyl1	229709	0.332889	0.998356
Amigo1	229715	-0.019	0.998356
5330417C22Rik	229722	-0.0744	0.998356
Clcc1	229725	-0.0728	0.998356
Slc25a24	229731	0.212176	0.998356
Cdc14a	229776	0.485427	0.998356
Ccdc76	229780	-0.83013	0.998356
Slc35a3	229782	0.057748	0.998356
Cenpe	229841	-0.42576	0.998356
Rap1gds1	229877	0.38138	0.998356
Gbp5	229898	-0.44314	0.998356
Gbp7	229900	-0.28816	0.998356
Gtf2b	229906	0.276564	0.998356
Znhit6	229937	-0.10093	0.998356
Coq3	230027	0.219282	0.998356
Ddx58	230073	0.368933	0.998356
Nol6	230082	-0.06596	0.998356
B230312A22Rik	230088	0.432	0.998356
E130306D19Rik	230098	0.151274	0.998356
Car9	230099	0.407364	0.998356
Gba2	230101	-0.2408	0.998356
Zbtb5	230119	-0.1313	0.998356
Mcart1	230125	0.167104	0.998356
Shb	230126	-0.11694	0.998356
Galnt12	230145	0.073486	0.998356

Tmeff1	230157	0.422525	0.998356
Acnat1	230161	-0.07821	0.998356
Aldob	230163	-0.10237	0.998356
Ikbkap	230233	0.057804	0.998356
AI314180	230249	0.18207	0.998356
Rod1	230257	-0.36155	0.998356
E130308A19Rik	230259	0.038723	0.998356
6330416G13Rik	230279	-0.03741	0.998356
Megf9	230316	-0.1305	0.998356
Haus6	230376	0.075224	0.998356
Usp1	230484	-0.06313	0.998356
Leprot	230514	0.2309	0.998356
Ttc22	230576	0.61121	0.998356
Pars2	230577	-1.39591	0.998356
Cyb5rl	230582	-0.17057	0.998356
Yipf1	230584	-0.07881	0.998356
Zcchc11	230594	0.055222	0.998356
Prpf38a	230596	0.189586	0.998356
Zfyve9	230597	0.266653	0.998356
Nrd1	230598	0.14102	0.998356
Ttc39a	230603	-0.01034	0.998356
Slc5a9	230612	-0.4046	0.998356
4732418C07Rik	230648	-0.03439	0.998356
Atpaf1	230649	0.04988	0.998356
Lrrc41	230654	0.142747	0.998356
Tmem69	230657	0.135862	0.998356
Tesk2	230661	0.10584	0.998356
Ipo13	230673	-0.12035	0.998356
Kdm4a	230674	0.429816	0.998356
Szt2	230676	-0.02732	0.998356
Tmem125	230678	-0.23026	0.998356
AU022252	230696	-0.15035	0.998356
Foxj3	230700	-0.1663	0.998356
Zmpste24	230709	0.077174	0.998356
Pabpc4	230721	-0.66496	0.998356
Rhbdl2	230726	0.499934	0.998356
Yrdc	230734	0.420017	0.998356
Gnl2	230737	-0.20585	0.998356
Zc3h12a	230738	-0.21676	0.998356
Fam176b	230752	0.705492	0.998356
Thrap3	230753	-0.19546	0.998356
Zfp362	230761	0.101356	0.998356
Iqcc	230767	0.222949	0.998356
Tmem39b	230770	0.064146	0.998356
Serinc2	230779	-0.16896	0.998356
Sesn2	230784	0.221404	0.998356
Fam76a	230789	0.67806	0.998356

Ahdc1	230793	-0.16921	0.998356
Wdtc1	230796	-0.1101	0.998356
Pigv	230801	0.70917	0.998356
Aim1l	230806	0.141595	0.998356
Pdik1l	230809	-0.10707	0.998356
Slc30a2	230810	0.117028	0.998356
A330049M08Rik	230822	0.400031	0.998356
Grhl3	230824	0.258358	0.998356
Il22ra1	230828	0.385684	0.998356
Zbtb40	230848	-0.6659	0.998356
Ece1	230857	0.00582	0.998356
Eif4g3	230861	-1.06049	0.998356
Vps13d	230895	0.277725	0.998356
Fbxo44	230903	-0.73002	0.998356
Tardbp	230908	-0.12743	0.998356
Dnajc11	230935	-0.24769	0.998356
Phf13	230936	0.485126	0.998356
Arhgef16	230972	0.211427	0.998356
Tnfrsf14	230979	0.353262	0.998356
9430015G10Rik	230996	1.79642	0.998356
Plekhn1	231002	0.478018	0.998356
Nupl2	231042	-0.31394	0.998356
Galnt11	231050	0.068443	0.998356
Mll3	231051	0.014864	0.998356
Insig1	231070	0.200429	0.998356
Hadhb	231086	0.226025	0.998356
Agbl5	231093	-0.18599	0.998356
Haus3	231123	-0.48828	0.998356
Fam193a	231128	-0.02839	0.998356
Tnip2	231130	-0.02445	0.998356
Dok7	231134	0.121135	0.998356
Sh3tc1	231147	0.711534	0.998356
Tada2b	231151	0.204294	0.998356
Cc2d2a	231214	0.058699	0.998356
Tapt1	231225	0.532792	0.998356
Sel1l3	231238	0.179722	0.998356
Guf1	231279	-0.01092	0.998356
Lrrc66	231296	0.602297	0.998356
Ppat	231327	-0.2797	0.998356
Polr2b	231329	-0.0672	0.998356
Uba6	231380	0.124601	0.998356
Ythdc1	231386	0.125214	0.998356
Grsf1	231413	0.161861	0.998356
Cox18	231430	-0.48795	0.998356
Sdad1	231452	-0.26343	0.998356
Cnot6l	231464	-0.15477	0.998356
Fras1	231470	-0.38019	0.998356

Tmem150c	231503	-0.40899	0.998356
Lin54	231506	-0.24834	0.998356
Lrrc8d	231549	-0.12591	0.998356
A830010M20Rik	231570	0.110041	0.998356
Rpap2	231571	0.169909	0.998356
Gak	231580	0.175639	0.998356
Chfr	231600	0.102707	0.998356
Ficd	231630	-0.17711	0.998356
Tmem119	231633	0.663794	0.998356
Ssh1	231637	0.158842	0.998356
Alkbh2	231642	0.002249	0.998356
Gcn1l1	231659	0.129362	0.998356
Vsig10	231668	0.066067	0.998356
Fbxo21	231670	-0.4035	0.998356
Fbxw8	231672	0.062265	0.998356
Trafd1	231712	-0.21624	0.998356
Naa25	231713	0.074317	0.998356
Fam109a	231717	0.415609	0.998356
Sfswap	231769	0.322124	0.998356
Lrch4	231798	-0.52095	0.998356
Mepce	231803	0.082258	0.998356
Pilra	231805	0.02238	0.998356
BC037034	231807	-0.32067	0.998356
Adap1	231821	0.757807	0.998356
Micall2	231830	0.052166	0.998356
Tmem184a	231832	0.020139	0.998356
Snx8	231834	0.085071	0.998356
Baat1	231841	-0.21942	0.998356
C330006K01Rik	231855	-0.24897	0.998356
Tnrc18	231861	-0.20236	0.998356
Fbxl18	231863	-0.06668	0.998356
Zfp12	231866	0.008519	0.998356
E130309D02Rik	231868	-0.34703	0.998356
Daglb	231871	0.322896	0.998356
Aimp2	231872	-0.12999	0.998356
Ccz1	231874	0.502376	0.998356
Lmtk2	231876	0.10573	0.998356
Bud31	231889	0.127505	0.998356
Uspl1	231915	-0.4431	0.998356
Gimap6	231931	0.538566	0.998356
Plekha8	231999	-10	0.998356
Vopp1	232023	0.113655	0.998356
Mat2a	232087	0.012919	0.998356
Elmod3	232089	0.704414	0.998356
Fam176a	232146	0.15809	0.998356
Mob1a	232157	0.002461	0.998356
Paip2b	232164	0.177952	0.998356

Smyd5	232187	-0.22362	0.998356
C87436	232196	-0.06464	0.998356
8430410A17Rik	232210	-0.55148	0.998356
Txnrd3	232223	0.449039	0.998356
Iqsec1	232227	0.152678	0.998356
Hdac11	232232	-0.31149	0.998356
C130022K22Rik	232236	-0.02739	0.998356
Tmf1	232286	0.007939	0.998356
Frmd4b	232288	0.213041	0.998356
Ppp4r2	232314	0.094273	0.998356
Vgll4	232334	-0.27591	0.998356
Zfp637	232337	-0.17078	0.998356
Ankrd26	232339	-0.06245	0.998356
Wnk1	232341	0.235546	0.998356
Clec2e	232409	0.327266	0.998356
H2afj	232440	-0.13664	0.998356
Dera	232449	-0.30733	0.998356
Pyroxd1	232491	0.24769	0.998356
Stk38l	232533	-0.33691	0.998356
Mrps35	232536	-0.05715	0.998356
Klhdc5	232539	-0.07026	0.998356
Amn1	232566	-0.23188	0.998356
Zc3hc1	232679	-0.3374	0.998356
Mgam	232714	0.149596	0.998356
Fam115c	232748	-0.1145	0.998356
Zfp212	232784	10	0.998356
Cnot3	232791	-0.05813	0.998356
Leng8	232798	0.145848	0.998356
Ppp1r12c	232807	0.026651	0.998356
Suv420h2	232811	1.69412	0.998356
Zfp628	232816	0.074299	0.998356
Zfp954	232853	0.229361	0.998356
Zfp418	232854	-0.05225	0.998356
Zfp772	232855	0.187616	0.998356
Zscan22	232878	-0.07038	0.998356
Zbtb45	232879	0.131262	0.998356
Grlf1	232906	0.241808	0.998356
Ap2s1	232910	0.270725	0.998356
Ccdc61	232933	0.235648	0.998356
Klc3	232943	0.645786	0.998356
Mark4	232944	0.137896	0.998356
Bloc1s3	232946	0.22101	0.998356
Ppp1r37	232947	0.168792	0.998356
Zfp428	232969	-0.17487	0.998356
Phldb3	232970	-0.0662	0.998356
Zfp574	232976	-0.28368	0.998356
B9d2	232987	0.424276	0.998356

Hnrnpul1	232989	-0.02213	0.998356
Itpkc	233011	0.255592	0.998356
Blvrb	233016	-0.14137	0.998356
Samd4b	233033	0.080072	0.998356
Zfp790	233056	-0.33058	0.998356
Alkbh6	233065	-0.30009	0.998356
AI428936	233066	-0.10066	0.998356
U2af1l4	233073	0.298787	0.998356
Ffar2	233079	-1.05689	0.998356
4931406P16Rik	233103	0.008243	0.998356
AI987944	233168	0.10133	0.998356
Ctu1	233189	-0.43526	0.998356
Tbc1d17	233204	-0.1894	0.998356
Scaf1	233208	-0.05801	0.998356
Prr12	233210	-0.26099	0.998356
Tubgcp5	233276	-0.01749	0.998356
Mtmr10	233315	0.04743	0.998356
Lrrk1	233328	0.31363	0.998356
Adamts17	233332	0.467264	0.998356
Vps33b	233405	0.024996	0.998356
Prc1	233406	-0.31486	0.998356
Zfp592	233410	-0.09221	0.998356
Picalm	233489	0.344135	0.998356
Crebzf	233490	-0.13924	0.998356
Rsf1	233532	-0.14838	0.998356
2210018M11Rik	233545	-0.02422	0.998356
Mogat2	233549	0.374752	0.998356
Gdpd5	233552	-0.30875	0.998356
P2ry6	233571	-0.38168	0.998356
Pgap2	233575	0.062389	0.998356
Tmem41b	233724	0.372395	0.998356
Ipo7	233726	-0.17644	0.998356
Galntl4	233733	-0.72439	0.998356
Spon1	233744	0.611494	0.998356
Plekha7	233765	-0.0943	0.998356
Smg1	233789	0.084751	0.998356
Thumpd1	233802	-0.07914	0.998356
Dcun1d3	233805	-0.15135	0.998356
Tmem159	233806	-0.08037	0.998356
BC030336	233812	0.169768	0.998356
Cog7	233824	0.243858	0.998356
Palb2	233826	-0.24178	0.998356
Tnrc6a	233833	-0.09924	0.998356
Gtf3c1	233863	0.11815	0.998356
D430042O09Rik	233865	-0.17113	0.998356
Tufm	233870	0.049411	0.998356
Atxn2l	233871	-0.13849	0.998356

Ino80e	233875	-0.3424	0.998356
Hirip3	233876	-0.23073	0.998356
Kctd13	233877	-0.07027	0.998356
Zfp553	233887	-0.03744	0.998356
Zfp768	233890	-0.11785	0.998356
Prr14	233895	-0.0865	0.998356
Gm166	233899	-0.02531	0.998356
Rnf40	233900	0.274614	0.998356
Fbxl19	233902	-0.45891	0.998356
Setd1a	233904	-0.12918	0.998356
Zfp646	233905	-0.04603	0.998356
Fus	233908	-0.45034	0.998356
Armc5	233912	0.112757	0.998356
BC017158	233913	0.309747	0.998356
Ppfia1	233977	-0.01309	0.998356
Tpcn2	233979	-0.18354	0.998356
Zfp958	233987	0.267502	0.998356
Arglu1	234023	-0.15691	0.998356
Pcid2	234069	-0.05708	0.998356
Tmco3	234076	0.072626	0.998356
Erich1	234086	0.223786	0.998356
Whsc1l1	234135	-0.08106	0.998356
Tti2	234138	-0.20625	0.998356
Sorbs2	234214	0.009217	0.998356
Neil3	234258	0.006201	0.998356
Cbr4	234309	0.023513	0.998356
Naf1	234344	-0.37716	0.998356
Psd3	234353	0.023123	0.998356
Zfp930	234358	-0.02122	0.998356
Zfp868	234362	0.510607	0.998356
Gatad2a	234366	-0.14143	0.998356
Tmem161a	234371	0.030196	0.998356
Sugp2	234373	-0.61871	0.998356
Ddx49	234374	-0.04564	0.998356
Klhl26	234378	1.849	0.998356
Mpv17l2	234384	0.484015	0.998356
Ccdc124	234388	-0.04467	0.998356
Ankle1	234396	0.366515	0.998356
Glt25d1	234407	-0.04681	0.998356
Zfp961	234413	-0.13539	0.998356
Tmem184c	234463	0.450388	0.998356
Inpp4b	234515	0.571042	0.998356
Heatr3	234549	-0.51165	0.998356
Cpne2	234577	-0.14621	0.998356
Ndrg4	234593	-0.06164	0.998356
Cnot1	234594	-1.14482	0.998356
Slc38a7	234595	-0.04576	0.998356

Dync1li2	234663	-0.03633	0.998356
Nae1	234664	0.024855	0.998356
Ces2c	234671	-0.96281	0.998356
Ces2e	234673	-0.35184	0.998356
D230025D16Rik	234678	0.45032	0.998356
Elmo3	234683	0.047639	0.998356
Edc4	234699	-0.14082	0.998356
Txnl4b	234723	-0.18092	0.998356
Tat	234724	0.702205	0.998356
Zfp612	234725	-0.21482	0.998356
Ftsjd1	234728	-0.09166	0.998356
Vac14	234729	-0.14808	0.998356
Fuk	234730	0.074117	0.998356
Ddx19b	234733	-0.00923	0.998356
Aars	234734	0.066388	0.998356
Atmin	234776	-0.13317	0.998356
Plcg2	234779	0.10461	0.998356
Klhl36	234796	-0.00397	0.998356
6430548M08Rik	234797	0.333344	0.998356
Mthfsd	234814	0.102783	0.998356
Klhdc4	234825	0.019225	0.998356
Il17c	234836	-0.86004	0.998356
Fam38a	234839	-0.02667	0.998356
Spg7	234847	0.464719	0.998356
Chmp1a	234852	0.327805	0.998356
Cdk10	234854	-10	0.998356
Spire2	234857	-0.01085	0.998356
Nup133	234865	0.285016	0.998356
Ttc13	234875	0.3701	0.998356
Med17	234959	0.043921	0.998356
Slc36a4	234967	0.238743	0.998356
Zfp426	235028	0.033507	0.998356
Ppan	235036	-0.44968	0.998356
Atg4d	235040	0.018329	0.998356
Kank2	235041	0.140923	0.998356
Tmem205	235043	-0.40046	0.998356
Zfp809	235047	-0.13486	0.998356
Zfp599	235048	0.044332	0.998356
Zfp810	235050	0.272441	0.998356
Igsf9b	235086	-1.24314	0.998356
Zbtb44	235132	0.270922	0.998356
Nfrkb	235134	0.030044	0.998356
Tmem45b	235135	0.471318	0.998356
Foxred1	235169	0.076052	0.998356
Gramd1b	235283	0.07152	0.998356
Sc5d	235293	0.085631	0.998356
Rnf214	235315	-0.34281	0.998356

Usp28	235323	-0.10504	0.998356
Dlat	235339	-0.18958	0.998356
Sik2	235344	-0.18223	0.998356
Snx33	235406	-0.10579	0.998356
Herc1	235439	0.071631	0.998356
Usp3	235441	0.202511	0.998356
Rab8b	235442	-0.03811	0.998356
Gtf2a2	235459	-0.16556	0.998356
Fam63b	235461	0.191028	0.998356
Zfp280d	235469	-0.26752	0.998356
Leo1	235497	-0.46289	0.998356
Slc17a5	235504	0.342451	0.998356
Gk5	235533	-0.14479	0.998356
Acpl2	235534	-0.01671	0.998356
Ppp2r3a	235542	0.311853	0.998356
Topbp1	235559	-0.23289	0.998356
Dnajc13	235567	0.155679	0.998356
Atp2c1	235574	1.61816	0.998356
Dusp7	235584	-0.46868	0.998356
Parp3	235587	0.463801	0.998356
Apeh	235606	0.067794	0.998356
Atrip	235610	0.23012	0.998356
Plxnb1	235611	-0.05525	0.998356
Scap	235623	0.384965	0.998356
Setd2	235626	0.192563	0.998356
Nbeal2	235627	0.018314	0.998356
Als2cl	235633	1.79558	0.998356
Dync1li1	235661	-0.1851	0.998356
Zfp445	235682	-0.08516	0.998356
Zfp71-rs1	235907	0.056791	0.998356
Zfp825	235956	0.089672	0.998356
Zfp709	236193	-0.84312	0.998356
Alms1	236266	-0.16384	0.998356
Eif2c1	236511	-0.40677	0.998356
Phgdh	236539	-0.43466	0.998356
Gbp9	236573	0.328617	0.998356
Pisd-ps1	236604	0.232879	0.998356
Rbm10	236732	-0.4963	0.998356
Usp11	236733	-0.23143	0.998356
Ddx26b	236790	0.309783	0.998356
Mmgt1	236792	0.094248	0.998356
Slc9a6	236794	0.089316	0.998356
Pdk3	236900	0.237255	0.998356
Ercc6l	236930	-0.40099	0.998356
Rbm41	237073	0.032674	0.998356
Nxt2	237082	-0.24483	0.998356
Gnl3l	237107	-0.20345	0.998356

Ofd1	237222	0.021745	0.998356
Zc3h12d	237256	0.174771	0.998356
Tbpl1	237336	-0.32867	0.998356
L3mbtl3	237339	0.191421	0.998356
Mex3d	237400	0.309853	0.998356
Ric8b	237422	-0.30931	0.998356
Gas2l3	237436	0.348518	0.998356
Cdk17	237459	-0.14984	0.998356
Tmtc3	237500	-0.93464	0.998356
Rassf9	237504	-0.24026	0.998356
Osbpl8	237542	0.252147	0.998356
Ankrd52	237615	0.023096	0.998356
Smcr7	237781	0.340129	0.998356
Smcr8	237782	0.018061	0.998356
Pfas	237823	-0.0498	0.998356
Rtn4rl1	237847	0.136225	0.998356
Ccdc55	237859	-0.101	0.998356
Ssh2	237860	0.153391	0.998356
Atad5	237877	-0.17372	0.998356
Slfn9	237886	-0.40829	0.998356
Usp32	237898	0.202893	0.998356
Brip1	237911	-0.23073	0.998356
Rsad1	237926	-0.33102	0.998356
Gpatch8	237943	-0.01475	0.998356
Cdr2l	237988	-0.25185	0.998356
Hexdc	238023	0.532934	0.998356
Fn3krp	238024	-0.04252	0.998356
Cog5	238123	0.039426	0.998356
Dock4	238130	-0.13762	0.998356
Arid4a	238247	-0.13304	0.998356
Tmem30b	238257	0.30084	0.998356
Syt16	238266	0.275618	0.998356
C130039O16Rik	238317	-0.09202	0.998356
Rps6kl1	238323	0.427742	0.998356
6430527G18Rik	238330	-0.06149	0.998356
Btbd7	238386	-0.02074	0.998356
Macc1	238455	-0.19941	0.998356
Mtr	238505	0.084318	0.998356
Zfp367	238673	-0.19487	0.998356
Zfp874a	238692	0.117063	0.998356
Zfp58	238693	-0.00913	0.998356
Tnpo1	238799	-0.95019	0.998356
Ppwd1	238831	-0.11373	0.998356
Pde4d	238871	0.285847	0.998356
Cdh24	239096	-0.23744	0.998356
Homez	239099	0.100386	0.998356
Zfhx2	239102	-0.30564	0.998356

Setdb2	239122	0.067399	0.998356
Fam160b2	239170	-0.00332	0.998356
Kctd12	239217	0.076603	0.998356
Abcc4	239273	0.37683	0.998356
Card6	239319	-0.24143	0.998356
Lrp12	239393	0.320518	0.998356
Phf20l1	239510	0.082695	0.998356
Eif2c2	239528	-0.10109	0.998356
Gpr20	239530	-0.06834	0.998356
Zfp647	239546	-0.26745	0.998356
Smcr7l	239555	-0.00326	0.998356
Ttc38	239570	0.169215	0.998356
Slc2a13	239606	0.12679	0.998356
Dip2b	239667	0.128512	0.998356
Mettl22	239706	-0.33152	0.998356
Mkl2	239719	-0.13989	0.998356
Liph	239759	0.522412	0.998356
1600021P15Rik	239796	0.535208	0.998356
Ccdc14	239839	-0.12482	0.998356
Gpr128	239853	0.539003	0.998356
Arid1b	239985	0.013522	0.998356
Dact2	240025	-0.09934	0.998356
Lnpep	240028	0.107649	0.998356
Zfp760	240034	0.043593	0.998356
Gm4944	240038	0.303655	0.998356
Zfp799	240064	0.074105	0.998356
Zfp952	240067	-0.01924	0.998356
Cchcr1	240084	-0.38511	0.998356
Mdc1	240087	-0.35089	0.998356
Zfp119b	240120	0.086595	0.998356
Thada	240174	0.006303	0.998356
Ythdc2	240255	0.041067	0.998356
Fem1c	240263	-0.1676	0.998356
Dmxl1	240283	0.148437	0.998356
Gm4951	240327	-1.27616	0.998356
Pcyox1l	240334	-0.38146	0.998356
Malt1	240354	0.181979	0.998356
Mex3c	240396	0.246214	0.998356
Adnp2	240442	-0.27885	0.998356
Zfp407	240476	0.136587	0.998356
Cdc42bpg	240505	0.095536	0.998356
Ccdc85b	240514	0.417888	0.998356
9930021J03Rik	240613	0.026083	0.998356
Ranbp6	240614	0.139133	0.998356
Slc16a12	240638	-0.56425	0.998356
Kif20b	240641	-0.12394	0.998356
Tmem20	240660	0.086154	0.998356

Ccnj	240665	0.0435	0.998356
Vwa2	240675	0.828501	0.998356
Sulf1	240725	-0.29962	0.998356
Slco5a1	240726	-0.36404	0.998356
Pik3c2b	240752	-0.01639	0.998356
Plekha6	240753	0.410956	0.998356
Klhl12	240756	-0.06347	0.998356
Tor1aip2	240832	0.045836	0.998356
Zbtb37	240869	0.1474	0.998356
Scyl3	240880	0.136893	0.998356
Adamts4	240913	-0.2866	0.998356
Pgap1	241062	0.517316	0.998356
Noxa1	241275	0.269407	0.998356
Lrrc8a	241296	0.084345	0.998356
Ralgps1	241308	-0.04289	0.998356
Zbtb34	241311	-0.30888	0.998356
Zbtb6	241322	-0.16859	0.998356
Olfml2a	241327	0.267942	0.998356
Galnt5	241391	0.115685	0.998356
Lass6	241447	-0.1914	0.998356
Rbm45	241490	-0.27162	0.998356
Harbi1	241547	0.102804	0.998356
Ldlrad3	241576	-0.08113	0.998356
Wdr76	241627	0.001743	0.998356
Prosapip1	241638	-0.18845	0.998356
Fermt1	241639	-0.33579	0.998356
Ralgapa2	241694	0.052652	0.998356
Lsm14b	241846	-0.33222	0.998356
Phc3	241915	-1.11768	0.998356
D3Ertd254e	241944	-0.21802	0.998356
Frem2	242022	0.254231	0.998356
Ppm1l	242083	0.12546	0.998356
Pde5a	242202	0.255709	0.998356
Slc44a5	242259	0.630408	0.998356
Impad1	242291	0.106634	0.998356
Manea	242362	0.054412	0.998356
Rgp1	242406	-0.01504	0.998356
Dcaf10	242418	0.162822	0.998356
D730040F13Rik	242474	0.360156	0.998356
D630039A03Rik	242484	-0.11391	0.998356
Rasef	242505	0.047174	0.998356
Klhl9	242521	0.053736	0.998356
Atg4c	242557	0.0322	0.998356
Slc35d1	242585	0.38485	0.998356
Podn	242608	0.353844	0.998356
Wasf2	242687	-0.14347	0.998356
Gpatch3	242691	0.568039	0.998356

Il28ra	242700	0.108365	0.998356
E2f2	242705	-0.09338	0.998356
Pramef8	242736	-0.25789	0.998356
Zfp933	242747	-0.27513	0.998356
Klhl21	242785	-0.14113	0.998356
Rsbn1l	242860	0.090799	0.998356
Napepld	242864	-0.16628	0.998356
Fbxl5	242960	0.298058	0.998356
Ugt2b35	243085	-0.35493	0.998356
2900026A02Rik	243219	-0.10158	0.998356
Sbno1	243272	0.030017	0.998356
A430033K04Rik	243308	-0.41683	0.998356
Stard13	243362	-0.30501	0.998356
AI854703	243373	-0.54658	0.998356
Ppm1k	243382	-0.32829	0.998356
Gprin3	243385	0.021287	0.998356
H1fx	243529	0.468817	0.998356
Styk1	243659	0.164509	0.998356
Ppp1r9a	243725	-0.11226	0.998356
2010107G12Rik	243753	0.021885	0.998356
E330009J07Rik	243780	-0.19526	0.998356
Leng9	243813	0.628395	0.998356
Ppp6r1	243819	0.096584	0.998356
Zfp324	243834	0.030208	0.998356
Gltscr1	243842	-0.30991	0.998356
Ccdc9	243846	0.129065	0.998356
Fkrp	243853	-0.05607	0.998356
Fbxo46	243867	-0.01645	0.998356
Nlrp9b	243874	0.44779	0.998356
Zfp568	243905	-0.53127	0.998356
Nfkbid	243910	-0.32683	0.998356
Zdhhc13	243983	0.431833	0.998356
Mctp2	244049	-0.25543	0.998356
Chd2	244059	-0.19903	0.998356
Nars2	244141	-0.13112	0.998356
Tsku	244152	-0.33369	0.998356
Nlrp10	244202	0.123897	0.998356
Zfp771	244216	0.268685	0.998356
Zfp668	244219	-0.15622	0.998356
Mcph1	244329	-0.4756	0.998356
Myst3	244349	-0.10162	0.998356
Erlin2	244373	0.129172	0.998356
Ppp1r3b	244416	0.094163	0.998356
D8Ertd82e	244418	0.232647	0.998356
Elmod2	244548	0.662842	0.998356
Tox3	244579	0.138633	0.998356
Rpgrip1l	244585	0.139206	0.998356

Pskh1	244631	0.01649	0.998356
Phlpp2	244650	0.212991	0.998356
Mtss1l	244654	0.599498	0.998356
Gm505	244666	0.146125	0.998356
Sipa1l2	244668	0.108735	0.998356
Cwf19l2	244672	-0.0746	0.998356
Zfp317	244713	-0.23847	0.998356
Zfp846	244721	-0.52419	0.998356
Fam55d	244853	-0.03093	0.998356
Zc3h12c	244871	-0.11197	0.998356
Npat	244879	0.112635	0.998356
Scaper	244891	-0.33534	0.998356
C230081A13Rik	244895	-0.02039	0.998356
Snx14	244962	0.423693	0.998356
Atr	245000	0.132015	0.998356
Zbtb38	245007	-0.11603	0.998356
Retnlg	245195	-0.96533	0.998356
Dkc1	245474	-0.03674	0.998356
Fam199x	245622	-0.02686	0.998356
Tbc1d8b	245638	0.125758	0.998356
Rbbp7	245688	-0.42553	0.998356
Tceanc	245695	0.032261	0.998356
Fat2	245827	-0.06146	0.998356
Trappc1	245828	0.083279	0.998356
Polr2h	245841	0.351266	0.998356
Amdhd2	245847	0.026574	0.998356
Ssh3	245857	-0.06973	0.998356
Atg9a	245860	0.278411	0.998356
Ift52	245866	0.154561	0.998356
Pcmtd2	245867	0.052682	0.998356
Mtap7d1	245877	-0.57747	0.998356
Ankrd27	245886	-0.13587	0.998356
Vps54	245944	0.430073	0.998356
Rbm47	245945	0.088425	0.998356
Chodl	246048	-0.10799	0.998356
Atxn7	246103	0.253732	0.998356
Vasn	246154	0.312068	0.998356
Fktn	246179	-0.18429	0.998356
Zfp277	246196	0.499055	0.998356
Mllt6	246198	0.122896	0.998356
Mpst	246221	-0.15044	0.998356
Vwa1	246228	0.196052	0.998356
Bivm	246229	-0.15701	0.998356
Fcgr4	246256	0.487977	0.998356
Ovca2	246257	-0.11673	0.998356
Csad	246277	0.101413	0.998356
Klhl8	246293	0.283663	0.998356

Hps5	246694	-0.80992	0.998356
Apoa1bp	246703	0.1701	0.998356
Emilin2	246707	-0.1793	0.998356
Rhobtb2	246710	0.08622	0.998356
Oas2	246728	0.982518	0.998356
Cd300lf	246746	1.51551	0.998356
Atpaf2	246782	0.019202	0.998356
Tox	252838	0.158099	0.998356
Usp7	252870	0.106098	0.998356
Mios	252875	-0.04587	0.998356
Gin1	252876	0.148861	0.998356
Ap1s3	252903	0.573812	0.998356
Cables2	252966	0.139642	0.998356
Tpcn1	252972	0.115906	0.998356
Grhl2	252973	-0.14493	0.998356
Acsf3	257633	-0.0582	0.998356
Sdsl	257635	-0.53472	0.998356
Olfr1372-ps1	257871	1.31815	0.998356
Olfr884	257996	1.18899	0.998356
Tubgcp3	259279	0.041124	0.998356
Ehd2	259300	0.175638	0.998356
Cadm4	260299	-0.2088	0.998356
Gga3	260302	-0.42214	0.998356
Cdc42ep3	260409	0.366341	0.998356
Hist1h3f	260423	-0.5241	0.998356
Plekhg3	263406	0.072185	0.998356
Pkn3	263803	-0.06274	0.998356
Spata2	263876	-0.01972	0.998356
Cdk8	264064	-0.01143	0.998356
Acsf2	264895	-0.03281	0.998356
Defb36	266620	-3.1585	0.998356
Irak4	266632	0.08218	0.998356
Cyb5r4	266690	-0.04046	0.998356
Cpne1	266692	-0.49492	0.998356
Snx17	266781	0.273032	0.998356
Rps15a	267019	0.031493	0.998356
Shprh	268281	-0.15362	0.998356
Rnf217	268291	0.027613	0.998356
Zbtb24	268294	-0.15076	0.998356
Scml4	268297	-0.06945	0.998356
Sowahc	268301	0.114183	0.998356
Ppia	268373	-0.12113	0.998356
Ahsa2	268390	-0.1589	0.998356
Mpg	268395	0.092006	0.998356
Sh3pxd2b	268396	-0.42604	0.998356
Zkscan17	268417	-0.074	0.998356
Alkbh5	268420	0.030331	0.998356

Ankrd13b	268445	-0.06934	0.998356
Phf12	268448	0.082851	0.998356
Rpl23a	268449	0.45874	0.998356
Rab11fip4	268451	0.085711	0.998356
Eme1	268465	-0.91417	0.998356
Zfp652	268469	0.065285	0.998356
Ube2z	268470	0.184383	0.998356
Rapgefl1	268480	0.119384	0.998356
Lsm12	268490	-0.24076	0.998356
Bahcc1	268515	-0.16769	0.998356
Zbtb1	268564	-0.19335	0.998356
Gphn	268566	0.086297	0.998356
Tmem229b	268567	-0.85844	0.998356
Sptlc1	268656	0.450298	0.998356
Ccnb1	268697	-0.07691	0.998356
2310021P13Rik	268721	-0.6942	0.998356
Arhgef40	268739	0.02157	0.998356
Tox4	268741	0.022226	0.998356
Rnf31	268749	0.1467	0.998356
Wdfy2	268752	-0.4201	0.998356
Mtmr12	268783	-0.18328	0.998356
Adck5	268822	0.131483	0.998356
Abat	268860	-0.04321	0.998356
AI480653	268880	0.250487	0.998356
Fbxo45	268882	0.156352	0.998356
Stfa2l1	268885	0.529828	0.998356
Robo2	268902	0.711727	0.998356
Nrip1	268903	-0.10024	0.998356
Pkmyt1	268930	0.514539	0.998356
Wdr24	268933	0.01481	0.998356
Brpf3	268936	0.115748	0.998356
Nlrc4	268973	0.312088	0.998356
Strn	268980	0.020311	0.998356
Ss18	268996	-0.09207	0.998356
Sh3rf2	269016	-0.15992	0.998356
Zfp608	269023	0.201763	0.998356
4930503L19Rik	269033	-0.64596	0.998356
Ctif	269037	-0.26583	0.998356
Cpsf7	269061	0.035286	0.998356
Nup54	269113	0.088455	0.998356
Kif26b	269152	0.233242	0.998356
Inpp4a	269180	0.263206	0.998356
Mgat4a	269181	0.239177	0.998356
Nbeal1	269198	-0.05942	0.998356
Pask	269224	-0.38639	0.998356
Fam171a1	269233	-0.02771	0.998356
Gtf3c4	269252	-0.05743	0.998356

Setx	269254	0.070888	0.998356
Rpl12	269261	-0.02117	0.998356
Ccdc32	269336	0.296568	0.998356
Vps39	269338	0.002159	0.998356
Ell3	269344	-0.12941	0.998356
Slc4a11	269356	0.410458	0.998356
Ahcy	269378	0.489969	0.998356
Znf512b	269401	0.287268	0.998356
Plch1	269437	-0.2568	0.998356
Wdr3	269470	0.012767	0.998356
Lrig2	269473	0.071997	0.998356
Fbxl4	269514	0.492333	0.998356
Vcp	269523	-0.20691	0.998356
Fbxo10	269529	0.376925	0.998356
Tex10	269536	-0.16163	0.998356
Clspn	269582	-0.26706	0.998356
Epb4.1	269587	-0.05745	0.998356
Sytl1	269589	-0.11176	0.998356
Luzp1	269593	-0.07413	0.998356
Plekhg5	269608	0.02409	0.998356
Pank4	269614	0.359487	0.998356
Plch2	269615	-1.20366	0.998356
C030048B08Rik	269623	0.421515	0.998356
5031425E22Rik	269630	0.147245	0.998356
Zfp512	269639	0.035729	0.998356
Golga3	269682	0.04972	0.998356
Gm15800	269700	0.011594	0.998356
Mphosph9	269702	-0.022	0.998356
Zfp664	269704	-0.09891	0.998356
Clip2	269713	-0.08779	0.998356
Orai2	269717	-0.10664	0.998356
Aak1	269774	0.097733	0.998356
Zfp384	269800	0.287357	0.998356
Pon3	269823	-0.11204	0.998356
Tspan12	269831	-0.08336	0.998356
Megf8	269878	-0.07724	0.998356
Chsy1	269941	-0.16463	0.998356
Idh2	269951	-0.10355	0.998356
D330012F22Rik	269952	-0.25413	0.998356
Nup98	269966	-0.36559	0.998356
Zfp747	269997	0.305365	0.998356
Orai3	269999	0.316901	0.998356
Mtap1s	270058	-0.14227	0.998356
Slc35e1	270066	0.169366	0.998356
Gcdh	270076	-0.01711	0.998356
Lpcat2	270084	-0.067	0.998356
Ogfod1	270086	-0.26117	0.998356

Rpl13	270106	-0.2869	0.998356
Irf2bp2	270110	-0.13195	0.998356
Nlrx1	270151	0.778123	0.998356
Amica1	270152	-0.18839	0.998356
AU019823	270156	0.058924	0.998356
Myo9a	270163	0.092538	0.998356
Clpx	270166	-0.92698	0.998356
Pfkfb4	270198	-0.1488	0.998356
Klhl18	270201	0.057882	0.998356
Taf1	270627	-0.31275	0.998356
Mbtps2	270669	0.161108	0.998356
Map3k15	270672	0.159418	0.998356
Mthfd1l	270685	-0.30964	0.998356
BC048403	270802	0.157706	0.998356
Prr11	270906	-0.31218	0.998356
Ankdd1b	271144	0.494159	0.998356
Phf21b	271305	0.259479	0.998356
Cd200r2	271375	0.563109	0.998356
Zbtb11	271377	-0.2956	0.998356
Rab5a	271457	0.196236	0.998356
Vps13a	271564	-0.19789	0.998356
Rpusd2	271842	-0.15129	0.998356
Tbck	271981	-0.07939	0.998356
Tstd2	272027	0.034054	0.998356
Arntl2	272322	-0.40918	0.998356
Zfp398	272347	-0.37184	0.998356
Irf2bp1	272359	-0.05257	0.998356
Gins2	272551	-0.16303	0.998356
Tbcel	272589	0.289418	0.998356
Eif5a	276770	-0.51064	0.998356
Pigs	276846	-0.26746	0.998356
D11Wsu47e	276852	0.082261	0.998356
Armc7	276905	0.4094	0.998356
Gemin4	276919	0.00246	0.998356
Marveld1	277010	-0.09109	0.998356
Kdm3b	277250	0.046088	0.998356
Prex1	277360	0.280867	0.998356
Trp53i11	277414	-0.10421	0.998356
Gpr107	277463	0.238465	0.998356
Depdc5	277854	0.269579	0.998356
C2cd3	277939	-0.25752	0.998356
Tmtc2	278279	0.08561	0.998356
Rilp	280408	-0.23477	0.998356
Flnb	286940	0.313888	0.998356
Klk15	317652	0.873221	0.998356
Sec22a	317717	0.606056	0.998356
Gimap9	317758	-0.07541	0.998356

Hist1h3c	319148	-0.44091	0.998356
Hist1h3d	319149	0.073707	0.998356
Hist1h3e	319151	-0.0048	0.998356
Hist1h3h	319152	0.237672	0.998356
Hist1h3i	319153	-0.43904	0.998356
Hist1h4c	319155	-0.32889	0.998356
Hist1h4d	319156	-0.27375	0.998356
Hist1h4f	319157	-0.70351	0.998356
Hist1h4i	319158	0.649608	0.998356
Hist1h4j	319159	0.11978	0.998356
Hist1h4k	319160	-0.10362	0.998356
Hist1h4n	319161	-0.62974	0.998356
Hist3h2a	319162	0.264885	0.998356
Hist1h2ac	319164	0.0814	0.998356
Hist1h2ad	319165	0.162818	0.998356
Hist1h2ae	319166	-0.50991	0.998356
Hist1h2ag	319167	-0.34485	0.998356
Hist1h2ak	319169	-0.29421	0.998356
Hist1h2ap	319171	-4.39826	0.998356
Hist1h2af	319173	0.45574	0.998356
Hist2h2ac	319176	-0.24131	0.998356
Hist1h2ba	319177	-1.87546	0.998356
Hist1h2bb	319178	0.303799	0.998356
Hist1h2be	319179	-0.06249	0.998356
Hist1h2bf	319180	0.341468	0.998356
Hist1h2bg	319181	0.423287	0.998356
Hist1h2bh	319182	0.314827	0.998356
Hist1h2bj	319183	0.469658	0.998356
Hist1h2bl	319185	-0.34774	0.998356
Hist1h2bp	319188	0.234875	0.998356
Hist2h2bb	319189	-0.08837	0.998356
Hist2h2be	319190	-0.10276	0.998356
Hist1h2ai	319191	-0.36582	0.998356
Hist2h2aa2	319192	-2.82025	0.998356
Rpl17	319195	-0.07165	0.998356
Trim12c	319236	0.676126	0.998356
Pcmtd1	319263	0.025112	0.998356
A130010J15Rik	319266	0.850044	0.998356
A230046K03Rik	319277	-0.0761	0.998356
A230050P20Rik	319278	-0.46585	0.998356
Sf3b2	319322	-0.3181	0.998356
Fam100b	319370	0.516653	0.998356
D030028A08Rik	319371	-0.54969	0.998356
Fndc3a	319448	0.218305	0.998356
A430071A18Rik	319454	-0.62627	0.998356
Ppm1h	319468	-0.20463	0.998356
Zfp672	319475	0.10769	0.998356

Wdr59	319481	-0.25007	0.998356
Pdpr	319518	0.172088	0.998356
Zfp750	319530	0.178999	0.998356
Zfp182	319535	-0.01124	0.998356
D430020J02Rik	319545	-0.3289	0.998356
Idi1	319554	-0.15125	0.998356
Syne2	319565	0.1454	0.998356
6430573F11Rik	319582	0.38325	0.998356
Lig4	319583	-0.08605	0.998356
4930539J05Rik	319587	-1.16485	0.998356
Hif1an	319594	0.094972	0.998356
Fam168a	319604	-0.28559	0.998356
Zfp944	319615	0.100717	0.998356
Dcp1b	319618	-0.0886	0.998356
Itpripl2	319622	0.292698	0.998356
Galm	319625	-0.16916	0.998356
Nt5dc1	319638	0.422807	0.998356
Usp37	319651	-0.02185	0.998356
5830418K08Rik	319675	-0.16609	0.998356
Frmd6	319710	0.249893	0.998356
4732471D19Rik	319719	-0.10689	0.998356
Zfyve27	319740	0.335006	0.998356
Mpzl3	319742	0.444122	0.998356
Zfp865	319748	0.144151	0.998356
Smo	319757	-0.15434	0.998356
Rfx7	319758	-0.13338	0.998356
Igf2bp2	319765	-0.22045	0.998356
Atp10b	319767	0.215974	0.998356
9630033F20Rik	319801	-0.33237	0.998356
Rc3h2	319817	-0.20375	0.998356
1500004A13Rik	319830	0.234725	0.998356
Slc17a4	319848	0.021371	0.998356
E030011O05Rik	319859	-0.31234	0.998356
4732490B19Rik	319871	-0.13912	0.998356
Cobll1	319876	0.116738	0.998356
Tmcc3	319880	-0.24595	0.998356
Zcchc7	319885	-0.07927	0.998356
E030030I06Rik	319887	-1.34241	0.998356
Dock6	319899	0.34055	0.998356
Sbf2	319934	0.1286	0.998356
Tns3	319939	0.139515	0.998356
Taf2	319944	0.139845	0.998356
Flad1	319945	-0.14551	0.998356
Ttll1	319953	0.318785	0.998356
Ercc6	319955	-0.13742	0.998356
Cc2d1b	319965	0.301852	0.998356
Auts2	319974	0.064298	0.998356

Casc4	319996	0.75543	0.998356
Sidt1	320007	0.151369	0.998356
Uggt1	320011	-0.11956	0.998356
Nceh1	320024	-0.03527	0.998356
Fstl4	320027	-0.61204	0.998356
Exph5	320051	-0.4097	0.998356
Olfml2b	320078	0.26734	0.998356
Zbtb39	320080	-0.16118	0.998356
Prr18	320111	0.590944	0.998356
Rps6kc1	320119	0.193327	0.998356
Adrbk2	320129	-0.11795	0.998356
Zdhhc17	320150	0.109319	0.998356
Ccdc45	320162	-0.29775	0.998356
Msrb3	320183	0.05122	0.998356
Lrrc58	320184	-0.16881	0.998356
Hook3	320191	0.055824	0.998356
4833442J19Rik	320204	0.169649	0.998356
Ddx11	320209	-0.49134	0.998356
Senp5	320213	0.035263	0.998356
4930473A06Rik	320226	-0.53074	0.998356
Ccdc66	320234	-0.02967	0.998356
Ttll5	320244	-0.17811	0.998356
Fubp3	320267	-0.04784	0.998356
Rasgef1b	320292	0.131524	0.998356
Iqcb1	320299	0.310221	0.998356
Glt28d2	320302	0.097626	0.998356
D230037D09Rik	320351	-0.33582	0.998356
Fry	320365	0.109706	0.998356
Bcorl1	320376	-0.06197	0.998356
Cenpt	320394	0.900882	0.998356
Lrig3	320398	0.049145	0.998356
Itpkb	320404	-0.36682	0.998356
Cadps2	320405	0.092602	0.998356
Gchfr	320415	0.610486	0.998356
Alg6	320438	-0.21844	0.998356
9930014A18Rik	320469	-0.3546	0.998356
Heatr5b	320473	-0.09844	0.998356
Heatr5a	320487	0.069613	0.998356
Lmbrd2	320506	-0.0222	0.998356
Cachd1	320508	0.281502	0.998356
Vps13c	320528	0.154054	0.998356
Tmem104	320534	-0.41792	0.998356
Ubn2	320538	-0.15427	0.998356
Slc35e2	320541	-0.06243	0.998356
A630089N07Rik	320586	-0.18992	0.998356
Svopl	320590	0.429305	0.998356
Phf8	320595	-0.10718	0.998356

Dopey1	320615	0.111768	0.998356
9230105E05Rik	320626	-0.44774	0.998356
Snrnp200	320632	-0.11844	0.998356
Zbtb26	320633	-0.27384	0.998356
Ocrl	320634	0.37001	0.998356
A630066F11Rik	320642	0.660686	0.998356
Pgap3	320655	0.149771	0.998356
D5Ertd579e	320661	0.213622	0.998356
Samd12	320679	-0.32904	0.998356
Zfp629	320683	-0.13701	0.998356
Dctd	320685	-1.16742	0.998356
Mysm1	320713	-0.01377	0.998356
D030016E14Rik	320714	0.16372	0.998356
Pptc7	320717	0.443364	0.998356
Slc26a9	320718	-0.25669	0.998356
Ipo8	320727	0.049314	0.998356
Chd7	320790	-0.03587	0.998356
Pkn1	320795	0.68973	0.998356
Zhx3	320799	-0.00445	0.998356
Gfm2	320806	0.12982	0.998356
Dcaf5	320808	-0.02384	0.998356
Ankrd16	320816	0.189004	0.998356
Atad2b	320817	-0.01786	0.998356
Samd5	320825	0.311717	0.998356
C530008M17Rik	320827	0.021187	0.998356
Mical2	320878	0.324425	0.998356
Itgb8	320910	0.094517	0.998356
Tnpo3	320938	-0.3459	0.998356
Atp11c	320940	-1.91796	0.998356
Pisd	320951	0.103114	0.998356
D330023K18Rik	320973	1.31287	0.998356
A330023F24Rik	320977	0.163323	0.998356
Arl4c	320982	-0.17907	0.998356
4933421E11Rik	321000	0.057553	0.998356
Xpnpep3	321003	-0.06513	0.998356
Vprbp	321006	-0.18576	0.998356
Serac1	321007	-0.29413	0.998356
Cdv3	321022	0.005559	0.998356
Tpm4	326618	-0.47194	0.998356
Hist1h4a	326619	0.72404	0.998356
Hist1h4b	326620	0.430123	0.998356
Upf2	326622	0.010636	0.998356
Ppip5k1	327655	0.088576	0.998356
Dna2	327762	-0.42855	0.998356
Frs2	327826	0.218514	0.998356
Ubtd2	327900	0.025975	0.998356
Pigl	327942	-0.00071	0.998356

Cyb5d1	327951	-0.75029	0.998356
Xaf1	327959	0.483656	0.998356
Slfn5	327978	0.418715	0.998356
Med13	327987	0.247392	0.998356
6530401N04Rik	328092	-0.31283	0.998356
Prps1l3	328099	-0.22541	0.998356
Fam179b	328108	-0.132	0.998356
Prpf39	328110	-0.07911	0.998356
Slc39a9	328133	-0.04591	0.998356
Trmt61a	328162	-0.43039	0.998356
Slc25a48	328258	0.95604	0.998356
Gm9776	328309	-0.44949	0.998356
Mast4	328329	-0.10546	0.998356
Zmiz1	328365	0.039452	0.998356
Rft1	328370	0.087185	0.998356
Parp4	328417	0.166606	0.998356
Ep300	328572	0.055779	0.998356
Tubgcp6	328580	-0.20302	0.998356
Pisd-ps2	328734	0.464692	0.998356
Zfp414	328801	-1.14461	0.998356
Mcc	328949	-0.13262	0.998356
9330117O12	328957	0.275814	0.998356
Zfp532	328977	-0.29248	0.998356
Zfp236	329002	-0.08648	0.998356
Zfp516	329003	-0.18103	0.998356
Atg2a	329015	0.304064	0.998356
Lipo1	329055	-0.27679	0.998356
9130024F11Rik	329160	0.165181	0.998356
Abi2	329165	-0.13007	0.998356
Lgr6	329252	-0.63551	0.998356
Dennd1b	329260	0.407157	0.998356
Ptrh1	329384	0.115841	0.998356
Nostrin	329416	0.202171	0.998356
Lcmt2	329504	-0.15057	0.998356
Ctdspl2	329506	-0.16435	0.998356
8430427H17Rik	329540	0.068794	0.998356
Zfp335	329559	0.043694	0.998356
Gm14325	329575	0.238036	0.998356
E130311K13Rik	329659	-0.22269	0.998356
Fnip2	329679	-0.00656	0.998356
4933431E20Rik	329735	0.170442	0.998356
Fam102b	329739	0.443456	0.998356
Pigk	329777	-0.02061	0.998356
Tmem67	329795	0.440299	0.998356
AI464131	329828	0.383897	0.998356
Dennd4c	329877	0.328335	0.998356
Usp24	329908	-0.01743	0.998356

Acot11	329910	0.048825	0.998356
Fam185a	330050	0.038231	0.998356
Cxcl3	330122	-0.69149	0.998356
Kctd10	330171	-0.70801	0.998356
2610524H06Rik	330173	0.383119	0.998356
Taok3	330177	0.357238	0.998356
Tmem120b	330189	-0.26228	0.998356
Vps37b	330192	0.114643	0.998356
Pon2	330260	0.318265	0.998356
Wipf3	330319	1.26124	0.998356
Tmcc1	330401	-0.09402	0.998356
B4galnt3	330406	-0.11386	0.998356
Tmem150b	330460	-0.10651	0.998356
Zc3h4	330474	-0.07829	0.998356
C230091D08Rik	330544	-0.34515	0.998356
Fan1	330554	0.095048	0.998356
Dock1	330662	-0.29375	0.998356
B4galnt4	330671	-0.22285	0.998356
Ctxn1	330695	-0.63215	0.998356
Zfp866	330788	0.09214	0.998356
Rnf150	330812	0.048547	0.998356
Lphn1	330814	0.028968	0.998356
Dhps	330817	-0.58596	0.998356
Slc7a6	330836	-0.22297	0.998356
Arhgap32	330914	-0.07966	0.998356
Snapc5	330959	-0.08097	0.998356
Ostb	330962	0.658492	0.998356
Slc9a9	331004	0.408059	0.998356
Gmppb	331026	0.098037	0.998356
Tgm4	331046	0.562998	0.998356
Gsdmc2	331063	0.699513	0.998356
Thoc2	331401	-0.05193	0.998356
Uprt	331487	-0.18069	0.998356
Bend3	331623	-0.04575	0.998356
Mapk15	332110	0.055643	0.998356
Gpd1l	333433	-0.09023	0.998356
Mamld1	333639	0.530539	0.998356
Ppp1r13l	333654	-0.16809	0.998356
N4bp2	333789	0.008606	0.998356
Mia2	338320	0.240218	0.998356
Cog3	338337	0.464362	0.998356
Zfp780b	338354	-0.32284	0.998356
Fkbp15	338355	-0.01787	0.998356
Supv3l1	338359	-0.15659	0.998356
Trim65	338364	-0.60233	0.998356
Slc41a2	338365	0.180199	0.998356
Mia3	338366	0.275957	0.998356

Myo1d	338367	0.437988	0.998356
A730011L01Rik	338371	0.054125	0.998356
Map3k9	338372	0.184191	0.998356
Morc3	338467	0.334035	0.998356
Fa2h	338521	0.780544	0.998356
Jhdm1d	338523	0.074868	0.998356
Agap1	347722	0.294048	0.998356
2900097C17Rik	347740	-0.10029	0.998356
Plekhm1	353047	-0.10706	0.998356
Egfl7	353156	3.18031	0.998356
Txlng	353170	-0.156	0.998356
Gars	353172	-0.32016	0.998356
Nr1d2	353187	0.505204	0.998356
Edc3	353190	-0.05919	0.998356
Zfp931	353208	0.000439	0.998356
Mrpl21	353242	-0.35533	0.998356
Ltv1	353258	0.100302	0.998356
Zfp703	353310	0.555437	0.998356
Peg13	353342	-0.17401	0.998356
Tmc4	353499	0.094267	0.998356
Hcfc1r1	353502	0.120544	0.998356
Myo18a	360013	0.081951	0.998356
Hist1h3a	360198	-0.50573	0.998356
Trim46	360213	-0.8823	0.998356
Zranb1	360216	0.291696	0.998356
Khdc1a	368204	0.229532	0.998356
Morn2	378462	-0.29046	0.998356
Gm10033	378466	0.233933	0.998356
Serf2	378702	0.104674	0.998356
Raet1e	379043	0.121468	0.998356
Fastkd5	380601	0.400791	0.998356
Tagap1	380608	0.174383	0.998356
Intu	380614	-0.07917	0.998356
Heca	380629	0.429643	0.998356
Lemd3	380664	0.185115	0.998356
Rdh18-ps	380674	0.760746	0.998356
Ccnjl	380694	0.063725	0.998356
Tmem102	380705	0.462614	0.998356
Rap1gap2	380711	0.084666	0.998356
Tlcd2	380712	-0.28689	0.998356
Rph3al	380714	0.028835	0.998356
Tssc1	380752	0.23321	0.998356
Atxn7l1	380753	-0.12609	0.998356
1810035L17Rik	380773	-0.55376	0.998356
Mrs2	380836	-0.30678	0.998356
Lyrm4	380840	-0.13337	0.998356
Rsl1	380855	0.087164	0.998356

Tmem171	380863	-0.5319	0.998356
Lrch1	380916	-0.24555	0.998356
Lmo7	380928	0.515043	0.998356
Alg10b	380959	-0.00814	0.998356
Tmem106c	380967	0.593412	0.998356
Higd1c	380975	1.39778	0.998356
Zfat	380993	-0.52134	0.998356
Parl	381038	0.06571	0.998356
Ccdc58	381045	0.655713	0.998356
Zfp948	381066	-0.03805	0.998356
Zfp229	381067	0.109918	0.998356
Tbc1d22b	381085	-0.03882	0.998356
Fam82a1	381110	-0.1554	0.998356
Capn13	381122	0.356946	0.998356
Fam59a	381126	0.108687	0.998356
Gm962	381201	0.058014	0.998356
Xkr9	381246	0.351393	0.998356
Als2cr4	381259	0.003645	0.998356
Hjurp	381280	-0.21736	0.998356
Kif14	381293	-0.26835	0.998356
Rc3h1	381305	-0.13602	0.998356
Iars2	381314	0.49244	0.998356
Nsl1	381318	-0.72333	0.998356
5930434B04Rik	381356	-0.07868	0.998356
Med19	381379	-0.32619	0.998356
Zfp408	381410	0.406998	0.998356
Gm5148	381438	-0.54494	0.998356
S100a7a	381493	0.877802	0.998356
Dpy19l4	381510	0.198614	0.998356
Pdp1	381511	0.457469	0.998356
AI427809	381524	-0.48883	0.998356
2610005L07Rik	381598	-0.39415	0.998356
Tbc1d2	381605	0.421147	0.998356
Rbm33	381626	-0.0302	0.998356
0610007C21Rik	381629	-0.09241	0.998356
Cep135	381644	-0.25448	0.998356
Thap6	381650	-0.07	0.998356
Gm1045	381651	-0.76948	0.998356
Fbrsl1	381668	0.409355	0.998356
B3galtl	381694	0.188508	0.998356
N4bp2l2	381695	0.305426	0.998356
Ssbp1	381760	0.045791	0.998356
2310040G24Rik	381792	-0.79303	0.998356
Tatdn2	381801	0.472065	0.998356
Tsen2	381802	-0.69371	0.998356
Lpar5	381810	-0.25158	0.998356
Efcab4b	381812	-1.13846	0.998356

2700089E24Rik	381820	-0.06231	0.998356
1190002F15Rik	381822	-0.93632	0.998356
Alg8	381903	-0.59067	0.998356
Taok2	381921	-0.05651	0.998356
Zbtb2	381990	0.0491	0.998356
Tmem188	382030	0.353795	0.998356
Gse1	382034	0.257952	0.998356
Urb2	382038	0.0359	0.998356
Pdp2	382051	-0.08891	0.998356
Crtc1	382056	-0.22045	0.998356
Defa22	382059	-0.35943	0.998356
Prdm10	382066	0.015817	0.998356
Ccdc84	382073	0.563997	0.998356
4922501C03Rik	382090	0.029349	0.998356
D9Ertd402e	382117	-0.21453	0.998356
Zfp167	382118	-0.33797	0.998356
Fdxacb1	382137	0.5442	0.998356
Phf16	382207	0.356407	0.998356
Brwd3	382236	-0.08446	0.998356
Tmem29	382245	0.067485	0.998356
A830080D01Rik	382252	-0.22807	0.998356
Cdkl5	382253	-0.78948	0.998356
Poc1b	382406	-0.11598	0.998356
Gm5176	382421	0.881777	0.998356
Atxn7l3b	382423	-0.05032	0.998356
Hist3h2bb-ps	382522	-0.04737	0.998356
Zbtb42	382639	0.202043	0.998356
Rrm2b	382985	0.165769	0.998356
Ypel5	383295	0.480767	0.998356
Aim2	383619	0.024081	0.998356
Gm1332	383766	-0.36198	0.998356
Glipr2	384009	0.228136	0.998356
Ephx4	384214	1.05719	0.998356
Gatc	384281	-0.03123	0.998356
Trim56	384309	0.315398	0.998356
Irs2	384783	0.111427	0.998356
Gm1943	384864	-0.42854	0.998356
Pglyrp4	384997	0.989236	0.998356
Fam55c	385658	-0.30145	0.998356
Rnf39	386454	0.676887	0.998356
Thoc6	386612	0.174321	0.998356
Nsfl1c	386649	-0.55381	0.998356
Znrf2	387524	0.316942	0.998356
Zhx2	387609	-0.19017	0.998356
Mettl7a2	393082	0.919573	0.998356
Ugt1a7c	394432	-0.33483	0.998356
Map4k5	399510	0.075795	0.998356

Btbd6	399566	-0.02148	0.998356
BC052040	399568	-0.6403	0.998356
Fam84b	399603	-0.04203	0.998356
Opa3	403187	0.090567	0.998356
H2afy2	404634	-0.55045	0.998356
Iqgap3	404710	-0.0522	0.998356
Bex4	406217	0.254369	0.998356
Tmem189	407243	0.206374	0.998356
Ndufs6	407785	0.580878	0.998356
Taf9b	407786	-1.59625	0.998356
Ndufa4l2	407790	0.242349	0.998356
BC030870	407795	0.350477	0.998356
BC031181	407819	0.156202	0.998356
Znrf3	407821	0.329367	0.998356
Baz2b	407823	-0.03488	0.998356
Zfp873	408062	0.195535	0.998356
Zfp738	408068	0.091015	0.998356
Tnip3	414084	0.344806	0.998356
D330050I16Rik	414115	-0.57411	0.998356
Zyg11b	414872	-0.07154	0.998356
Akap7	432442	0.005247	0.998356
Gm5424	432466	0.294112	0.998356
Hnrnph3	432467	-0.44626	0.998356
Gnptab	432486	-0.01942	0.998356
Cpsf6	432508	-0.18801	0.998356
Myo1a	432516	0.311275	0.998356
Adcy1	432530	-0.079	0.998356
Specc1	432572	0.333986	0.998356
Akr1c19	432720	-0.15341	0.998356
Zfp187	432731	-0.07463	0.998356
Gprin2	432839	-0.1312	0.998356
Fam105b	432940	0.512163	0.998356
Gm5480	432995	-2.41691	0.998356
Plcxd2	433022	-0.28454	0.998356
Gm5485	433023	-0.73634	0.998356
Cyp2c68	433247	-0.17771	0.998356
Acsl5	433256	-0.01248	0.998356
Sgpp2	433323	0.026448	0.998356
Creg1	433375	0.305741	0.998356
Gm13375	433408	0.321407	0.998356
Jmjd7	433466	0.324743	0.998356
Maml3	433586	-0.12001	0.998356
Ankrd13c	433667	-0.29553	0.998356
Akirin2	433693	0.129932	0.998356
Ncbp1	433702	-0.15429	0.998356
Hdac1	433759	0.129846	0.998356
Minos1	433771	-0.05688	0.998356

Pusl1	433813	-0.4271	0.998356
AI506816	433855	-0.7817	0.998356
Nom1	433864	-0.16587	0.998356
Ociad2	433904	0.164141	0.998356
Lrrc8b	433926	-0.09183	0.998356
Pigg	433931	-0.37714	0.998356
Heatr2	433956	-0.24901	0.998356
Whamm	434204	0.360692	0.998356
Nlrc5	434341	-0.3819	0.998356
Gm5617	434402	-0.10555	0.998356
Amt	434437	-0.49441	0.998356
Sp140	434484	0.088171	0.998356
Gm5643	434858	0.908196	0.998356
Shf	435684	0.005975	0.998356
Snora74a	436583	0.61367	0.998356
D830031N03Rik	442834	-0.19126	0.998356
Nup85	445007	-0.04813	0.998356
Znhit3	448850	0.563689	0.998356
Zfp960	449000	0.111959	0.998356
Zfp213	449521	-0.41072	0.998356
Snord15a	449630	2.89245	0.998356
Snord15b	449631	-0.3147	0.998356
Zbtb9	474156	0.047187	0.998356
Cbx6	494448	-0.2368	0.998356
Armcx5	494468	-0.20548	0.998356
Defa23	497114	1.63997	0.998356
Acd	497652	-0.04403	0.998356
Defa24	503491	-0.09689	0.998356
Zdhhc18	503610	0.079856	0.998356
1190007I07Rik	544717	0.172944	0.998356
Tug1	544752	-0.00519	0.998356
Arhgap27	544817	-0.20772	0.998356
Bdp1	544971	-0.21909	0.998356
Gm5801	545056	0.345969	0.998356
Wdr70	545085	-0.13001	0.998356
Arsi	545260	-0.4211	0.998356
Cep170	545389	-0.22457	0.998356
Ptpn3	545622	0.308078	0.998356
Mterf	545725	0.287431	0.998356
Zfp607	545938	-0.37631	0.998356
Lass3	545975	-0.23463	0.998356
Mast3	546071	-0.16068	0.998356
Gm5918	546143	0.275972	0.998356
6820431F20Rik	547150	-0.25252	0.998356
Parp14	547253	0.193484	0.998356
Zmynd15	574428	-0.21757	0.998356
Gsk3a	606496	0.028364	0.998356

A330069E16Rik	606735	-0.70208	0.998356
A730017L22Rik	613258	-0.31247	0.998356
BC029722	613262	-0.38441	0.998356
G630016D24Rik	619301	0.387278	0.998356
Rpl34-ps1	619547	3.62057	0.998356
Zcchc17	619605	0.02242	0.998356
Gm12669	620016	-0.45802	0.998356
Siglec15	620235	-0.0237	0.998356
Gm13889	620695	-0.31036	0.998356
Aldh3b2	621603	0.393396	0.998356
Gm6251	621697	1.27526	0.998356
Hist2h2ab	621893	-1.14208	0.998356
Tmem170b	621976	0.205927	0.998356
Kctd21	622320	-0.30673	0.998356
Akr1c12	622402	0.603435	0.998356
Ccdc107	622404	0.316504	0.998356
Arhgef26	622434	-0.07321	0.998356
Zfp827	622675	-0.40263	0.998356
Gm6402	623169	-0.48209	0.998356
Rad54b	623474	-0.03485	0.998356
Lipt1	623661	-0.53921	0.998356
Gm14137	623781	0.095599	0.998356
Gm6484	624219	0.718746	0.998356
Btnl6	624681	0.383624	0.998356
Gm6548	625054	-0.13405	0.998356
Gpx4	625249	1.05468	0.998356
Fam23a	625286	0.58369	0.998356
Gm6644	626009	0.663284	0.998356
Gm6654	626175	-0.73913	0.998356
Defa26	626708	0.257528	0.998356
Etohi1	626848	-0.07198	0.998356
Zfp800	627049	0.039134	0.998356
Gm6793	627828	-0.93156	0.998356
Gm14420	628308	-0.47263	0.998356
Fam124a	629059	0.249056	0.998356
1700008J07Rik	629159	-0.49405	0.998356
2010315B03Rik	630836	0.232837	0.998356
Cyp4f40	631304	-0.30098	0.998356
Gm12250	631323	-0.78811	0.998356
Btnl4	632126	0.855859	0.998356
Gm7120	633640	-1.11462	0.998356
Susd1	634731	0.003712	0.998356
Gm14851	634825	-1.50638	0.998356
9530082P21Rik	638247	0.52452	0.998356
Nrbf2	641340	-0.25536	0.998356
2310033E01Rik	641361	0.418557	0.998356
Tomm40l	641376	0.333716	0.998356

4930420K17Rik	652925	0.60577	0.998356
Nrp	654309	-0.45453	0.998356
Gm7334	654432	0.275469	0.998356
Gm10052	654467	-0.95947	0.998356
Sdr39u1	654795	0.08812	0.998356
Zfp784	654801	-0.118	0.998356
Ankrd37	654824	0.429461	0.998356
Gm7367	664849	0.815075	0.998356
2210411K11Rik	664968	0.095357	0.998356
Isoc2a	664994	0.800463	0.998356
Tnik	665113	-0.11415	0.998356
Gm14326	665211	0.08931	0.998356
Hist1h2ao	665433	2.70819	0.998356
Mthfd2l	665563	-0.06801	0.998356
Bod1l	665775	0.087999	0.998356
Rplp2-ps1	665931	-0.01957	0.998356
Vps13b	666173	0.088976	0.998356
Tmsb15b1	666244	0.151936	0.998356
Zfp498	666311	0.507254	0.998356
Atg4a	666468	-0.13382	0.998356
Samd1	666704	0.403368	0.998356
4632427E13Rik	666737	-0.14476	0.998356
Rbm24	666794	-0.28227	0.998356
Gm12191	666899	2.21056	0.998356
Pnp2	667034	0.592852	0.998356
Zbed6	667118	-0.10108	0.998356
Pex10	668173	-0.26754	0.998356
Mettl7a3	668178	-0.31895	0.998356
Zfp507	668501	-0.0366	0.998356
2410002F23Rik	668661	0.256786	0.998356
Parp10	671535	0.593643	0.998356
Zfp605	675812	0.149818	0.998356
Sap25	751865	0.860789	0.998356
Gm11428	1E+08	0.642017	0.998356
Mfap1b	1E+08	0.254498	0.998356
Gm15772	1E+08	-0.03974	0.998356
Gm16039	1E+08	-0.00104	0.998356
Dnajc3	1E+08	0.11408	0.998356
Rsph3b	1E+08	0.020313	0.998356
Fam174b	1E+08	-0.15803	0.998356
Gm10768	1E+08	0.035555	0.998356
Gm10516	1E+08	0.872784	0.998356
Gm15401	1E+08	0.213053	0.998356
Gm1976	1E+08	0.078697	0.998356
Gm15284	1E+08	-0.30708	0.998356
Gm14295	1E+08	-0.22331	0.998356
Gm15706	1E+08	0.28249	0.998356

Tgtp2	1E+08	0.004429	0.998356
Snhg12	1E+08	0.260835	0.998356
Gm12942	1E+08	-0.39259	0.998356
Dynlt1f	1E+08	-0.69555	0.998356
Dynlt1c	1E+08	-0.0569	0.998356
Hist1h4m	1E+08	-0.47883	0.998356
Gm11974	1E+08	-0.33886	0.998356
Gm11978	1E+08	0.678889	0.998356
Ly6c2	1E+08	-1.00276	0.998356
Gm3414	1E+08	-0.18613	0.998356
Amd2	1E+08	-0.78527	0.998356
Gm13157	1E+08	0.141129	0.998356
Gm10104	1E+08	1.72396	0.998356
Gm10451	1E+08	-0.60858	0.998356
Gm15293	1E+08	0.519369	0.998356
Gm15308	1E+08	0.089505	0.998356
Gm14850	1E+08	1.44928	0.998356
Gm15315	1E+08	-0.23136	0.998356
Gm10094	1E+08	-0.55248	0.998356
Gm3604	1E+08	-0.96365	0.998356
BC005561	1E+08	0.125484	0.998356
Rps15a-ps6	1E+08	-0.66847	0.998356
2610203C20Rik	1E+08	-0.92081	0.998356
Gm9781	1E+08	-0.21333	0.998356
9130023H24Rik	1E+08	-0.0479	0.998356
Gm4285	1E+08	0.331198	0.998356
5430417L22Rik	1E+08	0.080731	0.998356
Gm14005	1E+08	1.21347	0.998356
Nutf2-ps1	1E+08	-0.92911	0.998356
Zfp955b	1E+08	-0.00699	0.998356
1300002E11Rik	1E+08	-0.05287	0.998356
Gm4532	1E+08	-0.33732	0.998356
AI662270	1E+08	-0.68184	0.998356
Gm9846	1E+08	-0.42574	0.998356
R3hdml	1E+08	-0.07576	0.998356
Gm14378	1E+08	1.07623	0.998356
Gm20571	1E+08	-0.86459	0.998356
Srp54c	1E+08	0.232378	0.998356
Adat3	1E+08	-0.14456	0.998356
Krt83	1E+08	0.465616	0.998356
Sco2	1E+08	0.928667	0.998356
Snord22	1E+08	1.54226	0.998356
Gm11110	1E+08	0.137163	0.998356
Scarna6	1E+08	2.42123	0.998356
Snora44	1E+08	10	0.998356
Snora52	1E+08	-0.00206	0.998356
Snora81	1E+08	-0.02746	0.998356

AK010878	1E+08	-0.46351	0.998356
Gm16119	1E+08	-0.38901	0.998356
Snora21	1E+08	-1.72057	0.998356
5830417I10Rik	1E+08	-0.23366	0.998356
Gm12238	1E+08	3.69196	0.998356
Scarna13	1E+08	-0.01194	0.998356
Snora78	1E+08	10	0.998356
Gm10509	1E+08	0.060382	0.998356
Snora16a	1E+08	2.31995	0.998356
Dynlt1a	1E+08	0.311234	0.998356
Snord17	1E+08	1.84845	0.998356
Gm20605	1E+08	-0.96893	0.998356
Snora23	1E+08	0.584759	0.998356
Gm15545	1.01E+08	-1.0649	0.998356
1700021K19Rik	1.01E+08	0.034828	0.998356
1700007L15Rik	1.01E+08	0.378582	0.998356
Kifc1	1.01E+08	-0.06387	0.998356
5430411K18Rik	1.01E+08	0.220792	0.998356
Gm3336	1.01E+08	0.045006	0.998356
Gm17066	1.01E+08	0.115437	0.998356
2810013P06Rik	1.01E+08	-0.25955	0.998356
5430416N02Rik	1.01E+08	0.404748	0.998356
Snhg4	1.01E+08	-0.52448	0.998356
LOC100503496	1.01E+08	-0.14128	0.998356
Bbip1	1.01E+08	-0.25518	0.998356
Dos	1.01E+08	-0.45563	0.998356
Rpl5	1.01E+08	-0.0707	0.998356
Gm16861	1.01E+08	0.040154	0.998356
Ccdc149	1.01E+08	0.30457	0.998356
2900053A13Rik	1.01E+08	-0.19564	0.998356
Gm10345	1.01E+08	-0.68713	0.998356
1810008I18Rik	1.01E+08	-0.17713	0.998356
Gm16907	1.01E+08	0.148326	0.998356
2010003O02Rik	1.01E+08	0.131938	0.998356
Gm1987	1.01E+08	0.63745	0.998356
Gm20199	1.01E+08	-0.35128	0.998356
Gm20300	1.01E+08	0.219479	0.998356
Atg14	1.01E+08	-0.11536	0.998356
Tmppe	1.01E+08	-0.10187	0.998356
Raver1-fdx1l	1.01E+08	1.19853	0.998356
0610007N19Rik	1.01E+08	0.036601	0.998356
9330133O14Rik	1.01E+08	0.21689	0.998356
Mira	1.01E+08	0.300416	0.998356
Gm20604	1.01E+08	0.095194	0.998356
Plin2	1.01E+08	0.03871	0.998356
Ppp1cc	19047	-6.3E-05	0.998709
Rap2a	76108	0.00059	0.998709

Galnt2	108148	0.000534	0.998709
Tpr	108989	-0.00144	0.998709
Psmb3	26446	-0.0009	0.998724
Gm608	207806	-0.00117	0.998724
Cobl	12808	0.001028	0.998732
2210016F16Rik	70153	0.000932	0.998732
Slc18a1	110877	0.000182	0.998732
Prkci	18759	0.000173	0.998977
Map3k2	26405	-0.00012	0.998977
Zfp260	26466	1.09E-05	0.998977
Kif23	71819	0.000864	0.998977
Dusp4	319520	-0.00074	0.998977
Txndc9	98258	0.00035	0.999049
Rfwd3	234736	-1.7E-05	0.99912
Zmym2	76007	-0.00027	0.999135
Golgb1	224139	-0.00045	0.999135
Brd8	78656	-0.00056	0.999157
Kitl	17311	-0.00018	0.99955
